# Advancing Porous Carbons: Understanding the Importance of Surface Chemistry for the Energy–Environment Nexus

**DOI:** 10.1021/acs.chemrev.5c00719

**Published:** 2026-02-05

**Authors:** Conchi Ania, Teresa J. Bandosz, Diego Cazorla-Amorós, M. Fernando R. Pereira

**Affiliations:** † 129849 CNRS (CEMHTI, UPR 3079), Université d’Orléans, 45071 Orléans Cedex 2, France; ‡ Department of Chemistry and Biochemistry, 14770 The City College of the City University of New York, New York, New York 10031, United States; § Faculty of Chemistry, Maria Curie Sklodowska University, 20-031 Lublin, Poland; ∥ Department of Inorganic Chemistry and Materials Institute, University of Alicante, Ap. 99, Alicante E-03080, Spain; ⊥ LSRE-LCM, ALiCE, Faculty of Engineering, University of Porto, Rua Dr. Roberto Frias, 4200-465 Porto, Portugal

## Abstract

This review intends, in a critical way, the comprehensive view of the importance of porous carbons surface chemistry for their applications in an energy–environment nexus. Surface chemistry is presented as a combination of functional heteroatom-containing groups, dopants, and structural defects. First, we briefly address carbon surface chemical environment and the methods of its modification and characterization, indicating their practical limitations. Then, the effects of surface chemistry on separation, catalysis, energy storage, sensing and microwave absorption are introduced. Besides a critical analysis of published findings on these topics, we also include our views on the advancement in the processes which rely on porous carbons surface chemistry, and identify strategic areas and directions that should deserve further attention. We focus on new findings and important original contributions to the field. Since the community of carbon researchers grows following the strategic application of these materials, the role of functional groups, dopants and structural defects in various cutting-edge applications is emphasized, showing the progress in the field and the evolution of findings. A clear determination of the effects of carbon surface is often a challenge since carbons porosity and the locations of specific bonds/sites/defects in the carbon texture provide nanoconfinement effects.

## Graphical abstract

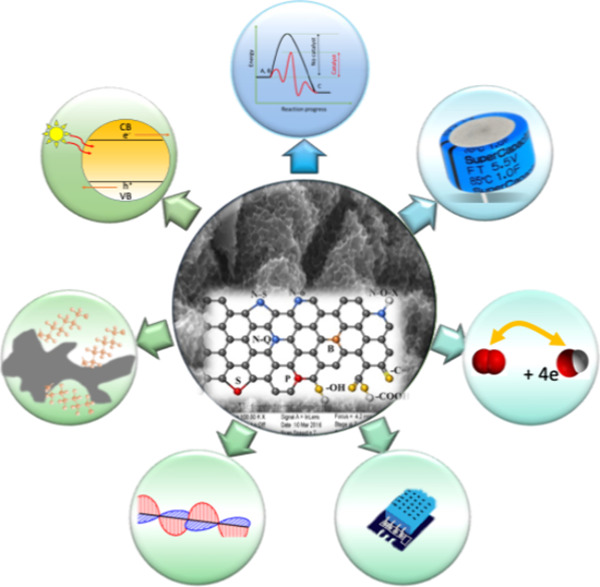

## 1 Introduction

Porous carbons are unique materials that nowadays play a major role in a variety of specialized applications ranging from gas adsorption and separation, environmental remediation, catalysis, energy conversion and storage, sensing, medical healing, cosmetic supplement production, soil amendments, radiation shielding, and many others. They are among the oldest materials used by humankind and some sources list their usage as early 3750 BC for reduction of metals by Egyptians and Sumerians.^1^ With the progress of time and increasing knowledge of natural sciences, charcoal was applied for water purification and as a primitive medical remedy. A real progress in the application of carbon materials as adsorbents happened after R. Von Ostreyko published his methods of charcoal activation starting in 1900.^2,3^ That development of porosity visibly broadened the applications of activated carbons for various separations from gas and liquid phases, benefiting humankind. An example is the use of gas mask filters during World War I.^4^

Despite broad categories of nanoporous materials (e.g., zeolites, metal–organic frameworks, silicas, polymers) synthesized in the past decades, research on porous carbons continues to be in a mainstream. While activated carbons provide a heterogeneity of pore sizes, controlled only to a relatively narrow extent by choosing activation methods, more structurally homogeneous porous carbons are nowadays produced using more “predesign” engineering approaches. Examples are polymer-derived porous carbons,^5^ template-synthesized ones^6^ or those based on a combination of various carbon allotropes.^7^

A broad variety of applications stems from the combination of porous carbons unique textural features (such as high specific surface areas, varied pore architectures and pore size distributions, modulated upon the choice of a precursor and a synthesis route), and the existence of a superficial layer of chemically bonded elements. The latter is due to their ability to react with heteroatoms or other carbon atoms (incorporating them to the surface or within the carbon matrix), leading to the development of multifunctional materials with unique properties. Both characteristics of porous carbons (surface chemistry and pore architecture) determine their self-organization, chemical stability, and reactivity.

From a structural viewpoint, porous carbons are mainly composed of distorted graphene-like layers of sp^2^ domains of carbon atoms that are rich in defects, including dangling bonds due to imperfections of graphene structure. These distorted graphene layers are typically stacked by weak van der Waals forces in a turbostratic structure, creating voids (pores) at a nanometric scale. In many cutting-edge carbon applications, the existence of specific pore sizes is an important feature. Besides, knowledge of surface chemistry of carbon materials is critical since the presence of heteroatoms located at the edges or inserted into basal-planes defines the reactivity and self-organization of porous carbons, ultimately controlling their performance in a target application. As a few examples, it is a well-known that even small amounts of heteroatoms can exert a significant influence on the electrochemical performance of carbon electrodes,^8^ as they impact the hydrophilic/hydrophobic character, wettability, reactivity or electronic conductivity. Likewise, these heteroatoms can favor/hinder the uptake of a target probe (gas or an adsorptive in an aqueous solution) due to the occurrence of specific interactions between the adsorbate and those surface groups. Recent studies have demonstrated that specific interactions of CO_2_ with polar oxygen groups may lead to an overestimation in the pore volume when characterizing the textural properties of those carbons.^9^ In the field of catalysis, whether the carbon is used as a catalyst support or as a catalyst itself, numerous works have focused on the role of surface chemistry with respect to the dispersion of catalyst sites, their form, including a recently emphasized single atom arrangement, or catalytic activity.

Since nowadays there are almost infinite possibilities of the modification of carbon surface chemistry, modified carbons provide an unlimited, imaginative and simple resources to face newly arisen environmental and energy related challenges. Recent years have demonstrated that porous carbons continuously attract researchers from a broad range of disciplines, and therefore their surface chemistry has become a topic of increasing interest. Despite this, little attention is devoted to a targeted design and a proper characterization of these materials (from a chemical point of view) and to analyze and understand the role of surface chemistry. What is more troubling is that the common and well established (old) knowledge about this topic seems to be neglected or forgotten in recent publications by researchers who have just started to explore these materials for their new applications. Even though well-cited reviews have been published on this topic in the past,^8,10–15^ new advances in science and a rapid development of new analytical methods justify readdressing the chemistry of porous carbons with emphasis on strategically important applications.

Owing to a broad variety of carbon allotropes and very rich literature on their properties and applications, we limit this review to porous carbons and their position in an energy–environment nexus. As porous carbons, we consider those carbon materials with a vast majority of carbon element in their structural units and having pores of various sizes (mainly micro- and mesopores of sizes less than 2 nm and less than 50 nm, respectively), important for specific target applications. The significance of the latter can be either in a contribution to a mass transfer, in providing storage of molecules through the variety of adsorption forces (fluid–solid interactions), or through promoting chemical/catalytic reactions governing the intended processes.

There is a general perception that diverse porous carbon application fields often do not cross their exploration paths, which does not benefit the modern-age application of the carbon materials, in general. Some fields, as for instance those addressing the activation of chars and synthesis of carbons from polymers, catalysis and radiation shielding, separation and sensing or energy storage and soil amendments, remain estranged in various aspects. Even though there are excellent review papers and books on characteristics and applications of carbon materials, they often remain specific to the subdiscipline of carbon science which they address,^5,16–19^ unintentionally further isolating carbon research fields. As carbon surface chemistry we consider not only heteroatom-containing functional groups but also heteroatoms doped within carbons rings, and structural/topological defects in the carbon matrix, affecting an overall electronic structure and conductivity and thus the performance of carbon materials. Besides briefly addressing the carbon surface chemical environment and the methods of its modification and characterization, we focus on separation, catalysis, energy storage, sensing and microwave absorption. The surface chemistry of carbon is still the chemistry as a well-defined scientific discipline. Particularly in the case of carbon materials, Carbon-X element bonds/arrangements stay the same regardless the application discipline. Thus, by providing this review, we attempt to reach a broad range of porous carbon researchers struggling to reveal the role of functional groups and dopants in various applications. In the applications that we address in this work carbon porosity plays an important role, and thus the locations of specific bonds/sites in a carbon texture is a factor which affects the performance by nanoconfinement effects. This brings a challenge to understand the behavior of a specific system and to design carbons with an optimal performance. And this challenge remains the same for those addressing the application of carbons from polymers and biomass and those advancing the adsorption of organic pollutants or behavior of charged ions in the carbon pore system. To provide the current view on these specific carbon fields, we summarize the recent findings (about half of the references are from the last 5 years) and emphasize the actual developments in the context of the research progress over the years.

## 2 Carbon Surface Chemical Environments

The surface chemistry, defects and edge sites in carbon materials have been in the spotlight for several decades because of their uncertain nature and also because they are known to govern many key features of the carbon materials (such as reactivity, electron-transfer rates, capacitance, optical, mechanical or magnetic properties, among others).^20,21^ While the different chemical reactivity of bulk graphitic basal planes and edge sites has been widely studied,^22–26^ the interest in the electronic properties of defects and edges of carbon materials has grown in the last years, and the formation of surface defects has proven to be a useful strategy to create and improve catalytic sites in carbons in various application fields.^20,27–29^

The lack of a fundamental understanding of surface chemistry, defects and edge sites has made it difficult to rationally design of engineered approaches to obtain carbon materials with high density and high-performance defect sites. To understand the complexity of this task, we must consider that carbon atoms can form a variety of structures, owing to the versatility of their hybridization states. At the atomic scale, the surface chemistry of sp^2^ carbons is mainly constituted of carbon atoms arranged in a hexagonal network with varying degrees of planarity and dimensionality, forming the basal planes and the edges (the so-called graphitic like structures). Along this structure, functionalization and doping can create modifications in the electronic and chemical properties of the sp^2^ hexagonal arrangement, resulting in different defects (Figure 1).

**Figure 1:**
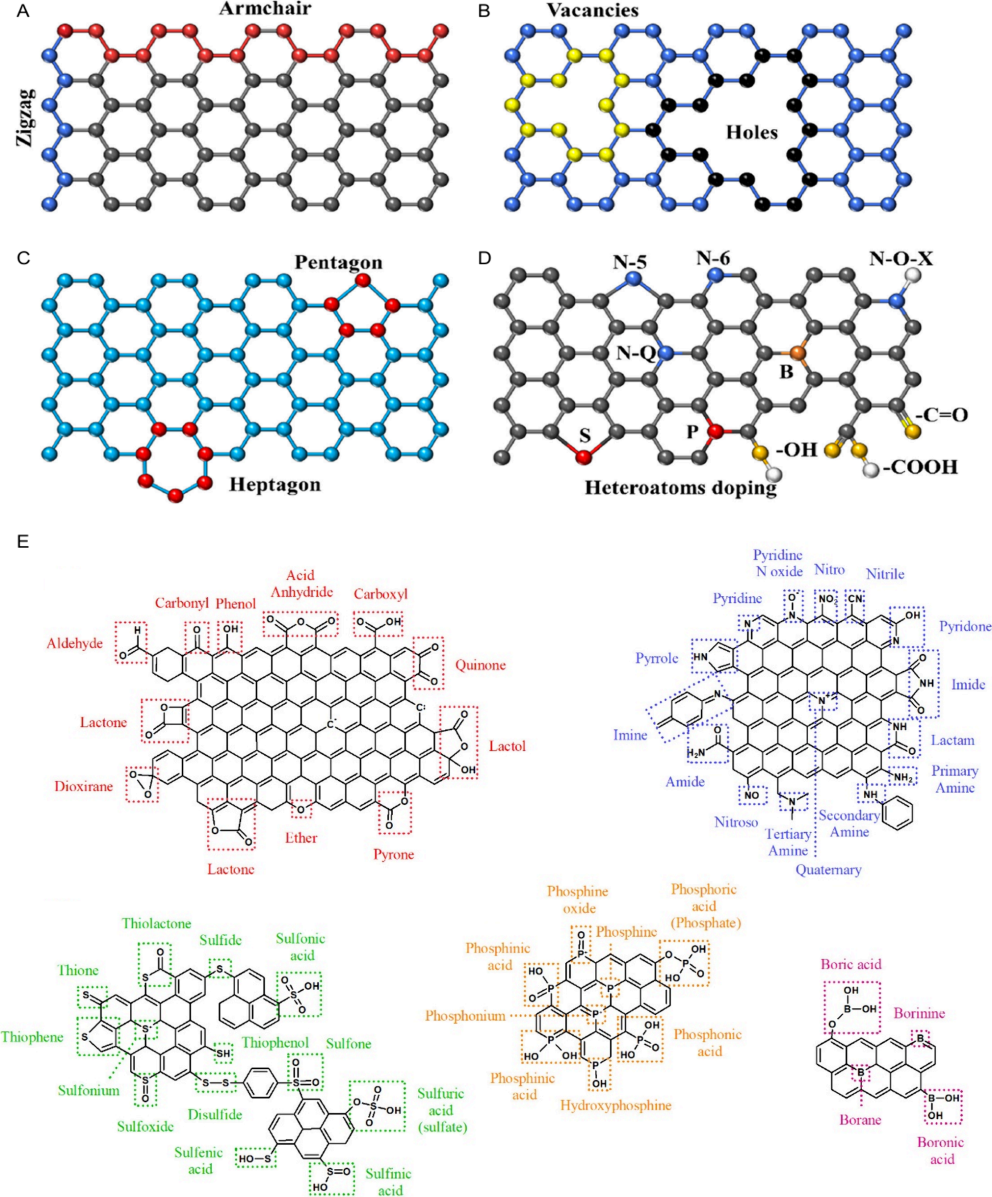
Schematic representation of the (A–C) topological (intrinsic) and (D) extrinsic defects on carbon surfaces. Reproduced with permission from ref.^28^ Copyright 2022, John Wiley and Sons; (E) scheme illustrating different functional groups on the surface of carbon materials. Reproduced with permission from ref.^30^ Copyright 2022, Elsevier.

These defect sites are more active than are the aromatic carbon atoms in the basal planes, owing to a high density of unpaired electrons. Lattice distortions, carbon vacancy defects, non-hexagonal arrangements and edge defects are commonly referred to as intrinsic defects (e.g., abnormal conjugated networks), while heteroatom functionalization and doping-induced defects (including metallic and not metallic) are considered as extrinsic defect sites.^28,31,32^ The modification of the surface chemistry of carbon materials upon the incorporation of non-metal heteroatoms has been largely investigated,^10,15,33^ but the interest for the intrinsic defects in carbons has grown in the past few years triggered by the need to understand the performance of defect-rich carbons.^25,34–36^

### 2.1 Intrinsic Defects

In general terms, topological defects refer to distortions of the carbon lattice due to the presence of non-hexagonal arrangements and dangling bonds, such as pentagons, Stone–Wales defects or vacancies. Vacancies are caused by the removal of one (single vacancy) or more carbon atoms (di- and multivacancies) from a graphene-like layer, and are usually accompanied by edge sites. The loss of carbon atoms induces a creation of dangling bonds (unpaired electrons in a carbon atom) and a deformation of the structure due to the modification of bond lengths. Those unpaired electrons are responsible for the magnetic moment of vacancy defects. Stone–Wales (SW) defects are formed by modifying the connectivity of two bonded carbon atoms, which results in non-hexagonal ring structures (pentagon–heptagon, octagon–pentagon pairs).^37,38^ Topological defects are ubiquitous in porous carbons as they are inevitably introduced during thermal rearrangement reactions (carbonization, gasification, graphitization),^37^ but they are difficult to observe due to the 3D structure of porous carbons.

Experimental and theoretical studies have demonstrated that to obtain a complete understanding of the performance of porous carbons as adsorbents or catalysts, a deep analysis of the topological defects should be carried out along with the (traditional) characterization of the nanoporous features (pore volume, average pore size). Topological defects are not only involved in the formation of nanopores, but can also create new adsorption/reactive sites. As an example, several studies have reported that pentagon rings in SW defects exhibit stronger CO_2_ adsorption than seven- and eight-membered rings due to a higher atomic charge density.^32,39^

Yet, a rational design of the appropriate topological defects to control performance remains a significant challenge. Wang et al. demonstrated the link between the electrocatalytic reduction of carbon dioxide on doped carbon materials and the presence of topological and extrinsic defects.^40^ The authors demonstrated an excellent catalytic performance for the electrochemical reduction of CO_2_ of undoped defective porous carbons owing to the presence of intrinsic defects.^40^

Paramagnetism is another property of disordered carbons that is associated with the density of the intrinsic defects and edge sites.^41–43^ Hence, electron paramagnetic resonance (EPR) can be used as a spectroscopic probe through the analysis of relaxation rates and signal shapes to determine modifications in the surface defects and edges induced upon heteroatom doping and/or adsorption. As an example, Wang et al. evidenced the roles of localized spins at defects and edge states and conduction electron spins in sp^2^ domains on the electrochemical capacitance of O- and N-doped carbons, using *in situ* electrochemical electron paramagnetic resonance spectroscopy to detect unpaired electrons.^43^

Edge sites (aka prismatic surfaces) refer to the boundaries of the carbon matrix. The most common structural edge geometries are zigzag (carbene) and armchair (carbyne) configurations,^25,44^ both of which are characterized by a large number of unpaired p-electrons. Edges (as well as vacancies) in carbon materials are mostly terminated by C–H bonds, functional groups, or other defects. Edge defects can also be caused by the lack of carbon atoms at the edges, both in zigzag and armchair configurations. The carbon atoms at edges usually display higher charge densities and chemical reactivities than those in the basal planes, with zigzag edge configurations being the most reactive.^45–49^ Numerous studies have reported that the edge defects in carbon materials play an essential role in an electrocatalytic activity, as they can modulate the electron density and charge distribution.^28,29,50^ Serp et al. have reported that prismatic surface edges are more efficient anchoring sites of single metal atoms than basal surfaces, owing to the presence of unsaturated carbon atoms.^51^

#### 2.1.1 Synthetic Strategies to Create Intrinsic Defects

Developing defect engineering approaches for obtaining carbon materials with modulated electronic properties is challenging, and numerous strategies have been explored aiming to obtain materials with homogeneous distributions of surface defects. The generation of extrinsic defects, particularly non-metallic heteroatoms, has attracted significant attention, with abundant reviews and monographs addressing the topic.

Mechanical ball-milling is an effective strategy to create warping, curvature and edge defects in carbon materials.^52,53^ Dangling bonds with unpaired electrons can also be formed through ball-milling, a strong acid treatment or upon the exposure of a carbon material to high temperatures (carbonization, graphitization), owing to the removal of surface groups located in the edges of the aromatic carbon backbone. However, due to their high reactivity, the saturation of the as-formed dangling bonds (generally as C–H bonds) is almost inevitable, for which further functionalization to create specific sites only occurs in the presence of other molecules. Interestingly, ball-milling and high temperature treatments can also repair the defective structure of the carbon network, leading to carbons with a decreased defect density by simultaneously removing the edges functional groups.^54,55^ Vacancies and edge defects are formed by removing carbon atoms via plasma, chemical and electron beam etching methods, whereas edge reconstruction methods by heteroatom removal and high temperature treatments under different atmospheres are typically applied for generating topological defects (Figure 2).^10,54,56–58^ Template-assisted methods are also an effective strategy to synthesize defective carbons with abundant edges, which are formed during the removal of a template.^59,60^ In most of these methods, the control of the nature and distribution of defects is a challenge, as they give rise to defective carbons with random defects. However, it has been reported that the decomposition of specific N- and O-functional groups can lead to a directional synthesis of specific topological defects. Zhang and Dai have reported that the decomposition of graphitic N leads to the formation of C585 divacancies, pyridinic N to separate pentagons, and pyrrolic N to adjacent pentagons.^54^ The study of Li et al. reported the generation of edge thiophene sites, pentagon defects and N–S–C active sites in N-, S-doped carbon aerogels upon the gradual decomposition of surface groups at temperatures between 600 and 1000 °C.^56^

**Figure 2:**
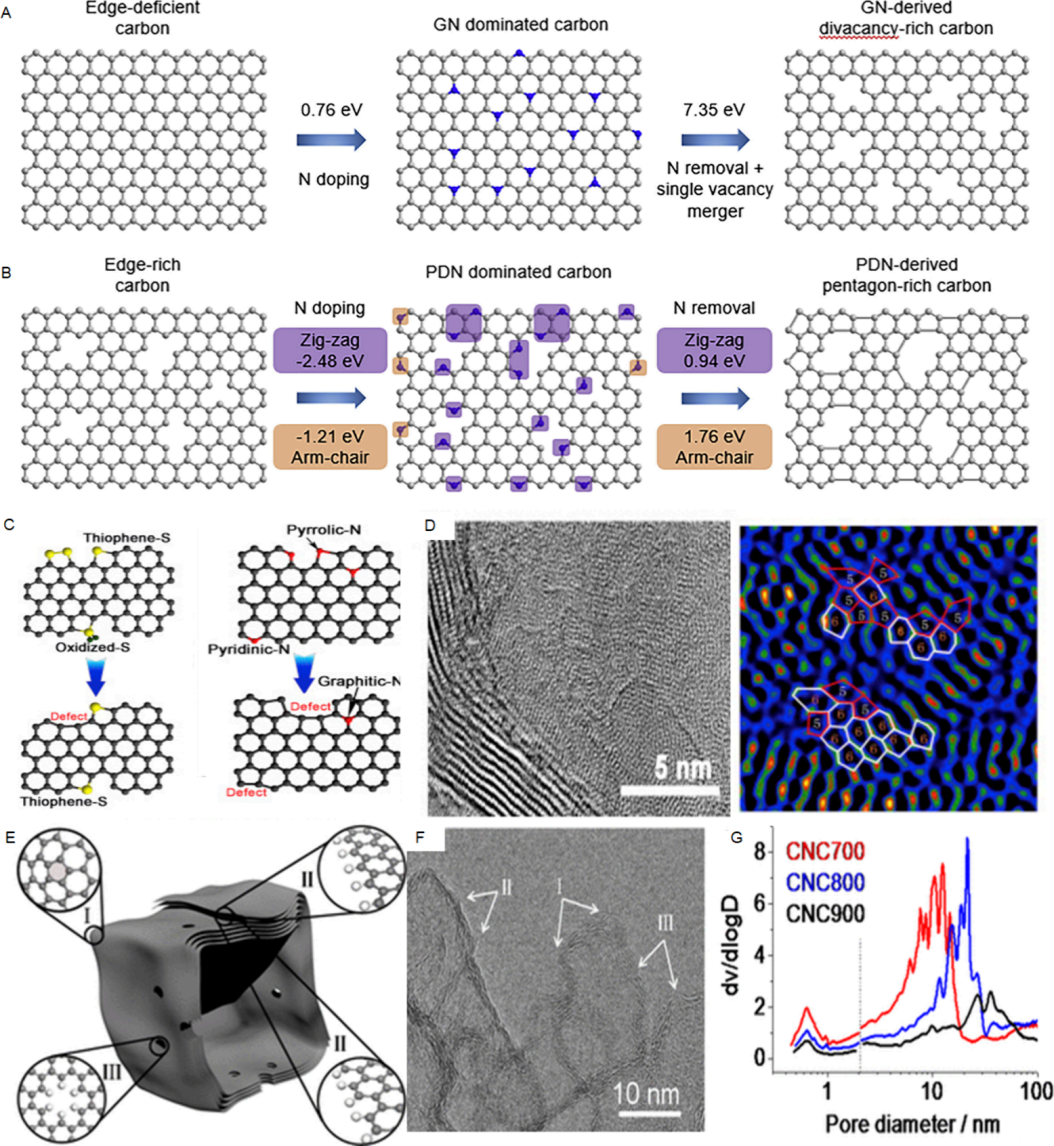
Directional defect synthesis in porous carbons: (A,B) Computational simulation of specific N-doping and removing process in different carbon models: (A) Schematic and formation energy calculation of transformation from edge-deficient carbon to GN dominated carbon and then to divacancy-rich carbon and (B) from edge-rich carbon to PDN dominated carbon and then to pentagon-rich carbon. Reproduced with permission from ref.^64^ Copyright 2020, Elsevier; C) Schematic illustration of the conversion of S- and N-heteroatom dopants through annealing treatment and D) STEM and filtered image revealing a random carbon framework structure predominating in the bulk and pentagon defects resulting from the removal of doped N- and S-groups. Reproduced with permission from ref.^56^ Copyright 2018, Elsevier; E) Schematic structural character of a carbon nanocage with typical defects (corner (I), broken fringe (II) and holes (III); F) High resolution TEM and G) pore size distribution. Reproduced from ref.^65^ Copyright 2015, the Author(s) under ACS Author Choice license.

Complex defects based on the combination of the topological defects and heteroatoms, or the confinement of metal species in carbon vacancies, can also be obtained following a similar heteroatom removal strategy at high temperatures.^56,61–63^ As an example, various configurations of Fe–N4–C were prepared involving center N- or edge N-divacancies.^63^

### 2.2 Extrinsic Defects

Extrinsic defects refer to the presence of *exotic* atoms (e.g., metals and non-metallic heteroatoms) in the carbon matrix that can react with the intrinsic defects or be adsorbed on the surface of the carbon material. The different electronegativity of those exotic atoms compared to carbon itself induces important changes in the electronic structure (charge and spin density) of the carbon material, which generally improves electrocatalytic or catalytic properties.^10,28,32,40^

#### 2.2.1 Nonmetallic Heteroatoms

Among various elements, O, N, B, S, P, and F have been extensively investigated as the modifiers of carbon surface chemical properties. They could be either incorporated as structural moieties to the graphitic matrix, with examples of nitrogen or boron in three-coordinated forms, or existing at the edges of the graphic sheets as for example ether, pyridinic, pyrrolic, hydroxyl, carbonyl, amines, cyanides, sulfones, sulfoxides, disulfides, fluorine, and phosphorus groups. Figure 1E presents a visualization of various functional groups related to the presence of heteroatoms.

The size of the heteroatoms and the difference in the electronegativity compared to that of the carbon atom control the location of the moieties (in the structure or at the edges) and the polarization, charge density and spin density distributions of the functionalized and adjacent atoms. The atomic size difference between the carbon atom and the heteroatom can induce a spatial distortion in the graphitic matrix, but this only affects the performance for very large heteroatoms, such as Br or I.

Moreover, the changes in the electronic properties determine the nature of the charge density redistribution, and thus the performance in many different reactions. For instance, doping with electronegative atoms like O and N creates the redistribution of the conjugated electronic density of the basal planes, generating a positive charge in the adjacent carbon atoms. On the other hand, doping with less electronegative atoms like B generates polarized C–B bonds with a partial positive charge on the boron atoms. These structural and electronic changes induced by the heteroatoms are responsible for modifying the adsorption and catalytic properties of the carbon materials, with numerous examples reporting the effect on the adsorption sites of strategic gases (O_2_, N_2_, CO_2_) and their catalytic conversion (ORR, CO2RR, OER, NRR).^20,66–69^ These aspects are further discussed in the next sections.

Oxygen functional groups are intrinsic to every carbon surface since they can be formed when the carbon material is in contact with the atmosphere. Surface oxygen groups are usually divided into two main groups considering their acidic-basic character. Carboxyl, lactones, lactols and hydroxyls in phenolic groups are acidic, while pyrone type structures have a basic character.^70^ The incorporation of oxygen groups is done through the conventional wet or dry oxidation methodologies, which are well described in the literature and briefly addressed below, but these methods do not provide a high selectivity toward specific groups. In the first case, the material is put in contact with a solution of an oxidizing agent and in the second case the oxidation is done with gas at mild temperatures (usually air is used) or with plasma.^71–74^ Further heat treatments can be applied to tailor, to some extent, the nature of the surface oxygen groups.

Nitrogen is one of the most studied heteroatoms in carbons because of interesting changes in the properties of these materials upon its introduction. The location of the N atom determines the structural and electronic changes. For example, pyrrole and pyridine groups, when located inside the graphene layers, generate a vacancy and quaternary N have different stabilities in a valley position or in the edge.^75–77^ It must be noted that N groups can be accompanied by oxygen atoms directly bonded to the N atom, like in pyridine, N-oxide, nitroso or nitro groups or in neighboring positions like amides or pyridone groups. These groups modify properties such as wettability, basicity, electrochemical properties and electrical conductivity, among others. Nitrogen functionalities can be generated in the carbon material through different methodologies that include:^78^ reaction with nitrogen-containing molecules, decomposition of a N-containing material that can be followed by thermal treatments and hydrothermal carbonization, among others. In these processes, the final heat treatment temperature plays a key role in the selectivity of the species formed, as discussed below.

Owing to their large atomic size (compared to C atoms), S and P functionalization also takes place mainly at the edges of the carbon matrix, mainly as sulfur oxides, sulfides, and P–O and P–C moieties. In both cases, the large charge density of these groups induces an enhanced reactivity in neighboring carbon atoms and edges through the creation of strong adsorption sites, catalytic sites, and modification of the hydrophobicity of the carbon surface.^79,80^ Phosphorus groups have been studied for many years because they reduce the reactivity of the carbon material with dioxygen, being relevant for oxidation protection.^81–83^ The properties that provide sulfur groups make them useful in heterogeneous catalysis, adsorption processes and storage and conversion of energy.^84^

One of the most used methods for functionalization with phosphorus groups is a chemical activation of a precursor (usually lignocellulosic) with H_3_PO_4_.^85–87^ Impregnation with P-containing compounds and a further heat treatment or hydrothermal methods are other frequent methods used for functionalizing carbon materials.^87,88^ Electrochemical methods can also be used for a more selective functionalization.^89,90^ The incorporation of sulfur can be done employing different S-containing reactants.^15,84^

Other less-frequently used heteroatoms can be also incorporated into the carbon material. For example, B can substitute carbon atoms in low amounts, but producing important changes in the physicochemical properties. In this sense, these species can improve a carbon material protection against oxidation, hydrogen adsorption, electrode properties in batteries and can modify electrocatalytic properties and favor graphitization processes.^91–94^ Halogens (F, Cl, Br, and I) are usually introduced in edge sites, involving the conversion of sp^2^ carbon bonds into sp^3^ ones, which causes important structural and electronic modifications in the aromatic basal planes of the functionalized carbon material. As an example, the electronic conductivity can be modulated from metallic to insulating upon the F/C ratio.^95^ As they have higher electronegativity than carbon atoms, halogen functionalization can polarize adjacent carbon atoms, facilitating catalytic reactions.^96^ The catalytic activity of these new sites seems to be related to the electronegativity of the halogen and the ionic–covalent character of the C–X bonds (partially ionized bonds favor a charge transfer). Studies on the incorporation of other heteroatoms are scarce, with some examples for selenium and silicon, mainly for exploring their electrocatalytic performance.^68^

#### 2.2.2 Metal-Atom Induced Defects

Carbon materials are well-known support platforms of highly dispersed metallic active particles, with many studies reporting the key role of porosity (surface area, pore volumes, average pore size) and surface functionalization (basicity, hydrophobicity, density and nature of surface moieties) to achieve a high metal dispersion and catalytic activity.^14^ Recently, the interest has settled on two main axes: carbons with dual metal sites, and the stabilization of single-atom and single-cluster catalysts (SAC, SCC) to achieve the atomic utilization efficiency of the catalytic activity. While most of the studies on the topic agree that creating defect sites in the carbon materials (defect engineering) is essential to stabilize the high-density atomically dispersed metal sites, correlating the nature of those surface defects with the catalytic activity and stability (for allowing a rational design of anchoring sites) is still a challenging task. Several authors have reported that heteroatom doping and the presence of the intrinsic defects in metal-N4@N-doped carbons are good strategies for achieving a high catalytic activity of SAC. The formation of strong interfacial interactions between the single metal atoms and the defects controls the coordination environment of the metal active sites, favoring a high metal dispersion.^97–101^ A high density of prismatic surfaces (edges) on the carbon materials also seems to stabilize the single metal atoms, whereas metal nanoclusters are more likely to form on basal surfaces of the distorted graphene layers.^51^

### 2.3 Functionalization vs Doping

As indicated in Section 2.1, various heteroatoms can be introduced to the carbon matrix using a broad range of surface modification methods, discussed in detail in Section 3. However, one should be aware that not all modification methods represent the process referred to as *doping*. The latter involves the introduction of heteroatoms to the conjugated carbon rings of the graphene layers/within the carbon framework. Depending on the electronegativity of the heteroatoms with respect to that of the carbon atom, the electronic structure of the distorted graphene sheets of the carbon pores is modified by inducing a positive charge either to neighboring carbon atoms or to the heteroatom itself^102^ (Figure 3A), as in the case of high-electronegativity-nitrogen doping, or as a result of a smaller electronegativity of dopants compared to that of the carbon atom, as in the case of boron. That uneven charge distribution is important for many catalytic and electrocatalytic applications. *Functionalization*, on the other hand, is an introduction of the heteroatom-containing functional groups analogous to those in organic chemistry, to the edges of the carbon layers.

**Figure 3:**
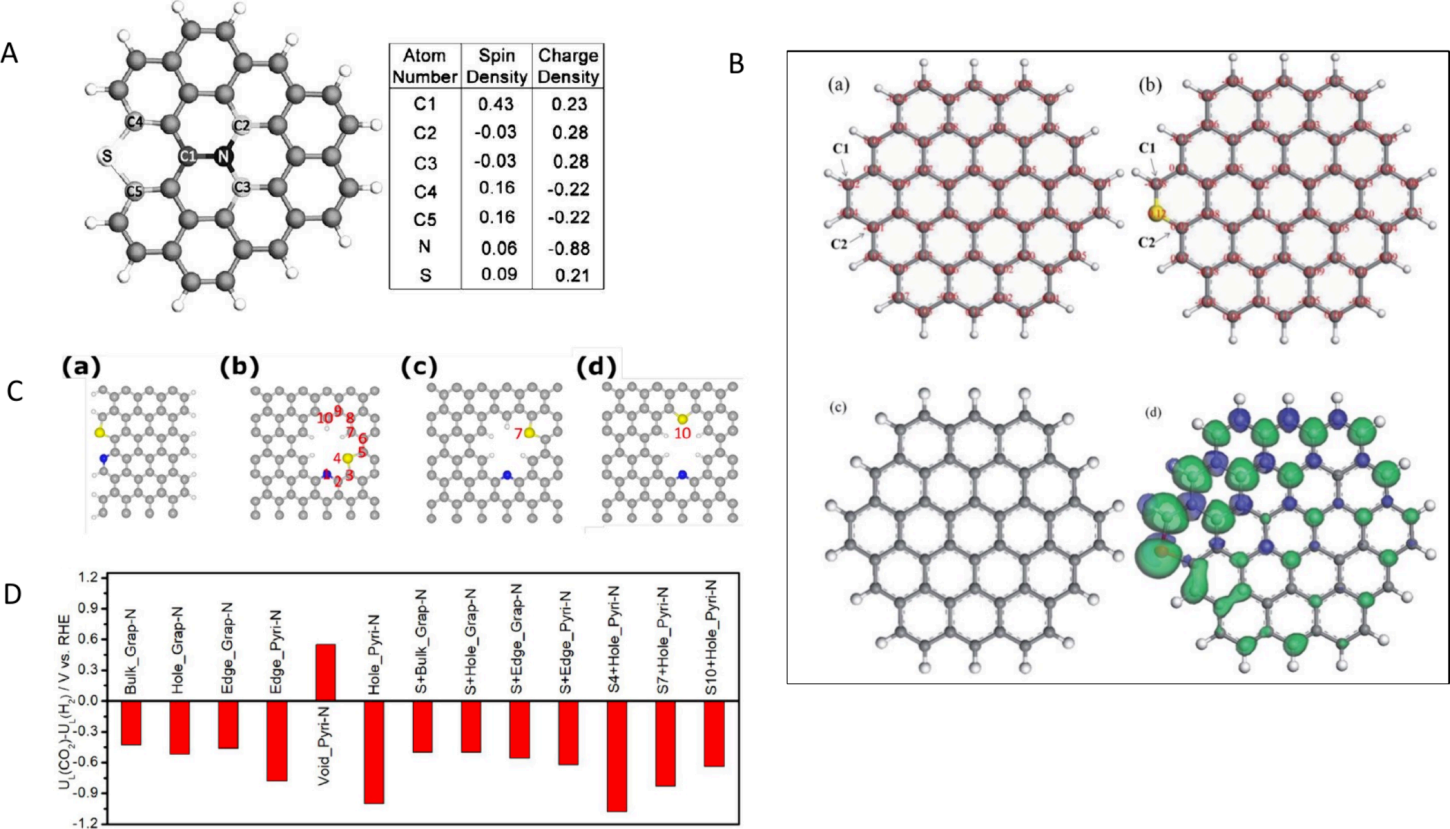
A) Spin and charge density of graphene network (gray) dual doped by N (black) and S (white). C1 has very high spin density, C2 and C3 have high positive charge density, and C4 and C5 have moderately high positive spin densities. Reproduced with permission from ref.^102^ Copyright 2012, Wiley and Sons; B) On-site Bader charge (a) and spin density isosurface (c) for undoped model carbon layer; on-site Bader charge (b) and spin density isosurface (d) for S-doped model carbon layer. Reproduced with permission from ref.^105^ Copyright 2020, the Royal Society of Chemistry; C) Proposed models of S+Pyri-N: (a) S+Edge_Pyri-N; (b) S4+Hole_Pyri-N; (c) S7+Hole_Pyri-N, and (d) S10+Hole_Pyri-N. The black, blue, yellow, and white spheres represent carbon, nitrogen, sulfur, and hydrogen atoms, respectively. Reproduced with permission from ref.^106^ Copyright 2019, Elsevier; D) Difference in limiting potentials for CO_2_ reduction and H_2_ evolution on various proposed models. Reproduced with permission from ref.^106^ Copyright 2019, Elsevier.

The introduction of nitrogen within the carbon framework brings the advantage of a minimal structural distortion. It is due to the similar sizes of N and C atoms resulting in similar bond’s length (C–N is 1.41 Å and C–C 1.42 Å). On the other hand, nitrogen is much more electronegative than carbon is (3.04 vs 2.55 for carbon) and thus brings a large positive charge to the neighboring carbons.^102^ Sulfur, or selenium, on the other hand, differs only slightly in electronegativity from carbon (2.58 for S and Se), but their atomic size is much larger, which results in the distortion of the carbon framework and in the enlargement of the graphitic spacing. This, in turn, leads to numerous defects and strain within the carbon matrix, which breaks the symmetry of the charge and spin density distribution, affecting the specific localization of electrons (Figure 3B). Another mechanism introducing defects as catalytic sites is the mismatch of the outermost orbitals between S and C, resulting in a nonuniform spin density (Figure 3A).

Boron is an electron deficient element. Its common configuration in the carbon matrix is in so-called “in-plane” doping. In this form, it maintains the sp^2^ hybridization of the carbon framework as BC3.^103^ Even though the length of the C–B bond is longer than that of the C–C bond (∼1.50 Å vs 1.42 Å; electronegativity of B is 2.04), the strong polarization between neighboring C atoms and electron-deficient B was indicated as minimizing the mechanical stress on the host carbon structure.^104^ Even though doping is very beneficial for altering the electronic structure of the carbon materials, the challenge remains in avoiding oxidation of boron into boron oxide.

Phosphorus doping, as that of boron, remains a challenge. P has a similar binding shell electron configuration to that of N but a lower electronegativity than that of carbon (2.19). Its large atomic size leads to an increase in the bond length (C–P; 1.76 Å), distorting the carbon framework^107^ and introducing more edges and topological defects.^108^ As boron, phosphorus can accept oxygen, leading rather to functionalization of carbons than its doping. Nevertheless, such configurations also distort the electronic structure of the carbon matrix due to an electron accumulation around the O and C atoms. It is due to the differences in their electronegativity (P-2.19; O-3.44 and C-2.55).

Co-doping with two or three heteroatoms can further distort the graphene matrix, leading to more defects and thus to more active catalytic sites. One example is N and S codoping where not only the nature of heteroatoms but also their location and distance from each other were found important (Figure 3C), as shown by the DFT calculations and controlled experiments of Pan et al.^106^ Their results showed a double role of incorporated sulfur in promoting CO_2_ reduction. It facilitated the generation of abundant pyridinic N species important for CO_2_RR sites and enhanced the intrinsic catalytic reactivity of both pyridinic and graphitic N by decreasing the energy barrier for the formation of *COOH intermediate (Figure 3D).

Another important doping of the carbon surface is that through the introduction of single metal atoms to the carbon matrix through coordination with nitrogen^109^ or sulfur.^110^ Nevertheless, this doping strategy, although very important, and of recent research interest, is beyond the scope of this review. We believe that those interested in this kind of doping can find useful information in heteroatom doping strategies presented in this paper, and for further inspirations the readers are referred to recent reviews on the single atom carbon catalysts^111^ or on double atoms.^112^

The examples of oxygen-containing chemical functional groups introduced to the edges of distorted graphene layers are carboxyls, carbonyls, phenols, ethers, and quinones. The introduction of nitrogen targets amines, imides, amines, pyridines, quaternary nitrogen or nitro groups. Sulfur can exist as mercaptans, sulfoxides, sulfone or sulfonic acid and phosphorus or boron in oxides or acids. While some of them have electron donor ability (−OH, −OR), others withdraw electrons from the carbon matrix (−NO_2_, −CN, −COOH, −SO_3_H). These groups affect the polarity of carbons and also their hydrophilic/hydrophobic character, which in turn alter their reactivity and interactions in the aquatic environments as discussed in detail in various sections of this review.

### 2.4 Brief Overview of Characterization Methods

There are several methods available for obtaining information about the surface chemistry of porous carbons, each of them offering unique advantages and limitations (cf. Table S1 in the Supporting Information). Most of these techniques require advanced equipment and careful handling of samples to ensure representativeness and avoid interference/contamination. A high level of expertise is also required to correctly interpret the data, which sometimes involves advanced modeling/fitting and/or theoretical calculations. These factors pose a significant challenge to achieving reproducibility across laboratories. Round-robin interlaboratory analyses using reference carbon materials should be conducted to increase confidence in the results and allow data comparison. This practice is unfortunately not common in material’s science characterization (not just in the study of the surface chemistry of porous carbons).

A combination of several techniques is often employed to comprehensively understand the surface chemistry of porous carbons. Among the available methods, XPS (X-ray photoelectron spectroscopy) is currently the most widely used to characterize the surface chemical environment. Its popularity stems from its unique ability to obtain both qualitative and quantitative information on all heteroatoms (e.g., O, N, S, P, and B) that can be introduced into carbon structures, providing not only an elemental analysis but also insights into the chemical states of these atoms, allowing for the identification of different functional groups and chemical environments. The main constraint of using XPS for porous carbons, which typically have an extended porosity, is ensuring that the external layers analyzed (only a few nanometers deep) are representative of the bulk material. This aspect becomes even more challenging without strict sample handling, as XPS is highly sensitive to surface contaminants, which can significantly change the actual surface chemistry. Comparing the percentage of each heteroatom obtained by XPS with that obtained by a bulk elemental analysis is the best way to confirm the representativeness of the surface chemistry. Another critical point, not always found in published results, is following the best available methodologies for peak spectra deconvolution, which is usually required to quantify different surface groups. A C 1s region, in particular, can be very complex, impeding a straightforward data evaluation. Namely, (i) due to the predominantly graphitic nature (sp^2^ bonding) of the carbon structure, the corresponding peak in the C 1s deconvolution should be asymmetric; (ii) for samples containing only carbon and oxygen, the ratio of the total amount of oxygen obtained from C 1s and O 1s deconvolutions should be close to 1, and a similar methodology can be applied to other heteroatoms whose contributions appear in different XPS peaks. Several publications are available in the literature addressing this topic.^11,113–115^

FTIR (Fourier transform infrared spectroscopy) is often used to analyze the surface groups of carbon materials due to its simplicity and easy accessibility. Nevertheless, the information obtained is usually limited and mainly of a qualitative nature. Carbons are black materials and absorb most of the IR irradiation, leading to low signal-to-noise ratios even for highly functionalized samples, making it difficult to distinguish between the different absorption bands, which usually appear overlapping. Despite this, several authors developed quantitative approaches, usually in combination with other methods. Kohl et al.^116^ deconvoluted the DRIFT (diffuse reflectance FT-IR) spectra region between 1500 and 1950 cm^–1^ with a set of five single Gauss functions and identified four surface oxygen groups by the combination of the results of TPD and DRIFTS, which were assigned to carbonyl (1602 cm^–1^), lactones (1740 cm^–1^), carboxylic acids (1765 cm^–1^), and anhydrides (1792 and 1855 cm^–1^). The specific concentration of each group was calculated from the integral and the conversion factor obtained from solving a mass balance. A similar semiquantitative approach had been used previously by Koch et al.^117^

TPD (temperature-programmed desorption) is a relevant method for the qualitative and quantitative characterization of oxygen-containing surface groups. The various groups decompose upon heating in an inert atmosphere, releasing CO, CO_2_, or/and H_2_O at different temperatures; the peak temperature and area provide information about the type and amount of each group, respectively. Compared to XPS, TPD has the advantage of giving information on bulk oxygen, which can be critical for porous carbons, and the deconvolution of the CO and CO_2_ profiles can be more informative in distinguishing among the different surface groups. Special care should be taken in the experimental operation to avoid secondary reactions and mass transfer effects and in the deconvolution methodology to prevent misinterpretations. Several references have addressed these points.^33,118–121^ TPD analysis of surface groups involving other heteroatoms besides oxygen is less mature, although some results have been obtained for N and S-containing groups.^122–125^ Recently, Nishihara and co-workers have introduced a high resolution TPD technique where heating of N-doped carbons up to 1800 °C enabled qualitative and quantitative analysis of a nitrogen environment at the ppm level.^126^

Information about their decomposition products and respective desorption temperatures was compiled by Herold et al.^33^ More recently, Ishii et al.^34,120,127,128^ applied a TPD methodology with an ultrahigh quantitative sensitivity using temperatures up to 1800 °C to quantify H-terminated edge sites, associated with surface defects, which may play a critical role in electrochemical applications. In the same group, Nishihara and colleagues have recently provided insights into the relationship between the number of edge sites and the number of unpaired electron spins by combining the high-sensitivity TPD technique with magnetic susceptibility measurements using SQUID (superconducting quantum interference device).^129^ They investigated how these factors influence the catalytic activity of dehydrogenative oxidation reactions and concluded that these reactions are catalytically promoted by unpaired electrons at edge sites. The recent study by Ishii et al.^127^ using deuterium-labeled temperature-programmed desorption to understand the chemical structure of carbon edge sites reported the relationships between the desorbed species determined by TPD and the chemical species formed at the carbon edge sites.

Intermittent TPD (ITPD) is a powerful variation of TPD in which a sawtooth heating program is applied to generate a number of interrupted desorption measurements. These desorption runs can be analyzed in their initial parts through applications of Arrhenius plots, allowing to obtain information about the desorbing species. One example of the application of this methodology to carbon materials is the work of Gaillard et al.,^130^ in which different CO_2_ and CO-type groups were distinguished from the various values of a desorption activation energy. Temperature-programmed analyses of carbon materials under reactive atmospheres can also be useful approaches to characterize the surface chemistry, providing quantitative and qualitative information on the reactivity of different species upon the choice of the atmosphere. While temperature-programmed reduction and oxidation (TPR, TPO) under a reductive (e.g., hydrogen) and oxidant atmosphere (e.g., oxygen and carbon dioxide), respectively, are extensively used for the characterization of zeolites and metal oxides (among others), their application to carbon materials is rather scarce.^33,123,131–133^

Overall, a good agreement has been reported between the characterization obtained from TPD and TPR/TPO experiments on functionalized carbon; the latter complementing the information about the reactivity of the surface groups under reducing and oxidizing atmospheres. Some recent studies that combine both TPD and TPR to get information about carbon active sites and the fate of oxygen functional groups provide quite interesting information about the physicochemical properties of oxygen functional groups, as well as on the formation of carbon active sites.^134,135^ Unfortunately, the use of other probes (e.g., organic vapors such as amines, pyridine or alcohols, CO_2_, NH_3,_ CO, NO, H_2_S, ...) and selective adsorption/desorption—chemisorption—for the characterization of the surface chemistry of carbon materials is scarce, despite being a common practice in the characterization of zeolites and metal-based catalysts.^136^ The few examples reported for carbon materials mainly refer to TPD of NH_3_ and of CO_2_ to determine acid/basic character.^137–139^ In these cases, the carbon material is exposed to the selected probe at ambient conditions to allow chemisorption at the acidic/basic surface sites, followed by a TPD experiment to quantify the amount desorbed and the strength of the chemisorbed sites. Based on the temperature profiles and the nature of the profile, it is possible to differentiate between Brönsted- and Lewis-type acidity. However, a careful interpretation of the desorption profiles must be carried out in the case of porous carbons due to (i) the contribution of physisorption of the probe in the pore network; (ii) reactivity of the probe with the carbon matrix to modify the porosity or create other functional groups; and (iii) the desorption of the functional groups existing in the carbon material.

The nature of acid/basic centers and the relative abundance of defective sites (edge sites, in-plane vacancies) can also be characterized by gas chemisorption. Indeed, the chemisorption of oxygen is a classic method to determine a so-called active surface area (ASA) as a quantitative indicator of the relative abundance of defects in carbon materials, owing to their different reactivity compared to that of basal planes.^22^ While it was a popular method more than 30 years ago,^24^ it has been recently barely used despite being a parameter of paramount importance for a better understanding of the chemical nature and the reactivity of edge sites in carbon materials.^25,140,141^ Some recent examples of the application of the ASA measurement that support the relevance of this technique can be found and these examples will be presented and discussed in the next sections of this review.

Probing prismatic and basal plane surfaces of carbon materials has also been recently reported by Serp and co-workers. By combining high temperature TPD, XPS and gas adsorption^142^ a good agreement was obtained from those three techniques for the analysis of carbon nanotubes and nanofibers. However divergences appeared for materials exposed to a high temperature thermal annealing. The authors attributed this effect to the presence of vacancies on closed carbon loop after the heat treatment, leading to the accumulation of very reactive defects at the loop level. A correlation has been reported between the spin density of the annealed carbons and their prismatic and basal surfaces, underlining the need to combine several techniques to characterize the prismatic/basal surfaces of carbon materials. The authors also found that surface defects play an important role in the stabilization of single metal atoms on carbon surfaces. In this context, carbon materials presenting predominantly prismatic surfaces facilitated the synthesis of metal single atoms (unsaturated edges of prismatic surfaces would serve as anchoring sites for the metal), as opposed to carbons with a high abundance of basal surfaces that would favor the formation of nanoclusters.^51^

Drawing inspiration from other disciplines and incorporating reactivity and stability concepts when characterizing carbon materials should improve our understanding of their surface chemistry and perhaps lead to a rationalization of performance indicators in different fields. Yet, selecting the probe for chemisorption measurements can be challenging, since the nature of the active sites in carbon materials (and the stoichiometric relationship with the selected probe) is not easy to determine.

Inspired by the concept of the active surface area based on oxygen chemisorption, and driven by the growing interest in carbon materials in various electrochemical applications, the so-called electrochemical active surface area (ECSA) has emerged as a useful indicator to describe the electrochemical reactivity of the carbon surfaces. By focusing on the interactions at the interface carbon material/electrolyte, useful information can be extracted about both the porosity (through the formation of an electrical double layer), the redox nature of electroactive carbon surface groups, and their interdependence in the overall electrochemical response of a carbon material. The determination of ECSA in non-porous metal-based materials is based on several reliable methods (mainly involving hydrogen/metal underpotential deposition, CO stripping, Randles–Sevcik and Cottrell equations). However, these methods are not universally applicable to all types of materials since not all of them are sensitive to those probes.^143–145^ In porous materials, ECSA is mostly determined through the capacitance of the double layer (*C*_dl_) divided by the specific capacitance per a geometric surface area of a flat electrode reference material (*C*_s_), but the method has several sources of error. Firstly, *C*_dl_ must be calculated in a non-Faradaic potential region to ensure that the electrochemical response is not affected by the contribution of electroactive species (note that this may be difficult in functionalized materials). Furthermore, most studies use a *C*_s_ reference value of 40 μF cm^–2^ regardless of the material and the electrolyte. This value was reported by McCrory et al. for metallic electrodes in an alkaline electrolyte,^146^ making it not appropriate for porous carbon materials nor for different electrolytes (since the electrochemical response is governed by the nature of the interactions between ions in an electrolyte and a solid surface). As an example, a recent study on the determination of the ECSA of a carbon felt has reported differences of almost 1 order of magnitude depending on the electrolyte (ca. 34 cm^2^ in 1 M H_2_SO_4_; 27 cm^2^ in 1 M Na_2_SO_4_; 95 cm^2^ in 1 M KOH).^147^ A more precise approach to determining ECSA in carbon materials would be to calculate experimentally *C*_s_ as the capacitance of a flat glassy carbon, or the non-porous carbon used as the substrate (e.g, carbon paper, felt) in the electrolyte under study. It should also be noted that the capacitance in porous carbons measures the surface available to the electrolyte, and not all this surface corresponds to the electrochemically active surface for a specific reaction.

Electrochemical techniques (linear-sweep/cyclic voltammetry, polarography, redox probes) can also be used to characterize electroactive surface groups on a carbon electrode, since many of those surface groups undergo electron transfer reactions (e.g., quinone/hydroquinone redox pair, lactones, pyrones).^148–153^ In general, it is possible to quantify electroactive groups by electrochemical techniques, but this is often limited by difficulties related to a poor electrical conductivity of some carbon materials (leading to a low surface sensitivity), and the difficulty in assigning the redox peaks to specific types of groups due to overlapping reactions.

Chemical titrations, often called Boehm’s titrations due to the pioneering work of Boehm,^154^ are typically limited to quantifying acidic oxygen-containing surface groups, and they fail to account for a large portion of the total oxygen content of a material.^70,155^ More detailed titration method leading to the information on the specific p*K*_a_ of groups existing on the carbon surface, along with their amount, was developed by Jagiello and co-workers.^156^ To derive that data, they used a rigorous mathematical procedure to deconvolute a proton binding curve. Unfortunately, the method cannot distinguish the p*K*_a_ of groups related to various heteroatoms and provides data only in the p*K*_a_ range between 3 and 11. Nevertheless, it is very useful to understand the effects of surface chemistry in an aqueous environment, where protonation/deprotonation can play a role. Titration methodologies can also be used to determine the pH at the point of zero charge (pH_PZC_). Above and below this value, the carbon surface becomes negatively or positively charged, respectively.^157,158^ This determination is essential for the adsorption of ionic compounds^159^ and for metal impregnation during catalyst synthesis.^160^

The best way to obtain an elemental analysis of the bulk sample of carbon materials is by using a CHNS-O technique, which is limited to the elements included in the name of the method. For metals, boron and phosphorus, inductively coupled plasma optical emission spectroscopy (ICP-OES) or inductively coupled plasma mass spectrometry (ICP-MS) are good options. EDS/EDX information should be considered with care due to its semiquantitative nature.

To obtain relevant information about the effect of the surface chemistry of porous carbons in different energy-environmental applications, correlations between their performance and the surface groups acting as active sites are mandatory, such as obtaining the turnover frequency in catalytic applications. For that, the critical point is to identify and primarily quantify the relevant surface groups. Among the different methods described, XPS can be applied for all the heteroatoms, with careful confirmation that the surface represents the bulk material, and TPD for oxygen-containing surface groups are the most relevant characterization methods.

Additional challenges arise when a functionalization with multiple heteroatoms is used. This approach is becoming more common and increases the complexity of the analysis. In XPS, the most critical point is the C 1*s* peak deconvolution since carbon can be bonded with all the heteroatoms (C–X, C=X, or X–C=X, where X can be any heteroatom). A careful mass balance should be considered by comparing the amount of each heteroatom obtained from its contribution to the C 1*s* and from the respective heteroatom peak. The deconvolution of the O 1s peak may suffer from the same problem, as it can also be bonded to different heteroatoms. In TPD, it is known that carbon materials containing phosphorus groups can reveal an additional CO peak between 700 and 950 °C, which results from the successive transformation of the C–O–P into C–P–O, C3–P=O, and eventually C3–P groups during the heat-treatment process.^161,162^

Solid-state NMR can provide additional information about specific functional groups on carbon surfaces through their distinct chemical shift signatures. For example, ^13^C NMR characterizes various carbon-based functionalities, ^31^P NMR detects P-containing groups,^163^ ^19^F NMR is used for fluoroalkylated porous carbons,^164^ ^15^N NMR identifies N-doped carbons,^165,166^ and ^11^B-NMR reveals boron-containing surface groups.^167^

Raman spectroscopy is very sensitive to the structural disorder of carbon, but cannot directly distinguish heteroatom-containing surface groups. Therefore, it is mainly useful for comparing the degree of graphitization or disorder among samples (e.g., via the D/G intensity ratio), which correlates with the abundance of edge sites and surface defects. Several reviews are available related to Raman spectroscopy in carbon materials.^168–170^ Raman spectroscopy is very useful for *in situ* measurements and there are examples of its application that are mentioned in this review. However, attention must be paid to avoid laser-induced degradation of the materials during the measurements under polarization conditions.^171^

More advanced analytical tools are needed to obtain detailed information about single atoms, which is increasingly important for various catalytic applications, especially those involving transition metals (e.g., Fe, Ni, Co, Al, Zn) for the ORR and OER. Several reviews are available on the characterization of single atom catalysts.^111,172–176^

Confirmation of single metal atoms in carbon matrices typically relies on a combination of high-resolution imaging, spectroscopy and specialized techniques. HAADF-STEM (aberration-corrected high-angle annular dark-field scanning transmission electron microscopy) and STM (scanning tunneling microscopy) provide real-space atomic resolution images of single atoms (direct evidence), while XAS (X-ray absorption spectroscopy) and Mössbauer (specific for Fe) provide an element-specific confirmation of atomic dispersion and chemical state (indirect but powerful evidence). Microscope-based EDS and EELS (electron energy loss spectroscopy) combine the imaging and spectroscopic approaches by identifying single atoms *in situ* in the microscope. Typically, several techniques are used in tandem; for example, HAADF-STEM to see single atoms, XAS to confirm their oxidation state and isolation, and in the case of Fe, if available, Mössbauer to double-check the absence of clustered phases. XAS, and more specifically XAFS (X-ray absorption fine structure), includes both X-ray absorption near edge structure (XANES) and an extended X-ray absorption fine structure (EXAFS). XANES provides insights into the electronic structure and oxidation states, while EXAFS offers detailed information on chemical bonding, an interatomic distance and the coordination number of the target element.^172^

HAADF-STEM is highly sensitive to single heavy atoms dispersed on carbon supports, with isolated metal atoms readily distinguishable as bright dots.^172^ XAS is one of the most critical tools for metal–nitrogen-carbon (M–N–C) single atom catalysts. These materials often have the metal coordinated by N/C in a carbon matrix. For example, for Fe, Fe K-edge XAS will show a Fe–N/C first shell at ∼1.4–1.6 Å (phase-uncorrected distance) and no Fe–Fe contributions if Fe is atomically dispersed. Mössbauer spectroscopy is a very specific way to study single Fe atoms, as in Fe–N–C catalysts.^177,178^ Combining EXAFS and Mössbauer results, Li et al. observed that no signals for iron carbides, iron oxides or metallic iron were detectable, demonstrating the high purity of the prepared single Fe atom catalysts, which contained only isolated Fe moieties with distinct N coordination environments.^178^ Time-of-flight secondary ion mass spectrometry (TOF-SIMS) could be another complementary technique to other high-resolution methods. As an example, it was used to confirm the presence of Al–N4–N motifs with axial N ligands in nitrogen-doped porous carbon^179^ and Ni–N bonding environments within Ni,N-doped carbons.^180^

Electron energy-loss spectroscopy (EELS) provides qualitative and quantitative information about the electronic structure of carbon materials, which can be related to the fraction of sp^3^ and sp^2^ coordination of carbon atoms. Since the ratio of sp^2^/sp^3^ density of the carbon atoms varies with the structure and the surface functionalization, this technique allows to follow the evolution of the local environment of the carbon atoms induced by the incorporation of heteroatoms. It also provides information about the evolution of electronic transitions involving π- or σ-electrons.

Recent advances in the field of electron tomography allow the 3D imaging of materials with an unprecedented spatial resolution in a micron to subnanometer range. Combined with energy dispersive spectroscopy (EDS) and electron energy loss spectroscopy, these techniques are capable of further expanding the structural resolution with chemical, crystallographic, and topological data of the materials.^181,182^ Owing to the complexity of these techniques, and the relatively long time of analysis and data interpretation, typically small regions of the materials are characterized, which may complicate the assessment of compositional heterogeneities in the materials. More recently, atom probe tomography has reached a subnanometer spatial resolution coupled with a compositional sensitivity, offering unique possibilities in the characterization of metallic catalysts (particularly of interest to rationalize the design of stable single atom catalysts).^183,184^

Electron spin resonance (ESR) or electron paramagnetic resonance (EPR) can be used to characterize spin properties in carbon surfaces, accounting for a delocalized conduction of π-electrons, free radicals, and defective carbon structures such as dangling bonds associated with terminating oxygen/nitrogen groups. Typically, removing surface groups in carbon materials via annealing or reducing protocols results in structural modifications that increase the density of unpaired electrons. Thus, EPR line-shape and temperature-dependence allow proving the relationship between structural/chemical transformations and catalytic/electrochemical signatures of the carbons through investigating the nature of the spins.^43,184–187^ For carbon materials, the EPR signal is assigned to two main contributions: a broad component associated with delocalized π-electrons in aromatic domains, and a narrow component related to localized sigma “dangling bond” spins of defective structures. Such defect-induced paramagnetism in carbon materials has been mainly related to point defects, such as vacancies in basal planes, the zigzag edges of prismatic surfaces, chemical doping of heteroatoms in the carbon lattice, or incompletely closed graphitic shells.^51,142^ A recent study on the characterization of basal and prismatic surfaces of various carbon materials using ASA (N_2_ adsorption), HT-TPD, XPS, EPR, and magnetic susceptibility measurements reported a reasonable agreement for the quantification of prismatic surfaces in pristine carbons.^142^ However the authors identified some discrepancies after thermal annealing, which they attributed to the formation of curved surfaces that cannot be distinguished by XPS but which can be detected by high temperature TPD, ASA, and magnetic measurements. Indeed, the elimination of surface groups (oxygen, aliphatic groups) upon thermal annealing creates a large density of vacancies and edges that rearrange at high temperature through the formation of curved structures (e.g, migration of vacancies toward the edges; growth of vacancies in the basal planes). Quantifying the unpaired electron density of spins (determined by magnetic susceptibility or EPR measurements) has also proven to be a useful approach for correlating the performance (electrocatalysis, energy storage and conversion) of carbon materials with their structure. Some correlations have been reported between the electrochemical storage capacity of activated carbons and hard carbons in supercapacitors and sodium ion batteries and the spin density and the electrochemical storage capacity of activated carbons, respectively.^43,185,186^

EPR is also a powerful tool to investigate paramagnetic intermediates formed through radical mechanisms, which are widespread in photo­(electro)­catalytic processes. By conducting *in situ* measurements or coupling with spin-trapping chemicals to form stable paramagnetic adducts (to increase the detection timescale), these radical species can be identified through their characteristic g-tensors and hyperfine couplings, and relaxation times.^188–190^

Quantitative evaluation of topological defects remains challenging. Although the topographic features are a direct consequence of structural defects in the graphene sheets or of introduced heteroatoms, there are no straightforward methods for their quantification. Some information, albeit rather local, can be obtained from the analyses of TEM, STEM, and AFM images. Raman spectroscopy can provide some semiquantitative structural information, but specific bands are assigned to a broad range of defects. Some attempts have been made in the literature^191^ to predict the plausible 3D pore structure of microporous carbons, using carbon models containing structural defects as vacancies, sp cross-links, and edges. These studies provide a semi-quantification of the contribution of those defects to the 3D structure of the porous carbons, with a good correlation with local high resolution TEM images of those carbons. However, they are constrained by the finite number of simulated kernels used for fitting and do not include the actual chemical composition of carbon.

Other works^35,75,192–196^ have proposed the quantification of structural defects of carbons (zigzag, armchair edges, 5,7-membered rings, pyrrolic and pyridonic N-sites) by the analysis of XPS data and FTIR. However the assignments of the peaks (particularly for XPS) are still under debate due to the difficulty for validating the deconvolution analyses by other techniques. The synthesis of those carbons with controlled structural defects is further discussed in Section 3.5.

## 3 Methods of Modification and Doping

The performance of porous carbons for various applications, including adsorption, catalysis or sensing, can be significantly enhanced by introducing heteroatoms in the form of surface groups or dopants, including those based on oxygen, nitrogen, sulfur, phosphorus, and boron. These heteroatoms alter the surface chemistry, improving properties such as hydrophilicity, catalytic activity, and electrical conductivity. The most common methods for modifying and doping carbon materials can be categorized into wet chemical treatments, dry processes, precursor-based strategies, surface defunctionalization, and bottom-up synthesis.

### 3.1 Wet Methods

Wet chemical methods involve liquid-phase reactions to introduce functional groups onto the carbon surface. These methods are generally straightforward and scalable, allowing for a uniform modification of the surface. Below, we discuss the introduction of different heteroatoms using wet methods.

#### 3.1.1 Oxygen Functionalization

Oxygen-containing functional groups, such as carboxyl, anhydrides, lactones, phenols and carbonyls, can be easily introduced onto porous carbons through oxidation treatments.^155^ The most common oxidizing agents include nitric acid (HNO_3_), hydrogen peroxide (H_2_O_2_), and ammonium persulfate ((NH_4_)_2_S_2_O_8_). Other treatments with sulfuric acid (H_2_SO_4_) and phosphoric acid also create oxygen surface groups, but they are mainly used to introduce sulfur and phosphorus-containing groups, respectively, as discussed below.

Nitric acid is widely used to introduce oxygen groups into the carbon surfaces. The process typically involves treating the carbon material from a room temperature to a boiling temperature (in this case under reflux conditions) with a 1 to 6 M solution of HNO_3_ for 1 to 24 h. This treatment introduces almost all types of oxygen-containing surface groups but is specially used to increase the more acidic ones (carboxylic, lactones, and phenols), increasing the surface acidity and hydrophilicity. The concentration of the surface groups can be adjusted by varying the acid concentration, the temperature or the contact time during the treatment process.^119,158^ The degree of functionalization also depends on the properties of the carbon material, namely on the number of unsaturated carbon atoms at the edges or the defects on the basal planes of the graphene domains. Under the most standard conditions used, liquid phase oxidations have the advantage of not significantly changing the textural parameters for porous carbons, although using very drastic conditions like concentrated acid for long times may cause the collapsing of the porous structure.^158,197,198^ Although in lower quantities, the nitric acid treatment can also introduce some N-containing surface groups, which are sometimes neglected. Pyridine and pyridine N-oxide structures are preferentially formed.^199^

Hydrogen peroxide is a milder oxidizing agent compared to HNO_3_ and it has the advantage of being considered a green oxidant. The treatment is usually carried out at room temperature with a 10–30% solution of H_2_O_2_ for 1–6 h. This process introduces all types of oxygen groups, but the more acidic oxygen groups (carboxylic and anhydrides) are formed in a lesser extent compared to HNO_3_, which is relevant for applications that need less acidic (hydroxyl are preferentially created) or neutral surface groups.^200^ Examples are catalysts for nitroarene reduction.^201^ Another advantage of using H_2_O_2_ is the preservation of carbon textural properties.^198^

Ammonium persulfate is a strong oxidizing agent that allows obtaining high oxidation degrees in carbon materials.^72,202^ The treatment with this agent is typically carried out at room temperature, which also allows preserving the porosity of the oxidized materials.^203,204^ The oxidation with ammonium persulfate usually generates a heterogeneous distribution of O-groups (carboxylic, lactones, hydroxyl) on the external surface of the carbon particles, as opposed to oxidation with nitric acid that mainly favors the fixation of the O-groups in the internal surface. In addition to oxygen functionalities, oxidation using persulfate may lead to the incorporation of small amounts of oxidized sulfur groups in the carbon.

#### 3.1.2 Nitrogen Functionalization

Nitrogen functionalities on porous carbon are mainly introduced by carbonizing nitrogen-containing organic compounds or by treatment at high temperatures of carbon materials with nitrogen-containing gases,^205,206^ as discussed below. Introducing nitrogen-containing surface groups to porous carbons using only wet methods is not so common, but it typically involves the chemical treatment of a carbon material with nitrogen-rich compounds. As an example, Raymundo-Piñero et al.^207^ used nitrogenated compounds like urea, NH_3_, dicyanodiamine (DCD), and N,N-dimethylformamide (DMF) to introduce N groups to an activated carbon surface. The reactions were carried out in an autoclave under pressure at 300 °C, using DMF as a solvent. Most of the methods involve a wet step to impregnate carbons with solutions or suspensions of nitrogen-rich compounds, like urea and melamine, but they are followed by a drying step and a heating step at high temperatures under an inert gas flow, generally above 600 °C to guarantee the decomposition of the nitrogen precursor used. Bandosz and collaborators prepared a series of N-doped carbon materials following this procedure, using urea^208,209^ and melamine.^210^ In some cases, a pretreatment with nitric acid was performed to introduce large amounts of oxygen-containing surface groups on the starting material, which are expected to act as anchor sites. Figure 4A shows a schematic representation of changes in surface chemistry of carbons due to saturation with urea at room temperature and heat treatment at 450 and 900 °C.^208^ The textural parameters of the obtained materials are usually affected by this type of treatment. The commercial activated carbon Centaur is obtained by urea modification of a low temperature char, followed by heat treatment.^211^

**Figure 4:**
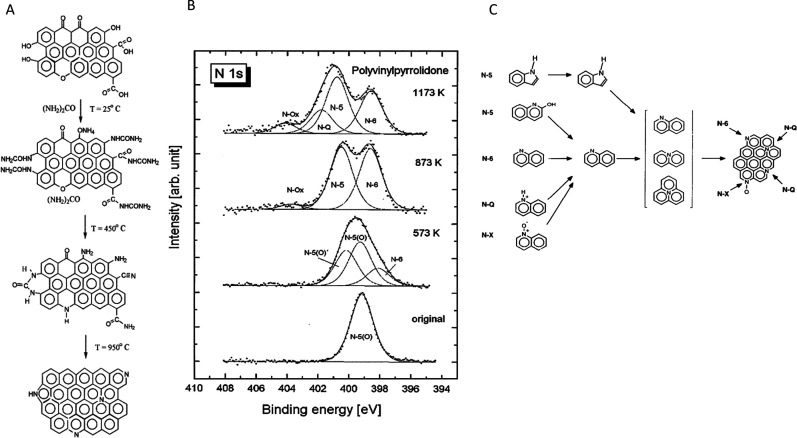
A) Schematic representation of changes in surface chemistry of carbons due to saturation with urea and heat treatment at 450 and 950 °C under Ar atmosphere. Reproduced with permission from ref.^208^ Copyright 2022 American Chemical Society; B) N 1s XPS spectra of polyvinylpyrrolidone and its chars obtained at various temperatures. Reproduced with permission from ref.^212^ Copyright 1999, Elsevier; C) Visualization of the evolution of nitrogen functionalities in carbonaceous materials during pyrolysis proposed by Pels et al. Reproduced with permission from ref.^213^ Copyright 1995, Elsevier.

#### 3.1.3 Sulfur Functionalization

The synthesis of S-doped carbon materials has been recently reviewed.^214^ Like for other heteroatoms, S can be incorporated to the carbon structure by *in situ* methods (carbonization using S-containing compounds) and by a post-treatment (S functionalities are introduced after the carbonization step) using different sulfur sources, which can be divided into three types: (i) elemental sulfur; (ii) inorganics (e.g., H_2_S, SO_2_, Na_2_S, CS_2_, P_4_S_10_, NaH_2_O_3_, K_2_S_2_O_8_; and (iii) organics (e.g., thiourea, l-cysteine, 2-mercaptobenzimidazole, amino acid, rhodamine, rice leaven).^214^ The synthesis process is significantly affected by the precursor used.

Concerning the wet methods, the most common is the treatment of the carbon material with sulfuric acid solutions. The extent of the functionalization depends on the temperature used (from the room to the boiling point temperatures), the solution concentration (1 to 5 M) and contact time (1 to 24 h). This treatment introduces mainly sulfonic acid groups, increasing the Bronsted acidity. Another approach consists of impregnating the carbon materials with a solution or suspension of a S-precursor followed by a heat treatment at high temperatures (from 100 to 1000 °C, 800 °C being the most typical) under an inert atmosphere to decompose the precursor. In most cases, the carbon material is previously treated with nitric acid to introduce oxygen-containing surface groups, which act as anchoring sites.

#### 3.1.4 Phosphorus Functionalization

The synthesis, properties and utilization of phosphorus-containing carbons have been recently reviewed.^215^ Phosphorus groups on carbon materials are sometimes neglected, although phosphoric acid activation is widely used both in industry and in scientific research to produce activated carbons, and thus porous carbons resulting from this preparation method usually contain phosphorus. Similarly to other heteroatoms, there are two main routes to introduce phosphorus on the carbon structure: (i) an *in situ* process, involving the carbonization of a carbon-containing precursor either mixed or chemically bonded with a phosphorus-containing compound; (ii) a postsynthesis method, involving the modification of a prefabricated carbon material with a phosphorus-containing compound, usually at high temperature. The most common phosphorus-containing source used to prepare P-doped carbons is phosphoric acid, but other activating/modifying agents have been used, namely pyrophosphoric acid, polyphosphoric acid, Na_2_HPO_4_, NH_4_H_2_PO_4_, (NH_4_)_2_HPO_4_, (NH_4_)_3_PO_4_, P_2_O_5_, phosphate esters (trimethyl phosphate, tributyl phosphate, triethyl phosphite), guanidine phosphate, triphenylphosphine, phytic acid, or phosphorus-containing ionic liquids.^215^

The wet methods usually include the treatment of a prefabricated carbon with a phosphorus-containing precursor. The most used phosphoric acid is typically used as an 85% solution, for 1 to 12 h, at room temperature to 150 °C. Samples prepared without a subsequent high-temperature step present mainly C–O–P groups and have the advantage of preserving the textural properties of a starting material. This approach was followed to prepare phosphorylated mesoporous carbons.^216^ But, in most cases, the impregnation at low temperature is followed by a heat treatment at high temperature, usually up to 800 °C. This temperature is usually selected because, irrespective of the P-doped carbon preparation method, it is where the P content in carbons reaches a maximum. At lower temperatures, the extent of carbon reaction with phosphorus is low, whereas above 800 °C phosphorus compounds tend to be lost by reduction and/or volatilization.^215^

The phosphorus content depends on the P-containing source, the temperature and concentration of activating/modifying agent (impregnation ratio), gases/atmosphere used during activation/modification, and reactivity, texture and surface chemistry of the prefabricated carbon. The temperature has a paramount relevance on the type of surface groups. Wang et al.^161^ study provides an excellent example of how P-groups transform under a heat treatment. They investigated two series of carbons: one prepared by an H_3_PO_4_ activation of lignocellulose and the other by an H_3_PO_4_ modification of activated carbon, both subjected to the heat treatment up to 900 °C in inert (N_2_) and reducing (H_2_) atmospheres. Significantly more phosphorus was removed in the H_2_ atmosphere. Typically, C–O–P linkages in phosphorus-containing groups progressively transform into C–P–O, C3–P=O, C3–P, and even elemental phosphorus during the heat treatment. In a N_2_ atmosphere, this transformation occurs extensively up to 800 °C, resulting in a significant formation of C3–P=O, while C3–P linkages do not form even at 900 °C. In contrast, in an H_2_ atmosphere, this evolution occurs extensively at temperatures as low as 500 °C, leading to the formation of C3–P linkages and eventually elemental phosphorus.

#### 3.1.5 Boron Functionalization

In a similar way as for the doping of the other mentioned heteroatoms, the general synthesis process of boron-doped carbons is either by an *in situ* method (involving the selection of a suitable carbon precursor, which is mixed with a boron-containing compound) or a postsynthesis method (in which a prefabricated carbon material is mixed with a boron-containing compound), and in both cases a final treatment step at high temperature (typically 800 to 1000 °C) is usually required. The most used boron-containing compound is clearly boric acid (H_3_BO_4_), but other boron sources can be used, such as boron oxide (B_2_O_3_),^217^ sodium borohydride (NaBH_4_),^218,219^ 4-hydroxyphenylboronic acid,^220^ ammonium borate (NH_4_B_5_O_8_·4H_2_O),^221^ in this case aiming to introduce N and B functionalities, boron trifluoride diethyl etherate (BF_3_Et_2_O),^222^ BCl_3_,^223^ borane tetrahydrofuran (BH_3_–THF),^224^ tetraphenylborate salt,^225^ 3-aminophenylboronic acid,^226^ and triisopropyl borate.^227^ In most recent cases, B-doping was applied to graphene materials, with an interesting review published recently,^228^ but the procedures are similar for porous carbons.

### 3.2 Dry Methods

Dry methods of surface modifications involve gas-phase reactions, often at high temperatures, leading to the introduction of heteroatoms to the carbon matrix. These methods have the advantages of being solvent-free and using fewer steps than the wet methods, offering a more precise control over the introduction of functional groups/dopants and are environmentally friendly. Another approach would be using plasma treatments, but these will not be considered here since they are usually not effective for porous carbons. Their effect is limited to a change in an external surface, leaving the internal surface almost unaltered.

#### 3.2.1 Oxygen Functionalization

Oxygen groups can be introduced through thermal treatments in an oxidative atmosphere, such as oxygen (usually in air streams), N_2_O, or ozone. An oxidative thermal treatment in air, eventually diluted in a N_2_ stream, is typically performed at 300–500 °C for 1–10 h. This process creates preferentially less-acidic, neutral and basic oxygen-containing groups on the carbon surface. Carboxylic groups are not present because they are not stable at the temperatures used but anhydrides can be formed. This treatment is preferentially used to introduce phenol and carbonyl groups, and their extent can be adjusted by changing the experimental conditions. Otake and Jenkins^71^ and Figueiredo et al.^119^ prepared several series of carbon materials with different degrees of oxidation, and they were able to obtain materials with different amounts of oxygen surface groups. The main drawbacks are the burnoff of the carbon material, which can be severe for the harshest conditions, and the change in the textural properties.

An ozone treatment generally involves exposing the carbon material to ozone gas at room temperature for 10–60 min. Increasing an ozone dose or contact time enhances the oxidation of the carbon, resulting in a greater number of acidic groups, particularly carboxylic groups, on the carbon surface.^229^ Increasing the treatment temperature up to 100 °C can improve the oxidation efficiency.^230^ The textural properties are also affected, and, usually, porosity decreases with the severity of the treatment. Much milder surface oxidation can be achieved using ozone dissolved in an aqueous solution,^231^ but it becomes a wet method.

#### 3.2.2 Nitrogen Functionalization

Nitrogen groups can be introduced via thermal treatments with nitrogen-containing gases that thermally decompose with generation of highly reactive radicals. Ammonia (NH_3_) is the most commonly used reagent, typically at temperatures ranging from 600 to 1000 °C. At these temperatures, ammonia decomposes, especially upon contact with carbon surfaces, forming radicals such as H^•^, NH_2_^•^, NH^••^, and finally producing H_2_ and N_2_. These free radicals react with the carbon surface, leading to the incorporation of nitrogen species into the carbon surface and some degree of a carbon loss.^205^ Other agents can also be used, such as hydrogen cyanide (HCN) or cyanogen gas (NC–CN).^232^ The quantity of nitrogen groups introduced is smaller than with NH_3_, particularly for (CN)_2_, but the overall results are similar. At the higher temperatures these treatments can form pyridinic, pyrrolic, N-quaternary, and N-oxide groups, enhancing the basicity of the carbon material. Besides the surface chemistry, the textural parameters can be changed depending on the experimental conditions used.^233,234^

Another effective dry method to obtain N-doped carbons is by heat treatment of well-mixed solid mixtures of carbon materials with nitrogen-containing precursors, such as melamine or urea. This process is usually conducted at 500–900 °C (above the decomposition temperature of the nitrogen containing sources) for 2–6 h. A good contact between carbon and the N-containing solid precursor is a critical parameter, and more recently, ball-milling methods have been applied with a great success.^235^ Another approach is to use first a solution/dispersion of the N-precursor to impregnate the carbon material followed by a drying step, as discussed above in the case of the wet methods.

The N-containing groups also change with the temperature used during the synthesis of the carbon material. As a good example, Figure 4B shows the evolution of the nitrogen groups measured by XPS during the pyrolysis of polyvinylpyrrolidone.^212^ It is clear that N-5 nitrogen is converted to N-6 at moderate temperatures (up to 600 °C), and at high temperatures (up to 900 °C), N-6 content decreases while N-Q nitrogen increases. It is interesting to mention that using various N-precursors (polyvinylcarbazole, polyvinylpyridine and polyvinylpyrrolidone), different evolutions of the N-containing groups occurred with the increasing heat treatment temperature, but at 900 °C, irrespective of the starting precursor, only small or no differences were found in the N 1s spectra.^205,212^ These results are aligned with the proposed evolution of the nitrogen functionalities in carbonaceous materials during pyrolysis (schematically presented in Figure 4C) of the seminal work of Pels et al.^213^

Recently, Quílez-Bermejo et al.^236^ used a straightforward postfunctionalization methodology to prepare N-doped carbon with different amounts of N-functional groups, keeping the textural parameters almost unchanged. For that, a commercial activated carbon (MSC30) was mixed with urea at different weight ratios (MSC30-U1 and MSC30-U2) and heated in air at 350 °C. Then it was subsequently heat-treated at high temperatures (from 700 to 1300 °C) to tailor the amount and the type of the different nitrogen species in the final carbon structure. Figure 5 illustrates the results obtained. It was proposed that (i) pyridones and pyrrolic N species convert to pyridine species at temperatures above 600 °C through the loss of oxygen from pyridones and the expansion of pyrrole rings, as previously reported by Pels et al.;^213^ (ii) an increase in the total graphitic N content at high temperatures was explained by converting pyridines and pyrrole/pyridone species into graphitic nitrogen groups; (iii) the surface nitrogen species were completely removed from the carbon surface at 1100 or 1300 °C, depending on the carbon sample. A similar approach was earlier used to treat polyaniline samples.^237^

**Figure 5:**
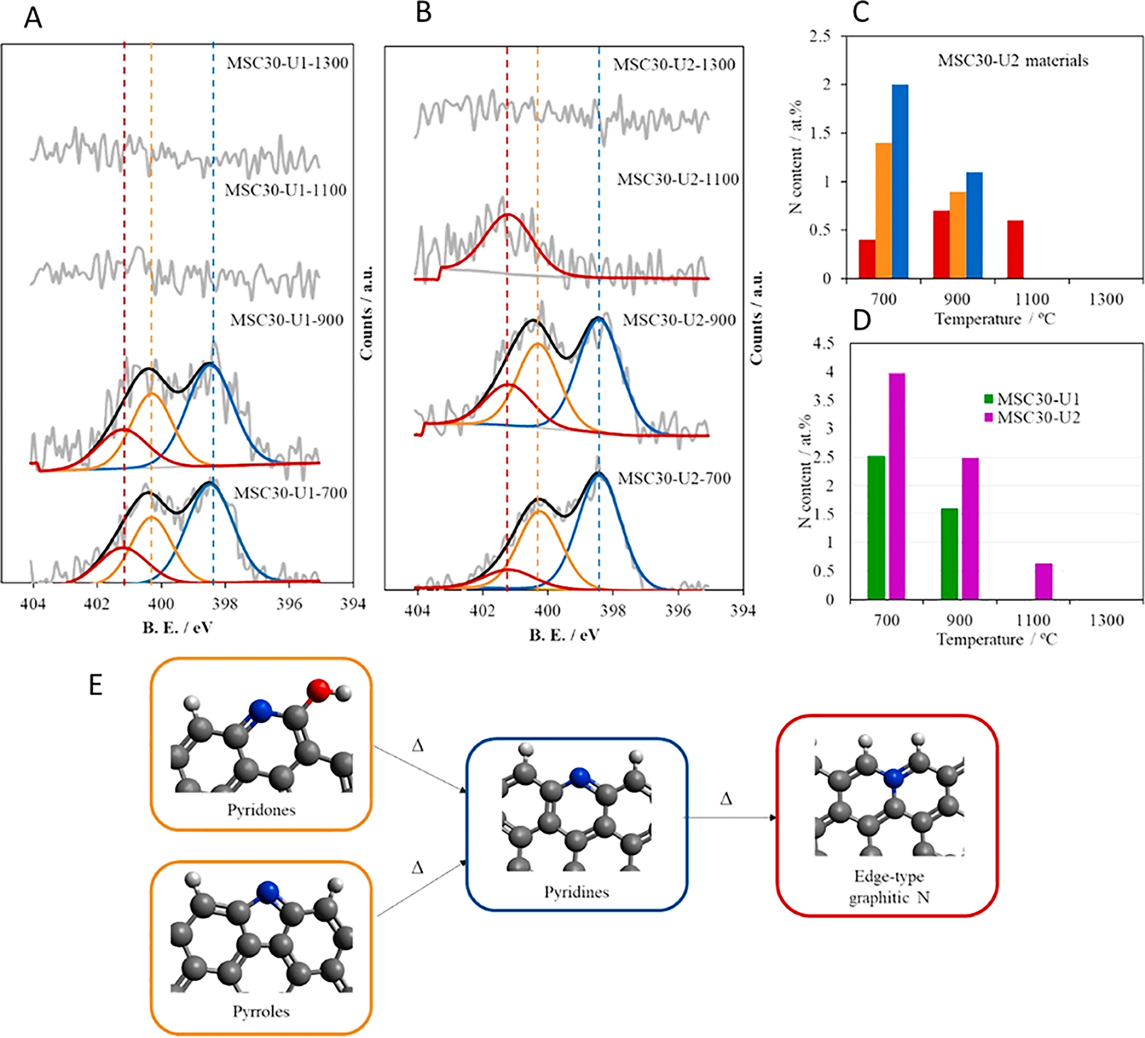
A) and B) N 1s spectra of (A) MSC30-U1, and (B) MSC30-U2 samples after heat treatment at 700, 900, 1100, and 1300 °C. The blue peak at 398.3 eV represents pyridines, the orange peak at 400.3 eV represents pyrroles or pyridones, and the red peak at 401.2 eV represents graphitic nitrogen species; C) Contributions of each kind of nitrogen species in the MSC30-U2 samples; D) Surface N content determined by XPS in the heat-treated samples; E) Schematic illustration of the conversion of pyrroles and pyridones into pyridines and the conversion of pyridines into edge-type graphitic nitrogen. Reproduced with permission from ref.^236^ Copyright 2022, Elsevier.

#### 3.2.3 Sulfur Functionalization

Sulfur groups can be introduced through thermal treatments with sulfur-containing gases, hydrogen sulfide (H_2_S) or sulfur dioxide (SO_2_) being the most used, or by directly mixing a sulfur-containing solid (including elemental sulfur) with carbon, followed by heating. Puri^238^ reported the formation of C–S complex with sulfur containing gases, such as H_2_S, CS_2_, SO_2_, and sulfur vapor at 100–1000 °C, but the heat treatment is typically conducted at 400–800 °C for 1–4 h. Different degrees of functionalization can be achieved, depending on the properties of the initial carbon, the S-precursors, and the experimental conditions selected, with the temperature playing a key role. Sulfur content up to around 10% was achieved by heating activated carbons at 900 °C in the presence of H_2_S.^239^ It was proposed that H_2_S reacts with quinonic and phenolic groups to form thioquinone and thiophenol groups, and the loosely bonded sulfur attaches to less reactive unsaturated sites, potentially leading to the formation of sulfide and hydrosulfide groups.^238^

#### 3.2.4 Phosphorus Functionalization

Phosphorus groups are mainly introduced by the wet methods described above. In dry methods, they can be introduced via thermal treatments with phosphorus-containing gases, such as phosphine (PH_3_) or phosphorus trichloride (PCl_3_), or by mixing phosphorus with carbon followed by heating. Safety considerations should be carefully considered if these phosphorus-containing gases are used due to their toxic, corrosive, and reactive nature. Most of the works using these gases focuses on obtaining P-doped carbon nanomaterials. For example, Pumera et al. studied the codoping of graphene oxide with phosphorus and halogens by exfoliating different graphite oxides by a thermal shock at 1000 °C in an atmosphere of various phosphorus halides (PX_3_, i.e., PCl_3_, PBr_3_, and PI_3_).^240^ Another approach to obtain P-containing surface groups is by a chemical vapor deposition using gas mixtures, for example of CH_4_ and PCl_3_, toluene and triphenylphosphine, pyridine and triphenylphosphine.^215^

#### 3.2.5 Boron Functionalization

Boron-doped porous carbons are mainly obtained using boric acid as a boron source, involving an impregnation step followed by a thermal treatment. Nevertheless, boron groups can be introduced through a chemical vapor deposition (CVD) or other thermal synthesis processes using boron-containing sources that decompose at high temperatures to release boron atoms that can be incorporated into carbon materials. This approach has been used mostly to obtain B-doped nanocarbons like graphene, carbon dots, carbon nanotubes and nanodiamonds. Some examples of boron-containing vapors commonly used in these processes are diborane (B_2_H_6_),^241^ boron trichloride (BCl_3_),^242^ boron tribromide (BBr_3_),^243^ trimethylborane (B­(CH_3_)_3_),^244^ triethylborane (B­(C_2_H_5_)_3_),^245^ and triphenylborane.^246^ These boron-containing compounds are chosen based on their volatility, reactivity, and compatibility with the synthesis process to achieve the desired level of boron doping in carbon materials.

### 3.3 Carbon Precursors as a Source of Heteroatoms

#### 3.3.1 Synthetic Polymer-Derived Carbons

Besides functionalization and doping of porous carbons post their synthesis by using various liquids or gases having target heteroatoms in their chemical formulas, as discussed in Sections 3.1 and 3.2, so-called self-doping or self-functionalization is another commonly used approach. In this carbon synthesis path, the precursors have heteroatoms in their built-up. Polymers and specific biomasses are examples of such carbon sources. In the case of polymers, both porosity and surface chemistry can be modulated using particularly designed approaches. Regarding the porosity, and very briefly, since it is not the scope of this review, template syntheses are the most explored routes to provide polymer-derived carbons of different, and even highly ordered structures. These textural properties are important since they determine the availability and dispersion of heteroatoms incorporated to the polymer-derived carbon matrices. Generally, the template should be chemically inert and relatively easy removed by washing either with water (best case scenario), acid or alkaline solutions, or even by vaporization. Its physical properties such as sizes of crystals or particles, sizes of pores, volume of pores are determining factors for the porosity of resulting carbons; however, not always the porosity of carbons is an exact reverse image of the porosity of the template due to the rearrangement of carbon atoms within the carbon matrix or its shrinking during a high temperature treatment.

Deriving carbons from polymers or other organic materials has already been of continuous research interests for few decades. Various inorganic templates, including colloidal silica,^247–250^ ordered silicas,^251^ aluminosilicates and zeolites,^252,253^ nanostructured metal oxides, NiO, MgO, CaO, CeO, among others,^254,255^ or metal salts (examples are NaCl, KCl, ZnCl_2_, Na_2_SO_4_)^256,257^ have been used. The reader is directed to the numerous reviews on this topic providing details on the synthesis procedures resulting in a specific porosity, depending on the template type, its chemistry and morphology.^5,258–262^

Another synthesis approach leading to the development of a specific porosity, and also chemistry, in polymer derived carbons, especially those of an ordered structure, relies on using polymer nanostructures as sacrificial templates. The polymers are removed either by dissolution or calcination and the latter is often combined with a carbonization process.^263,264^ Here, the examples of the sacrificial templates are polystyrene^265^ or block copolymers.^266,267^

Besides the porosity, the morphology of polymer derived carbons can be also controlled. The examples are carbons spheres synthesized by a modified Stober-based method by Zhao et al. from dopamine via the formation of specific microemulsions in a waste/ethanol system.^268^ Another important methodology and relatively easily controlled, owing to the properties of some polymers, is the method leading to fibrous systems, including 2-D structure and mats. In this approach, electrospinning and a chemical vapor deposition are explored. In the former, PAN, polyimide, and poly­(vinyl alcohol) are often used to produce fibers followed by their carbonization, which could be carried out with activators/heteroatom-providers, as for instance H_3_PO_4_, as explored by Wu et al.^269^ whose carbon fibers were both N and P doped.

The porosity in polymer derived carbons can be also developed due to intrinsic properties of the polymers, which can contain specific functional groups decomposing during carbonization and acting as pore formers or even containing metal ions in the form of polymeric salts. Examples are highly porous carbons derived from polystyrene sulfonic acid/maleic acid salts.^270^ Another approach often used is more traditional and involves the chemical activation of polymer-/organic compound-derived chars, and here CO_2_, H_2_O, and KOH are commonly used as activation agents.

Even though the direct control of the porosity in polymer derived carbons using the hard or soft templating is certainly their important asset, the possibility of the spatially homogeneous introduction of heteroatom-containing groups or their doping into the carbon matrix during polymer carbonization is also of paramount importance and it is one of the factors directing the attention of researchers to these synthesis routes. To achieve rich and homogeneous surface chemistry, either template carbonization or a direct synthesis can be utilized. An important requirement is the presence of heteroatoms in the polymeric precursor and here nitrogen and sulfur are the targets, owing to their presence in organic compounds/polymers.

The most commonly used polymers providing nitrogen containing carbons are those referred to as PAN (polyacrylonitrile), PI (polyimide), PAM (polyacrylamide), PVP (polyvinylpyrrolidone), PoPDA (poly­(o-phenylenediamine)), PPy (polypyrrole), PANI (polyaniline), PDA (polydopamine), and imidazolium-containing PILs (polymeric ionic liquids). Examples are nitrogen rich ordered carbons prepared in a SBA-15 template from polydopamine (Figure 6A)^251^ or ordered mesoporous carbons obtained via the block copolymer-templated high-temperature carbonization of nitrogen-containing polymeric precursors (N-containing oligomeric precursor amic acid (AA)) and a stabilizing cross-linker, resol.^267^ In the latter, as in other methods, the content of nitrogen decreased with an increase in the carbonization temperature and its removal from the matrix was indicated as leading to the formation of structural defects. An important asset of some organic precursors is the possibility of using a specificity of their condensation and thermal transformation processes for a chemistry evolvement. Here, an example could be supramolecular carbonaceous materials prepared from eutectic mixtures of cyclohexanehexone and urea, where formation of a cross-linked intermediate and its thermal treatment in an inert atmosphere led to nitrogen-functionalized carbons containing primarily pyridinic and pyrazinic nitrogen.^271^ Some carbons derived from ionic liquids can exhibit “noble carbons” resistance to oxidations^272^ or provide a good performance for sodium-ion batteries owing to their porosity and relatively high nitrogen content.^273^

**Figure 6:**
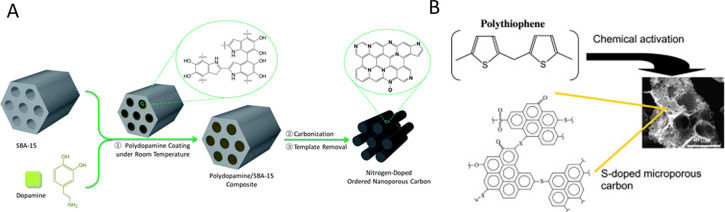
A) Schematic illustration of the polydopamine-coating-directed synthesis of NONCs. Reproduced with permission from ref.^251^ Copyright 2013, the Royal Society of Chemistry; B) TOC graphics scheme showing the synthesis and surface chemistry of S-doped carbons derived from Polythiophenes. Reproduced with permission from ref.^274^ Copyright 2012, Elsevier.

The most often explored source of sulfur-self-doping in polymer-derived carbons, and not originating from the modification additives, is polythiophenes (PTh) (Figure 6B).^274,275^ Salts of polystyrene sulfonic acids are other examples.^270,276^ In this case, the speciation of sulfur depends on the temperature of the heat treatment and a variety of groups can be introduced, including sulfoxides/sulfones/sulfonic acid as well as sulfur in thiophenic configurations. In the case of the polymeric salts, when ammonium salts are used, both N and S can be directly incorporated to the carbon matrix.^277^ Thiophene and thiophene-based compounds are also important sources of S-doped polymer derived carbons. Here, the examples are 2-thiophenemethanols^278^ that need to be polymerized with FeCl_3_. Their carbonization leads to S incorporated to thermally stable 5-ring sulfides.

#### 3.3.2 Biomass-Derived Carbons

While addressing self-doped carbons, one cannot forget biomass as their precursors. The complex chemical composition of biomass of different origins, their high carbon content and low costs make them attractive candidates for the production of heteroatom-doped carbons. Here, to introduce a sufficient quantity of heteroatoms of specific chemistry, biomass rich in those heteroatoms should be chosen. Examples of biomass that contains more than 3% of nitrogen are various kinds of sludges/animal waste, waste coffee (owing to caffeine) and aquatic biomass such as *Spirulina* or *Nanochloropsis Oceanica* or *S. platensis*, which have over 7% of nitrogen.^279^ Those particular biomasses are also relatively rich in sulfur, with more than 0.5% of this element. Nevertheless, to get porous carbons from these materials, the conventional methods of physical or chemical activation need to be applied. Examples are carbons obtained from algae with 2.9% of nitrogen and a marked capacitive performance^280^ or those derived from seaweed suitable for high voltage supercapacitors in neutral electrolytes.^281^ Spent coffee, owing to its high nitrogen content, has been also shown as suitable self-doped carbons precursor, deriving carbons being able to provide high capacitance^282^ or to convert the marked amount of H_2_S into elemental sulfur.^283^

### 3.4 Defunctionalization

Surface defunctionalization is a methodology for tailoring the surface chemistry of porous carbons, which involves a deliberate removal or alteration of specific functional groups. The initial sample is usually selected to have a large amount of surface groups. This approach has been primarily used for oxygen and, to a lesser extent, for nitrogen or phosphorus surface-containing groups. In certain cases, the process not only removes existing functional groups but also induces the formation of new ones, which then qualifies as a functionalization method. This section focuses only on the defunctionalization of oxygen-containing groups. Heat treatments involving other heteroatoms (e.g., nitrogen or phosphorus) were covered in Section 3.2.

A thermal treatment is the most widely used method for surface defunctionalization of O-containing groups. By heating porous carbons under an inert (e.g., nitrogen, argon) or reducing (e.g., hydrogen) atmosphere, heteroatom-containing groups are decomposed, releasing specific gases. The temperature and duration of the thermal process determine the extent of defunctionalization. Figure 7 clearly illustrates what happens in the case of oxygen-containing surface groups. Starting from a carbon material previously treated with nitric acid (sample CNT-N) to introduce a significant amount and variety of functional groups, it was observed that with a mild heating up to from 200 to 400 °C, the carboxylic groups decomposed (releasing CO_2_); with a moderate heating from 400 to 600 °C all the anhydrides were removed (releasing CO and CO_2_); applying high temperatues, from 600 to 900 °C all lactones (releasing CO_2_) and phenol (releasing CO) were removed and only a few basic groups like carbonyl and quinones (releasing CO) remained. Temperatures higher than 900 °C are used for a thorough defunctionalization. Using heat treatments at temperatures below those used in the synthesis of the starting carbon materials usually leads to samples with a low variation in the textural parameters.^119^ Samples heat-treated under inert atmospheres are very susceptible to reoxidize after cooling down, when exposed to air and moisture. Using H_2_ in the heat treatment or in the final treatment at high temperatures stabilizes the reactive sites formed at the edges of graphene layers due to the oxygen surface group decomposition.^141^

**Figure 7:**
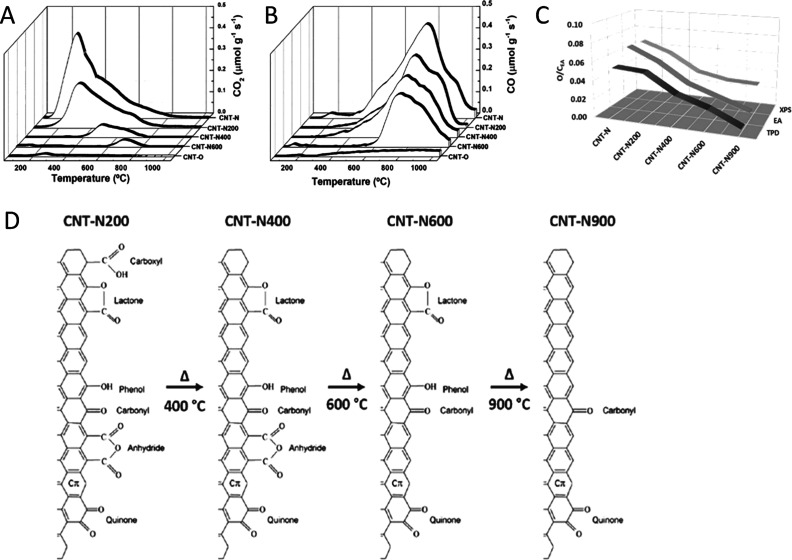
Profiles of (A) CO_2_ and (B) CO evolved in TPD-MS of pristine CNTs (CNT-O) and modified samples: nitric acid (CNT-N) and thermally treated (CNT-N200, CNT-N400, CNT-N600); C) Functionalization degree of preoxidized and thermally treated CNT samples by different characterization techniques; D) Schematic illustration of O-containing groups released from the surface of oxidized CNTs during thermal treatment under inert atmosphere. Reproduced with permission from ref.^118^ Copyright 2023, Elsevier.

### 3.5 Bottom-Up Synthesis of Porous Carbons

There are two main approaches for the synthesis and functionalization of nanomaterials, namely bottom-up and top-down. The top-down methods introduce the desired surface functionalities/dopants (heteroatoms, metallic groups) by exposing the carbon material to a chemical compound acting as source of the heteroatom (as examples: ammonia for N-groups, H_2_S for S-groups, HNO_3_ for O-groups), as addressed above, while bottom-up methods use individual atoms/molecules and build them up into large nanomaterials. Top-down methods are frequently used since they are suitable for large scale synthesis, but very often O-groups are incorporated to the carbon material in addition to the target heteroatom,^284–287^ making the control in the composition, the correlation of performance and the effect of the surface chemistry challenging issues. On the other hand, the bottom-up approaches offer the advantage of enabling a selective introduction of the target surface groups by using precursor molecules with the desired functional groups as building blocks (Figure 8).^195,288–293^ The resulting carbon materials typically display a more homogeneous chemical composition.

**Figure 8:**
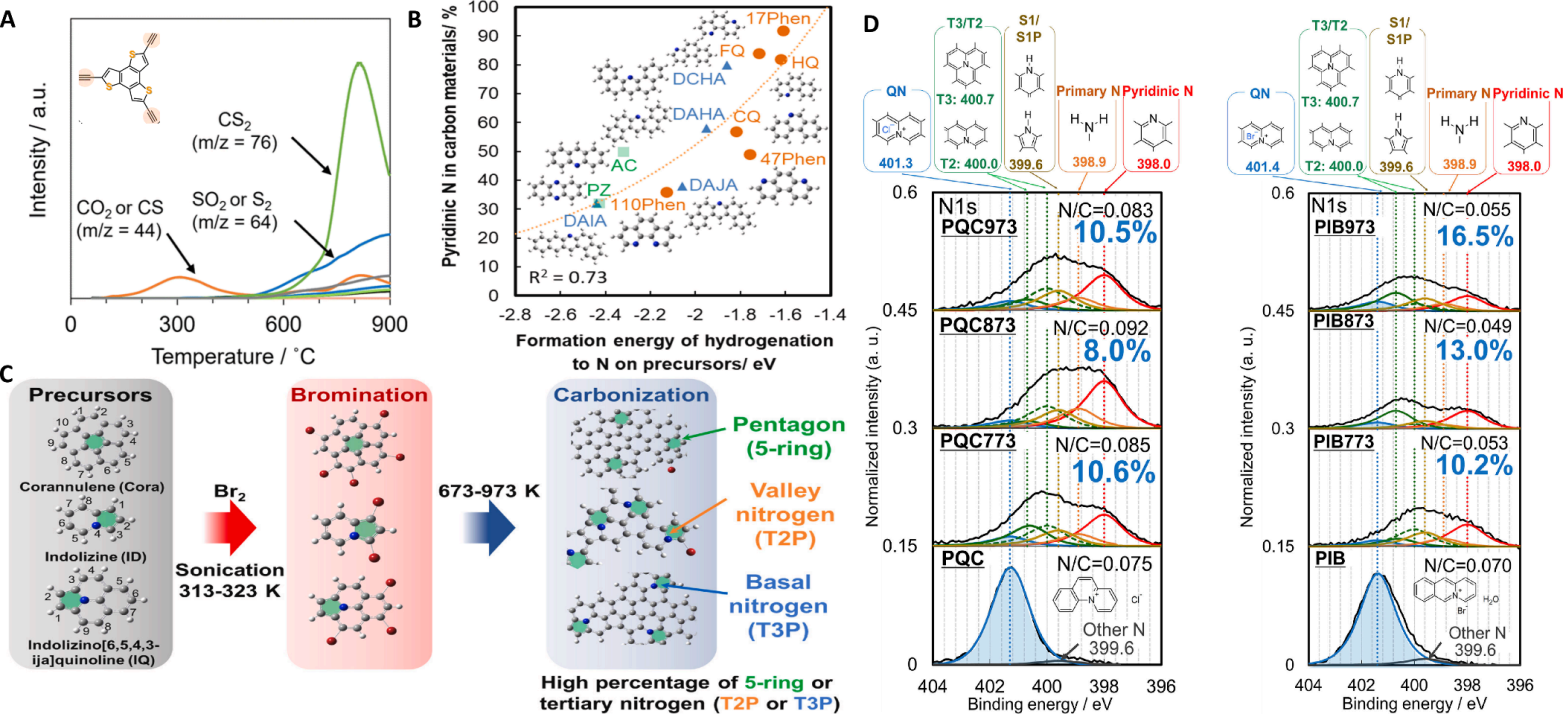
A) TPD-MS profiles of a highly S-doped porous carbon obtained from 2,5,8-tri­(triethynyl)­benzo­[1,2-b:3,4-b:5,6-b″]-trithiophene as precursor. Reproduced with permission from ref,^315^ Copyright 2024, the authors under CC-BY 3.0 licene; B) Correlation between the percentage of pyridinic N in carbon materials prepared at 700 °C and the formation energy of hydrogenation to N on the precursors. Reproduced with permission from ref.^325^ Copyright 2022 Elsevier; C) Structures of precursors and ideal carbonization of brominated compounds with pentagons and tertiary nitrogen. Blue sphere: nitrogen atom. Gray sphere: carbon atom. Red sphere: bromine atom. White sphere: hydrogen atom. Reproduced with permission from ref.^323^ Copyright 2023, Elsevier; D) N 1s XPS spectra of N-doped carbons (PQC and PIB) heated at 500–700 °C, with indication of the percentage of quaternary nitrogen. Reproduced with permission from ref.^196^ Copyright 2022, Elsevier.

Bottom-up methods for the synthesis of carbon materials are based on the carbonization and/or polymerization of the precursors (some examples are aromatic hydrocarbons, polysaccharides, melamine, thiophene, urea, furfuryl alcohol, ...) using various methods such as a chemical vapor deposition, solution synthesis, coprecipitation, sol–gel approaches, or hydrothermal/solvothermal methods, among the most representative.^294^ The reader can find numerous reviews providing a detailed analysis on the role of different synthesis parameters (temperature, time, ratio of precursor, pH, ...) on the composition, porosity and nature of surface groups on carbon materials.^291,295,296^ In this section, we will describe some of those works leading to the control of the surface chemistry of the resulting carbon materials.

Biomass-derived saccharides (glucose, fructose, sucrose, glucosamine hydrochloride, cyclodextrins, xylose, arabinose, ...) are most commonly used building blocks for the synthesis of carbon materials through bottom-up techniques, mainly using hydrothermal and soft-templating methods.^297,298^ Both strategies often lead to the synthesis of porous carbons with controlled pore architectures and high contents of heteroatoms. In addition, the use of surfactants combined with the molecular precursor often leads to the synthesis of carbon materials with an ordered hierarchical porosity.^299–301^ Recently, synthetic efforts have been focused on the use of bottom-up methods combined with lithographic and additive manufacturing methods, yet most attention is directed to describing the porosity and mechanical characteristics of these materials, with little attention given to their surface chemistry.^302–306^

In general, carbons obtained from polysaccharides display moderate oxygen contents, with a hydrophobic core with cross-linked furanic and ether or keto-aliphatic chains, and a hydrophilic external shell with a high density of oxygen surface groups of acidic nature (i.e, hydroxyl, carbonyl, or carboxylic). The presence of other heteroatoms depends on the choice of the composition of the precursor molecules (urea, thiourea, melamine, chitosan, pyrrol). As examples, carbons prepared from the hydrothermal carbonization of glucose and chitosan mixtures have been reported to display both acidic (carboxyl) and basic (amines) groups;^307^ the hydrothermal carbonization of polysaccharides with sulfuric acid and sulfonic acids (e.g., hydroxyethylsulfonic, p-toluenesulfonic, dodecylbenzenesulfonate) renders carbon materials with relatively high sulfur contents (mainly oxidized sulfur forms).^308–310^ The use of mixtures of carbon precursor molecules with other functionalized molecules (thiourea, melamine) allows to obtain multifunctional carbon materials containing various heteroatoms.^311–313^ As an example, Tian et al.^311^ reported the synthesis of carbon materials with a large specific surface area and high content of N/O/S heteroatoms. The resulting carbon framework displayed interesting polarity linked to those heteroatoms, and good performance as electrodes in supercapacitors and for the separation of CO_2_/N_2_ gas mixtures.^311^ Zhang et al. reported a similar effect of an uneven electrostatic potential distribution in B,N-co-doped carbon porous membranes.^314^

Chida et al.^315^ reported a bottom-up fabrication approach for the preparation of S-rich porous carbons from molecular precursors via the carbonization of molecules with thermally stable S-containing building blocks and polymerizable ethynyl moieties (Figure 8A). The thermal treatment of those precursors yielded microporous carbon materials with an unprecedentedly high S content (above 15 wt %). The authors described the role of the S moieties as effective anchoring sites for single-atomic platinum (Pt) species, with high electrocatalytic performance as anodes in PEFCs.^315^

The polycondensation of hydroxylated aromatic molecules (e.g., resorcinol, phloroglucinol, furfuryl alcohol, gallacetophenone, pyrogallol, tannic acid) in the presence of aldehydes (formaldehyde, glyoxal) or surfactants has been extensively investigated for the preparation of porous carbons through soft-templating and sol–gel approaches.^295,313,316,317^ The resulting porous polymeric materials contain abundant oxygen functionalities before carbonization. Further carbonization converts them into carbon materials with moderate oxygen contents, predominantly in the form of furanic and ether-type moieties.

The versatility of building blocks incorporating functionalized molecules (e.g., melamine, pyrrol, urea, thiophene, 2-hydroxymethylthiophene, 3-hydroxypyridine) allows the synthesis of functionalized carbons with targeted heteroatoms.^318–321^ Recently, Yamada and co-workers have developed bottom-up approaches based on the thermal decomposition of suitable precursors for the synthesis of carbon materials with a high nitrogen content and selectivity toward the nitrogen moieties (pyrrolic, pyridinic, pentagons, tertiary) (Figure 8B–D).^195,196,322^ Given the different reactivity and surface charge density of N-groups, the synthesis of carbon materials with selective N-moieties gained increased interest in catalytic applications (see Section 4.4). The authors reported selectivities of up to 94% in the functionalization of carbons with pyrrolic groups using brominated carbazole derivatives as precursors. They also claim the need to use low carbonization temperatures to stabilize the pyrrolic moieties, and the position and the number of the bromo groups of the precursor to control the selectivity.^195^ Ultrasonic bromination of an organic precursor (e.g., corannulene, indolizine, indolizino) allowed to obtain carbon materials with controlled structures and high amounts of nitrogen in pentagon and tertiary configurations (Figure 8C,D).^196,323^ Similarly, Tian et al. have reported single-site pyrrolic nitrogen doped carbons with nitrogen content up to 4 at. %, based on a low temperature two-step pyrolysis of halogenated phenol-formaldehyde co-condensed resins.^324^

A recent work by Li et al. has reported a self-templated method to synthesize boron-doped porous carbons from 2LiBH_4_·CO_2_ and CO_2_ as precursors, with a content of boron of ca. 6.6 wt %. The material displayed excellent lithium storage performance and cyclability.^326^

Exotemplating (hard-templating) approaches based on sacrificial scaffolds have also been largely investigated to prepare porous carbons. Typical sacrificial scaffolds are zeolites, mesoporous silica/oxides, opals, colloidal silica suspensions, and MOFs,^6,253,254,327,328^ with chemical vapor deposition (e.g., ethylene, propylene) and impregnation/carbonization of a monomer (e.g., furfuryl alcohol, glycerol, pyrrol) as the most common synthetic routes. The resulting templated carbons display unique properties with exceptionally high surface areas (porosity is defined by the scaffold) and a high density of edge sites (ca. 10-times higher for type-I zeolite templated carbon than in conventional activated carbons). Most of the carbon edge sites in templated carbons are naturally terminated by oxygen-functional groups in the resulting material, due to the exposure to oxygen or water upon the removal of the template. However, at the carbon/zeolite composite stage, they exist as dangling bonds, making further functionalization possible by exposure to gases at high temperature.^329,330^

Besides the ubiquitous O-groups of zeolite templated carbons, the incorporation of other heteroatoms such as nitrogen, boron, and sulfur has been reported through the choice of adequate precursors (acrylonitrile, pyrrol, ammonia, dimethylamine borane, thiophene-derivatives, poly­(styrene sulfonic acid-*co*-maleic acid) sodium salt, ethylenediamine, 2-thiophenemethanol, 1-ethyl-3-methylimidazolium tetracyanoborate).^221,253,331–334^ As an example, Castro-Muñiz et al. used an ionic liquid (1-ethyl-3-methylimidazolium tetracyanoborate) as boron and nitrogen source to obtain boron and nitrogen codoped ordered microporous carbons with high surface areas using NaY zeolite as a scaffold.^221^

Another example of bottom-up synthesis is that leading to ordered carbonaceous frameworks (OCFs). They are three-dimensional ordered structures synthesized by carbonization of metalloporphyrin crystals with polymerizable moieties.^335^ An important feature of these materials is single metal atoms (M-N_4_) incorporated to the matrix with a regular arrangement. OCFs have an ordered structure and are composed of nonstacked graphene sheets and exhibit a high conductivity and chemical/thermal stability.

## 4 Effect of Surface Chemistry on a Targeted Performance of Carbon Materials

### 4.1 Removal of Gases

The range of gases and vapors that are separated from various media on activated carbons is enormous. Since this review is rather oriented to the environment–energy nexus, we limit our scope to the role of carbon surface chemistry in the removal of a relevant and rather narrow range of species. We focus on pollutant gases produced either by combustion of fuels or existing as fuel contaminants, and on those affecting the outdoor and indoor air quality. We have also chosen them since their physical adsorption is rather weak and for their efficient retention on the carbon surface an enrichment of adsorbent surface chemistry is needed. Examples of approaches used are presented below.

#### 4.1.1 NOx Abatement

Porous carbons have been explored as catalysts for removal of nitrogen oxides from flue gas, in particular via selective catalytic reduction (SCR) of NO with NH_3_ and via direct NO oxidation to NO_2_. Activated-carbon catalysts exhibit high de-NO_*x*_ activity in the NH_3_–SCR reaction (4 NO + 4 NH_3_ + O_2_ → 4 N_2_ + 6 H_2_O) at 100–200 °C, well below the 300–400 °C operating window of conventional vanadium-based catalysts. This low-temperature performance aligns with the exhaust temperatures of coking, steel, cement, ceramics, and industrial boilers, whose flue gases are typically released at 120–300 °C. The identity of the active sites, however, remains controversial. Guo et al. correlated the degree of surface oxidation of ACs with their SCR rates and attributed the rate enhancement to carboxyl, anhydride and phenolic groups that enhanced NH_3_ adsorption.^336^ A comparable trend, relating an activity to the amounts of CO and CO_2_ evolved during TPD normalized by *S*_BET_, had been previously found for carbons treated with concentrated H_2_SO_4_.^337^ Both cases supported the earlier hypothesis that the reaction involved the formation of surface complexes, NH_3_ interacting with carboxylic acids, lactones and phenols to form CO^–^(NH_4_)^+^, and NO with carbonyl to form C­(ONO) (Figure 9A).^338^ Li et al.,^339^ using DFT calculations, supported the same mechanism, with the surface oxygen complexes changing the electronic structure of an adjacent carbon atom and promoting the reactants’ chemisorption.

**Figure 9:**
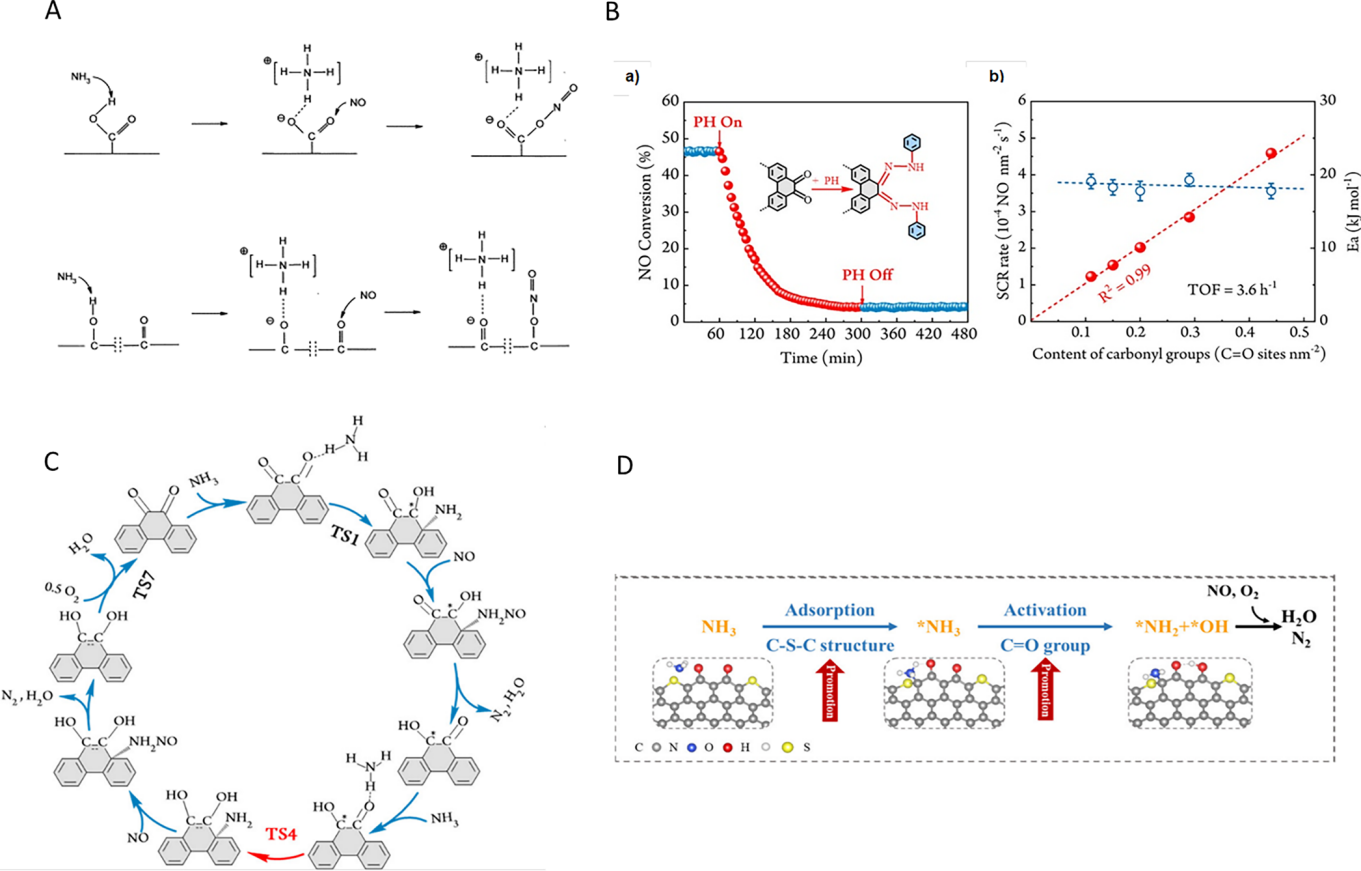
A) Reaction scheme for the formation of surface complexes during NO reduction with NH_3_ over carbon catalysts. Reproduced with permission from ref.^338^ Copyright 2001, Elsevier; B) (a) NO conversion on oCNTs-700 as a function of time during *in situ* passivation process, (b) SCR rate (red) and apparent activation energy (*E*a, blue) on catalysts with different contents of ketonic C=O groups. Reproduced with permission from ref.^340^ Copyright 2022, American Chemical Society; C) NH_3_–SCR reaction pathway on ketonic C=O groups over the carbon catalyst, TSi is the i transition state. Reproduced with permission from ref.^340^ Copyright 2022, American Chemical Society; D) Potential synergetic mechanism between the ketonic C=O group and the C–S–C structure for SCR over a metal-free carbon catalyst. Reproduced with permission from ref.^344^ Copyright 2024, American Chemical Society.

A recent proof-of-concept study by Li and collaborators^340^ provided a direct evidence through both *ex situ* and *in situ* selective passivation that nucleophilic ketonic carbonyl (C=O) groups are the active sites for the SCR on metal-free carbons. The authors followed a similar approach used by Su and co-workers,^341,342^ previously described for an oxidative dehydrogenation reaction, where they used phenylhydrazine to passivate the ketonic carbonyl groups. The SCR activity of the pristine and passivated CNTs was tested at 200 °C (lower than the decomposition temperature of CNT derivatives) and a pronounced decrease was obtained for the passivated samples. They have also conducted *in situ* selective passivation measurements by premixing phenylhydrazine with SCR reactants, and the NO conversion decreased dramatically when phenylhydrazine was introduced (Figure 9B-a). The NO conversion was not restored after turning off the phenylhydrazine addition, which indicated the irreversibility of passivation. By measuring a turnover frequency (using samples where the number of C=O surface groups was adjusted via an annealing treatment), the authors established the first quantitative structure–activity relationship for carbon SCR catalysts (Figure 9B-b), with a TOF value of 3.6 h^–1^. At the same time, the apparent activation energies almost did not change with an increase in the C=O content, meaning that the reaction pathway was the same. This study also included DFT calculations, supported by *in situ* DRIFTS and quasi-*in situ* XPS, and revealed that the reaction followed a redox cycle between ketonic carbonyl and phenolic pairs (see scheme in Figure 9C), in which a rate-limiting step is the activation of NH_3_ on a ketonic C=O site adjacent to C–OH (transition state 4 (TS4) in Figure 9C).^340^ Experiments on commercial activated carbon confirmed the same mechanism, underscoring the generalization of ketonic C=O as the active site. Beyond identifying the active center, the work offered a robust benchmark for comparing carbon catalysts with conventional vanadia systems and demonstrated analogous mechanistic features between metal-free carbons and transition-metal oxides. These insights can guide the design of high-efficiency carbon catalysts for low-temperature NH_3_–SCR.

Recently, Duan et al.^343^ proposed a slightly different mechanism. Combining DFT calculations and experimental observations (using a series of nitric acid oxidized activated carbons annealed at different temperatures), they proposed that the hydroxyl and epoxy groups are the key active species. However, the annealing temperature used for the most active sample (700 °C) is compatible with a relevant presence of carbonyl groups, which suggests that the experimental results would be aligned with the mechanism proposed by Li and collaborators described above.

Li and collaborators, in a follow-up study,^344^ assessed the effect of S-doping on the SCR activity over carbon catalysts and showed the synergetic mechanism between the C–S–C structure and the ketonic carbonyl group. The NO_*x*_ conversion rose from 19% (undoped) to 85% (S-doped). DFT calculations revealed that the C–S–C structure significantly changed the electronic structure of the C atom adjacent to the C=O groups, altering the NH_3_ adsorption configuration and capacity. S-doping also induced an extra electron transfer between the N atom of the NH_3_ molecule and the C atom of the carbon plane, promoting the activation of NH_3_ over the C=O group. Figure 9D presents a scheme of this approach.

N-doping, besides O-doping, is probably the most common approach to enhance the catalytic activity of carbon materials for NH_3_–SCR of NO. A common trend is that nitrogen functional groups increase the surface basicity of a catalyst, with pyridinic nitrogen (N-6) being proposed as the most active group,^345,346^ enhancing NO_*x*_ and NH_3_ adsorption and facilitating the oxidation of NO to NO_2_ and the activation of NH_3_ to NH_2_. Zhang and co-workers^347^ showed the synergistic effects of oxygen and nitrogen dual-dopants. Using DFT calculations, they proposed a new reaction pathway, in which N-quaternary enhances O_2_ activation and NO oxidation, and carboxyl groups (Bronsted acid site) mediate H transfer pathways, enabling the formation and decomposition of intermediate NH_2_NO with decreased energy barriers. This mechanism is similar to the one initially proposed by Teng et al.^338^

Boron doping of carbon aerogels^348^ was also demonstrated to be effective for NH_3_–SCR and the promoting effect was ascribed to the Lewis acid sites (BC3, BC2O, and BCO2 sites were identified), which play an important role in adsorption and dissociation of NH_3_ and O_2_, favoring NO reduction to N_2_. For P-doped samples,^349^ the proposed mechanism involved O_2_ adsorption on C3–P=O structures, its dissociation to O* that can readily interact with NO to generate NO_2_. Simultaneously, NH_3_ was coordinated on Brønsted acid sites (mainly C–OH and P–OH species) to form NH_4_^+^, then NO_2_ could react with NH_4_^+^ to generate an ammonium nitrite intermediate, which decomposed directly into N_2_ and H_2_O. The C3–P=O structure was observed to be unstable and oxidized to C–O–P during the reaction, leading to some deactivation.

Another technology for removing NO from flue gas is the oxidation of NO to NO_2_: 2 NO + O_2_ → 2 NO_2_. This is an interesting method since it can be carried out at room temperature in the presence of air and the NO_2_ formed, contrary to NO, is highly soluble in water, ultimately producing nitrate. Carbon materials can catalyze this reaction mainly through their basic surface sites (defects, π-electrons or oxygenated basic groups). Mochida et al., using pitch-based ACFs annealed at high temperatures (900 °C), observed an increase in the activity for NO oxidation compared to the nontreated samples, suggesting that defects created by removing oxygen functional groups during the heat treatment could be the active sites for this reaction.^350^ N-doping (which increases electron density on certain carbons) tends to improve NO oxidation. Sousa et al., using carbon xerogels containing nitrogen in their structure prepared by introducing melamine and urea in the sol–gel process,^351^ or N-doped activated carbons^352^ observed a linear relationship between the rate of NO converted normalized by the surface area and the amount of N-containing surface groups.

Compared to NH_3_–SCR, the NO oxidation has been less studied, and the reaction mechanism remains under debate, with an Eley–Rideal (reaction occurred between adsorbed NO and O_2_ in the gas phase) or Langmuir–Hinshelwood (NO and O_2_ both adsorbed) being proposed by several authors.^353^ An additional difficulty is that the strong oxidizing environment alters the oxygen functionalities during the reaction, affecting the stability of the catalyst.

#### 4.1.2 SO_2_ Oxidation

SO_2_ oxidation following this reaction SO_2_ + 1/2 O_2_ → SO_3_ (which then forms H_2_SO_4_ with water) on activated carbon is a classic carbon-catalyzed process, historically used in flue gas desulfurization. Activated carbons on their own catalyze this reaction at ambient temperature, combining adsorption and catalytic steps.^354^ Earlier studies sometimes gave conflicting interpretations of the role of surface chemistry, but a consensus has emerged that basic surface sites are crucial. In particular, N-doped carbons show an enhanced SO_2_ oxidation activity. Raymundo-Piñero et al.^207^ using activated carbons and activated carbon fibers demonstrated that introducing nitrogen increased carbon basicity and the SO_2_ uptake, with pyridinic N (N-6) sites being the most active both for SO_2_ oxidation to SO_3_ or to H_2_SO_4_. They obtained a linear correlation between catalytic activity (normalized per surface area) and the concentration of pyridinic N on a series of carbons (Figure 10A). This implied that pyridinic sites act as the active centers for O_2_ activation and subsequent SO_2_ oxidation. Acidic surface groups, on the other hand, were found to inhibit SO_2_ oxidation (likely by consuming the base sites or blocking adsorption).

**Figure 10:**
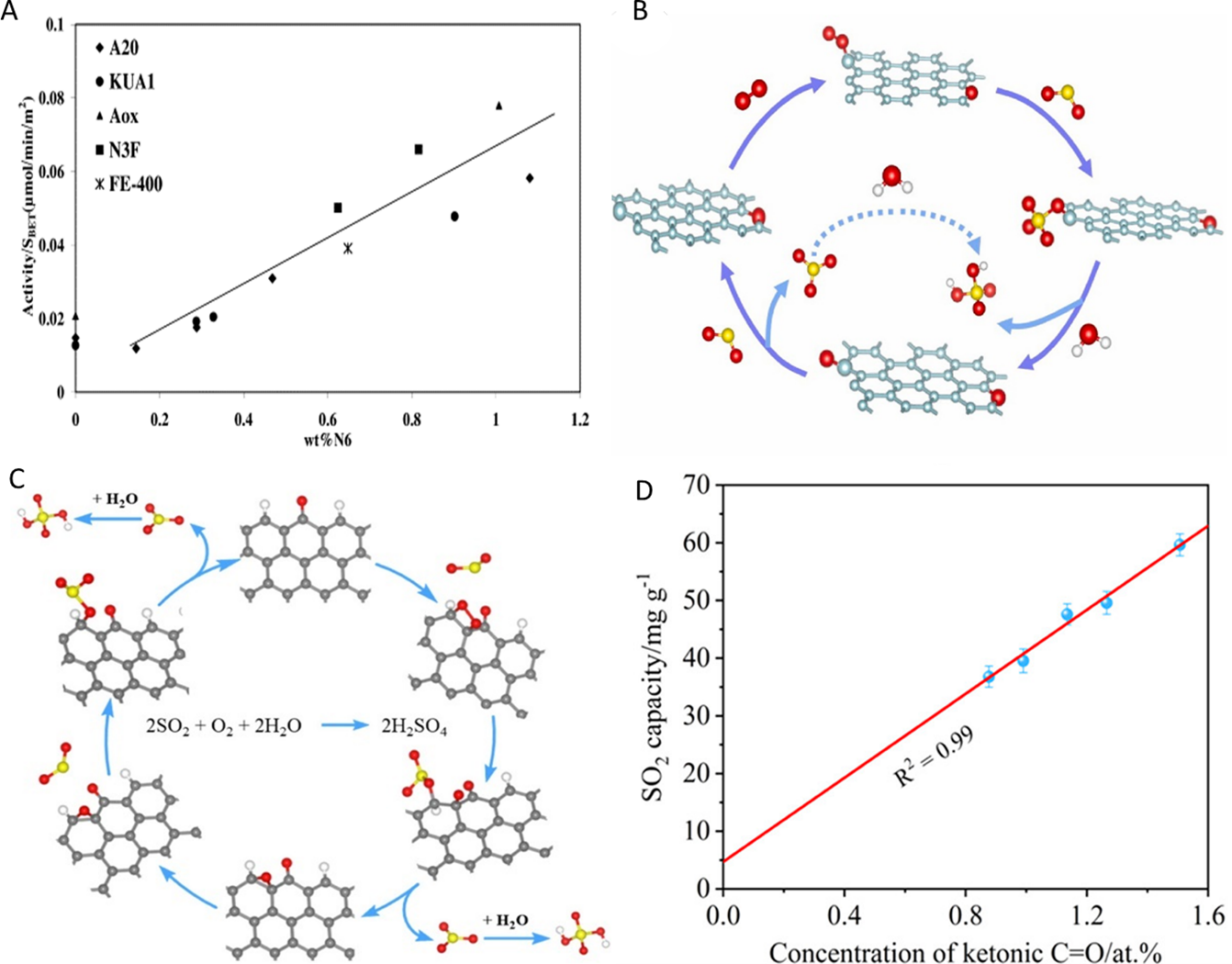
A) Activity normalized by surface area for SO_2_ oxidation into H_2_SO_4_ versus surface pyridinic-like nitrogen percentage for various porous carbon materials. Reproduced with permission from ref.^207^ Copyright 2003, Elsevier; B) Scheme of overall cycle of SO_2_ catalytic oxidation over oxygen-doped carbon cluster. Reproduced with permission from ref.^356^ Copyright 2023, Elsevier; C) Desulfurization reaction pathway on ketonic C=O groups over carbon. Reproduced with permission from ref.^355^ Copyright 2023 American Chemical Society; D) SO_2_ capacity of carbon catalysts with different amounts of ketonic C=O groups. Reproduced with permission from ref.^355^ Copyright 2023, American Chemical Society.

The mechanism of carbon-catalyzed SO_2_ oxidation is complex and has been debated, with an indication of different active sites. Two main trends have been followed: one involving carbonyl/quinone^355^ and others involving ester or epoxy^356,357^ surface groups as active sites, with surface defects being also included by several authors,^356^ claiming that the active sites can be generated *in situ* on these defects under the reaction conditions.^358^

DFT calculations allowed to conclude that nitrogen-^359^ and oxygen-doped^357^ carbons can efficiently catalyze SO_2_ oxidation and the active sites were ascribed to the carbon atoms near nitrogen or oxygen dopants, where O_2_ molecules are adsorbed and activated, accelerating the generation of highly chemically reactive O-containing species. Recently, Qu et al.^356^ by combining computational and experimental investigations using various oxygen-doped carbon clusters and site-specific descriptors considering a dynamic electron transfer effect, were able to show that the catalytic activity is determined by both oxygen groups and exposed edges. The active sites provide both strong electron donation ability and highly positive charge population. The proposed mechanism is (see Figure 10B): the carbon atom with the high electron donation ability first activates the O_2_ molecule to highly reactive intermediate C–O–O structure (O_2_^–^); subsequently, the formed C–O–O structure oxidizes SO_2_ via an O transfer process, which produces a sulfate occupying active site; assisted by a hydration process H_2_SO_4_ is formed, the C–O structure can be released and further oxidizes SO_2_ to SO_3_, after which the catalytic active site is recovered for a new catalytic cycle.^356^

Li and co-workers,^355^ by using experimental and theoretical methodologies for the SO_2_ oxidation, similar to those previously described for the NH_3_–SCR reaction, namely involving the selective passivation of different oxygen-containing surface groups, showed that desulfurization on metal-free carbon proceeds predominantly through a Langmuir–Hinshelwood pathway (see Figure 10C): nucleophilic ketonic C=O groups chemisorb SO_2_, while adjacent sp^2^-hybridized carbons dissociatively activate O_2_. Formation of H_2_SO_4_ was shown to be the reaction barrier step. Using a series of carbons with different numbers of ketonic groups but of comparable pore structures, the authors observed a linear increase in the SO_2_ uptake with the number of these groups (Figure 10D), establishing ketonic C=O moieties as the intrinsic active sites.

#### 4.1.3 H_2_S Oxidation/Reactive Adsorption

Activated carbons as media to remove hydrogen sulfide from various gaseous matrices have been studied for decades and the efforts of various researcher groups led to a marked improvement in understanding H_2_S-carbon surface interactions that resulted in very efficient separations/oxidation processes. Even though impregnation of porous carbons with various chemicals or modifications by the deposition of active inorganic species, such as metal oxides of alkaline nature or those capable of redox processes, have been certainly extensively explored to increase the efficiency of H_2_S separation process, these methods are beyond the scope of this review and the reader is directed to articles summarizing these approaches.^360–362^ Here, we will focus solely on the importance of surface chemistry for the intensity/extent of the reactions/interactions which take place on the surface of carbon adsorbents/catalysts and which govern specific separation processes. Nevertheless, the nexus of surface chemistry and porosity is critical here since a high volume of pores provides a storage for formed sulfur, contributing extensively to the efficiency of the catalytic performance.

Virgin carbon materials have a low activity for H_2_S adsorption or its catalytic oxidation.^363^ Therefore, various surface modifications are necessary and the incorporation of heteroatoms has been explored with a great success. Nitrogen and some oxygen functionalities have been indicated as providing a basic environment for H_2_S dissociation. An oxygen activation process leading to O* or/and O_2_•^–^ is another very important factor advancing the H_2_S separation. The high volume of pores is also a must to supply space to store sulfur formed in surface reactions.

Regarding oxygen activation sites in the carbon matrices, Chen et al.^364^ proposed that the edge (or defects) carbon atoms, due to an increased electron cloud density, have the ability to capture molecular O_2_ and to dissociate it into adsorbed oxygen (O*). Pan et al.^365^ indicated that the edge carbon defects promote the charge transfer from edged carbon atoms to molecular O_2_, which allows the O_2_•^–^ formation. Another mechanism of the oxygen activation is directly related to the incorporation of nitrogen to the carbon matrix. Even though this effect was mentioned in the early work by Stohr et al.,^232^ Strelko et al.^366^ calculated a band gap of pure carbon and nitrogen-containing carbons with different configurations of these species. Their results suggested that pyrrolic and pyridinic nitrogen lower the band gap of pure carbon, leading to an increase in a charge mobility which, in turn, efficiently promotes the activation of oxygen. Similar finding was reported by Pan et al.^365^ who indicated that the doped N atoms enhance a π* electron migration in sp^2^ carbon by conjugation due to localized unpaired electrons. As a result, an electron transfer from the carbon matrix to adsorbed O_2_ molecules takes place and O_2_•^–^ is formed. This analysis lacks the specificity of N-species involved in this process. It is possible that, due to altering the electronic structure of the carbon atoms in the vicinity of the nitrogen atoms, adsorption of O_2_ is enhanced and electrons are transferred to O_2_, resulting in its activation.^367,368^

Addressing the effect of nitrogen incorporated to the carbon matrix on the H_2_S removal, Bandosz’s group^208,210,373^ proposed that quaternary and pyridinic nitrogen configurations promoted the dissociation of H_2_S and their activity added up to the above oxygen activation ability. Yu et al. found a relationship between the H_2_S removal capacity and the N content in carbons.^370^ Sun et al.^374^ reported the relationship between the H_2_S removal capacity and the pyridinic N content. It was proposed that pyridinic N attracted H_2_S resulting in its dissociation into HS^–^ and, as a next step, the dissociated HS^–^ was chemisorbed on the carbon atom adjacent to pyridinic N to form a stable compound^369^ (Figure 11A), which was oxidized by oxygen to elemental sulfur, with the pyridinic groups converted to pyridones. The important role of pyridine nitrogen was supported by Yu et al.^370^ who calculated the binding energies of H_2_S with various N configurations (Figure 11B). They found that the binding energy with pyridinic N was much higher than those with other N configurations. Amine groups were indicated as promoting the dissociation of H_2_S by Wang et al.^375^ and Sun et al.^376^

**Figure 11:**
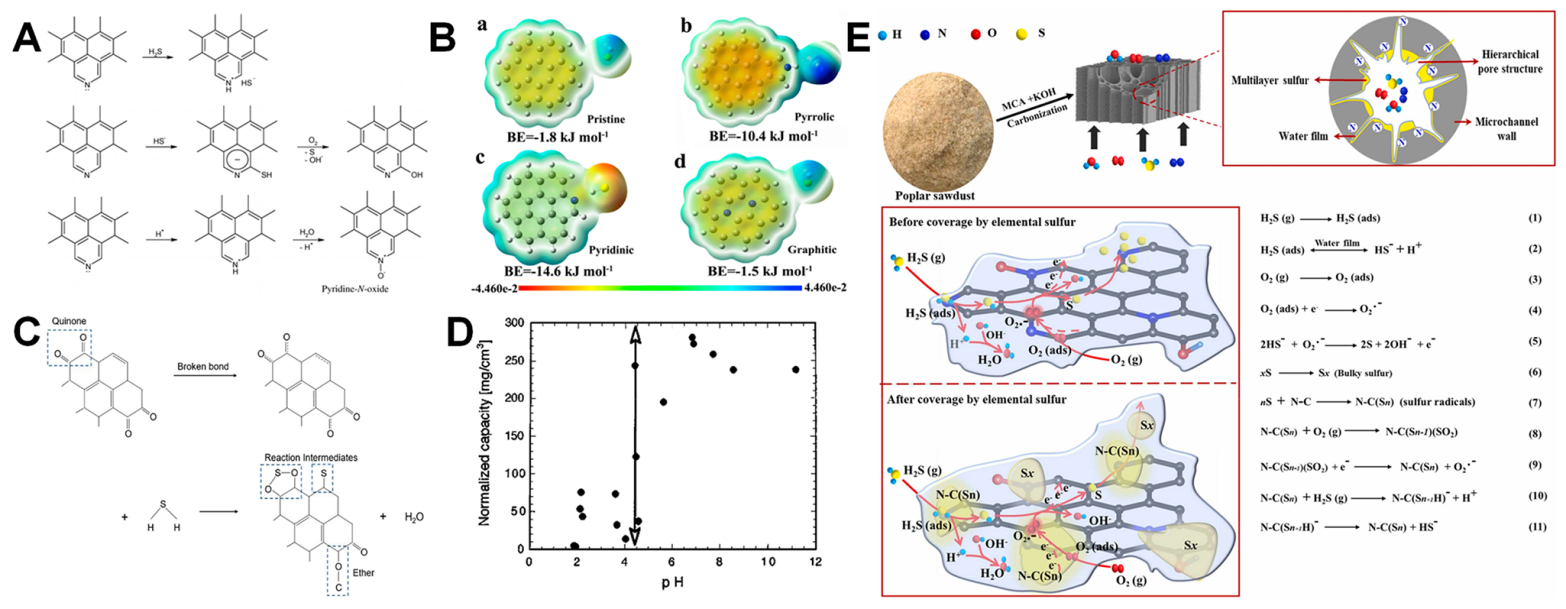
A) Possible surface reaction paths. The condensed aromatic rings represent the fragments of graphene layers. Reproduced with permission from ref.^369^ Copyright 2007, American Chemical Society; B) The atomic geometry of H_2_S molecule binding on (a) pristine carbon surface, and carbon surface doped with (b) pyrrolic N, (c) pyridinic N and (d) graphitic N. The gray, yellow, blue and white spheres are C, S, N, and H atoms, respectively. Reproduced with permission from ref.^370^ Copyright 2015, Elsevier; C) Changes of the functional groups on AC during H_2_S oxidation. Reproduced with permission from ref.^371^ Copyright 2019, Elsevier; D) Normalized H_2_S breakthrough capacity versus pH of carbon surface. Reproduced with permission from ref.^363^ Copyright 2001, Elsevier; E) Schematic illustration of the catalytic oxidation of H_2_S on the NBACs. Reproduced with permission from ref.^372^ Copyright 2022, Elsevier.

Besides nitrogen, oxygen groups were also found as playing an important role in H_2_S oxidation on the carbon surfaces. While basic groups enhanced the H_2_S removal process, acidic oxygen groups inhibited it.^208,208,377^ A correlation between the H_2_S removal efficiency and the content of basic oxygen groups was reported by Nowicki et al.^378^ An important role of these groups in the H_2_S retention was also indicated by Feng et al.^379^ It was also reported that pyrones and benzoquinones, enhanced H_2_S catalytic oxidation due to their ability to transfer electrons from the carbon matrix to adsorbed oxygen.^380,381^ Interestingly, quinones and carbonyl groups were indicated as reacting with H_2_S.^371^ In this process, C=O bonds and C=C bonds were broken and S–O and C–S bonds were formed as intermediates (Figure 11C). While the latter led to the formation of elemental sulfur, the former was converted into SO_2_. Moreover, the authors proposed that the oxygen groups could participate in oxidation of SO_2_ to sulfate; however, no specific groups were named.

By studying H_2_S removal at ambient conditions on a series of virgin carbons, it was suggested that an average surface pH might be an indication of the ability of carbons to retain H_2_S.^208,363^ At the pH value smaller than the experimentally established threshold of 4.3, adsorption/oxidation of H_2_S was limited due to the small extent of its dissociation (Figure 11D). A higher pH led to more HS-ions, which could form either SO_2_ (when small micropores were present) or elemental sulfur at supermicropores/mesopores. That pH is directly linked to heterogeneity of the carbon surface and represents a combined effect of the number of groups and their dissociation constants, and it might be affected by the confinement effect in carbon pores.

Based on the above, recent years resulted in the development of very efficient H_2_S no-alkaline-species-containing carbon adsorbents/reactive adsorbents/catalysts. An example is a work of Wu et al.^372^ who prepared N-doped sawdust derived activated carbon (NBAC) in one-step pyrolysis. They used supramolecular melamine cyanurate as a nitrogen source. Due to a very high nitrogen content of (19.6 at. %) and microchannel structure, the H_2_S removal capacity on this material reached 1872 mg g^–1^. While abundance of nitrogen provided active sites for H_2_S catalytic oxidation, the specific structure favored the mass transfer of reactants and the migration of elemental sulfur toward efficient pore filling. The mechanism is presented in Figure 11E.

### 4.2 Removal of Volatile Organic Compounds

#### 4.2.1 Removal of Formaldehyde

Porous carbons are known as excellent adsorbents of organic compounds, both in vapor or liquid form. This is generally attributed to a hydrophobicity of their surfaces and strong π–π interactions with benzene rings. However, many organic compounds, especially those negatively affecting human health and referred to as volatile organic compounds, VOCs, contain polar functional groups affecting surface interactions. This is especially important for small molecules such as formaldehyde and its derivatives, and smaller the molecule is, more important are the specific interactions. We have chosen to analyze here the effect of carbons chemistry on the removal of those species. It is owing not only to their surface chemistry but also to their low concentrations in air, rendering their removal challenging at ambient conditions and in the presence of humidity.

Formaldehyde, HCHO, is a VOC commonly used in industry for the production of a variety of household items and, owing to its high volatility, it is almost always present in indoor air. Its aldehyde groups directed the attention of researchers toward employing specific interactions targeting its removal.^382–384^ Usually, functional groups that can provide such interactions exist rather in larger pores than in ultramicropores and their engagement in physical or chemical adsorption processes can lead to the higher utilization of the carbon surface. Such groups can also contribute to an oxygen activation^232,365^ leading to HCHO oxidation to formic acid, which might increase the extent of an acid–base adsorption mechanism.^384,385^ In ultramicropores, dispersive interactions are expected to be a driving force for HCHO adsorption. The problem arises when moisture is present in air since water molecules compete with formaldehyde for an occupation of polar sites on the carbon surfaces.

Nitrogen groups are the most studied carbon surface entities that enhance HCHO adsorption.^382,383,385–389^ While pyridines and amines were reported as specifically attracting HCHO through polar interactions,^382,387,390^ pyrrolic nitrogen was indicated as most active to retain HCHO via hydrogen bonding and polar interactions.^386,388,391^ Yuan and co-workers^392^ considered pyrroles as consisting of Lewis base sites (doped nitrogen atoms with strong electronegativity) and Lewis acid sites (electron deficient intrinsic carbon atoms) and proposed that this specific pair is able to catalytically oxidize formaldehyde to CO_2_ and H_2_O with the help of active oxygen species. The source of the latter in this specific case was PtNP dispersed on the surface.

It was suggested that the amination of carbon surface increases HCHO adsorption due to a direct reaction of amines with HCHO, leading to the formation of imines.^385,393^ To increase the nature of specific interaction, the surface of carbons was also modified with aminosilanes.^394,395^ The carbonyl groups on HCHO not only reacted with amines but also with silica through cycloaddition onto a silicon group.^394^ Kang et al.^385^ proposed that a direct reaction of HCHO with various nitrogen-containing groups such as −NH_2_, −NH, −C=N, and −C–N can take place and amine groups can lead to amine–aldehyde conjugation through covalent bonding. Unglaube et al. reported that quaternary nitrogen was an important specific center for HCHO adsorption and their finding was supported by a factorial dimensionally reduction method.^396^

Some works also indicated the synergistic effects of the proximity of oxygen- and nitrogen-containing groups on formaldehyde retention on carbon surfaces. An example is the work of Lee et al.^400^ who indicated that close proximity of O and N groups in the carbon matrix favors effective hydrogen bonds with formaldehyde, or Ryu et al.^401^ who found the increased activity of amine groups when hydroxyl groups were in their vicinity. An interesting insight into HCHO adsorption on N-modified porous carbons was provided by Jiao et al.^397^ Their experiments and calculations revealed that nitrogen-doping did not affect the optimal pore size of 0.6 nm for formaldehyde adsorption. On the other hand, nitrogen-doping improved the nonbonding interactions between formaldehyde molecules and activated carbon. It was due to the changes in the electrostatic distribution on the surface that enhanced the adsorption of formaldehyde molecules (Figure 12A). The so-called nonbonding interaction between the formaldehyde molecules and activated carbon surface was found to decrease with an increasing pore size. Nevertheless, doping enhanced those nonbonding interactions, leading to an increase in limiting pore size for formaldehyde adsorption, which allowed multilayer HCHO adsorption, significantly increasing its removal efficiency. Electrostatic potential maps of HCHO interactions with perfect graphite, pyrrolic, pyridinic, and graphitic nitrogen were also investigated by Xiang et al.^398^ They also found that the highest interaction energy was on pyrrolic nitrogen, followed by graphitic N and pyridinic N (Figure 12B). Kowalska et al.^399^ by DFT calculations, reported that the coexistence of both hydroxyl and amine groups next to each other results in a nucleophilic addition to the carbonyl group, in which a new covalent C–N bond is formed (Figure 12C). In such a scenario the formaldehyde molecule, when approaching the surface, prefers to form a hydrogen bond with the hydroxyl group rather than with the amine one. Then, the hydroxyl hydrogen atom migrates to carbonyl oxygen of formaldehyde, which is accompanied by the simultaneous formation of the C–N bond and a proton transfer from amine to the hydroxyl group. The final product is the secondary amine *surface*–NH–CH_2_–OH (hemiaminal), which is more stable than the substrate.

**Figure 12:**
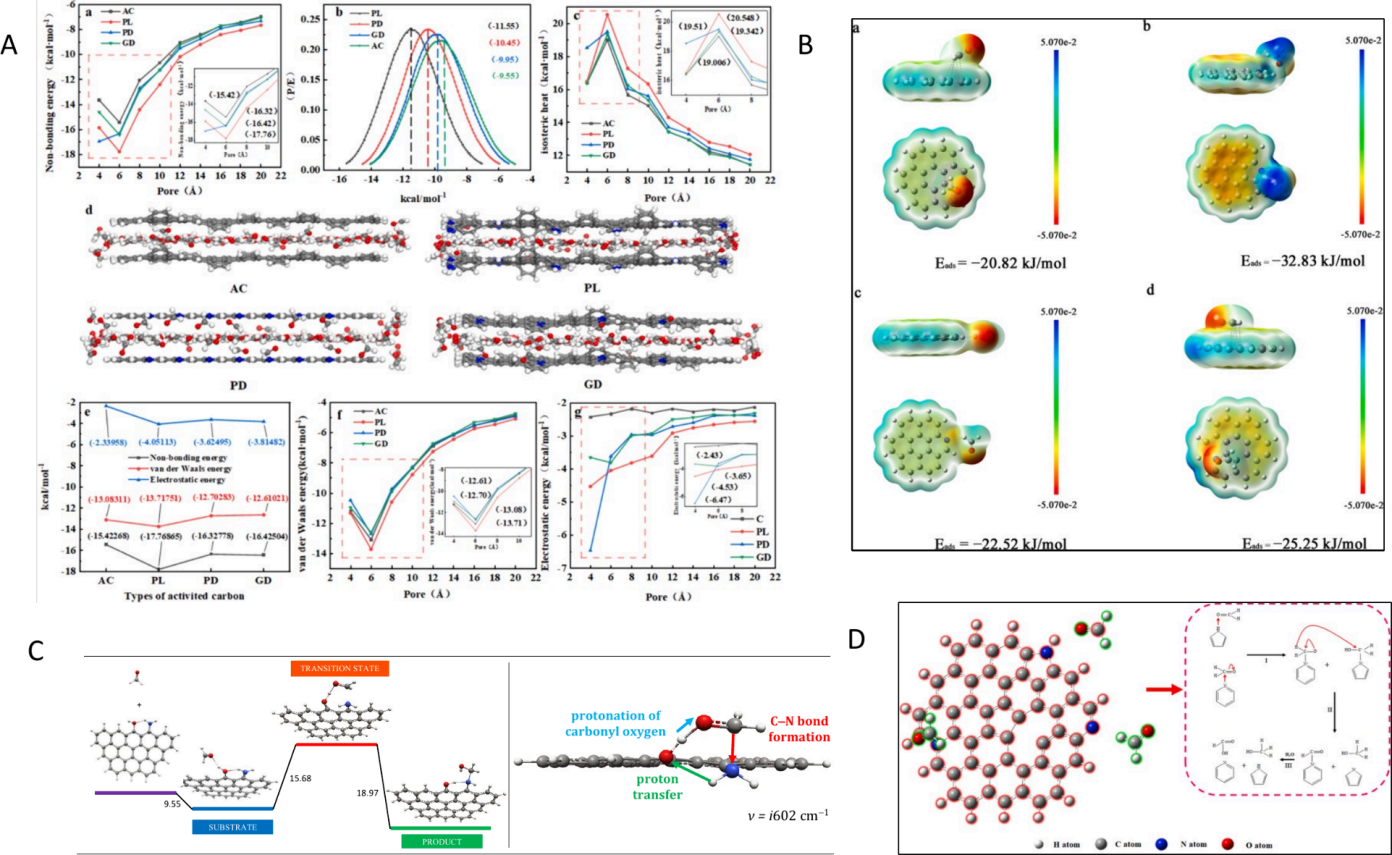
A) (a) Nonbonding energy of formaldehyde molecules by different pore sizes of nitrogen-doped and AC; (b) energy curves at 6 Å pore size; c, isosteric heat of adsorption of formaldehyde at different pore sizes; (d) Formaldehyde adsorption configuration diagrams at 6 Å pore sizes; (e) Splitting of formaldehyde nonbonding energies of nitrogen-doped and AC at 6 Å pore size; (f) van der Waals energy of the surface of the activated carbon on formaldehyde at different pore sizes; (g) Electrostatic energy of the surface of the activated carbon on formaldehyde at different pore sizes. Reproduced with permission from ref.^397^ Copyright 2024, Elsevier; B) Electrostatic potential maps of optimized HCHO physisorption configurations and corresponding HCHO physisorption energy over perfect graphite (a), pyrrolic N doped graphite (b), pyridinic N doped graphite (c) and graphitic N doped graphite (d). Reproduced with permission from ref.^398^ Copyright 2024, Elsevier; C) The transition state of the nucleophilic addition reaction (left) and the rearrangement of atoms is shown by arrows (right). Reproduced with permission from ref.^399^ Copyright 2023 Elsevier; D) Enhancement mechanism of HCHO removal over N-doped AC. Reproduced with permission from ref.^398^ Copyright 2023, Elsevier.

Important chemisorption interactions of HCHO with the pyridinic N atom promoting a Cannizzaro-type disproportionation reaction were indicated by Xiang et al.^398^ After adsorption of the HCHO molecule on the pyrrolic N and pyridinic N sites, the C atom of HCHO became more electrophilic due to the larger electronegativity of the O atoms. This process was hypothesized as leading to the lone electron pair of pyridinic N triggering the nucleophilic addition of HCHO. The authors suggested that after complex reaction steps, formamide and CH_3_OH were formed, and a pyrrolic N was left on the surface. Further reaction with adsorbed water would result in HCOOH and pyrrolic N formed (Figure 12D).

Since oxygen is always present on the carbon surface, oxygen-containing groups were also proposed as enhancing HCHO adsorption on carbons. Thus, phenols were reported as chemically active to immobilize HCHO via chemical reactions with the formation of hydroxybenzyl alcohol, which was retained on the surface.^383^ Among oxygen groups, carboxyl groups were indicated as having the highest adsorption energies (−14.5 kcal mol^–1^) and as being the most beneficial among the O-containing functional groups responsible for enhancing HCHO adsorption.^402^ It was attributed to forming hydrogen bonds with HCHO.^402,403^ Besides the specific type of the oxygen groups, the density of basic surface oxygen groups such as chromenes, pyrones, and quinones was also indicated as beneficial. These groups, in conjunction with the delocalized π-electrons in the basal planes of activated carbon, were suggested as responsible for HCHO adsorption.^404^

Besides nitrogen and oxygen, sulfur,^382,383,391,395,405^ phosphorus,^391^ and even chlorine^405^ incorporated to the carbon surface were reported as enhancing HCHO retention. Even though the reports on the effect of the last two are rather scarce, the experimental results of Su et al. suggested that the C–O–P­(O)­(OH)_2_ groups interact strongly with HCHO.^391^ Kang et al.^405^ indicated that C–Cl group increased HCHO adsorption via chemical reactions; however, the specific paths were not discussed. Sulfur incorporated to the carbons surface in various groups has also been reported as increasing HCHO removal. Thus, bulky sulfonic groups located in mesopores were suggested as reacting with formaldehyde forming hydroxymethanesulfonates and thiols reacted following the Mannich condensation reactions^383^ leading to the formation thiomethanols. Further studies of the role of sulfur were carried out by Kim et al.^395^ Su et al.^388^ indicated that the adsorption energies of HCHO with sulfur groups in so-called trioxidized configurations are stronger than those with nitrogen configurations (−0.527 eV vs −0.413 eV for pyrroles), leading to an increase in the HCHO removal efficiency. Strong interactions of HCHO with S–O incorporated to the carbon surface were also reported by Kang et al.^405^

The carbon surface can be even more prone to retain HCHO when both N- and S-containing groups are incorporated to the carbon matrix. Unglaube et al.^396^ reported HCHO removal on the series of two carbons modified with thiourea at temperatures between 400 and 950 °C. They found that HCHO adsorption was enhanced on low-temperature-modified carbons due to the abundance of amine groups. On the other hand, treatment at high temperatures, besides the conversion of amines to pyridines and to quaternary nitrogen, also resulted in the incorporation of sulfur to the carbon rings, leading to the very efficient adsorbents. The authors speculated that positively charged carbon atoms neighboring N and S heteroatoms attracted HCHO through specific interactions, which added to the dispersive forces-governing adsorption in ultramicropores, indicating the importance of a chemistry/specific porosity synergy.

#### 4.2.2 Removal of Other VOCs Indoor Air Pollutants/Formaldehyde Derivatives

Other indoor air pollutants creating health hazards are formaldehyde derivatives such as acrolein, acetaldehyde, and acetone. Their sources in indoor air are cigarette smoke and combustion products. They are also byproducts of cooking with heated oils. Acetaldehyde is a component of tobacco smoke and is naturally produced in fruits and as a fermentation byproduct. Acetone is a common solvent found in many products, and is also a byproduct of metabolism. As formaldehyde, they can also interact specifically with a functionalized carbon surface since in their molecules a carbonyl group is present and one or both hydrogen atoms are replaced by methyl or vinyl group(s). Experimental results on acrolein specific interactions/chemisorption with the carbons surface have not been reported. In the case of acetone adsorption, mainly physical interactions were evaluated.^406–411^ Chakrapani et al.^409^ concluded that a carbon nanotube curvature and topological defects impose acetone chemisorption. Amines were found as enhancing the adsorption of this molecule,^412^ and pyrrolic nitrogen along with oxygen groups were indicated as increasing the amount of acetone adsorbed on porous carbons.^406,407^

In the case acetaldehyde adsorption on porous carbons, its physisorption has been also the main focus of research. El-Sayed and Bandosz^413^ addressed its heat of adsorption at zero surface coverage, where adsorption is expected to take place only in the smallest pores. They found that *Q*_st_° on oxygen-rich carbons exceeded that one expected for physical adsorption (16 kcal mol^–1^ vs 12 kcal mol^–1^), suggesting chemisorption in large pores, where oxygen groups could exist. An increase in the amount adsorbed with an increase in the oxygen content was also reported. For N-modified carbon, a similar trend was found, which led to speculations that nitrogen groups are also active acetaldehyde adsorption centers.^209^ The positive effect of oxygen groups on acetaldehyde adsorption on carbide-derived carbons was also indicated by Wang et al.^414^

The adsorption of these formaldehyde derivatives on carbon surfaces has been also quite extensively studied from the theoretical points of view, where the specific focus of calculations led to the evaluations of the role of various isolated surface functionalities of the carbon surface. While on pure graphene surfaces dispersive interactions were found as the driving adsorption forces, regardless of the polarity of the molecule,^415,416^ the introduction of oxygen led to an increase in the amount adsorbed due to the strong contribution of hydrogen bonding, as indicated by Wang et al.^414^ who studied adsorption of acetaldehyde on carboxyl, anhydride and hydroxyl functionalized surfaces. Chen et al.^410^ studied the adsorption/desorption of acetone on the representative fragments of pristine graphene and its carboxyl, hydroxyl, ketone, and lactone-functionalized surfaces. The calculated adsorption energies increased in the following order: lactone group < carboxyl group < hydroxyl group < ketone group. Kowalska et al.^417^ studied acrolein, acetaldehyde, and acetone adsorption of functionalized graphene surfaces and found OH and NH_2_ groups as most likely to interact with carbonyl oxygen, resulting in the covalent bond formation. They reported that the efficiency of this process depends on steric constraints and on the charge on the carbon atom of the carbonyl group, which was affected/decreased by the presence of electron withdrawing moieties in the molecules.

### 4.3 Adsorption of Pollutants from Aqueous Solutions

In this section we focus on the role of carbon surface chemistry in adsorption of a new class of pollutants from water, PFAS and pharmaceutical compounds, aiming to rationalize the importance of the chemical characteristics of the porous carbon on their removal efficiency.

#### 4.3.1 Adsorption PFAS

Perfluoroalkyl and polyfluoroalkyl substances (PFAS) are a class of highly fluorinated aliphatic compounds. In their molecules, hydrogen atoms of a carbon chain are partially (polyfluoroalkyl acids) or entirely (perfluoroalkyl acids) replaced by fluorine atoms. They also contain a terminal functional group, which brings hydrophilicity (carboxylic, sulfonic, phosphonic, phosphinic, sulfonamide, ether carboxylic, or ether sulfonic) and makes them water-soluble. Owing to their hydrophilic–lipophilic character, they have been widely applied in stain repellents, carpets, food packaging, waterproof fabrics, cleaning products, pesticides, fire-fighting foams, cookware, and electrical wires coverings.^418^ They are characterized by a very high chemical and thermal stability and therefore, when released to the environment, they are considered as “forever chemicals”. This, along with their bioaccumulation and adverse effects on human health, creates an urgency to remove them from the environment. Adsorption is one of the methods widely employed for this purpose and porous carbons are considered as one of the best adsorbents. PFAS have low values of acid dissociation constants (p*K*_a_) and thus exist in the environment in their anionic forms (negatively charged). Based on the length of their C–F chains, they are classified into long and short chain PFAS. This property significantly affects their interactions with adsorbents’ surfaces.

In general, porous carbons are considered as good adsorbents of PFAS owing to their surface hydrophobicity and high pore volumes/surface areas. Nevertheless, extensive studies have demonstrated the importance of surface chemistry and this aspect increases in its weight with a decrease in the C–F chain of the PFAS molecules. Specific PFAS are usually referred to as 4–6 letter acronym addressing their chemical formulas. Owing to a broad variety of PFAS we refer the reader to the review papers addressing specific chemistry and properties of PFAS.^419–422^ Here, we focus mainly on the interactions of hydrophilic groups of PFAS, the most frequently studied in the literature.

Obviously, the hydrophobic character of carbon surfaces is their important asset to retain PFAS, as indicated by the results of Saeidi et al.,^423^ Park et al.,^424^ and Carter et al.^425^ Nevertheless, there are also reports stressing that PFAS adsorption capacity of porous carbons is strongly affected by electrostatic interactions. Saeidi et al.^426^ studied the removal of PFOA (perfluorooctanoic acid) and PFOS (perfluorooctylsulfonic acid) on commercial activated carbon felts of different surface features in terms of an anion exchange capacity (AEC), cation exchange capacity (CEC), the point of zero charge (PZC), and oxygen content. Adsorption parameters (*q*_m_ - maximum adsorption capacity and *K*_d_ - constant related to the adsorbate–surface affinity) were normalized to the surface area and their dependence on the specific descriptor of surface chemistry was analyzed (Figure 13A). AEC was found as positively influencing *q*_m_ and *K*_d_, which one could expect owing to the anionic forms of PFAS, and the larger adsorption capacities were found for carbons having lower CEC. At pH 7, carbons with PZC = 5.9 and 6.5 repelled the PFAS anions (electrostatic repulsion), due to a negative surface charge. On the other hand, carbons with PZC 7.1 and 7.3 were found as attracting the PFAS anions (electrostatic attraction), which resulted in an increase in the adsorption capacity. Since generally a high oxygen content of carbon reflects in a decrease in a hydrophobicity and an increase in a surface acidity, carbons with less oxygen adsorbed more PFOA and PFOS than did carbons with more oxygen (Figure 13A). This was reflected in low PZC and low AEC of high oxygen-containing carbons. These analyses led Saeidi et al.^426^ to state that in the case of PFOA and PFOS adsorption, the electrostatic interactions might overcome the hydrophobic ones and the specific combination of the hydrophobicity of PFAS and electrostatic interactions between PFAS anions and positively charged AC increased the adsorption performance. Studies of other porous carbons led to similar findings.^427–431^

**Figure 13:**
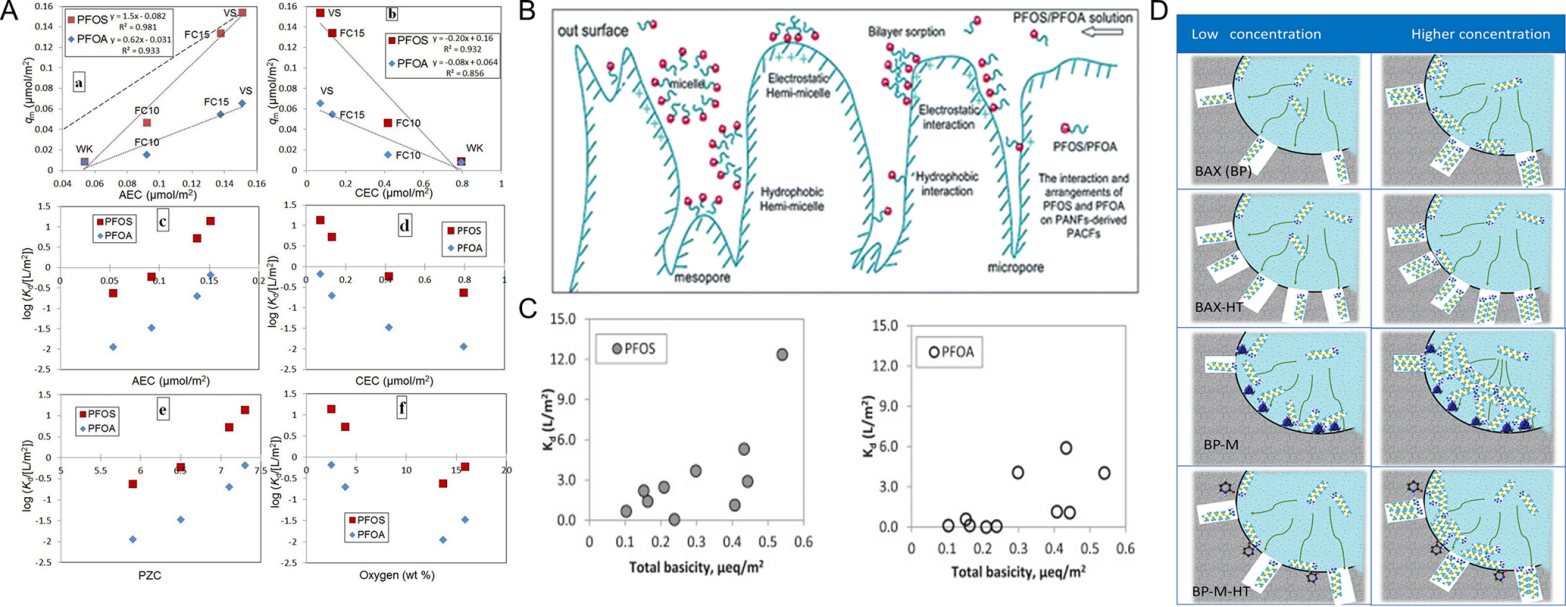
A) Correlation between chemical surface properties and adsorption parameters (*q*_m_ and *K*_d_). *K*_d_ was calculated for *C*_e,PFOA_ = 0.05 μmol L^–1^ and *C*_e,PFOS_ = 0.016 μmol L^–1^. The dashed line in (a) represents the hypothetical case of *q*_m_ = AEC. Reproduced with permission from ref.^426^ Copyright 2021, Elsevier; B) Schematic diagram for the sorption of PFOS and PFOA on the positively charged PANF-derived PACFs. Reproduced with permission from ref.^432^ Copyright 2017, The authors under CC-BY license; C) Correlation between adsorption distribution coefficient *K*_d_ (L m^–2^) and carbon surface basicity, PZC, or oxygen content for the ten carbonaceous adsorbents. Adapted with permission from ref.^430^ Copyright 2015, Elsevier; D) Mechanisms of PFOS adsorption on the carbon samples tested based on their surface features. The mechanisms on BP basically follow that on BAX, although the extent of adsorption is affected by the differences in the pore volumes. Reproduced with permission from ref.^433^ Copyright 2023, Elsevier.

The role of basicity in PFAS adsorption was stressed by Zhi and Liu^430^ who found a good performance for PFOA and PFOS adsorption on carbons rich in surface basic groups (Figure 13C). They analyzed a series of commercial carbons and suggested that oxygen-containing groups of pyrone and chromene, when protonated, and thus positively charged, might contribute to an enhancement in the performance. The possibility of dispersive interactions with “soft” π-electrons (Lewis base, electron pair donor) from a carbon graphene plane with “soft” organic carboxylic or sulfonic acids (Lewis acid, electron pair acceptor) was not excluded. The role of specific groups here can be considered as speculation since the acidity and basicity were only evaluated by treatment with HCl and NaOH, respectively. One tested carbon had a relatively high content of nitrogen (∼1 wt %), but this aspect of surface chemistry was not analyzed.

When analyzing PFAS adsorption on porous carbons, one has to consider their ability to form micelles at specific concentrations. These self-aggregated structures were found more easily formed when PFAS anions of high concentrations interacted with the positively charged surface of carbons.^434^ Deng et al.^435^ suggested that micelles and hemimicelles, when adsorbed at the entrances of micropores, could block the adsorption sites resulting in a decrease in the adsorption capacity. Chen et al.^432^ proposed a complex mechanism of PFAS adsorption involving electrostatic and hydrophobic surface interactions, a monolayer and bilayer micelles, and hemimicelles of PFAS on the surface of ACF (Figure 13B).

More detailed analysis of the mechanism of PFAS adsorption on porous carbons was presented by Du et al.^420^ They indicated that oxygen atoms from the terminal functional group of PFAS are capable to form hydrogen bonds with the functional groups −NH, −OH, and −COOH of a carbon adsorbent. Hydrogen bonding interactions with the surface OH groups were also indicated as important by Wang et al.^436^ and Deng et al.^434^ There are works which report the enhancement in PFAS adsorption on carbons through strong (−)­CAHB (negative charge-assisted hydrogen bonds) between the acidic groups of PFAS and the surface functional groups of similar p*K*_a_ values.^426,437^ Thus, Saeidi et al.^426^ found (−)­CAHB formation at pH 3 between the carboxyl groups of PFOA and the carbon surface. The analysis of ATR-FTIR spectra of carbons before and after PFAS adsorption by Zhang et al.^437^ suggested that the hydrogen bonds were formed between C=O oxygen and PFAS, and that ionized PFAS and the nonaromatic ketones, sulfides, and halogenated hydrocarbons formed (−)­CAHB on the surface at pH 4.5.

Besides the studies focusing on the effects of broad surface chemistry features addressed above, some specific modifications of carbons surfaces have been carried out to test their effects on PFAS adsorption. Zhi and Liu^438^ found that carbon treated under anhydrous ammonia at 700 °C showed better performance than that heat treated at 1000 °C. It was attributed to the basic character of the carbon surface. Even though a high temperature heating under an argon flow was expected to reduce the surface, the basicity remained practically unchanged and it could be due to the electron-rich character of the surface. The high-temperature treatment under ammonia atmosphere not only removed oxygen-containing functionalities but also introduced basic nitrogen-containing groups that are able to interact with PFAS anions. A heat treatment in H_2_ at 900 °C and modification of the surface with ethylenediamine were carried out by Saeidi et al.^423^ Interestingly, they found that the PFAS removal capacity on the heat-treated sample was higher than on that modified with ethylenediamine. It was explained by the efficient reduction of acidic groups in the former sample, advancing adsorption. On the other hand, the introduction of amines had a negative effect only on the concentration of carboxylic groups. That hypothesis was supported by a direct dependence of the measured adsorption capacities on the PZC values. On the sample treated at 900 °C, AEC was larger than on the amine treated one and CEC was smaller. Thus, the amine-modified sample still had a high density of groups repulsing PFAS.

A quite sophisticated treatment toward an introduction of nitrogen to the carbons surface and thus an increase in PFAS adsorption was conducted by Sun et al.^431^ They targeted quaternary nitrogen-grafted activated carbon. A series of samples was synthesized through HNO_3_ oxidation followed by a thermal treatment in an ammonia atmosphere and finally that sample was subjected to the Menschutkin reaction in which, in the presence of methyl iodide, quaternary ammonium salts were formed on the surface. Subsequent treatment steps resulted in an ∼100% increase in the adsorption capacity compared to that of untreated carbon. While oxidation slightly increased the total oxygen content from 6.05% to 7.70%, after the ammonia treatment, a marked increase in the nitrogen content was recorded (from 0.90% to 3.02%). The quaternization reaction, as expected, increased the contribution of NR_4_^+^ from 0.59% for ammonia treated carbon to 0.86%. An increase in PZC up to 9.61 confirmed an increase in surface basicity. At a groundwater pH of 7.1, the surface of final carbon was more positively charged than that of the carbon subjected to prior treatments, increasing the electrostatic attractions of the PFOA anions with the carbons surface. A treatment with quaternary ammonium species was also reported as increasing PFAS adsorption by Yuan et al.^427^ They used 3-chloro-2-hydroxylpropyltrimethylammonium chloride and 3-chloro-2-hydroxyl-propylcocoalkyldimethylammonium chloride to modify carbons and they found the formation of epoxides with hydroxyl and carboxyl groups on the carbon surface. Loading with the former resulted in more nitrogen on the surface than that with the latter one, leading to a more positive charge and thus an increase in PFAS adsorption.

Pauletto et al.^433^ modified porous carbons with amines via a thermal treatment with melamine. Surface chemistry of modified carbons was evaluated in details by XPS. On their carbons, PFOS was adsorbed by both hydrophobic interactions and specific interactions, including electrostatic attractions of negatively charged sulfonic groups with protonated amine groups or hydrogen bonding of sulfonic groups with surface polar oxygen groups. The authors indicated that the mechanism was pore-size dependent, and at a low PFOS concentration, a hydrophobic chain had a preference to be adsorbed in small pores, due to their strong adsorption potential and, with an increase in the concentration, adsorption via dispersive interactions in larger pores followed that one in ultra- and micropores (Figure 13D). The presence of functional groups on the larger pore surfaces caused that PFOS at low concentration was adsorbed simultaneously in micropores, vis dispersive forces, and on the functional groups in mesopores, through specific interactions. The latter led to a monolayer formation and thus to an increase in the efficiency of the physical adsorption process. Multilayer formation on the previously adsorbed PFOS molecules was a natural consequence of an increase in the PFAS concentration. Since the specific interaction centers usually exist on the mesopore surface, it was concluded that the presence of mesopores along with micropores is important to fully utilize the capability of the carbon surface.

Recently, Kim et al.^439^ studied the adsorption of a broad variety of long and short-chain PFAS containing various acidic groups on porous carbons modified by oxidation with H_2_O_2_, a high temperature ammonia treatment and heat treatment in an inert atmosphere. The surface chemistry was extensively characterized by XPS. The ammonia treated sample had the highest nitrogen content of 2.22% and it was in pyridinic-, pyrrolic-, graphitic-, and oxidized-N configurations. On the other hand, the oxidized sample had the highest density of C=O species. Boehm titration confirmed the presence of lactones and carboxylic groups. The authors determined a polarity index defined as (N+O)/C and it was the highest for the oxidized and nitrogen treated samples. All carbons were microporous and their porosity was only slightly affected by the modifications applied, compared to the initial sample. The collected results revealed that the surface chemistry had a prominent effect on the adsorption characteristics of the PFAS tested. The high polarity index was indicated as responsible for the strongest direct adsorbate–adsorbent interactions. Basic surface properties or a reduced polarity favored multilayer adsorption mechanisms, increasing the efficiency of the removal process. The authors also found that basic carbons or those of the lower polarity were excellent adsorbents of all PFAS tested, regardless the chain length and the electron affinity of the polar head, suggesting their most universal suitability for separations of PFAS of various configurations. The mechanisms of removal were diverse and they included hydrophobic interactions, electron donor–acceptor interactions, electrostatic interactions, and negative charge-assisted hydrogen bond, (−)­CAHB, formation.

Recently, due to a ban on long chain PFAS, researchers have turned their attention to short chain ones.^440^ The problem with their removal via adsorption is in their much higher hydrophilicity compared to their long-chain analogues, high mobility and an easiness of a replacement in complex matrices.^441^ In the case of these PFAS, the electron donating nature of acidic groups might play an important role and their interactions with the carbon surface may require the introduction of specific functional groups.

#### 4.3.2 Adsorption of Pharmaceutical Compounds

Pharmaceutical compounds and personal care products (PPCPs) have been considered water pollutants of increasing public concern for several decades now.^442^ Pushed by the development of analytical techniques with low detection limits and high sensibility, PPCPs have been detected at trace levels in natural aquatic environments across the world, with extensive data sets on in various water bodies (rivers, groundwater, wastewater) in the United States, many European countries, China and some other countries (although there is no data for over 50% of the countries in the world). PPCPs include a numerous variety of chemicals and their occurrence in water represents a high environmental risk due to their therapeutic and/or biological activity (even at trace levels), their long persistence in aquatic environments and their continuous introduction into waterways owing to the large volume discharged and the large amounts produced and consumed. The removal of PPCPs from water has become a largely explored topic in past decades, and among most investigated, adsorption processes on porous materials have proven to be effective, affordable, and compatible with other conventional technologies since they can be either implemented as an end-of-pipe technology or added to an existing facility.^443–449^ It is well-known that an adequate porosity (surface area and microporosity) is the main asset of activated carbons for a successful removal of most organic pollutants from water, including PPCPs. It is indeed a mature technology in water treatment approaches, and most drinking water facilities and wastewater treatment plants with so-called tertiary and quaternary treatments already incorporate adsorption on activated carbons to eliminate organic pollutants. Granular or powdered activated carbons with a high surface area and hydrophobic character are mostly applied.^443,450^ However, the large chemical variability of pharmaceutical compounds calls for a deep understanding of the performance–structure–composition correlation to optimize their adsorption performance in real water environments.

The adsorption of pollutants from a solution on porous carbons typically involves a complex interplay between specific and non-specific interactions at solution/pollutant/carbon interfaces. Such interplay depends on many factors including the porosity and composition of the carbon adsorbent, the properties of a pharmaceutical compound, and adsorption conditions (solution pH, temperature).^23,159^ Nonspecific dispersive and hydrophobic interactions (van der Waals, electron donor–acceptor) are predominant in hydrophobic nonionic pollutants, while polar or charged compounds can be retained through specific interactions (ion exchange, electrostatic) (Figure 14A,B). Dispersive interactions with the aromatic structure of the carbon materials are the dominant driving forces in the adsorption of aromatic pollutants, while functionalization with electron withdrawing groups (as acidic O-moieties) decreases the π-electron density, thereby weakening the dispersive interactions.

**Figure 14:**
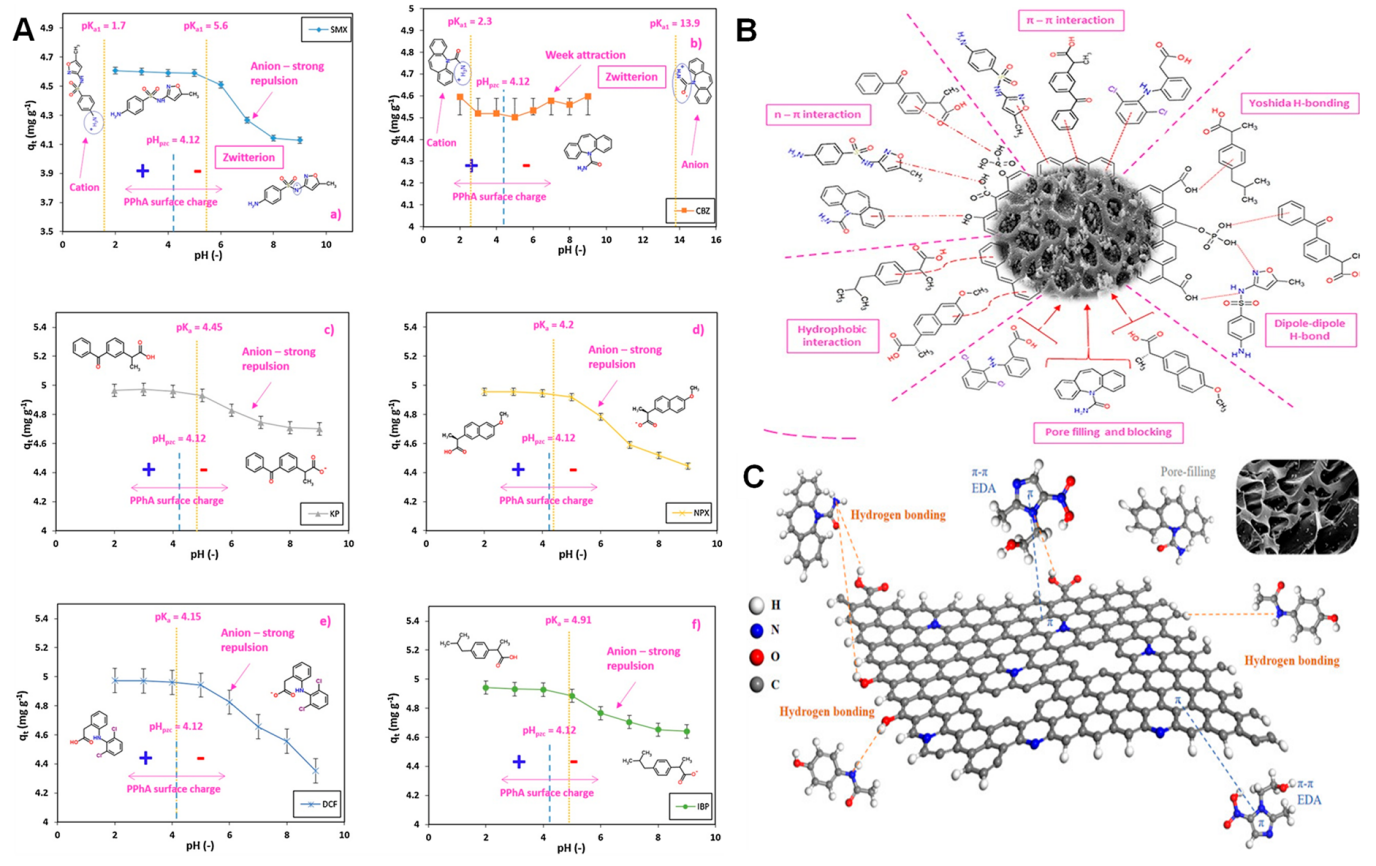
A) Effect of pH on the adsorption of various pharmaceuticals—sulfamethoxazole (SMX), carbamazepine (CBZ), ketoprofen (KP), naproxen (NPX), diclofenac (DCF), ibuprofen (IBF)—and B) schematic representation of the different mechanisms responsible for the adsorption of PPCPs on biomass-derived carbons. Reproduced with permission from ref.^451^ Copyright 2019, Elsevier; C) Representation of the adsorption mechanism on acetaminophen, carbamazepine and metronidazole on an O- and N-doped mesoporous carbon. Reproduced with permission from ref.^452^ Copyright 2024, the authors under CC BY License.

A recent study by Viegas et al.^453^ reports a deep analysis of the adsorptive removal of pharmaceuticals (carbamazepine, diclofenac, sulfamethoxazole) from real wastewater effluents on powdered activated carbons. To rationalize the removal efficiency of the different systems, the authors proposed a model based on 25 descriptors, covering the characteristics of the pharmaceuticals, the wastewater and the properties of the carbons (i.e., surface area, pore volumes, surface charge, pH, apparent density, particle size). Most suitable descriptors for describing the adsorption performance were found to be the solvation energy and octanol/water partition constant of the pharmaceuticals, and the surface area, micropore volume and density of activated carbons. The descriptors of the water matrix did not seem to be relevant, except for accounting for a decreased performance for competitive adsorption of an organic matter. Although the study covered activated carbons with rather similar surface chemistry (basic surface pH, positive surface charge, hydrophobic), the multivariate analysis revealed that the hydrophobic interactions between the pharmaceuticals and the carbon surface play a major role in the adsorption process on non-functionalized activated carbons.

On the other hand, the functionalization of activated carbons has been studied for years to modify their adsorptive properties, as they can promote specific interactions with surface moieties of a pollutant (Figure 14B). In these cases, the solution pH becomes a key operating factor since it affects both the ionization state of the pollutant (many pharmaceutical compounds are weak electrolytes) and the surface charge distribution in the activated carbon (due to the dissociation of the surface functionalities). Thus, it is essential to consider the hydrophobicity and p*K*_a_ of PPCPs when designing an effective adsorption process for removing of these contaminants from water systems.

In this sense, the uptake of PPCPs displaying carboxylic moieties (e.g., ibuprofen, naproxen, salicylic acid, clofibric acid, levofloxacin, penicillin, ...) on carbons is typically favored from their protonated form (solution pH < p*K*_a_), and when repulsive electrostatic interactions with the carbon surface are minimized (solution pH ≈ or < pH_PZC_ carbon). The adsorption is dominated by dispersive interactions in micropores, which commensurate the size with the molecular dimensions of the pollutant favoring a strong affinity to the carbon surface. Despite an adequate porosity, numerous examples in the literature report that oxidation of carbon typically leads to a sharp decrease in the uptake, discussing the dependence on the surface pH and the oxidation extent.^454–458^ The decreased uptake upon oxidation of carbon was attributed to the weakening of the dispersive interactions, the competitive adsorption of water as the carbon becomes hydrophilic, and the contributions of electrostatic repulsive interactions between charged moieties in the pollutant and the ionizable O-groups incorporated to the carbon upon oxidation.

Electrostic interaction forces an operate when the carbon surface is negatively charged (solution pH > pH_PZC_ carbon) and PPCP has charged moieties (anionic, cationic, zwitterions). An example is the adsorption of zwitterionic PPCPs containing both an acidic and a basic group (e.g., tetracycline, ciprofloxacin, β-lactam antibiotics, amino acids). In those cases, both ionic and nonionic exchange interactions may contribute to adsorption, as is the case with sulfamethoxazole and carbamazepine, both presenting zwitterionic forms.^459–461^ As an example, the adsorption of lysine on hydrophobic carbons is favored at pH 10, where the pollutant is primarily in the zwitterionic state.^461^ The cationic (pH 1–8) and anionic forms (pH above 10) displayed significantly lower adsorption capacities (almost an order of magnitude lower). The adsorption and separation performance significantly increased upon oxidation of the activated carbon and the incorporation of O-groups that could be deprotonated (upon the pH), owing to electrostatic interactions with positively charged amino groups of the amino acid. The authors also reported a correlation of the adsorption and separation factors of several pollutants with the accumulated amounts of CO_2_ and CO (normalized by specific surface area) of different adsorbents, characterized by TPD-MS.

For PPCPs displaying hydroxyl moieties, the molecular form is predominant in a solution unless very basic solution pH conditions are applied (p*K*_a_ of hydroxyl moieties usually ranges between ca. 8–9), thus their adsorption in most cases is due to dispersive interactions from the neutral form and/or H-bonding (common pH values of waters and wastewater are in the range 6–8, as most countries apply restrictions to the discharge pH of industrial effluents). Through the characterization of porous carbons before and after the adsorption, Sekulic et al.^451^ reported the occurrence of specific interactions between O- and P-groups in the carbon adsorbent and several PPCPs (sulfamethoxazole, carbamazepine, ketoprofen, naproxen, diclofenac, and ibuprofen). The authors associated FTIR shifts after adsorption to H-bonding interactions between the carboxylic and hydroxyl moieties of the porous carbon and the aromatic rings of various PPCPs, and dipole–dipole H-bonding between the hydroxyl groups of carbon and the nitrogen atoms of sulfamethoxazole, carbamazepine, and diclofenac. The authors also suggested the occurrence of donor/acceptor interactions between carbonyl and P-groups—present in carbons prepared by phosphoric acid activation—acting as donors, and the aromatic ring of the PPCPs (acting as acceptor).^462^

A special case is the reactive adsorption of penicillin ((2S,5R,6R)-3,3-dimethyl-7-oxo-6-[(2-phenylacetyl)­amino]-4-thia-1-azabicyclo[3.2.0]­heptane-2-carboxylic acid, p*K*_a_ 2.74)^463^ in acidic carbons. It has been reported that acidic porous carbons catalyze the degradation of this antibiotic into various intermediates (penillic and penilloic acid derivatives), in a process similar to its hydrolysis in strong acidic solutions. The acidic surface chemistry of carbons accelerated the degradation of the adsorbed penicillin molecules, owing to a strong local pH decrease inside the pores. The degradation also occurred in activated carbons of basic pH, but at a much lower extent and with different speciation of intermediates. Reactive adsorption on porous carbons has also been reported for carbamazepine and sulfamethoxazole, and attributed to the ability of carbon surfaces decorated with oxygen groups to activate oxygen and form superoxide ions that react with the pollutants inside the pores.^464^ The authors described that these oxygen-groups should exist in large micropores and mesopores where typically the affinity for the pollutant is typically weak. Thus the reactive adsorption mechanism enhances the ability of activated carbons to remove these pharmaceutical compounds.

Sulfur functionalization of carbons has been reported to have a different effect depending on the nature of the PPCPs. As an example, Vidal et al. observed that S-modified carbons exhibited higher uptake of trimethoprim than with no sulfur incorporated in the carbon matrix,^465^ and explained this behavior to acid–base interactions between the sulfur species present in large pores (sulfoxide, sulfones, sulfonic groups) and the amine and pyridinic nitrogen of trimethoprim. On the other hand, the adsorption capacity of sulfamethoxazole and diclofenac (polar pollutants) decreased on those S-doped carbons, owing to a decrease in the polarity of the carbon surfaces after the modification. The effect of the functionalization on the retention of pharmaceutical compounds on porous carbons was addressed in a recent study of Serna-Carrizales et al.^466^ They explored the adsorption of various sulfonamide compounds—sulfamethoxazole, sulfadiazine, and sulfametazine—and found that the affinity of activated carbon for sulfamethoxazole, followed by sulfadiazine and sulfametazine increased with an increased hydrophobicity of the pollutants, owing to hydrogen bonding and π–π interactions.^466^

Barczak et al. reported the N-functionalization of carbon textiles with favorable impact of pyridone groups on the removal of diclofenac. The authors attributed the improved retention of the pharmaceutical to the combined role of dispersive and electrostatic interactions, highlighting the indirect effect of deprotonated functional groups. Through theoretical studies, the authors demonstrate the favorable interactions of diclofenac on carbon surfaces functionalized with hydroxyl-based arrangements (particularly adjacent functional groups) and protonated amines functionalities.^458^

Co-doping with various heteroatoms has also been explored for improving the adsorption of PPCPs.^467–469^ While most of these studies attribute the differences in the uptakes to the presence of those heteroatoms, very often the differences in the (micro)­porosity of doped carbons are not discussed in sufficient detail. This makes it difficult to draw clear conclusions about a global adsorption mechanism and the impact of the heteroatoms. This should deserve better attention. As an example, Chu et al. reported the adsorption of acetaminophen, carbamazepine, and metronidazole on O- and N-doped mesoporous carbons.^452^ The study demonstrated the increased retention of the pollutants through the formation of the hydrogen bonds between carbonyl and hydroxyl groups on the carbon material and hydroxyl/amine and carbonyl moieties (respectively) on the pharmaceutical compound (Figure 14C). The presence of pyrrolic nitrogen favored acid–base interactions with Lewis sites in the pharmaceuticals. Similarly, N-doped carbons prepared from the carbonization of a polyaniline at various temperatures displayed improved metronidazole retention in carbon with graphitic nitrogen groups, due to favored π–π electron acceptor–donor interactions.^469^

Camparotto et al. prepared mesoporous carbons doped with B- and N-groups for the adsorption of diclofenac and paracetamol.^467^ The authors described an important effect of the dopants, with higher adsorption capacities of B-doped carbon, compared to N-doped and the pristine material, and attributed this to the interactions of B-sites (Lewis acid sites) with the electronic density of the pollutants. Unfortunately, the differences in the (micro)­porosity of these carbons were not sufficiently discussed in their study.

Krstić et al.^468^ described the preparation of nitrogen and sulfur doped carbon cryogels. The authors reported an improvement in the adsorption of various pharmaceuticals (carbamazepine, naproxen, diazepam, diclofenac) on codoped carbons with high concentrations of nitrogen and sulfur. Their carbons displayed a very different porosity (surface areas were 780 and 1530 m^2^ g^–1^, for undoped and doped carbons, respectively) and similar heteroatoms contents. Hence, the increased uptakes on the doped carbons might be related to those differences in the porosity, with a minor impact of the surface functionalization.

In this context, theoretical studies are a useful tool to explore the adsorption behavior and mechanisms of PPCS on functionalized carbon surfaces, with a focus on both the chemistry of the pollutant and the surface chemistry and the changes in the electronic cloud density of carbon materials upon functionalization. Molecular dynamic studies on carbon surface models have shown that antibiotic molecules are most likely to be adsorbed at a distance of 4–5 Å from the aromatic carbon surface,^466^ and pharmaceutical molecules preferentially adsorb either on the curved surface (pores) of the carbon or at oxygenated-sites.^470^ N atoms in pyrimidine and aniline rings (e.g., sulfametazine) are stronger electron-donating groups than are O- and S-atoms in the heterocycles of the pollutant (e.g., sulfamethoxazole, sulfadiazine), resulting in an enhanced adsorption through π–π interactions. On the other hand, methyl substituents are the weak activators of an aromatic ring, allowing for less active H-bonding interactions.^466,471^ Regarding the surface chemistry of carbons, theoretical studies have demonstrated that oxygen functionalization can improve the uptake by increasing the strength of specific interactions. Specifically, oxygen of pyran and ketone groups can participate in H-bonding with the specific moieties of the pollutant (e.g., amines, amides) and these interactions are stronger than dispersive π–π stacking.

A parametrized theoretical study on the adsorption of various PPCPs on activated carbons with varied oxygen contents (from 3 to 10 wt %) described statistical correlations between the adsorption of the drugs and two characteristics of the carbons: the functionalization grade (oxygen content) and the void space (surface area).^470^ According to this correlation, the authors discriminated a dominating mechanism in the adsorption of the studied pharmaceuticals, and described the adsorption as more or less prone to change with the surface area and/or oxygen content.

The surface functionalization of porous carbon also affects the adsorption of PPCPs in competitive environments owing to the differences in surface species and the speciation of the pollutants in mixtures.^456,472–475^ Most studies focus on describing the competitive adsorption of different mixtures in terms of the carbon porosity, paying less attention to the role of their surface chemistry (mainly because unfunctionalized carbons are generally better PPCPs adsorbents). Mansouri et al.^456^ investigated the competitive adsorption of amoxicillin and ibuprofen at various molar ratios in the binary mixtures on different carbons, and observed that the perturbing effect of the second component in the mixtures was more significant for amoxicillin (that showed a lower affinity than ibuprofen for the studied carbons) and for the oxidized carbons. Nielsen et al.^474^ described the importance of matching the surface chemistry of the adsorbent to favor the simultaneous removal of pollutants of varied characteristics. For instance, the uptake of polar compounds (sulfametoxazole and trimetroprim) can be improved by specific interactions (e.g., chelation, acid–base reactions), whereas the least polar pollutants (carbamazepine) are better retained in hydrophobic sites.

### 4.4 Catalytic Processes

#### 4.4.1 Conventional Catalysis

This section focuses on liquid and gas-phase reactions catalyzed by metal-free carbons that have the potential to be used in industrial applications. Porous carbons generally offer sustainable, low-cost alternatives to metal catalysts. They are highly stable and can easily be tailored to produce targeted surface chemistries, including heteroatom doping, as discussed in Sections 2 and 3. Additionally, there is no risk of metal leaching in liquid reactions. The use of carbon catalysts for electrochemical reactions (ORR, OER, HER, CO_2_RR, etc.), one of the most relevant topics in recent years, is not addressed here, as a full section is dedicated to these reactions (Section 4.4.2); the same applies to photocatalytic applications (Section 4.4.3). Numerous reviews have examined carbon materials as metal-free catalysts. The earliest dates back to the 1960s,^476^ followed by several influential surveys in later decades,^206,354,477–479^ with some more recent updates on this field.^480–483^ Our aim here is to highlight studies in which the catalytic activity can be clearly linked to a specific surface site, thereby supporting a detailed reaction mechanism. However, meaningful correlations require a careful experimental control, namely: (i) Reactions must be run in a true chemical-kinetic regime, free of mass-transfer limitations. This means eliminating external diffusion limitations through adequate stirring (in batch or continuous stirred tank reactors) or high linear velocities (in plug-flow reactors) and suppressing internal diffusion by using suitably small catalyst particles. (ii) Catalyst series should differ in only one targeted parameter (typically the concentration of a particular surface group), while textural parameters (surface area, pore size distribution) and all other surface functionalities should remain as constant as possible. Changes in textural properties can mask a real chemical effect. (iii) Deactivation must be completely prevented; otherwise, kinetic data must be extracted from a deactivation-free window (initial-rate measurements are often the simplest option). (iv) Inorganic residues with a potential catalytic activity must be eliminated from the carbon samples, for example, by acid washing with HCl, or by using metal-free synthesis conditions, as is the case with polymer-based carbons.

Table 1 summarizes the most widely studied reactions with metal-free carbon catalysts and identifies their proposed active surface sites. Selected examples for environmentally related reactions are discussed in greater detail below. Although oxidative dehydrogenation (ODH) is only tangentially considered an environmental reaction, it is included here because it is the most thoroughly studied carbon-catalyzed transformation; the mechanistic insights gained from ODH have been pivotal for identifying carbon active sites and for demonstrating how metal-free catalysts can enable lower-temperature and more energy-efficient processes. Acetylene hydrochlorination was included as a prime example of the potential of metal-free carbon catalysts to replace hazardous catalysts with a strong environmental impact, such as HgCl_2_ catalysts. Gas phase reactions for the removal of pollutants (NO_*x*_, SO_2_, and H_2_S) were already discussed in Section 4.1. Advanced oxidation processes targeting the removal of organic pollutants were chosen to highlight the catalytic effectiveness of carbon materials in water and wastewater treatments.

**Table 1:** Most Studied Reactions Using Metal-Free Carbon Catalysts and the Proposed Active Surface Sites

Reaction (Catalytic Transformation)	Active Site(s) on Carbon	Ref.
**Gas phase**		
Oxidative dehydrogenation	Carbonyl/quinone (C=O) groups	^342,484–493^
Dehydration of alcohols	Carboxylic acids	^494,495^
	Brønsted-acid phosphorus species (P–OH + C–O–P)	^496–499^
Dehydrogenation of alcohols	Lewis acids and basic sites	^494,495^
NOx reduction (SCR with NH_3_)	Acidic surface oxides (carboxylic, lactone and phenols) + basic sites (carbonyls or N5, N6)	^338,339,347^
	Carbonyl/quinone (C=O) groups	^340^
	Hydroxyl and epoxy species	^343^
	C3–P=O structures + Brønsted acid sites (mainly C–OH and P–OH species)	^349^
	Lewis acid sites (BC3, BC2O, and BCO2)	^348^
NO oxidation	Basic sites (N-containing groups)	^350–352^
SO_2_ oxidation	Basic sites, pyridinic—N6	^207^
	Carbonyl/quinone (C=O) groups	^355^
	Ester or epoxy groups	^356,357^
H_2_S oxidation	Basic sites, pyridinic—N6	^374,500–503^
Acetylene hydrochlorination	Doped N species and intrinsic defective sites	^504,505^
	High-curvature defective carbon	^506^
**Liquid phase**		
Catalytic wet peroxide oxidation	Basic sites (basic oxygen groups or graphitic π-sites)	^507–509^
Catalytic ozonation	Basic sites (basic oxygen groups or graphitic π-sites)	^510–512^
	Basic N groups (N5 + N6)	^513^
Catalytic wet air oxidation	Basic sites (basic oxygen groups or graphitic π-sites)	^514^
	Basic N groups (N6)	^515^
Esterification/transesterification of organic acids	–SO_3_H groups	^516–521^
	Carboxylic acids	^522^
Selective oxidation of alcohols (including HMF)	Pyridinic—N6	^523^
	Graphitic N	^524–528^
	Coexistence of P=O, P–OH functionalities along with the graphitic regions	^80^
	Graphitic N and C3PO species	^529^
Hydrolysis of Cellulose	SO_3_H, COOH, and OH Groups	^530^
	Brønsted acids (P–OH) + thermally stable oxygen functionalities (phenol, carbonyl, and lactone)	^531^
Selective hydrogenation of *o*-chloronitrobenzene	Turbostratic carbon (unsaturated carbon atoms on zigzag edges)	^532^
Selective oxidation of ketones	Carbonyl/quinone (C=O) groups	^533^
Diesel fuel desulfurization (Oxidative desulfurization)	Carbonyl/quinone (C=O) groups	^358,534–536^
	Lactones and carboxyl groups	^537^
	B–C, B–O, and BCO_2_	^538^
Knoevenagel condensation	Pyridinic—N6	^539^

##### 4.4.1.1 Oxidative Dehydrogenation of Hydrocarbons

The oxidative dehydrogenation (ODH) of hydrocarbons is one of the most studied carbon-catalyzed reactions. The conversion of ethylbenzene (EB) to styrene was the first ODH reaction to draw the scientific community’s attention. Activated carbons can catalyze this reaction at moderate temperatures (300 to 400 °C), achieving a performance comparable to that of some metal oxides but working at lower temperatures. Following an evidence that polymers such as polynaphthoquinone could be active,^486^ Alkhazov et al. were the first to use activated carbon,^540^ and subsequent works established that surface carbonyl/quinone groups are the active sites for ODH.^486,487^ Pereira et al. prepared a series of oxidized carbons with different concentrations of carbonyl/quinone groups while maintaining textural properties and found a linear correlation between a styrene formation rate (at 350 °C) and the concentration of surface C=O groups.^488^ Specifically, the activity (*a* in μmol·g^–1^·s^–1^) increased roughly linearly with a quinone density ([*Q*] in μmol·g^–1^), yielding a relationship *a* = 3.87 × 10^–4^·[*Q*] + 0.71. This quantitative correlation was among the first clear evidence linking the catalytic activity to a specific carbon surface functional group. Later, Su et al. presented an elegant approach to identify and quantify active sites using a chemical titration.^342^ They proposed a novel method of a chemical titration to quantify the three main types of oxygen functional groups (carbonyl, phenol, and carboxylic acid) on CNTs (Figure 15A). In this method, phenyl hydrazine reacts selectively with carbonyl groups. Catalytic tests revealed that the activity declined only when oxidized CNTs were passivated with phenyl hydrazine, demonstrating that ketonic carbonyls are the dominant active sites. The remaining activity matched that of a control sample annealed in argon at 1300 °C, where all oxygen groups had been removed, leaving only structural defects. Since phenyl hydrazine has a similar size and polarity to reactant EB, this reaction allows one to quantify the number of active sites accessible to EB. This method was shown to be equivalent to or more accurate than traditional TPD or XPS methods. The researchers observed a linear dependence of the ODH activity on titrant consumption (phenyl hydrazine) (Figure 15B), which clearly indicates that the surface ketonic carbonyl groups are the active sites. The turnover frequency (TOF), obtained from the slope of the fitting, was 4.10 × 10^–4^ s^–1^, which is of the same order of magnitude as that obtained by Pereira et al. for activated carbons using TPD to quantify the carbonyl groups.^488^ A later study showed that an *in situ* titration approach during the reaction also worked and produced similar results.^341^ The researchers tested five different carbon materials (carbon nanotubes, graphene oxides, and onion-like carbons) and observed similar TOF values for all of them, comparable to those obtained using an *ex situ* method. This suggests that the chemical nature of the active sites is identical for all materials and that the microscopic structure only minimally influences their intrinsic reactivity.

**Figure 15:**
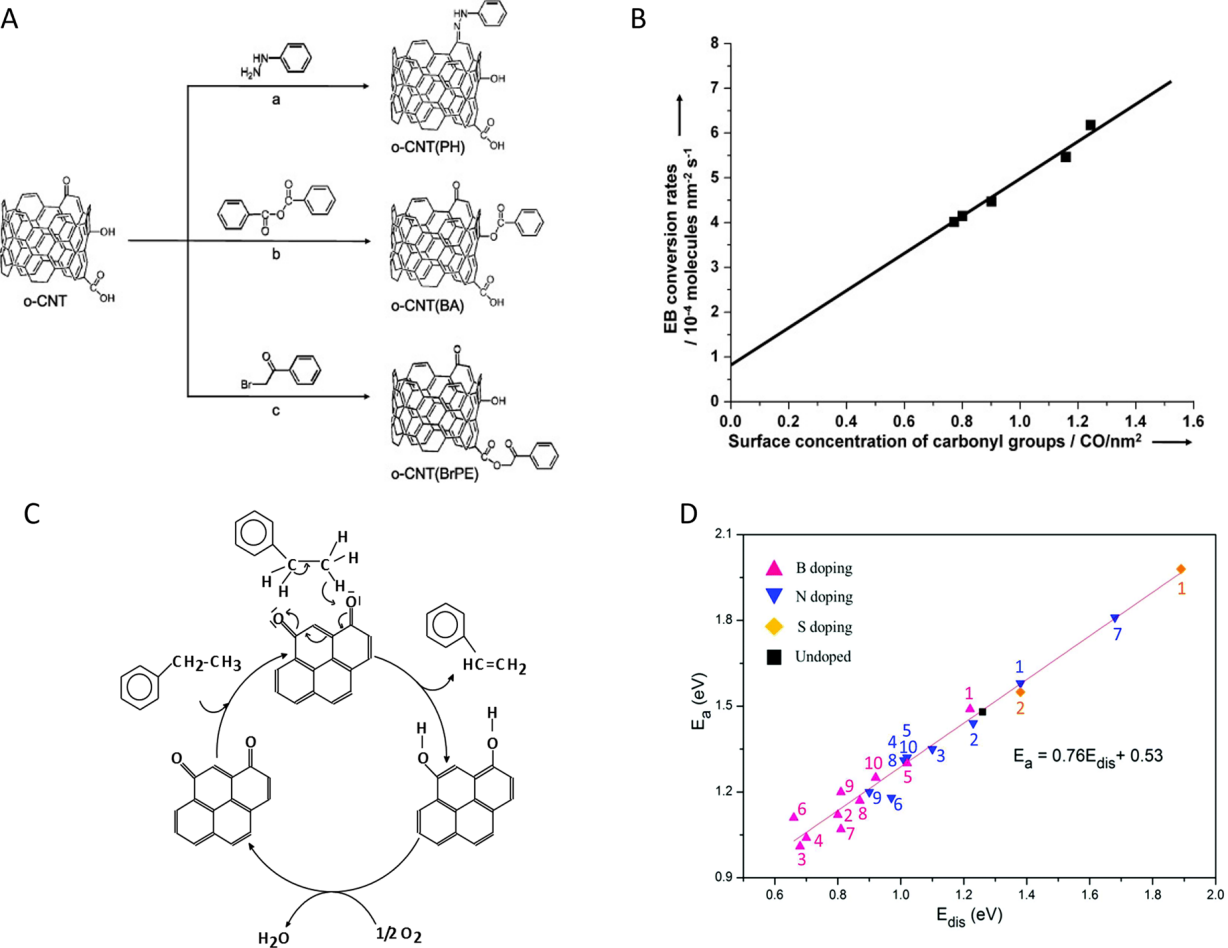
A) The titration processes for a) ketonic carbonyl with phenylhydrazine, b) phenol with benzoic anhydride, and c) carboxylic acid groups with 2-bromo-1-phenylethanone on CNTs. Reproduced with permission from ref.^342^ Copyright 2014, Wiley; B) EB ODH conversion rates on o-CNTs with different surface concentrations of carbonyl groups. Reproduced with permission from ref.^342^ Copyright 2014, Wiley; C) Catalytic cycle involved in the ODH of ethylbenzene with carbon catalysts. Adapted with permission from ref.^541^ Copyright 1983, Elsevier; D) Relationship between the barrier (*E*_a_) for the first C–H bond breaking in ethane and the dissociation energy (*E*_dis_) on the undoped, boron (B)-, nitrogen (N)-, and sulfur (S)-doped catalysts. Reproduced with permission from ref.^542^ Copyright 2015, The Royal Society of Chemistry.

One of the main constraints of using activated carbons for the ODH of ethylbenzene is their instability due to coke deposition and deep oxidation, which leads to the formation of CO_2_ as a side reaction. Additionally, oxygen at relatively high temperatures can oxidize the catalyst, resulting to its consumption.^543^ To overcome these drawbacks, Schlögl and co-workers were the first to report the use of nanocarbons (carbon nanotubes, carbon nanofibers and onion-like carbons), which were more stable toward oxidation and presented less carbon formation.^489–491,544^

Following the pioneering studies on the ODH of ethylbenzene, a substantial body of research has appeared on the ODH of light alkanes to olefins over metal-free carbon catalysts, where coke formation is less severe (a recent review that also covers boron-based catalysts is available in ref.^545^). The earliest contributions addressed the ODH of propane over CNTs,^492^ showing that surface phosphoric oxides passivate the carbon surface and suppress gasification. This finding was later confirmed in the ODH of butane by Su and co-workers^493^ in the ODH of isobutane over carbon xerogels,^484^ and in the ODH of 1-butene to butadiene over CNTs.^485^ The crystallinity of the material was also proved to be a critical parameter for avoiding combustion, as evidenced by the superior performance of CNTs compared to that of activated carbons.^485^ The active sites responsible for the ODH of light alkanes are almost invariably assigned to surface quinone groups. Notably, the TOF measured for the ODH of isobutane on carbon xerogels, 3.17 × 10^–4^ s^–1^,^484^ is of the same order of magnitude as the TOF values reported for ethylbenzene ODH. In the same study, the additional presence of carboxylic anhydrides lowered the TOF. This was attributed to the electrophilic character of these groups, which decreases the electron density at the active sites and thus reduces the catalytic activity.

Figure 15C illustrates the accepted ODH mechanism on carbon catalysts, which relies on the redox cycling of surface quinone/hydroquinone pairs. In this scheme, for ethylbenzene ODH, surface quinone extracts a hydrogen atom from the alkyl group, yielding the corresponding alkenyl and reducing the site to a hydroquinone group. Gas-phase O_2_ then reoxidizes the hydroquinone, regenerating the quinone and releasing H_2_O. Su and co-workers provided a relevant review about this mechanism, supported by microcalorimetry, ambient-pressure XPS, and *in situ* chemical titration techniques.^546^ An additional evidence came from DFT calculations^547^ and catalytic tests with a phenanthrenequinone macrocyclic trimer containing only quinone moieties,^548^ both of which confirm that diketone-like (quinone) groups are the true active sites. Using graphene nanoribbon models decorated with edge oxygen functionalities, Schwarz and co-workers showed by DFT that dicarbonyls on zigzag edges and quinones on armchair edges provide the optimum balance for the high ODH activity.^549^

To study the effect of heteroatom doping, Su and collaborators used first-principles calculations to examine the activation of the first C–H bond in ethane on carbon catalysts doped with boron, nitrogen, and sulfur.^542^ Their results indicated that B and N doping could enhance the activity of carbon catalysts, whereas S-doping tended to suppress it (Figure 15D). The different performance of all three dopants was attributed to the energy level of the acceptors after doping, which influences the electron transfer between the reactant and catalyst. Experimentally, using nitrogen-doped CNTs as catalysts for the ODH of propane showed high reaction rates and a large selectivity to propene, in a positive correlation with the graphitic-nitrogen content.^550^ In nitrogen-doped carbon xerogels, Pelech et al. likewise reported that the catalytic activity increased with the surface nitrogen content.^484^ For boron-doped carbon nanofibers (CNFs), an optimal activity was found at ≈2% B,^551^ while phosphorus-doped CNFs delivered the highest selectivity at iso-conversion among all catalysts studied.

In summary, the most promising carbon-based catalysts for the ODH reaction should: (i) possess a highly graphitic framework to resist oxidation, an effect that surface phosphoric groups can also reinforce; (ii) contain a high density of quinone functionalities that serve as the active sites; (iii) be doped with the optimal amounts of boron, followed in priority by nitrogen; and (iv) be essentially free of sulfur and strongly electron-withdrawing oxygen groups, such as carboxylic anhydrides.

##### 4.4.1.2 Acetylene Hydrochlorination

Acetylene hydrochlorination (C_2_H_2_ + HCl → CH_2_=CHCl) has become one of the most notable success stories of metal-free carbon catalysis over the past decade. The reaction is central to a coal-based PVC production and offers a sustainable alternative to the traditional, highly toxic HgCl_2_ catalysts. Over the years, the activity and stability of carbon catalysts have improved so significantly that they now rival, and in many cases surpass, the metal catalysts, making a large-scale deployment a real possibility. A recent comprehensive review^552^ summarizes these developments in detail.

The breakthrough came from Wei and co-workers,^553^ who showed that nitrogen-doped carbon nanotubes (N-CNTs) exhibit a clear linear relationship between an acetylene conversion and the quaternary N content. This discovery triggered an extensive search for optimal N-doped carbons derived from various precursors, including graphene, MOFs, biomass, and synthetic polymers. Subsequently, single- and codoping with other heteroatoms was explored. Boron-doped carbons,^554^ P/N-codoped materials^555^ and S-doped carbons^556^ delivered notable activity enhancements. Attention then shifted to the role of intrinsic defects. Li’s group^557^ systematically screened hundreds of carbon samples and obtained a volcano-type dependence of the acetylene conversion on the sp^3^/sp^2^ carbon ratio, with an optimum around 32–35%.^558^ This finding underscored the decisive influence of defect structures on this catalytic process.

More recently, the synergistic interplay between heteroatom doping and defect engineering has been demonstrated, yielding record activities for N-doped defective carbons.^504,505^ This enhanced performance is attributed to the increased adsorption capacity for C_2_H_2_ and HCl that occurs when both the density of defects and the content of nitrogen increase. Finally, a carbon curvature emerged as a powerful design parameter. High-curvature defective carbon (HCDC) outperformed the benchmark 0.25% Au/activated-carbon catalyst and exhibited an acetylene uptake nearly 2 orders of magnitude greater than that of planar defective carbons.^506^ Figure 16 summarizes this decade-long evolution, from early N-doped catalysts to today’s high-curvature defective carbons, and tracks the concurrent rise in space–time yield (STY) of vinyl chloride.^552^

**Figure 16:**
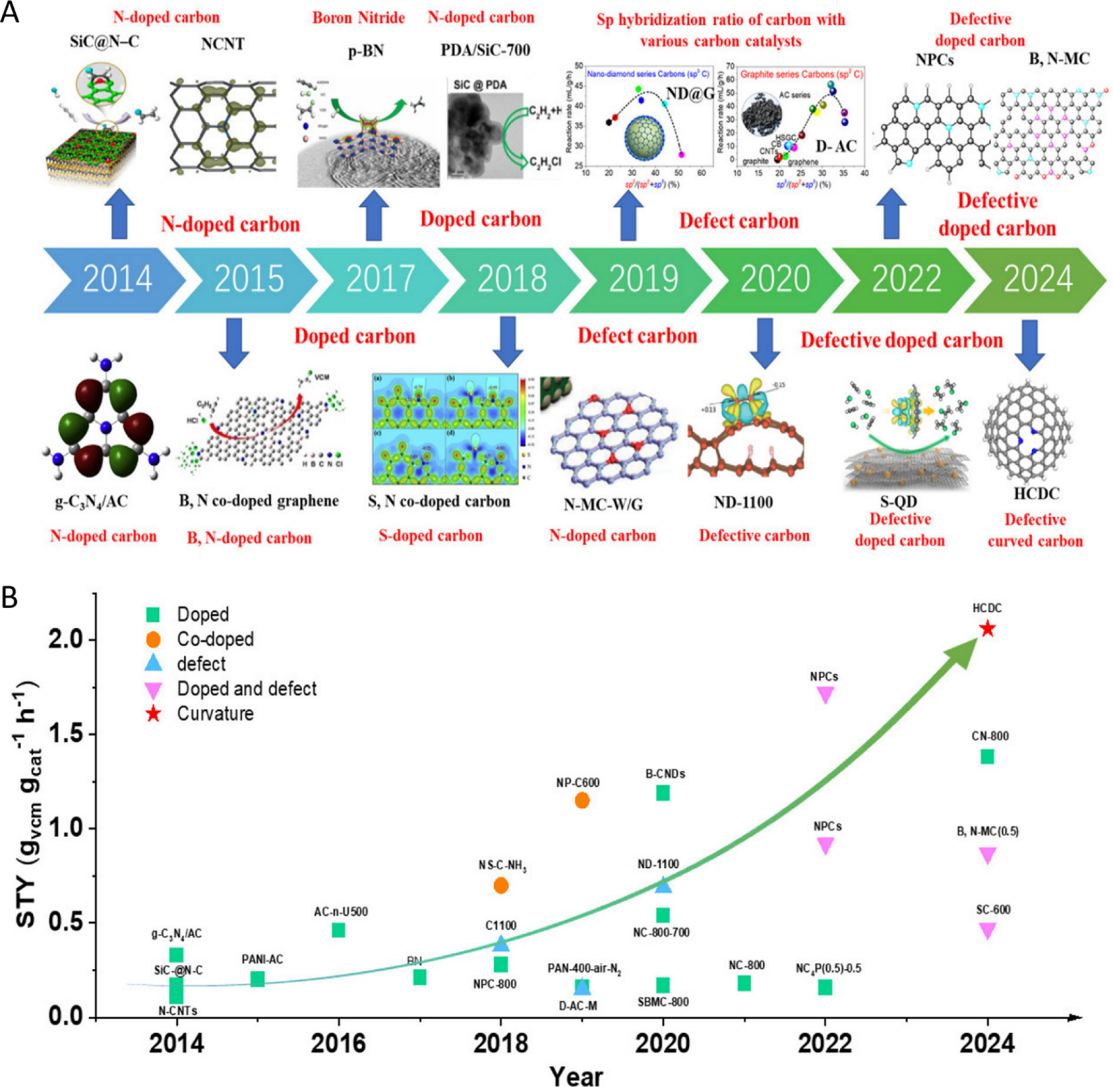
A) Progression of metal-free catalysts for acetylene hydrochlorination, from heteroatom-doped carbons to high-curvature defective carbons (HCDC); B) Timeline of carbon-based metal-free catalysts and their vinyl-chloride space–time yields (STYs). Reproduced with permission from ref.^552^ Copyright 2025, Elsevier.

##### 4.4.1.3 Advanced Oxidation Processes in Water and Wastewater Treatment

Porous carbons are widely used as catalysts in liquid-phase oxidation of organic pollutants, particularly in advanced oxidation processes (AOPs) such as catalytic ozonation,^510,512,559–565^ catalytic wet air oxidation,^566,567^ and catalytic wet peroxide oxidation.^507,568,569^ The catalytic decomposition of ozone (O_3_) in water by activated carbon to produce reactive oxygen species (ROS) like ozonide radical (O_3_•^–^), hydroxyl radical (•OH), superoxide radical (O_2_•^–^), hydrogen peroxide (H_2_O_2_), and singlet oxygen (^1^O_2_) was one of the first documented metal-free catalytic processes. Pioneering work by Jans and Hoigné showed that carbon suspensions accelerate the transformation of O_3_ into reactive radicals (like •OH) in solution.^570^ Subsequent studies confirmed that both the chemical and textural properties of carbon strongly influence ozone decomposition. Specifically, carbons with higher surface basicity (i.e., enriched in basic oxygen groups or graphitic π-sites) are much more active for ozone decomposition. Rivera-Utrilla et al.^512^ and Alvarez et al.^511^ found that O_3_ is reduced on basic carbon surfaces to produce OH^–^ and H_2_O_2_, which then yield hydroxyl radicals (•OH) that are the true oxidants that mineralize pollutants. They identified delocalized π-electrons (graphitic carbon sites) and basic oxygen functionalities such as chromene and pyrone groups as the likely catalytic centers for O_3_ activation. These conjugated base sites can donate electrons to O_3_, initiating its breakdown (for example, chromene-like structures react with O_3_ to form surface ozonides or radicals).

When carbons are N-doped, additional active sites emerge for ozonation. Basic N groups, especially pyrrolic (N-5) sites, were shown to act as active centers for O_3_ decomposition.^513^ An ozone attack on a pyrrolic group can yield an N-oxide group and a superoxide (HO_2_•) radical, which then accelerate radical chain reactions. Thus, N-doping enhances O_3_ conversion by providing more electron-rich sites (pyrrolic, pyridinic) that can initiate radical formation. Faria et al.^571^ also noted that the electrostatic interactions matter: if the carbon’s point of zero charge (pH_PZC_) is above the solution pH (making the carbon surface positively charged), it will attract OH^–^ from water, aiding in ozone’s conversion to •OH radicals. This explains why highly basic carbons (high pH_PZC_) perform best, especially in neutral pH conditions.

The first mechanism of catalytic ozonation with carbon that was accepted by the research community was a free-radical chain mechanism. The carbon surface (particularly its basic sites) initiates the chain by converting O_3_ to O_2_ and an oxygen radical (O_2_•^–^ or HO_2_•). These radicals then produce •OH in a solution, which oxidizes organic pollutants.^513^ Another proposed mechanism involves activated carbon adsorbing the organic compounds, which then react on the surface both with O_3_ and oxygen radicals. Both mechanisms can occur simultaneously, as shown for the ozonation of dye solutions, where the addition of a radical scavenger only partially decreased the activity when using activated carbon, contrarily to a cerium oxide catalyst where only the solution reaction was promoted.^510^ It has been generally found that the ozonation activity drops after the first uses as surface basic sites decrease (carbon becomes more oxidized and acidic, due to a clear increase in the acidic oxygenated surface groups with electron-withdrawing properties), decreasing the ozone decomposition to produce radicals and the adsorption capacity of organic pollutants. Designing carbons with stable basic sites (e.g., quaternary N that does not oxidize easily) could be a strategy for robust ozonation catalysts.

Recently, there has been a significant increase in the study of catalytic ozonation using nanocarbons (e.g., CNTs, GO, and r-GO, both doped and undoped), which has been reviewed in detail by the groups of Y. Xie^572^ and S. Wang.^573^ Interestingly, the previous findings for porous carbons were confirmed and reinforced. Specifically, the coexistence of a nonradical and a radical pathway was confirmed. Figure 17A summarizes the current view: (i) the radical pathway involving the formation of free and surface-confined •OH, which attack organic pollutants unselectively, with O_2_•^–^ and H_2_O_2_ also present as mild oxidants; (ii) the nonradical pathway involving the formation of activated surface-O_3_ complexes and a surface-adsorbed atomic O (*O_ad_) as nonradical ROS with high oxidation potentials capable of attacking aliphatic acids, and at the same time ^1^O_2_ are often detected, which have a mild oxidation potential and are able to oxidize electron-rich aromatics.^573^ The role of different ROS in the reaction mechanism remains debatable. They can be identified using quenching/trapping methods. However, due to the high concentration of quenchers/trapping agents typically employed, the results can be misleading causing the misinterpretation of the catalytic ozonation mechanism.^574,575^ Nevertheless, EPR spectroscopy in the presence of selected spin traps is a powerful qualitative tool for identifying ROS. The probe method (using low-concentration probes) together with principle-based kinetic models is recommended for revealing the role of ROS in catalytic ozonation.^576^ Regarding the active sites, there is a consensus that they are associated with the basic surface groups. Ma and collaborators^577^ employed N-doped carbon hollow spheres in the catalytic ozonation of ketoprofen and discovered that graphitic N promoted pollutant degradation by transferring electrons from the pollutant molecule to O_3_ via a nonradical reaction process, whereas pyrrolic and pyridinic N, located at defects in the graphitic structure, promoted the decomposition of O_3_ into •OH for degrading ketoprofen via a radical oxidation process (Figure 17B).

**Figure 17:**
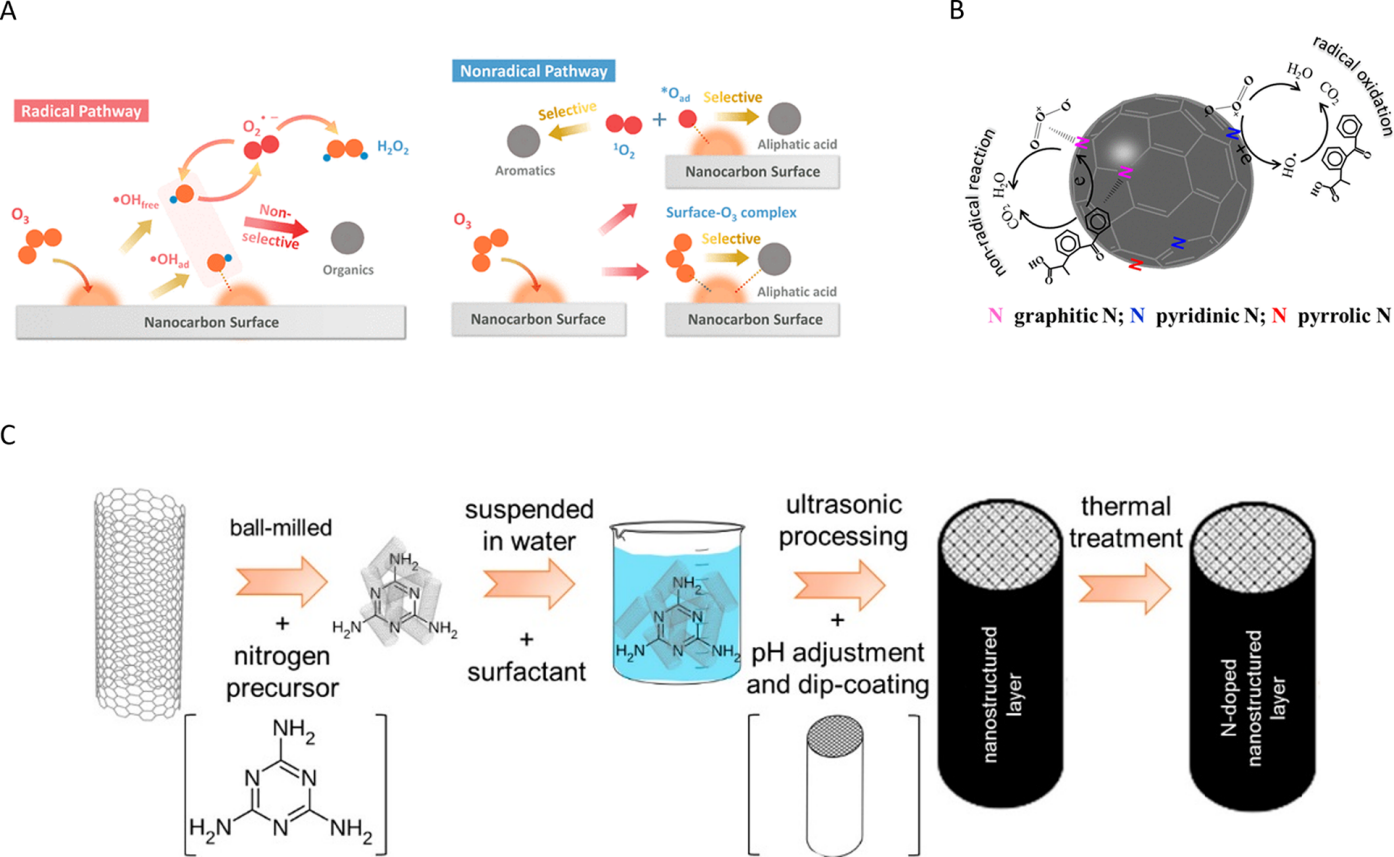
A) Schematic illustration of a nonradical and a radical pathway for the O_3_ dissociation on the surface of nanocarbons toward the formation and evolution of diverse ROS. Reproduced with permission from ref.^573^ Copyright 2020, American Chemical Society; B) Illustrative representation of the mechanism of the catalytic ozonation of ketoprofen with N-doped carbon hollow spheres. Reproduced with permission from ref.^577^ Copyright 2019, American Chemical Society; C) Synthesis of a nitrogen-doped MWCNTs-nanostructured layer supported on cordierite macrostructures. Reproduced with permission from ref.^586^ Copyright 2020, American Chemical Society.

DFT calculations, combined with machine learning models, have become an increasingly important tool for studying various reaction pathways and for predicting the catalytic performance of novel catalysts. For instance, Yu et al.^578^ investigated various pathways for O_3_ activation in N-doped defective carbons, considering ten potentially active sites. The DFT results indicated that O_3_ decomposition into *O_ad_ on the pyrrolic N can react directly with the organics, whereas *O_ad_ formed on the other sites promotes the generation of ROS. Although ^3^O_2_ states are generally predominant, ^1^O_2_ can be generated on the N and C sites of the N_4_V_2_ system (quadri-pyridinic N with two vacancies) and on the pyridinic N site at the edge. Machine learning models were then applied to correlate the O_3_ activation activity with the catalyst’s surface properties. By combining experimental and computational calculations, López-Francés et al.^579^ studied the catalytic ozonation of oxalic acid over several cyclodextrin-derived graphitic carbons. They demonstrated that the active species is ^1^O_2_ (its presence was confirmed by electron spin resonance measurements with trapping agents), which is obtained from the interaction of O_3_ with the carbon surface. ^1^O_2_ then reacts with the graphitic domains of the carbon surface via an endoperoxide intermediate, promoting the degradation of oxalic acid. To rule out the possibility that oxygen-containing surface groups are the active sites, the authors used a similar approach to that previously proposed by Su^342^ and described in Figure 15A, using phenylhydrazine, benzoyl chloride, and phenacyl bromide to selectively mask C=O, C–OH, and C–OOH groups, respectively. This confirmed that none of these groups were involved in the reaction mechanism, leading the authors to conclude that C=C graphene bonds are most likely the active sites for catalytic ozonation.^579^ Although outside the scope of the current revision, research is now emerging on the use of single-atom catalysts based on non-noble metals (e.g., Fe, Co, Mn, Ni) supported on carbon catalysts in a metal-N_4_ structure for catalytic ozonation.^580,581^

Beyond ozone, catalytic wet air oxidation (CWAO) using oxygen as an oxidant, which requires working at moderate/high temperatures and pressures, and catalytic wet peroxide oxidation (CWPO) or peroxide activation by carbons, which, like ozonation, usually works at room temperature and pressure, follow similar principles. Activated carbons can activate oxygen on the surface to originate ROS in CWAO^567^ and catalyze the decomposition of hydrogen peroxide (H_2_O_2_) to produce hydroxyl radicals in CWPO.^507^ The active sites for both reactions are the same previously assigned for catalytic ozonation. Pereira and collaborators studied the CWAO of oxalic acid (a model organic compound that is refractory to noncatalytic oxidation) over a series of MWCNT with different surface chemistries and observed an increase in the activity with the pH_PZC_.^514^ Later, using N-doped MWCNT prepared by an easy ball-milling methodology, they compared the mineralization of oxalic acid in CWAO with that in catalytic ozonation.^515^ Under the chosen operation conditions, oxalic acid in CWAO was completely mineralized in 5 min, whereas catalytic ozonation required 4 h. The outstanding performance was ascribed to the high content of N-functionalities incorporated into the carbon lattice, mainly as N-6 groups. An XPS analyses before and after repeated runs confirmed that these N-groups remain largely intact, especially during CWAO. In catalytic ozonation, some deactivation was observed, in line with an increase in the number of surface carboxylic groups, resulting in a mild loss of the activity accompanied by an increase in the number of surface carboxylic groups, consistent with the oxidation of the carbon surface by ozone. Comparable results were reported for N-doped carbon xerogels, with the activity in both processes correlated with the N-content, and the samples showed a good stability in CWAO.^582^ Finally, experiments with a radical scavenger indicated that oxalic acid degradation in CWAO proceeds mainly through a surface reaction pathway.

Sulfur-doped carbons produced by treating the precursor with concentrated H_2_SO_4_, or with the mixed HNO_3_/H_2_SO_4_ acid, exhibit a very strong Brønsted acidity and outstanding activity in the CWAO of phenol.^125^ This result seems to be contrary to the general rule, where activity increases with the basicity of the sample. Nevertheless, the activity was justified by the presence of surface sulfonic groups. At the reaction temperature employed for CWAO (140–160 °C), these groups thermally decompose, originating sulfate-derived radicals that drive phenol mineralization in the solution. Because the acid sulfonic groups are consumed in the process, almost all activity is lost after the first catalytic cycle, with XPS and TPD confirming the disappearance of these S-species after reaction.^125^ Other authors have reported that carboxylic acidic groups created upon HNO_3_/H_2_SO_4_ acid treatment of a carbon material are active sites for CWAO.^583,584^ Owing to the modification protocol used, their samples likely contained sulfonic groups, which would be most probably the true catalytic sites. In catalytic ozonation, by contrast, the presence of sulfur functionalities is detrimental, in line with the general observation that the higher surface basicity favors this process.^585^

N-doped MWCNT prepared by ball-milling pristine MWCNTs with melamine has been shown to be a very active catalyst for oxalic acid ozonation, as mentioned above.^515^ Nevertheless, nanosized powders are difficult to use in water-treatment plants. To overcome this, Pereira and co-workers immobilized the pretreated MWCNTs on cordierite honeycomb monoliths, which are robust macro-supports that operate with negligible pressure drop in continuous column reactors. Their procedure involves dip-coating the monolith in an aqueous slurry of the ball-milled N-MWCNTs stabilized with Triton X-100; after drying and calcination in N_2_ (∼550 °C) the surfactant (and any optional organic binder) decomposes, leaving a uniform, strongly adhered carbon layer (see scheme in Figure 17C).^586^ The resulting structured catalyst presented a heterogeneous first-order rate constant almost three-times higher than a reference monolith coated by conventional CVD growth of N-doped MWCNTs. This gain was attributed to the larger fraction of pyridinic-nitrogen (N-6) sites and to the thinner, more permeable wash-coating produced by the ball-milling/dip-coating route. Ultrasonic adhesion tests confirmed that virtually no mass was lost from the coating, meeting the mechanical stability expected for a real-scale operation.

Therefore, general observations for liquid-phase oxidations with carbon catalysts, which play a key role in water and wastewater treatment, can be summarized as (i) Mechanisms involve reactive oxygen species. The carbon facilitates the generation of surface-adsorbed atomic O and radicals (•OH, O_2_•^–^, etc.), which carry out the oxidation in the bulk solution or can react on the surface with adsorbed species. Catalytic ozonation follows both pathways; CWAO mainly occurs on the surface for N-doped carbons. (ii) Basic carbons are the best catalysts. Carbons with higher pH_PZC_ or electron-donating groups promote the formation of ROS more effectively. N-doping is a good strategy for increasing activity. (iii) Acidic carbons (mainly those with electron-withdrawing groups, like carboxylic acids) are less effective or may even scavenge radicals. (iv) Carbons with an extended mesoporosity are usually a better option to avoid mass transfer limitations in water treatment processes.

However, AOPs have some limitations that require further study. First, complete mineralization of organic pollutants is not guaranteed, and it is not assured that the resulting organic byproducts are less toxic than the original compounds. The second limitation is the production of inorganic compounds with some toxicity; the most relevant ones are nitrates and bromates. Particular attention is given to the presence of bromates after ozonation, as they may require an additional treatment for their removal. An integrated catalytic treatment system has recently been proposed for continuously removing organic and inorganic pollutants from water.^587^

#### 4.4.2 Electrocatalysis by Metal-Free Carbon Materials

Carbon materials constitute a key component in many electrocatalytic-based devices. They have a clear relevance in electrochemical energy generation systems, and commercial fuel cells are one representative example. In fuel cells, carbon materials are used in the current collector, the gas diffusion layer and as catalyst supports for both anode and cathode.^588–590^ If we focus on the electrocatalysts, recently, metal-free carbon-based electrocatalysts are a subject of extensive research effort since they can be a feasible alternative to the precious metal-based electrocatalysts that are used in commercial devices. In fact, the application of precious metals as catalytic species hinders the commercialization of the electrochemical devices that are essential components in the pool of environmentally friendly energy conversion and storage technologies. The research done in the last 20 years demonstrated that the synthesis of new and improved carbon materials for their application as electrocatalysts is a rapidly developing field in which researchers mainly focus on the preparation of a material that can have a performance similar to that of a precious metal-based counterpart, but pay less attention to unveiling the role of the carbon materials properties responsible for their amazing electrocatalytic ability. Carbons constitute an enormous family of materials with a wide range of structures and nanostructures at long- or short-range distances, and with various porosities and surface chemistry. The combination of all these parameters contributes to the electrocatalytic properties and, consequently, to the understanding of the role of the different parameters, which is essential to achieve the best electrocatalyst and, if possible, the best multifunctional electrocatalyst. In this sense, the effects of defects, surface charge, dopants, structural order, and porosity of carbon materials in their electrocatalytic properties must be emphasized.^21,65,591,592^

Great interest has been dedicated to defect-engineering in carbon materials, there are since correlations between the electroactivity and stability of carbon-based catalysts and intrinsic or induced defects. Structural defects and disorder are intrinsic in carbon materials and are present in higher concentrations in short-range structural ordered materials. They are usually formed during synthesis or post-treatments, and they include vacancies, voids, dislocations, nonhexagonal topologies, grain boundaries, etc.^21^ Among the most relevant for the generation of electrocatalytic sites, vacancies, and Stone–Wales defects should be mentioned. Vacancies consist of point defects in which one or few carbon atoms are missing and, as a result, local changes in C atom hybridization and electron distribution occur. As an example of the vacancy defect, which is considered of interest in electrochemical reactions, is the combination of pentagon–octagon–pentagon carbon rings (D858 or (585) defects).^593,594^ Carbenes or/and carbynes at the edge of a vacancy have been proposed as electrocatalytic active sites.^595^ Stone–Wales defects involve changes in the rotation of C–C bonds of π-bonded carbon atoms that result in the formation of five- and seven-membered rings (D5775) that change the electroneutrality of the local region containing the defect.^593^ In all cases, the defects modify the electronic structure and charge density of carbon atoms compared to the perfect graphene layer. This leads to sites with a higher activity and reactivity and that effect is similar to that brought by heteroatom doping. The possibility of synthesizing specific defects in the adequate concentration without compromising other important properties like electrical conductivity is a route to produce highly effective catalysts.

Textural properties, including the porous structure are another important factor often overlooked in the literature. Porous carbons are materials that may have a wide range of surface areas, pore size distributions and porous structures and these properties have an undeniable influence on the catalytic performance. Pores in the range of micropores (pores of size below 2 nm) can act as nanoreactors facilitating or being the active sites for reactions. This makes the size and geometry of pores critical, not only because they may contain catalytic sites or participate in a reaction, but also because they are essential for achieving adequate mass transfer and residence time for the reactants and products.^596^

Doping carbon materials with heteroatoms (mainly N, B, S, and P) is the most studied topic in this field.^480,597,598^ The incorporation of heteroatoms may produce local structural changes, a charge redistribution, changes in electronic properties and defects,^480^ which are responsible for the outstanding electrocatalytic activity found in the literature. The most studied heteroatom is nitrogen because there are different straightforward routes for its incorporation into the carbon matrix and it is possible to adapt the experimental conditions to generate the most active functionalities with their high amounts on the surface. Although other heteroatoms do not seem to produce high electrocatalytic activity sites, codoping or multidoping with more than one heteroatom (especially N plus another heteroatom) seems to produce much better activities due to synergistic effects.^599^

Although quite interesting materials have been synthesized by different strategies and combining different factors indicated above, and they are promising as catalysts and catalyst supports for different electrochemical energy conversion processes,^600^ the understanding of the reaction mechanism is still far from the desired level to allow the practical application of the metal-free carbon-based electrocatalysts. Future challenges include the synthesis of multifunctional catalysts that can be applied to different electrochemical reactions.

Metal-free porous carbon materials have been used as electrocatalysts in devices for different energy technologies, such as fuel cells, water electrolyzers and fuel production from a CO_2_ reduction reaction (CO2RR). Among the three mentioned processes, porous carbon materials have been the most studied as electrocatalysts for the oxygen reduction reaction (ORR) due to their outstanding performance. In the case of water electrolysis, although some studies have been published on the use of porous carbons as electrocatalysts for the hydrogen evolution reaction (HER),^601^ their performance is far from that of the precious-metal containing catalysts.^602^ On the other hand, porous carbons have been much studied for the electrochemical storage of hydrogen.^603–606^ This research has shown that porous carbons can store hydrogen produced from H_2_O dissociation at negative potentials, and that the amount stored depends on the porosity and surface chemistry and the storage mechanism is related to the formation of C–H bonds. This hydrogen storage mechanism is due to the high overpotential for the H_2_ evolution, in agreement with the low electrocatalytic activity of these materials. In the case of CO2RR, the literature available is still not numerous; however, there are reports clearly indicating the role of carbon chemistry in this process.^468,607–615^ Consequently, in this review, we mainly focus on the ORR, with less stress on CO2RR, as the electrocatalytic processes showing the potential of carbon materials applications as electrocatalysts and the relevance/interplay of their surface chemistry, porosity and structure in the electrocatalytic performance.

##### 4.4.2.1 Oxygen Reduction Reaction

The ORR is undoubtedly the most studied due to the very promising results obtained that have given rise to an important number of reviews on this topic.^480,597,598,616–619^ This is a very important reaction that shows slow reaction kinetics and an enormous technological relevance. In the specific case of fuel cells, platinum is the catalyst used both in the anode and the cathode. Pt-loading in the anode electrode is close to 0.05 mg·cm^–2^, whereas in the cathode electrode it is more than five times higher due to the unfavorable kinetics of the ORR,^617^ resulting in more than 80 wt % of platinum incorporated to the surface.

Before discussing the topic, we should comment on the experimental electrocatalytic measurements because, frequently, either wrong experimental procedures are used or wrong data analyses are provided. For this reason, some kind of normalization of electrocatalytic properties should be applied to allow researchers from different disciplines to synthesize, characterize and publish comparable data on electrocatalysts across various reactions. Some publications detail best practices and testing protocols for electrocatalysts characterization. Most of them address the ORR^620–625^ —where the use of rotating disk electrodes is required—and the HER.^626^ Nevertheless, the understanding of the fundamentals of the electrochemical methods and how they can be applied for a correct experimental evaluation of a studied system is necessary. For this purpose, some essential books are recommended and one of them is the well-known textbook by Bard and Faulkner.^627^ When using a rotating ring electrode, special attention should be paid to the roughness of the film, presence of pin-holes, pores, etc., that have a strong influence on the measured experimental results.

There is a general agreement that the ORR in aqueous solutions occurs mainly by two pathways, a two-step reduction pathway (2 + 2 electron mechanism) consisting of two consecutive reactions and a direct four-electron reduction, which is the most energy-efficient route.^628^

The electrocatalysis of the ORR is considered as one of the challenging subjects in electrochemistry, which has been studied for over a century with attempts to understand the reaction mechanism and to identify the most adequate catalyst. In the review on this topic by Yeager,^629^ the catalysis on carbon and graphite was described to proceed through peroxide pathway because in the 80s of the last century, metal-free carbon electrocatalysts working through the four electrons pathway were still not discovered. It is interesting to note several important aspects that have been already remarked in the electrocatalysis of the ORR by carbon materials and that are of great importance in the followed research on this area: (i) the electrocatalytic activity of a graphite basal plane is much lower than that of glassy carbon or pyrolytic graphite, which (as we know) contain a high concentration of edge sites and defects; (ii) the reaction mechanism is described as occurring through the formation of adsorbed superoxide species following an associative mechanism without a bond scission in the O_2_ molecule and (iii) quinone groups seem to play a role in the ORR to peroxide as catalytic sites for this reaction. It is also important to remark that the reaction mechanism described for Pt surfaces or transition metal complexes is very similar to that used for metal-free N-doped carbon electrocatalysts that catalyze the reaction through the four electrons pathway (i.e., associative, or dissociative with respect to the O_2_ chemisorption). Figure 18 summarizes the most proposed reaction schemes for catalysis of the ORR by carbon materials (either nondoped or heteroatom doped), which include different elementary steps that may proceed through a 2 or 4 electrons pathway.

**Figure 18:**
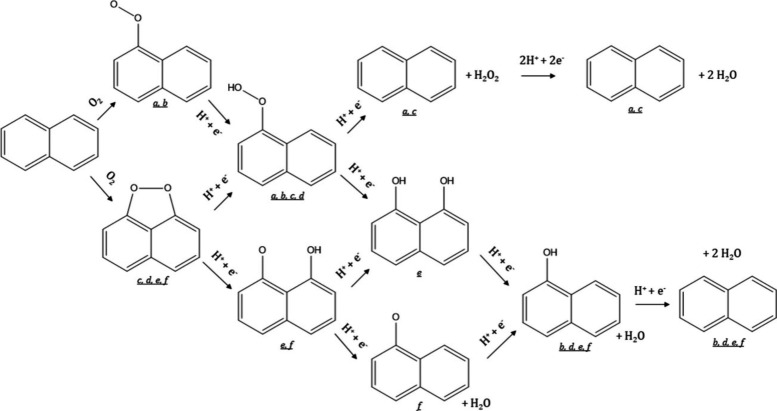
Representation of the adsorption mode, electron pathway and mechanisms of the different proposed ORR paths in the scheme. a) Adsorption mode: terminal, 2 electrons pathway and mechanism: associative. b) Adsorption mode: terminal, 4 electrons pathway and mechanism: dissociative. c) Adsorption mode: bridging, 2 electrons pathway and mechanism: associative. d) Adsorption mode: bridging, 4 electrons pathway and mechanism: associative. e) Adsorption mode: bridging, 4 electrons pathway and mechanism: dissociative. f) Adsorption mode: bridging, 4 electrons pathway and mechanism: dissociative. Reproduced with permission from ref.^617^ Copyright 2020, Elsevier.

In spite of the important progress on this topic, the elementary steps involved in the above-mentioned pathways as well as the nature of the electrocatalytic active sites are still the subject of intense research and deeper knowledge is still necessary for the adequate design and synthesis of the most active and stable electrocatalysts, that can compete with the precious metal-based electrocatalysts in different electrolytes. The complexity of the system arises because of the multiple adsorption/desorption and electron transfer reaction steps that participate in the ORR, the presence of competing side reactions, the complex properties of carbon materials in terms of structure, porosity and surface chemistry, and the effect of the electrolyte, as the main factors.^617–619,630,631^ Understanding surface chemistry of carbon materials and, more specifically, interfacial chemistry and electrochemistry of carbon surfaces^632^ is essential to comprehend the electrocatalysis of the ORR on these materials. The chemical state of the carbon surface under polarization conditions, in the presence of an electrolyte and the influence of the porosity, will determine the reaction mechanism and the electrocatalytic activity. It is because they not only strongly affect the interactions with the reactants but they also affect the structure of the double layer and the stability of the intermediates.^619,630,631,633,634^

##### 4.4.2.2 Influence of Porosity on the ORR

Regarding the *influence of porosity* on the kinetics and catalytic activity of the ORR, the literature does not dedicate as much attention to it as to other parameters such as heteroatom doping. However, this is a very important property of carbon materials that can affect the electrocatalytic activity from different perspectives. The porosity has a strong influence on the mass transport and the effect of mesopores in this specific aspect is well-known from different areas of adsorption and heterogeneous catalysis. The role of porosity in the kinetics of the ORR has been assessed in nondoped carbon materials, showing that their performance is undoubtedly related to their porosity.^596,635^ The modeling study reached the conclusion that well-developed microporosity (pore size <2 nm) is important in O_2_ reduction.^596^ In addition, the narrow microporosity (ultramicropores, pore size <0.7 nm) positively affects the HO_2_^–^ reduction in an alkaline environment,^596^ suggesting that the differences in the adsorption potential and mass transfer between wider micropores and narrow micropores may explain the higher activity for this reaction in the narrow microporosity. Figure 19A presents a scheme that summarizes the effect of narrow and wide microporosity in catalysis of the ORR in porous nondoped carbon materials. The relevance of microporosity in the reduction of H_2_O_2_ or its disproportionation reaction has also been observed in acidic electrolytes, demonstrating the relevance of the increased residence time in the microporosity to increase the rate of the hydrogen peroxide conversion to H_2_O.^636^ While the microporosity is considered to positively affect the activity toward the ORR from the viewpoints of the onset potential and the number of electron transfer, the mesoporosity (diameter 2–50 nm) is also essential as mass transport pathways for O_2_ to facilitate the access to these active sites.^637^

**Figure 19:**
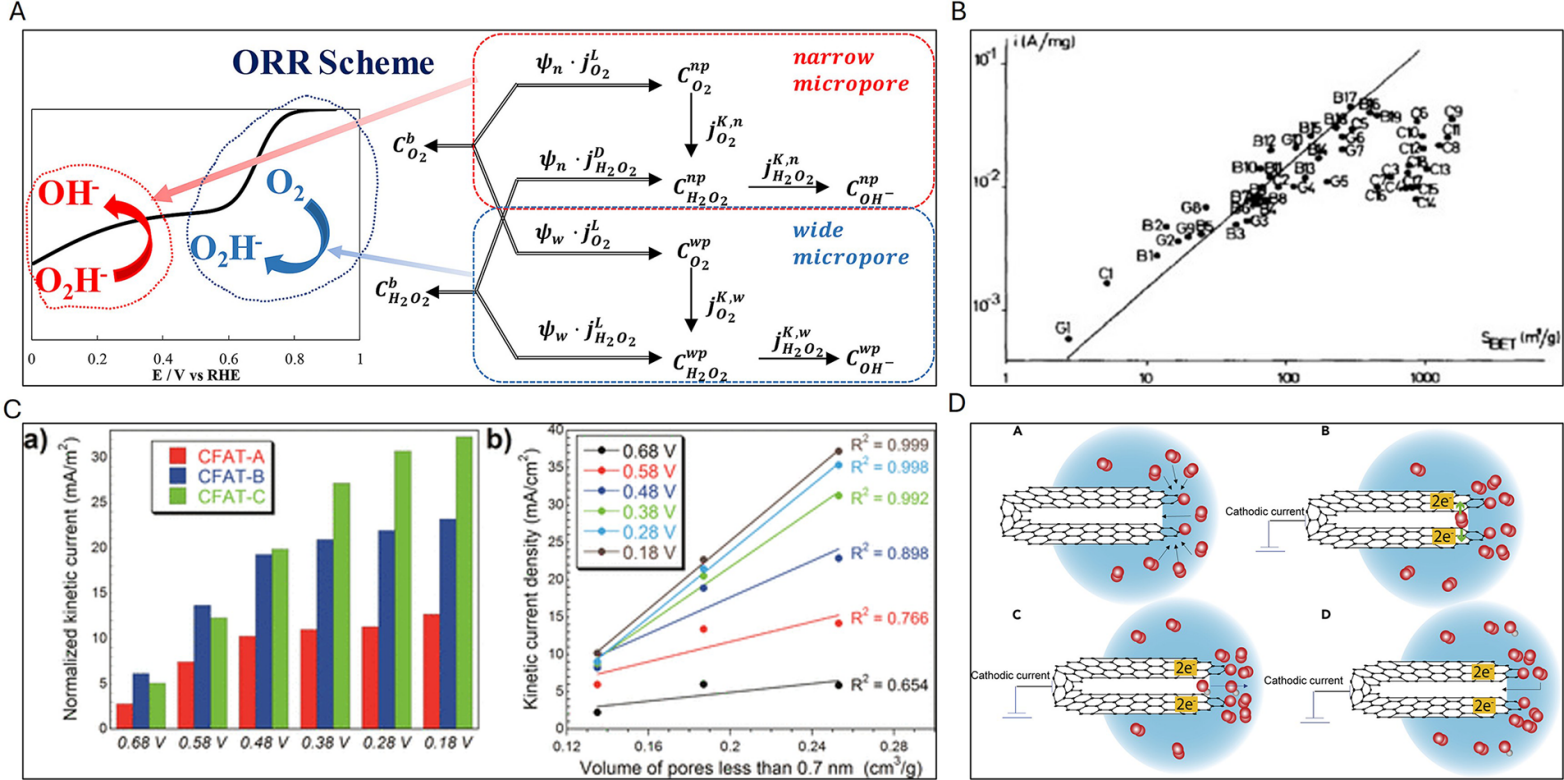
A) Scheme for the heterogeneous micropores model proposed for the mathematical description of the ORR mechanism of porous carbon materials. Reproduced with permission from ref.^596^ Copyright 2019, Elsevier; B) Activity for oxygen reduction (A mg^–1^) at 0.85 V vs NHE, 6 N KOH, 1 atm. O_2_, as a function of BET area/g carbon. Reproduced with permission from ref.^596^ Copyright 1979, Elsevier; C) a) Normalized kinetic current densities per *S*_BET_ surface area of carbon materials having a micro/mesoporous texture and a high volume of macropores; b) Dependence of the kinetic current density vs volume of pores smaller than 0.7 nm. Reproduced with permission from ref.^638^ Copyright 2016, American Chemical Society; D) Visualization of ORR on CMS: (A) Withdrawal of oxygen from an electrolyte by the hydrophobic surface of small pores and their high adsorption potential; attraction of oxygen to ethers, (B) Splitting of O_2_ molecule due to strong adsorption forces from both pore walls followed by its reduction; (C) With the help of cathodic current the reduction of oxygen in pores takes place accompanied by its protonation owing to the proximity of water; OH^–^ is formed and released to water owing to its affinity to the water phase and (D) Oxygen accumulated on ethers enters pores to be further reduced. Reproduced with permission from ref.^639^ Copyright 2021, Elsevier.

Although microporosity undoubtedly plays a key role in the activity of porous carbons, this effect is ignored in most of the literature because the presence of other catalytic species with high catalytic activity, or sites that are heterogeneously distributed on the carbon surface, may hinder the assessment of its contribution. In fact, the presence of a high concentration of highly active sites may lead to the conclusion that the catalytic sites prevail over the porosity,^640^ which can be a correct observation for some specific electrocatalysts. However, other studies found a correlation between the catalytic activity in N-doped carbon materials and porosity (and ultramicropores).^641–643^ These results demonstrated that the microporosity, and especially the narrow micropores, must be considered as essential players to explain the ORR activity in carbon materials since this feature is present in most of them.

Few works report a systematic approach for assessing the role of the microporosity of bare carbon materials. In this sense, the study by Appleby and Marie was one of the first that reported a direct relationship between the ORR activity of carbon blacks in an alkaline solution and their BET surface area^644^ (Figure 19B). However, this tendency was not found in the case of activated carbons (ACs). The authors concluded that although the high surface area provides a higher concentration of active sites, those existing in the micropores are not accessible to the electrolyte, including dissolved oxygen molecules. On the other hand, Liu et al. studied the contribution of micro and mesoporosity in ORR electrocatalysts^637^ and found an increase in the activity attributed to the microporosity, while the mesoporosity facilitated the accessibility to the active sites. Following this conclusion, Bandosz et al. prepared hydrophobic ultramicroporous carbon catalysts^638^ and found that the ultramicropores (i.e., pores of size below 0.7 nm) have an important role in the ORR activity (Figure 19C) since the strong adsorption of dioxygen molecule in the hydrophobic ultramicropores weakens the dioxygen molecule bond, promoting its splitting^645^ (Figure 19D). Further studies performed with nondoped and N-doped microporous carbons strengthened the role of the ultramicropores.^641,643^ Moreover, the results suggested that a precise engineering of the highly active sites located in the pores of the carbon materials can lead to the good performance in different electrolytes, including acidic conditions in which the activity of metal-free porous carbons is usually poor (see later discussion on the effect of the pH). The relevance of hydrophobicity on the enhancement of the ORR activity has also been supported by the involvement of the hydrophobic interface in the formation of gas reservoir cavities that ensure a fast diffusion of the gas to the reaction site. It demonstrated the need for an efficient control of the liquid/gas/solid interface in such a three-phase system involved in the ORR.^646^ Nevertheless, the mechanism for the ORR in the presence of nanopores needs further exploration to get a more detailed view of the different steps involved in the reaction. Considering that the dioxygen reduction begins inside the nanopores, and either through an associative dioxygen reduction or a dissociative reduction, charged species would be formed that have to react with H_2_O (in alkaline media) or H^+^ (in acidic electrolyte) to continue with the reduction mechanism. Since water or hydronium cations are excluded from the nanopores, these negatively charged species formed from the first steps of dioxygen reduction have to migrate through the nanopores and leave them to continue the reaction. The feasibility of this migration mechanism should be analyzed, especially considering that these charged oxygen species may have a high reactivity toward the positively polarized carbon material under the ORR conditions. A second possibility is that the active sites are located at the edges of the nanopores, in agreement with studies done with pyrolytic carbon that revealed the much higher activity of the edges than the basal plane of graphene layers,^647^ and that the nanopores participate as a gas reservoir^639^ then providing a higher concentration of O_2_ near the active sites that will increase the reaction rate. These possibilities should be further studied through experimental and computational studies.

Recent molecular simulation studies combined with experimental results have suggested that dissolved O_2_ adsorption is enhanced in an alkaline electrolyte for the carbon whose surface is rich in dissociating groups.^648^ Under the applied voltage, a cation docking process in subnanopores increases the charge of carbon atoms in the larger pores, which favor O_2_ dissociation owing to a small energy barrier. This process can even occur spontaneously, when oxygen is in close proximity to these carbon atoms. The extent of docking depends on the cation type through the stability of its hydration shell. The events of oxygen splitting in subnanopores were also found through this simulation approach.

Another important factor that contributes to the ORR electrocatalytic activity of metal free carbon-based materials is the *curvature of the graphene layers* that build the structure of the material and, consequently, the pore structure. In addition, these curved graphene layers may contain various amounts of defects of a different chemical nature that, as a whole, will determine the catalytic activity. The curvature itself can be a consequence of graphene layer rolling, as occurs in carbon nanotubes and it is a parameter used to describe layers that are wrinkled, rippled, crumpled,^649,650^ or warped.^651,652^

Recently, the ORR activity of carbon materials with different structures and porosities was analyzed with respect to their carbon-dioxygen gasification properties (that is, carbon gasification reactivity and active surface area measured by O_2_ chemisorption).^655^ A positive linear correlation was found between the ORR activity and Active Surface Area (ASA) and gasification reactivity for all the carbon materials analyzed. The slope for both the ORR and gasification reactivity versus ASA plots was higher for the carbon nanotubes (CNT)-based samples, indicating that the active sites for both reactions are affected by the curvature (Figure 20A). The high gasification reactivity in CNT has been linked to the strong local curvature, defects and helicity.^653,654^ A correlation between the reactivity and CNT diameter was found, with the higher reactivity observed for CNT of smaller tube dimeters, which have increased curvature. Similarly, CNT showed the higher ORR activities than the non-CNT samples.^655^ The correlation observed between the ORR activity and O_2_-carbon gasification reactivity indicates that both reactions share the active sites, at least during their first steps, which involve in both cases dioxygen chemisorption. As for the CNT-gasification reaction, in the case of the ORR, it has been reported that curvature plays an important role also in oxygen adsorption, diffusion of reactants and reduction in CNT^656,657^ and that the limiting potential depends on the curvature near the active sites^658^ as well as the presence of defects.^659^ Then, high curvature, high electrical conductivity and defects population seem to be the most desirable factors for achieving the highest activity of the carbon materials.

**Figure 20:**
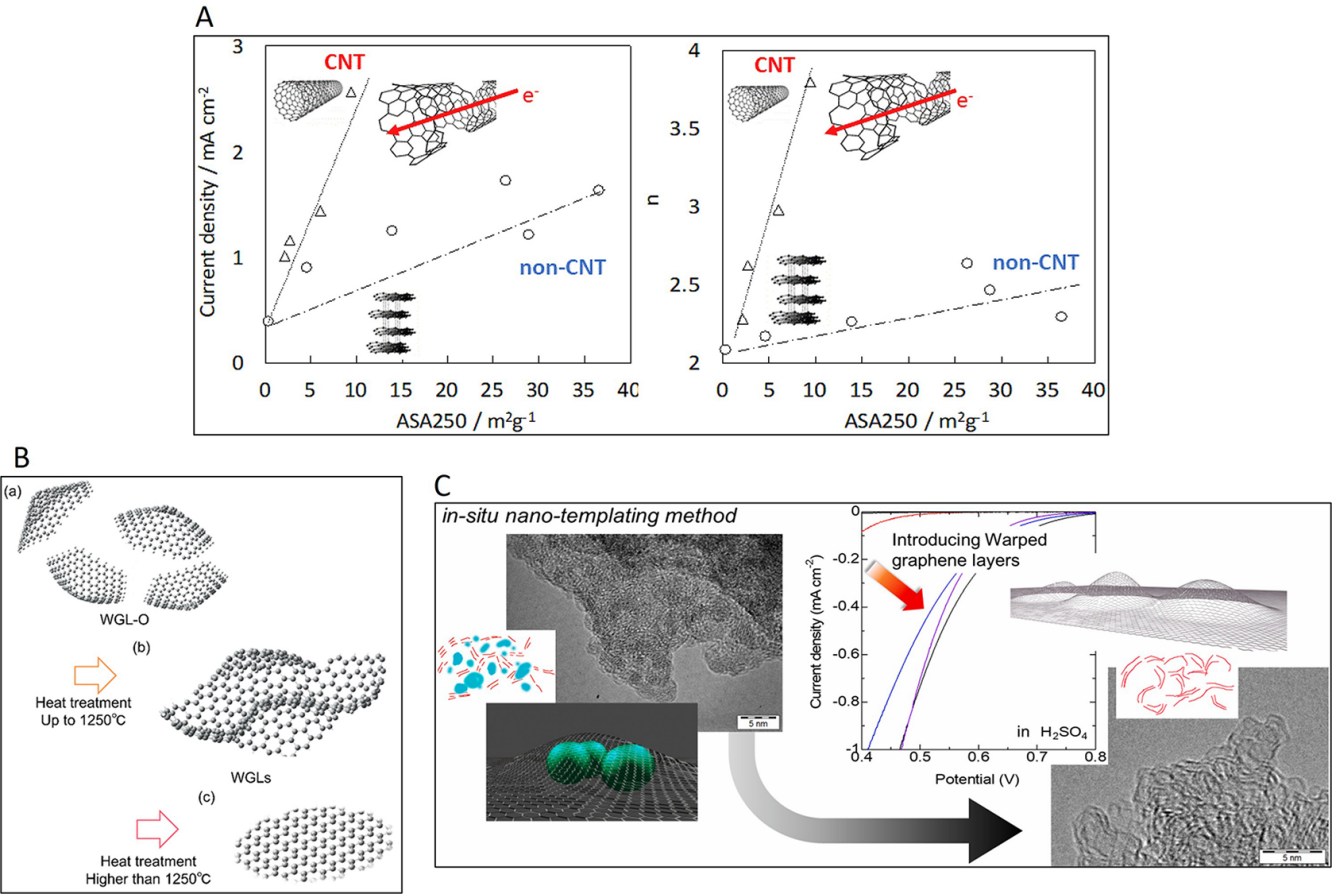
A) ASA dependence of current density and *n* measured 0.7 V in O_2_-saturated 0.1 M KOH solution at 1600 rpm. Δ: CNT-based samples (MW, SW, SW_HNO_3_, and Herring). ◯: rest of samples (KS4, XC72, CB, CNF, AC, and YPF). Sample loading is 400 μg cm^–2^. Reproduced with permission from ref.^655^ Copyright 2019, Elsevier; B) Model diagrams of (a) WGL-O (oxidized warped graphitic layers), (b) a continuous WGL, and (c) a planar structure of a WGL. Reproduced with permission from ref.^652^ Copyright 2023, American Chemical Society; C) Scheme for the efficient preparation of carbon materials composed of warped graphene layers via *in situ* nanotemplating and estimation of oxygen reduction reaction activity. Reproduced with permission from ref.^651^ Copyright 2024, Elsevier.

A recent experimental work carried out by Ozaki and co-workers^651,652^ investigated the dependence between the ORR activity and the curvature of warped graphene layers. High purity warped graphene layers with three-dimensional curvature and formed by hexagonal sp^2^ carbon atoms arrangement were prepared through different methodologies, including *in situ* nanotemplating that permits the synthesis of graphene layers with a curvature radius below 4 nm.^651^ Figure 20B contains model diagrams of the warped graphene layered materials and Figure 20C summarizes the *in situ* templating approach used. The results showed a strong correlation between the ORR activity and the curvature radius of the warped graphene layers, and the experimental data indicated that the highest activity corresponds to a curvature radius below 2.5 nm. The characterization of the materials showed that adsorption of O_2_ is favored by the curvature, that the continuity of the graphene layer results in an increase in the π-electron density and that the Fermi level for these materials is more appropriate for an electron transfer to O_2_.

The findings discussed above suggest that the active sites are present on the convex outer surface of the curved carbon materials. However, a computational study with Si-doped CNT^652^ suggested that the active sites for the ORR are in the concave inner surface of the tube because of the lower stability of the intermediates that play a key role in the catalytic activity. Nevertheless, this study was done for Si-doped CNT, where Si atoms determine the nature of the active sites. The curvature effect of the surface in dual-atom site of the ORR catalyst was analyzed in M–N–C systems by computational studies,^660^ which found that the curvature of the concave surface has a positive effect on the catalytic activity. Even though the literature shows that curvature is an important factor that contributes to the ORR, some contradictory conclusions appear regarding the location of the active sites, either on the concave or convex surface. Consequently, more efforts should be dedicated to understanding the effect of the curvature and, especially, to reconcile the computational and experimental works.

##### 4.4.2.3 Influence of Defects on the ORR

In addition to the porosity and curvature of graphene layers, defects and heteroatoms provide a more complete view toward understanding of the electrocatalytic activity of metal-free porous carbon materials. Although the role of defects in heterogeneous electrocatalysis has not been explored in depth, it is clear that defects play a very important role in creating high-activity electrocatalytic sites for various electrochemical reactions.^661^ Defect engineering may contribute to the development of carbon materials with superior catalytic performance for the oxygen reduction reaction (ORR), surpassing both pristine carbon and even competing with heteroatom-doped carbon materials.^658^ Interestingly, some points and topological defects seem to play a significant role in enhancing the ORR activity and in improving a charge transfer efficiency. Among these, pentagon-containing defects (such as 585 or Stone–Thrower–Wales defects) and carbene-like zigzag edges have been proposed as particularly effective in facilitating O_2_ chemisorption and its subsequent reduction through the four-electron pathway.^65,595,658,662^ The improvement in the catalytic activity resulting from defects seems to be a consequence of a spin/charge density redistribution from unpaired electrons within the sp^2^ carbon lattice. Theoretical studies suggested that five-membered rings act as electron acceptors, while seven- or eight-membered rings generate donor-like electronic states, thereby modifying the local electronic structure that results in an improvement in the ORR activity.^593^ It is widely accepted that the most reactive sites in carbon materials are those with local structural distortions, including edges, defects, or regions of a high curvature and strain.^658,663^ Nevertheless, achieving an optimal defect density while maintaining the integrity of the sp^2^ carbon lattice is crucial for preserving a high electrical conductivity and other desirable features for catalytic applications.^664^

Among different carbon defects, edge sites are proposed as active sites for the ORR.^647,665,666^ Shen et al. studied the ORR on highly oriented pyrolytic graphite and observed the higher catalytic activity of edge sites^647^ (Figure 21A). DFT calculations by Zhong et al.^667^ revealed an electron transfer between edge carbon atoms and the neighbor atoms that favor O_2_ adsorption. Among the zigzag and armchair edge defects, Jiang et al. proposed,^65^ based on a theoretical study, that zigzag edge defects contribute to the increased the ORR activity since they induce an increased spin density that promotes the ORR (Figure 21B). Similar results were found by Jiang et al.^668^ Radovic and co-workers^25,595^ suggested that a carbene-like nature of the zigzag edge sites makes them active in O_2_ adsorption. Figure 21 C summarizes the elementary steps in the two- and four-electron ORR occurring at the carbene-type zigzag site in graphene. Waki et al.^669^ proposed that hole defects on the walls of CNTs, created by removing CO-type groups, can give rise to local carbon restructuring and to the formation of topological defects, which constitute the active sites for the ORR.

**Figure 21:**
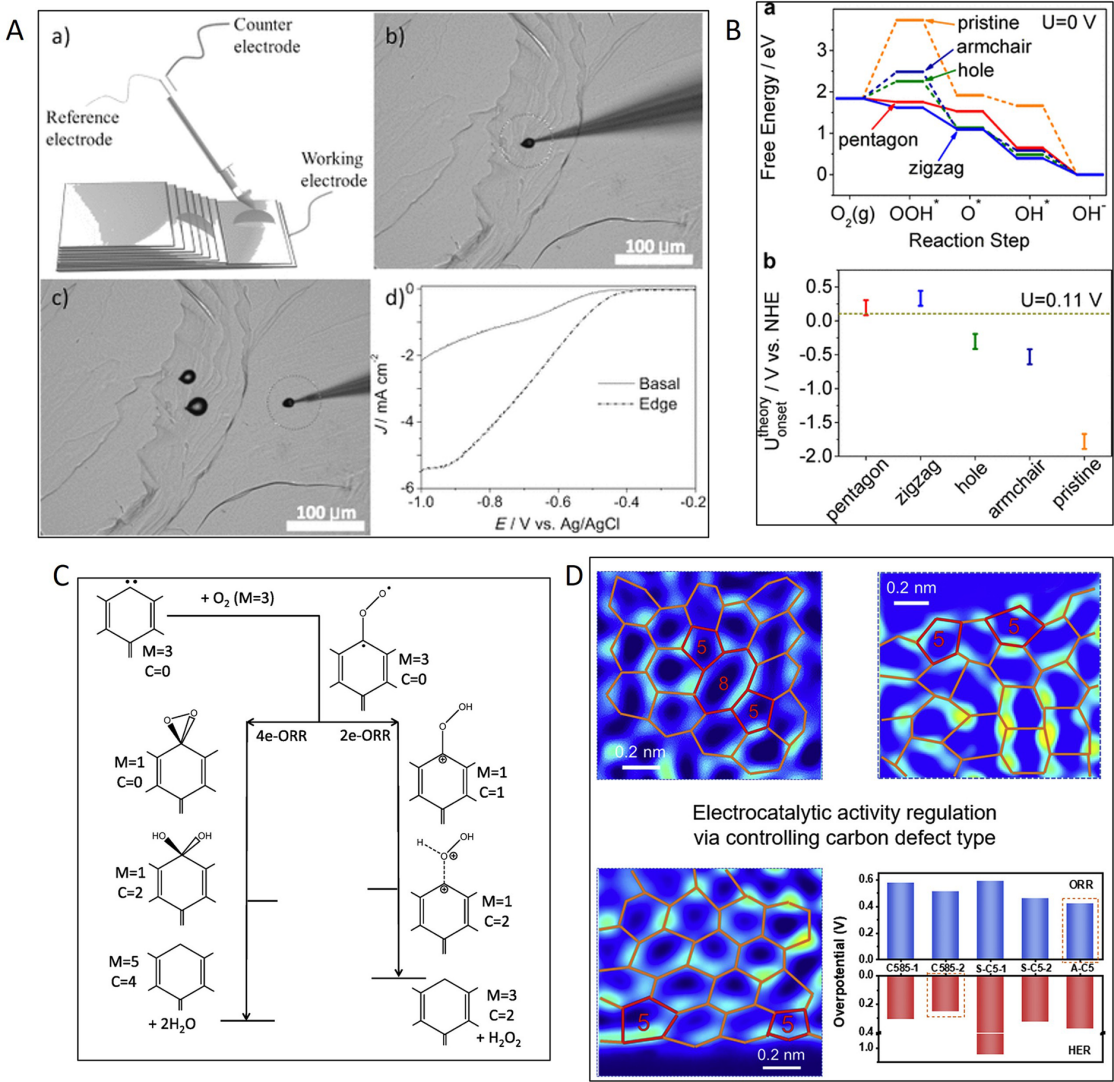
A) a) Micro apparatus for the ORR electrochemical experiment. b) Optical photograph of the HOPG as the working electrode with the air-saturated droplet deposited on the edge of the HOPG. c) The air-saturated droplet was deposited on the basal plane of the HOPG electrode. d) LSV curves of the ORR tested for a droplet located either on the edge (as shown in panel B) or on the basal plane (as shown in panel C) of the HOPG. Reproduced with permission from ref.^647^ Copyright 2014, Wiley; B) DFT calculations for ORR activities of different defects. a) Free energy diagrams. b) *U*_onset_^theory^ ranges (bars) and *U*_onset_^exp^ of CNC700 (dashed line). Reproduced with permission from ref.^65^ Copyright 2015, American Chemical Society; C) Principal mechanistic steps in the two- and four-electron ORR occurring at the carbene-type zigzag site in graphene. Reproduced with permission from ref.^595^ Copyright 2020, Elsevier; D) Identification of the Most Active Defect Sites toward ORR and HER in Carbon Catalysts via Electrochemical Test and DFT Calculations. Reproduced with permission from ref.^64^ Copyright 2020, Elsevier.

Topological defects have also been reported to be active for the ORR. Zhao et al.^670^ reported that the catalytic activity of 585 defects (pentagon-octagon-pentagon) is similar to that of Pt for the ORR, although this conclusion was reached only through DFT calculations. In contrast, Wang et al.^64^ found that pentagon-type defects exhibit the higher catalytic activity for the oxygen reduction reaction (ORR), whereas the 585 defects are more favorable for the hydrogen evolution reaction (HER). Figure 21D indicates, with dot boxes, the most active sites proposed by the authors. Additionally, theoretical studies indicated that both C5 and zigzag edge sites positively contribute to the enhancement in the ORR activity.^65^

Unfortunately, most of the conclusions about the nature of the defects that have the highest catalytic activity are obtained from theoretical studies. Experimental studies usually do not provide the unique evidence of the type of defects responsible for the catalytic activity because of the heterogeneous nature of the carbon materials, and the difficulties in assessing the nature of the defects. Evaluation of defects in carbon materials is usually done by HRTEM, STM or Raman spectroscopy. While microscopies are difficult to apply to porous carbons due to their high heterogeneity, Raman spectroscopy provides an average view of the structural properties of the material. The following references can be useful to understand how Raman spectroscopy can be applied to obtain structural information on carbon materials with the assignment of specific bands to specific types of defects. It is not straightforward, but a specific assignment could not be done with this technique.^671–675^ If we just refer to the first order spectra of carbon materials between 1200 and 1600 cm^–1^, they can be analyzed considering five bands,^674,675^ named as D, D′, G, D″, and D*. Essentially, the G band corresponds to the ideal graphitic lattice and the D bands reflect the presence of defects. The literature assigns the D’ band above 1600 cm^–1^ to different types of defects, including grain boundaries, vacancies or sp^3^ carbon atoms, and the D* and D″ bands at around 1200 and 1500 cm^–1^, respectively, can be related to disordered graphitic lattices with sp^2^–sp^3^ bonds and amorphous carbon. The position of these last two bands depends on the oxygen content. Considering the great heterogeneity of low structural order carbon materials, it is difficult to relate the specific bands derived from the deconvolution to a given type of defects.

In fact, in a recent study^676^ the catalytic activity to the ORR was measured for a variety of materials with different structures and porosities and the results were linked to the Raman spectra deconvolution results. No clear correlation was found between the ORR catalytic parameters and structural features determined from Raman spectroscopy. In another study, Tang et al.^677^ explained the role of edge and topological defects in metal-free carbon in oxygen electrocatalysis. They concluded that these defects are of critical importance to the catalytic activity of carbons and proposed, based on theoretical calculations, that pentagon and heptagon carbon rings have the lowest overpotential for both the ORR and OER. However, although the presence of those types of defects can be deduced from the Raman spectra and the TEM images, it is not possible to identify the specific type of defects using the techniques used in the studies, particularly given the high degree of disorder of the materials. Chen et al.^678^ prepared quite interesting carbon nanomaterials with a cage-like cubic morphology that have a high catalytic activity to the ORR in acidic conditions, which was related to the pentagon-like defects (usually, the catalytic activity of metal-free carbon materials is poor in these conditions). Similar conclusion was reached by Bai et al.,^679^ who considered 1,3-cyclopentadiene-like defects as the main pH-universal active sites. In the study by Chen et al.,^678^ C_60_ and C_60_-ethylenediamine were used as precursors for carbon materials without and with N-doping. In that study, the authors assigned the D″ band at around 1500 cm^–1^ (named as D3) to the pentagon type defects and its intensity was used to determine the amount of these defects. This assignment was done considering that the C60 precursor provides a large number of pentagons, which are preserved when annealed at temperatures up to 1100 °C. However, from most of the literature on Raman spectroscopy, this band is usually related to amorphous carbon and analysis of defects in graphene like materials is usually done through the D and D′ bands.^673^ Nevertheless, the results from that study are remarkable and demonstrate the importance of defect engineering at the nanoscale in order to achieve the most active metal-free carbon electrocatalysts.

As indicated above, it is very difficult to unravel the role of specific types of defects in the ORR using Raman spectroscopy, which is the technique mostly used in the literature for this purpose. Therefore, other parameters to evaluate the defects could be helpful. Considering that in the ORR the interactions between the O_2_ molecule and the carbon material followed by O_2_ dissociation are common steps to the carbon gasification reaction with O_2_, which is also governed by carbon defects, Gabe et al.^655^ studied the relationship between the ORR catalytic activity and carbon–oxygen gasification reaction for carbon materials of different structures. The Active Surface Area -ASA- was determined from oxygen chemisorption and carbon gasification reactivity was measured in oxygen. A correlation was found between both the ORR activity and ASA, although differences between carbon nanotubes and noncarbon nanotubes materials were observed due to the different structures and the influence of the curvature, as explained above. Nevertheless, the ORR activity and carbon gasification reactivity were linearly correlated for all samples, suggesting that both reactions involve the similar active sites. Later, a redox probe Fe­(CN)_6_^3–^/Fe­(CN)_6_^4–^, which is sensitive to edge sites^680^ was used to calculate the electroactive surface area (EASA) and to get additional information about the ORR activity of the materials with different structures. The EASA values obtained from the redox probe were compared with the ASA values, the reactivity in oxygen gasification, and Raman spectra of those carbons.^676^ The study showed that the redox probe is useful for evaluating the edge sites that can be the active sites for the ORR, although no precise structural information could be obtained.

In summary, although the number of defects could be estimated using probe molecules, the chemical nature of those defects cannot be uniquely determined employing the usual structural characterization of Raman spectroscopy. In addition, theoretical calculations using different structural models for the carbon materials suggested a high catalytic activity for different sites. More powerful techniques must be developed and under *operando* conditions to obtain information about the chemical changes occurring in the carbon material during the reaction. This is one of the most important challenges to be solved in order to gain a deeper understanding of the detailed reaction mechanism.

##### 4.4.2.4 Influence of Heteroatoms on the ORR

In the field of porous carbons as the electrocatalysts for the ORR, their structure, porosity, defects and the *presence of heteroatoms* constitute the most important parameters that determine the catalytic activity. In fact, in search for high-performance metal-free electrocatalysts, heteroatom-doped carbons have emerged as key materials in ORR catalysis.^617^ Regarding oxygen functional groups, they are naturally formed on the carbon surface when exposed to the atmosphere and they can also be incorporated into the carbon framework through oxidation treatments. Although these groups do not seem to significantly contribute to ORR activity, their presence can enhance the catalytic activity of other heteroatoms through cooperative effects.^617,681^ Undoubtedly, a remarkable breakthrough started in 2009 when Dai and co-workers^682^ demonstrated that incorporation of nitrogen heteroatoms into the carbon lattice of vertically aligned CNTs significantly improved the ORR activity compared to that of conventional Pt/C catalysts in alkaline electrolytes, and with high tolerance to CO poisoning and methanol crossover. Following this discovery, other publications appeared in which various nitrogen-doped carbon structures were synthesized using different nitrogen precursors and doping methodologies, with improved catalytic activity.^683–687^ The preparation of N-doped carbons usually involves strategies such as a CVD synthesis,^688^ growing polymers from nitrogen-containing conjugated monomers, (polypyrrole or polyaniline are very often used), either alone or combined with carbon materials and further carbonization^689^ and pyrolysis of N-containing compounds such as ionic liquids and nucleobases,^690^ that can be done with or without templates. The use of templates^688,691,692^ permits a more precise control of the structural properties of the final material, further optimizing its electrocatalytic properties.

Due to the remarkable activity found on N-doped carbon, many research groups addressed this particular aspect of the ORR. The local structural modifications and charge redistribution caused by the electron-withdrawing effect of nitrogen heteroatoms, as well as the changes in the electronic structure of the carbon material were found as facilitating O_2_ adsorption in a terminal or bridging binding modes, enhancing a charge transfer, and promoting oxygen molecule dissociation while enabling easy desorption of reaction products.^480,617,693–695^ These effects have been corroborated through both experimental results and density functional theory (DFT) calculations, particularly when nitrogen was incorporated near the edges of the carbon framework.^237^ N-doped carbon materials usually contain different nitrogen configurations, and it makes it challenging to assess the precise contribution of each functional group to the ORR catalytic activity. In addition to the intrinsic catalytic activity induced by the N-groups, the other factors already discussed, such as porosity, curvature and defects, have to be considered since they also influence the overall ORR activity.

Although somewhat contradictory results are still under discussion, especially regarding the contributions of specific nitrogen species (such as pyridinic, pyridonic, pyrrolic, and quaternary (graphitic) nitrogen), recent experimental and theoretical studies have provided valuable insights to unravel the role of the nitrogen groups.^617,689,696^ Among these nitrogen species, an important number of researchers observed a direct correlation between the ORR activity and the number of pyridinic N sites at the armchair edges of carbon layers.^697–699^ Recent studies concluded that dioxygen preferentially adsorbs and reduces at carbon atoms adjacent to pyridinic N species, which act as Lewis basic sites^694^ and that the optimization of the heteroatom content is essential to achieve the best performance.^700^ It has also been demonstrated that the pyridine groups located at zigzag positions generate a lower catalytic activity than pristine carbon materials do, highlighting the crucial role of the location and configuration of the N functional groups on the carbon surface.^237,701^ Experimental studies and DFT calculations suggest that the catalytic activity of pyridine-containing carbon materials originates from the formation of pyridonic-like nitrogen (N–C–O) at armchair positions as an intermediate in the early stages of the ORR.^237,694,701–703^ These species were also detected in post-ORR materials.^694,702,704^ Based on these results, either pyridinic groups or pyridonic species, formed through the contact of the N-doped carbon material with the electrolyte at an open circuit potential or when applying a potential, as previously observed,^702^ are active sites for the reaction or that pyridonic species are intermediates of the reaction.^705^ Figure 22A presents XPS results for carbons before and after the ORR and OER and the schemes proposing the formation of −OH intermediates. The shifts in the binding energies of the N-species showed that these intermediates induced the conversion of pyridine into pyridone groups. Consequently, the precise role of pyridine and pyridone-type species in enhancing the ORR activity of carbon materials remains a topic of discussion and the elucidation of this issue requires the use of *in situ* or *operando* techniques that may provide a direct observation of the dynamics of the functional groups responsible for the ORR.

**Figure 22:**
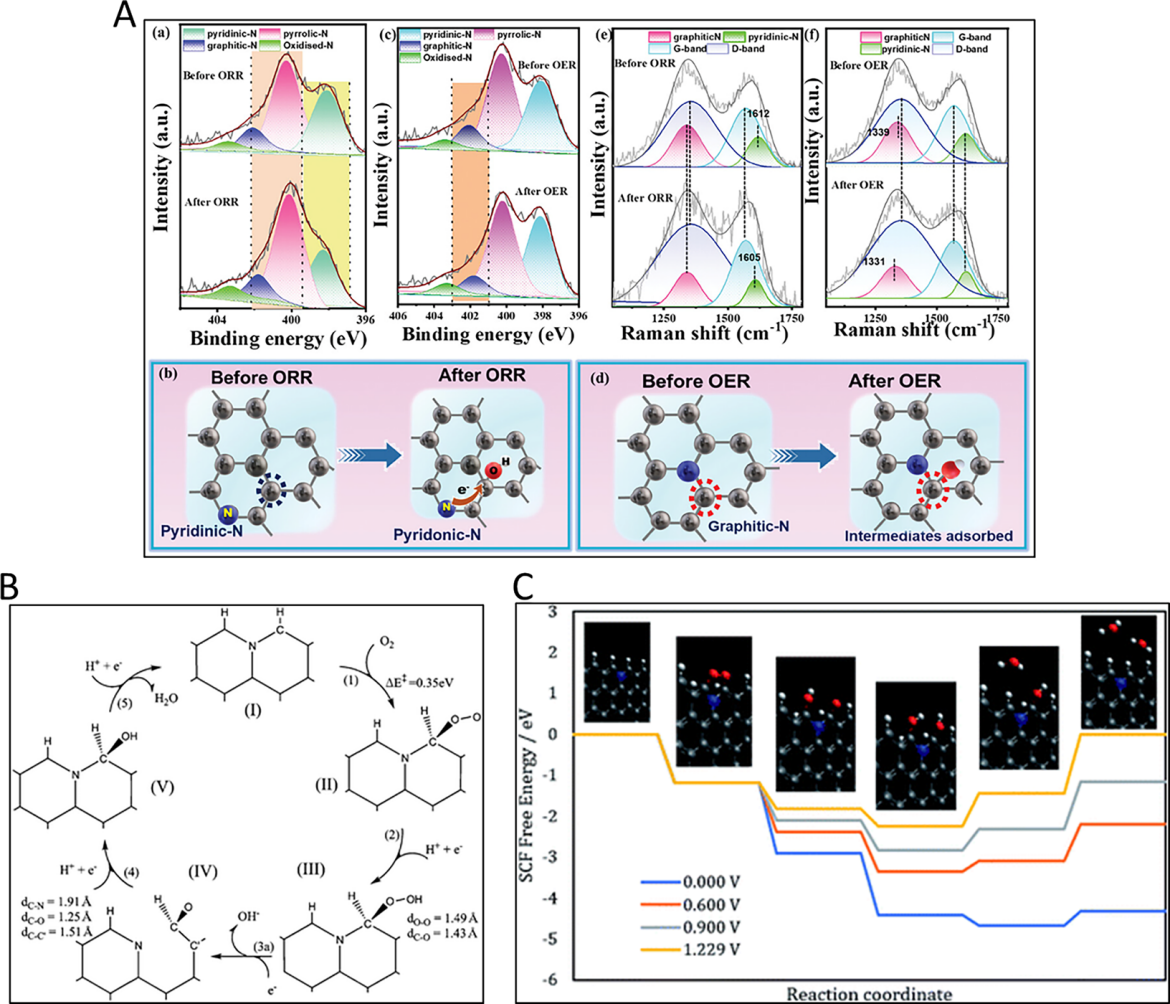
A) (a) Deconvoluted N 1s XPS spectra of Trz-1050 before and after ORR; (b) schematic of formation pyridonic N on Trz-1050 after ORR; (c) Deconvoluted N 1s XPS spectra of Trz-1050 before and after OER; (d) schematic of attachment of intermediates on Trz-1050 during OER; Comparative deconvoluted Raman spectra of Trz-1050 before and after the (e) ORR; (f) OER. Reproduced with permission from ref.^705^ Copyright 2025, Wiley; B) Proposed ORR catalytic cycle for edge-type quaternary structure via C–O–O. Reproduced with permission from ref.^709^ Copyright 2011, The Royal Society of Chemistry; C) SCF energy diagram and proposed mechanism for ORR of edge-type quaternary nitrogen via C–O–O–C. White represents hydrogen, gray represents carbon, blue represents nitrogen and red represents oxygen. Reproduced with permission from ref.^237^ Copyright 2019, The Royal Society of Chemistry.

Pyrrolic N groups are not frequently considered as active sites in the ORR because of their low stability, as they can be converted into pyridones in oxygen-containing environments.^706^ However, there are studies that propose these species as enhancing the catalytic activity and selectivity in the ORR.^679,685^ This enhancement seems to be more important when the pyrrolic groups are present together with pyridinic species.^707,708^ Despite this, due to the challenges in accurately distinguishing pyrrolic N from other similar groups like pyridones and their high reactivity in the presence of oxygen, it is likely that pyrrolic N does not have a relevant contribution as active sites. Moreover, it has to be noted that the adsorption of −OH intermediates on the neighboring carbon atom in pyridinic or graphitic species, is reflected by an XPS peak at similar binding energies as pyrroles,^702,705^ and it can be wrongly identified as an increase in the amount of these pyrrole species.

Other researchers proposed that edge-type quaternary (graphitic) nitrogen sites are the most favorable for generating the most active sites for the ORR, particularly those in zigzag configurations, which are highly selective for the four-electron reduction pathway.^237,701,709–712^ Pyridonic-like nitrogen (N–C–O) has also been identified in post-ORR materials^702^ and its formation is predicted from computational studies.^713^ As observed in the case of the pyridinic-type species, the formation of N–C–O species can be responsible for determining the structure of the active site, provoking the deactivation of the catalyst (because of the strong stability of these species) or just being an intermediate of the reaction. This is an open research topic that still has to be analyzed in detail. Additionally, a synergistic effect between the pyridinic and edge-type graphitic N species seems to be responsible for an increase in the ORR activity.^699,714^ Some studies suggested that while pyridinic N influences the onset potential, graphitic N species enhance the limiting current density of the ORR.^715^ Other authors proposed that the pyridinic sites are responsible for the ORR while the graphitic N species generate sites that are very active toward oxygen evolution reaction (OER).^705^ Nonetheless, these findings still remain inconclusive due to the many factors affecting the ORR catalytic activity of porous carbons and due to the difficulty in the selective synthesis of specific functionalities or defects. The lack of experimental evidence under *operando* conditions that may unravel the role of the N-functional groups is a very important issue and this challenge needs to be addressed in the near future. In fact, few studies report the use of *operando* techniques to follow the reaction mechanism.^679,705^ For example, Bai et al. used^679^ a total reflectance surface-enhanced infrared absorption spectroscopy (ATR-SEIRAS) to follow changes in vibrational bands associated with oxygen–hydrogen species that can be related to the catalytic activity of the ORR active sites. Since XPS is a powerful technique to identify N species and can provide information on the changes occurring after the reaction, the application of this technique under *operando* conditions could be an important outcome to further explore this issue. Another important consideration is that some catalysts labeled as “metal-free” might exhibit a high ORR activity due to trace metal residues from their synthesis or modification processes. Therefore, significant efforts must be made to rigorously confirm the absence of metallic impurities.^696,716^

The effect of pH on the activity of nitrogen-containing sites and on the stability of intermediate species is also a crucial factor. As explained previously, there are studies that indicate that edge-type graphitic N species and highly graphitized carbon structures exhibit a high ORR activity in alkaline conditions.^717^ This is attributed to mechanistic changes that promote oxygen chemisorption via a bridging binding mode, facilitating a product release.^237^ However, pyridinic species are also considered responsible for the observed catalytic activity in the alkaline conditions. In acidic environments, pyridinic N sites are associated with more positive ORR onset potentials,^694,717^ although other studies relate the observed catalytic activity in acidic conditions to graphitic-type nitrogen at zigzag edge.^711^ Thus, this is still a contradictory research topic that seems to be very much dependent on the different properties of the carbon materials analyzed. Nevertheless, a common finding of the published research is that the catalytic activity decreases with a decrease in the pH and this seems to be a consequence of the formation of the protonated form in the case of the pyridinic species.^717^ In the case of the edge-type graphitic nitrogen species, the strong stabilization of protonated intermediates may explain the observed differences in the ORR onset potentials between acidic and alkaline solutions (Figure 23A).^634,717^ Computational studies performed with the pyridinic and graphitic functional groups showed that the active sites switch from the pyridinic (being the most active in acidic conditions) to graphitic ones at high pH, with −OH attached to a neighboring carbon atom.^713^ A recent study concluded that pentagon rich-caged carbon materials (derived from N-doped carbon by a heat treatment up to 1100 °C) are responsible for the high activity found in acidic conditions, which was much higher than that on a pristine pyridinic group-containing carbon material.^678^ Similar conclusions were reached by Bai et al.^679^ In an interesting study, the authors proposed a unified pH-performance in the ORR of nitrogen doped carbon materials prepared by a heat treatment up to 1150 °C (Figure 23B). In both studies, the authors assigned this high activity to pentagon-like defects and, more specifically, to 1,3-cyclopentadiene-like defects.^679^ However, after annealing to 1100 °C, the carbon material still may contain N species, as detected from XPS, with graphitic N as the predominant ones,^678^ and known as having the high catalytic activity. Moreover, the catalytic activity was similar to that measured on graphitic N containing carbon.^634^ The existence of these groups upon heat treatment and the conversion of pyridinic groups into graphitic N is well-known.^712^ In fact, this experimental strategy is used to prepare N-doped materials with a selective doping with this type of species.^237,712^ Additionally, the removal of the N functional groups requires temperatures above 1600 °C, as demonstrated from high temperature vacuum TPD.^126^ The graphitic N species are the most thermally stable and they were indicated in many studies as the most active in the ORR. It cannot be discarded that traces of highly active sites generated by N functionalities govern the observed catalytic activity. The most probable is that the observed high activity could be due to the combination of the pentagon-like structure (or other type of defects) and the presence of the graphitic N species, thus reinforcing the need for engineering at the nanoscale the structure of the active sites. Overall, these findings highlight the critical role of nitrogen species in ORR electrocatalysis, though further research is necessary to fully understand their specific contributions.

**Figure 23:**
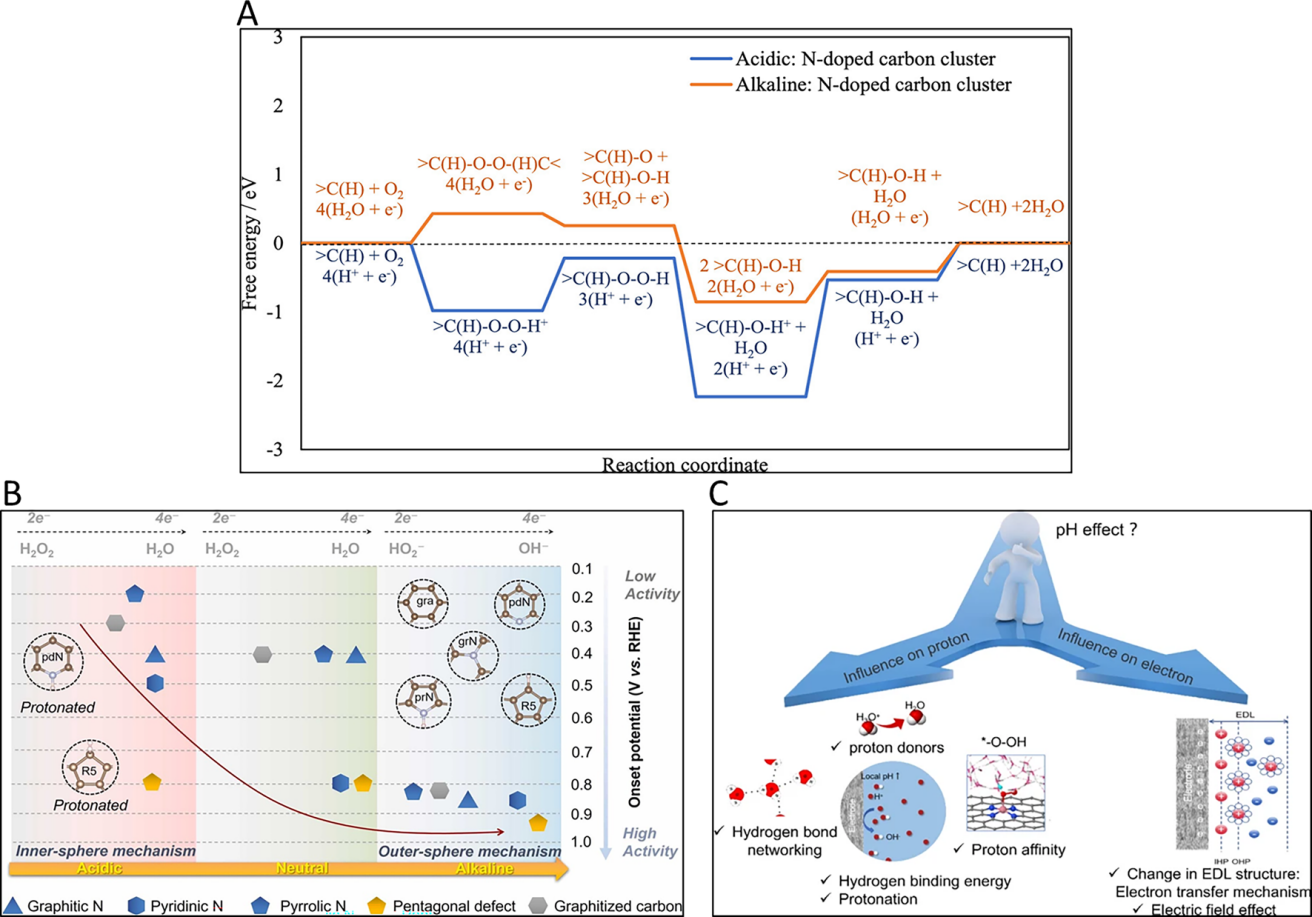
A) Free energy diagram for the ORR of N-doped carbon flakes in alkaline (orange) and acidic (blue) environments calculated for 1.23 V NHE. Reproduced with permission from ref.^634^ Copyright 2020, American Chemical Society; B) Proposed unified pH-performance diagram for Nitrogen-doped carbon materials (NCMs) and proposed 1,3-cyclopentadiene-like defects (i.e., the edge pentagonal defects) as main pH-universal active sites. Reproduced with permission from ref.^679^ Copyright 2025, Elsevier; C) Scheme that summarizes the different effects of pH in proton-coupled electron transfer reactions. Reproduced with permission from ref.^619^ Copyright 2024, Springer.

Other heteroatom-containing carbons, such as those doped with boron, sulfur and phosphorus, have also been reported as catalysts for the ORR. However, the literature on this type of carbon materials is much less abundant compared to that on N-doped carbons. These heteroatoms have lower (or similar) electronegativity than carbon atoms and lower compared to that of nitrogen. This means that a different electron arrangement will occur with respect to N-doped carbon atoms. For example, in the case of B-doped carbon, an improvement in the catalytic activity is related to interactions between electron deficient B atoms and dioxygen^595,599^ causing the chemisorption on this atom instead of on the neighboring carbon atoms, as it occurs in N-doped carbons. An electron transfer through B sites can also be effective in producing the high ORR activity, similar to that of Pt containing catalysts.^93,220^ Phosphorus-doped carbon materials can induce defect generation and improve electron donor characteristics favoring the ORR.^718,719^ Some studies propose that S-doping, especially when forming oxidized S at the edges of graphene layers, is beneficial to increase the catalytic activity because of electronic structure changes that increase a spin density.^720,721^ The formation of defects and local structural changes seems to be the main factors that explain the improvement in the catalytic activity of these heteroatom-doped carbons, although the N-sites seem to be the most active.^722^ Taking into account the structural and electronic changes induced by B, S, and P heteroatoms and the high catalytic activity of N-doped carbons, the research on codoping or multidoping with the different combinations of N and another heteroatom^723^ can be a possibility to improve and solve the problems of N-doped carbon materials, such as the low activity in the ORR in acidic electrolytes, or to lead to a targeted development of multifunctional catalysts. For example, remarkable results have been found for N and P codoped porous carbon with high activity to the ORR and HER^724^ and for the ORR and OER.^725^ Interestingly, N–B and N–P doping produced ORR catalysts with a good performance in both alkaline^723,726^ and acidic electrolytes^727^ and tridoping with N, P, and S has also led to the high activities in alkaline and acidic conditions.^728^ The codoping of carbon materials is a topic that requires further research in order to optimize the structure of the catalytic sites to obtain high activity catalysts with high stability that can be applied to various electrocatalytic reactions in different electrolytes.

##### 4.4.2.5 Other Factors Affecting the ORR

To conclude this section on ORR electrocatalysis, other factors should be mentioned that might constitute specific reviews by themselves and that must be considered to achieve a complete view of the electrocatalytic properties on porous carbon materials. First, and to put in context the factors that will be discussed shortly, we have to consider that under the electrochemical reaction conditions, the electrodes are polarized. For example, in the case of the ORR, the electrode is positively polarized considering the usual open circuit potential of the electrode. This is important to understand the effect of the composition of the electrolyte, among others. The first important factor to be analyzed is the effect of pH in electrocatalytic reactions involving a proton transfer. Although previously we have just mainly focused on how this parameter affects the chemical nature of the N-species, the pH has a very important influence on a mass transport, the extent of oxygen adsorption, EDLC composition and structure and chemical interactions between species in the solution and the surface of the carbon material. Some of these factors have been covered in an interesting review by Liu et al.^619^ Figure 22C presents a scheme that summarizes the different effects of pH in proton coupled electron transfer reactions. The proton-involving reactions produce local changes in the pH, which can be more important in porous carbons, thus resulting in changes in the reaction rate and in the selectivity. The local change in pH can have an effect on the intermediates binding energy and can modify the hydrogen bonding structure in water-based electrolytes, thus affecting the local concentration of ionic species. The electron transfer mechanism is also affected by the pH since the mechanism of the reaction can change from an inner sphere to an outer sphere electron transfer mechanism, and became determined by the chemical nature of the active site at the specific pH conditions and the electrode potential. The alkaline pH was found important, by molecular simulations and experiments, to open the pore sizes of oxygen rich carbon via dissociation of oxygen groups.^648^ That “opening” significantly enhanced the amount of oxygen adsorbed, compared to that in water, and thus increased the efficiency of the ORR process. Consequently, a detailed analysis of the chemical speciation of the active site and of the surrounding atoms (i.e., Pourbaix diagram determination) is necessary to understand the activity.

An electric field is another important factor that affects the composition of the electrolyte/electrode interface, the structure of the double layer (which should be important in porous carbons) and hydrogen bonding structure in a water-based electrolyte since it will be affected by an ion concentration. The electric field is responsible for electrostriction, which, in general corresponds to the contraction of a material under the effect of an electric field.^729^ It is well-known how the ions in a solution generate an electric field that produces changes in the volume of the solution that, in the case of water solutions, is a consequence of the change in the hydrogen bonding network. The electric field generated during the polarization of the electrodes may produce marked changes in the water-based electrolyte solutions due to the generated local electrostriction pressure, leading to phase transitions nearby the electrode surface^730,731^ and changes in physical properties of the electrolyte in nanopores.^732,733^ These phenomena also occur in water-based electrolytes when confined in nanopores and may affect the mass transfer (ion and gas molecules diffusion). This means that a detailed analysis of the structure of the electrolyte when confined in pores and the implications of this issue on the electrocatalytic activity is necessary to fully understand the effect of the different parameters already described. This detailed analysis should be carried out through both computational and experimental studies. Computational studies at the level of detail necessary to take into account the effect of the electric field and electrode polarization, which will have a strong influence on an electrolyte dielectric constant, and solvation effects considering explicit solvent interactions, require a high computational cost^630^ and it is difficult to achieve considering that the system under analysis contains gas, liquid and solid phases.

Regarding experimental work, the use of *in operando* techniques is mandatory to gain a deeper understanding of the reaction mechanism, but the system under study is complex and the results for real catalysts are still scarce for metal-free porous carbon electrocatalysts. From an experimental perspective, most of the studies in the field focus on the synthesis, characterization and evaluation of electrocatalysts in three electrode cells, which is necessary to assess the intrinsic catalytic activity. However, studies on the application of the electrocatalysts in fuel cells are not often found, which indicates that either the preparation of membrane electrode assemblies employing adequate alkaline membranes is difficult compared to acidic membranes (a limiting issue) or the electrocatalyst is not applicable under more realistic conditions. It is also expected that, in the near future, Machine Learning will help deepen our understanding of the reaction mechanism in electrocatalysis since it integrates both experimental and computational models.^734^

##### 4.4.2.6 CO_2_ Electroreduction

Although not much explored, the electrochemical CO2RR using porous carbon is a topic under development with two main challenges associated with (i) the low production rates compared to metal-based electrocatalysts, and (ii) the fact that selectivity seems to be dominated by the formation of CO and CH_4_, rather than to methanol, formic acid and other C2 compounds commonly reported for metal electrocatalysts.

Most of the studies on the use of porous carbons for this reaction are focused on introducing modifications to the composition of a carbon material, and the improved performance is mainly attributed to the effects of heteroatoms on the electronic properties of carbon, and/or the creation of new electrocatalytic active sites.^468,607–615^ There is a general agreement on the beneficial effect of the incorporation of N-moieties to carbon materials, to achieve high CO_2_ conversions and faradaic efficiencies due to the improved electronic properties of the N-doped carbon.^609,735,736^ Li et al.^736^ tested carbons with introduced nitrogen in various configurations and found the positively charged sites introduced to the carbons matrix, pyridinic and quaternary and also N-oxides outside the ring (C N+O−) were active in CO2RR. They also established a linear correlation between the CO FE and the amount of the pyridinic-N, indicating the predominant role of these species in the reduction process (Figure 24A).

**Figure 24:**
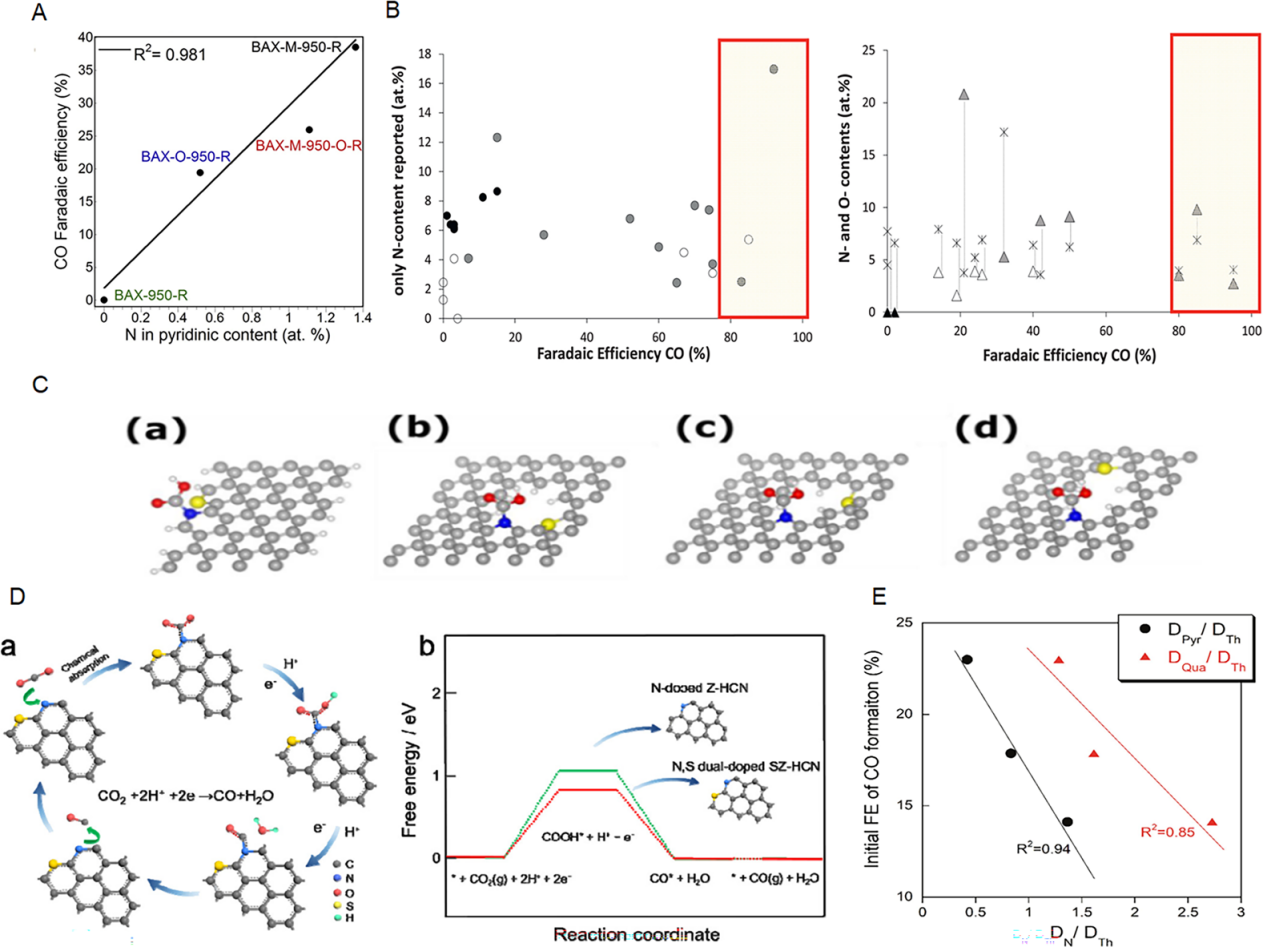
A) The Faradaic efficiency for CO formation (1 h) as a function of the content of pyridinic N for the reduction pretreated porous carbons. Reproduced with permission from ref.^736^ Copyright 2017. Elsevier; B) Correlation between faradaic efficiency of CO and the (left) nitrogen content and (right) nitrogen + oxygen content. Circles (only N-data is reported); triangles (N- and O- are reported); squares (N- or O-content below 3 at. % as representative of low functionalization). Color of symbols refers to the applied potential: black symbols (−0.60 V vs RHE); gray symbols (−0.67 V vs RHE), white symbols (−0.90 V). Red rectangles highlight FE over 80%. Adapted with permission from ref.^287^ Copyright 2023, Elsevier; C) a) Proposed mechanism for the nitrogen-doped catalytic site. (b) Calculated free energy diagram of CO_2_ reduction to CO on N-doped Z-HCN and N,S dual-doped gSZ-HCN. Reproduced with permission from ref.^738^ Copyright 2020, Elsevier; D) *COOH adsorption configuration on S+Pyri-N: (a) S+Edge_Pyri-N; (b) S4+Hole_Pyri-N; (c) S7+Hole_Pyri-N, and (d) S10+Hole_Pyri-N. The black, blue, red, yellow, and white spheres represent carbon, nitrogen, oxygen, sulfur, and hydrogen atoms, respectively. Adopted with permission from ref.^106^ Copyright 2019, Elsevier; E) Dependence of the Initial Faradaic efficiency for CO reduction on the dispersion of nitrogen to thiophenic-S group ratio. (D_N_ represents either D_Pyr_ or D_Qu_. Reproduced with permission from ref.^739^ Copyright 2021, Elsevier.

Li et al. also found that the acidic surface of the porous carbons increased the overpotentials required for obtaining a high faradaic efficiency, and linked this observation to an increased competitive hydrogen evolution reaction.^610^ Unfortunately, nitrogen groups enhancing CO_2_ electroreduction can get easily oxidized during this process, negatively affecting the efficiency of the catalysts. To address this problem, Li et al.^737^ used carbons rich in pyrazinic nitrogen with total surface nitrogen content between 2 and 5.8 at. % as the CO_2_ reduction electrocatalysts. Besides, carbons tested contained pyrroles/pyridines and quaternary nitrogen. The results indicated that pyrazinic N protected the pyridinic nitrogen—the main catalytic center for CO_2_ reduction—from oxidation. The authors also suggested that quaternary nitrogen might be active in CH_4_ formation, since its amount on the surface during the CO_2_ reduction process decreased. Based on the detection of C_3_ products (not commonly reported in the literature), the authors also proposed that the structural uniqueness of the pyrazinic N in the pore space might promote the C–C bond formation.

As mentioned in the previous sections, N-doped carbons typically display high amounts of oxygen groups. This fact is often overlooked when discussing the electrocatalytic activity of N-doped carbons. This has been recently addressed by Gomis-Berenguer et al.^287^ and it is illustrated in Figure 24B. By collecting own and other’s groups data, the study showed the correlations between the electrocatalytic activity of N-doped carbons with the nitrogen and oxygen content of the carbon electrodes. The authors pointed out the complexity of the data interpretation given the disparity of textural properties and chemical composition, and recommended reporting disaggregated data of porosity and composition and including a complete characterization of the nature of the surface groups (beyond a heteroatom content) by combining various techniques to better describe the materials. Unfortunately, very often incomplete data sets (composition and porosity) are reported, with discussions lacking adequate correlations between the electrocatalytic performance and the relevant physicochemical parameters of carbon electrodes (Figure 24B).

Dong et al.^47^ reported the influence of topological defects in carbon networks on CO_2_ electroreduction due to the modification of the local electronic redistribution of the carbon matrix. The authors reported the preparation of porous carbons with a varied density of topological defects, pyrrolic and pyridonic sites by NH_3_ thermal treatment of the material, and observed the key role of edge sites involving 5- and 8-member ring defects formed by removing pyridinic N and pyrrolic N species in CO2RR (pentagon edge defects are dominant, favoring the formation of CO over the competitive HER).

Recent studies have shown the benefits of dual codoping with various heteroatoms.^79,613,740^ As an example, Jia et al.^613^ described the good electrocatalytic performance due to N- and B-codoping in a carbon material with a 3D hierarchical porous structure. The authors reported the key role of codoping, with B-sites enhancing the adsorption of CO_2_ and the conversion to COOH* intermediates into CO*, and pyridinic N sites promoting the first single electron transfer reactions (promoting H* transfer and COOH* generation). Density functional theory calculations have demonstrated that P–C sites are more reactive than P–O ones for the formation of COOH*, a key intermediate in the electrochemical CO2RR.^740^

Both S- and N-codoping have also been explored as means to increase the performance of metal free carbons as CO_2_ electrocatalysts. Li et al.^610^ compared the performance of S-doped and S- and N-codoped polymer derived porous carbons. Even though S-doping carbon exhibited some activity, linked to the modulation of the charge and formation of sites for CO_2_^•–^ intermediates adsorption, it was much less active than carbons with nitrogen and sulfur coexisting in its matrix. The FE of the latter for CO formation was four times higher than that of the S-doped catalysts. The authors also observed easy oxidation of thiophenic S to sulfones and sulfoxides, decreasing the activity of the S-doped carbons. Moreover, that carbon had also much lower porosity than S- and N-doped ones and this feature was also suggested as negatively influencing the reduction process.

Following this line of research, Wen and co-workers achieved the FE for CO formation of 85.4% on nitrogen and sulfur-doped carbons.^740^ Li et al. prepared N, S-dual doped porous carbon by a one-step pyrolysis of N-containing polymer and S powder^738^ and their CO FE was ∼93% with partial current density of 5.2 mA cm^–2^ at the overpotential of 0.490 V (Figure 24C). Here, also, S- and N-codoped carbon performed much better than its solely S-containing counterpart. It had more defects and more developed porosity. DFT calculations revealed that combining N and S doping decreased the Δ*G* value for the formation of a COOH* intermediate (Figure 24D). A similar performance and DFT results were reported by Lin and co-workers.^741^ Pan et al.,^106^ through experiments and calculations, concluded that S- and N-codoping led to an increase in a defect population, and incorporating S in thiophenic configurations led to the generation of more active pyridinic N groups for CO_2_ reduction (Figure 24D). Their results suggested that S-doping enhancement depended markedly on the relative distance between pyridinic N and S atoms, and an increase in the distance between S atom and the pyridinic N group was predicted to increase the catalytic activity. This effect was explained by the increased spin density of N–C due to the effect of sulfur, which was suggested as facilitating an electron transfer and adsorption of COOH* in the CO_2_ reduction process.^742^ This was reflected in an increase in reaction rates at low overpotentials on the N–C catalytic sites. Moreover, Pan et al. found that incorporating S significantly enhanced the selectivity of pyridinic N for CO formation. Li and Bandosz^739^ also studied this effect, reporting the dependence of the initial Faradaic efficiency for CO reduction on the ratio of the dispersion of nitrogen to thiophenic-S group (Figure 24E). Those ratios have been established as reflecting the distance between either pyridinic or quaternary nitrogen and thiophenic sulfur. The lower the density, the higher the dispersion of the specific groups on the surface. Once again, in these complex configurations, the results suggested that the pyridinic groups were more active than quaternary nitrogen. More sulfur on the surface was not associated with the higher contribution of pyridinic nitrogen, as was also reported by Pan and co-workers.^106^

Another challenge in porous electrocatalysts is to isolate the effects of porosity from those of surface chemistry. The synthesis of carbons with controlled pore architectures and functionalization very often does not allow obtaining a pertinent undoped material with a similar porosity, or nonporous doped materials with an identical composition.

The role of the porosity of carbon materials in the CO2RR has been less addressed. The size of meso-/macropores seems to be important to boost the selectivity of CO over H_2_ (due to confinement effects),^615^ while the geometry of carbon nanopores can influence the selectivity toward C2 compounds, due to steric hindrances to form bulky intermediates.^611^ Surprisingly, despite the well-known affinity of the CO_2_ molecules for carbon materials with a well-developed microporosity, the effect of micropores on the catalytic activity of porous carbons for the electrochemical CO2RR has been less investigated and often neglected.^287,610,614,615,736,743^ As an example, Song et al. reported the enhanced ethanol production in the CO2RR using N-doped porous carbons. The high activity and selectivity toward ethanol was attributed to the desolvation effects in medium sized micropores, which would facilitate the coupling of the C1 intermediates to form ethanol, and to the fast charge transfer at pyridinic and pyrrolic sites.^614^ This demonstrates the importance of analyzing the electrocatalytic activity considering the contributions of both factors simultaneously. Li et al.^610^ proposed that the methane formation on porous carbons might not be electrochemically driven but it is rather governed by the adsorption of CO and H_2_ in small pores, where high pressure and a strong adsorption potential lead to bond splitting and the formation of CH_4_ in the chain of reactions analogous to those of a Fischer–Tropsch catalytic process. Although this hypothesis has not been yet fully experimentally supported, methane formation is not commonly reported on non-porous nanocarbon electrocatalysts.

Yet, the area still needs a considerable development, and further research should be conducted in this direction to clarify the effect of the nanopore confinement and specific surface functionalization in pore spaces of carbon materials.

#### 4.4.3 Photocatalysis

In recent decades, research on photocatalytic applications and the development of efficient photocatalysts capable of harnessing solar photons to drive key chemical reactions in various fields (environmental pollution control—water treatment, self-cleaning surfaces, air purification, production of energy vectors—hydrogen and oxygen from the photochemical water splitting, fuels from the photorreduction of CO_2_, energy production in solar cells) have become a compelling strategy to face current challenges related to an energy demand and environmental protection. Despite the potential of most photocatalytic reactions, their wide scale deployment remains to be proven, owing to various unresolved technological challenges related to the low efficiency of most photocatalysts (stability, mass transfer, quantum efficiency) and the inefficient design of catalytic photoreactors.

Photocatalytic reactions in heterogeneous phase are dominated by wide band gap semiconductors as photoactive materials, and even though most carbon materials are strong light absorbing solids, they have found their way to contribute to this field. While the zero-bandgap nature of graphene limits its application in photocatalysis, defective forms of carbon offer interesting alternatives in the field. Despite their relatively lower photocatalytic activity compared to many benchmark semiconductors, carbon photocatalysts have proven advantages in terms of high photostability and wide spectrum response.^744^

In this context, extensive work has been carried out on exploring the photocatalytic activity of porous carbon/semiconductor mixtures for many applications. The reader is referred to the early works of Hermann and co-workers on TiO_2_/activated carbon composites,^745–747^ Faria and co-workers on TiO_2_/carbon nanotubes composites^14^ and Inagaki et al. on TiO_2_/carbon coatings^748^ for the degradation of water pollutants, as well as recent reviews covering a wide variety of semiconductors and carbon materials.^749–752^ All these studies attribute the role of the carbon materials to several factors depending on their physicochemical characteristics. These cover the improved spectral response of the semiconductor/carbon composites, the enhanced mass transfer of the overall photocatalytic reaction, the decreased recombination rate of the photogenerated species, or the modification of the electronic band structure of the semiconductor. As examples, the enhanced photoactivity of semiconductor/carbon composites based on carbon materials with high electron mobility is mainly attributed to strong interfacial electronic effects between both phases. For porous carbons, the nanoconfinement in the pores is also important.

More recently, photocatalytic applications based on semiconductor-free carbon materials have also attracted the interest of the scientific community. In 2009, Luo et al. reported the photocatalytic activity of highly defective carbon nanotubes under visible light for the degradation of hydrogen peroxide, which was attributed to the presence of topological defects (vacancies).^753^ In 2010, Velasco et al. described the photocatalytic activity of porous carbons under UV–visible light for the degradation of phenol from solution,^754^ attributing the phenomenon to the confinement effect in the porosity of the carbon material. This work has opened interesting perspectives in the regeneration of spent carbon adsorbents.^755,756^

In this section we limit the scope of the discussion to the analysis of the role of the surface chemistry of porous carbons in photocatalytic reactions of strategic interest, either in their role as additives to semiconductors or as self-photocatalysts (metal- and semiconductor-free). We have only focused on relevant works on the topic that analyze the correlations structure–properties–performance, with a focus on the porous carbon component (particularly its surface chemistry). For a detailed analysis of the optical properties of porous carbons and the origin of their photocatalytic activity, the reader can refer to some recent works.^757–761^

##### 4.4.3.1 Photocatalytic Degradation of Pollutants

The degradation of water pollutants using heterogeneous photocatalysis has been intensively studied due to the advantages of high oxidation efficiency and mineralization of the pollutants.^762,763^ Current research efforts are directed to synthesizing stable and efficient photocatalysts that are active under solar light, and optimizing the geometry of photoreactors to optimize degradation rates and performances. As TiO_2_ is the benchmark photocatalyst for water treatment, TiO_2_/carbon composites have also been largely investigated for the photooxidation of pollutants. All types of carbons and morphologies have been studied and applied for the degradation of a large number of water pollutants, with different outcomes depending on many factors such as the characteristics of the composite, the nature of the pollutant, the synthetic route, the illumination conditions, etc. The discussion of this section will focus on illustrating the role of the characteristics of the porous carbon in TiO_2_/carbon composites.

TiO_2_/carbon composites are commonly prepared using porous carbons with large surface areas and pore volumes. In those hybrid photocatalysts, the porous carbon typically facilitates the diffusion and transfer of reactants to active sites, and boosts the interface interaction of the pollutant and the photocatalyst via adsorption. The specific role of the porous carbon component in the activity of semiconductor/carbon photocatalysts includes: the ability to tune the bandgap, improved charge carrier density, improved dispersion of photoactive particles, act as electron sink and photosensitizer, and/or a more efficient charge carrier separation owing to the smaller diffusion length through the pores.^764–767^ All of these are important parameters to improve photocatalytic efficiencies, but the specific role of surface chemistry of porous carbons remains unclear. The narrow microporosity of the carbon material is usually underexploited due to the difficulty of efficiently immobilizing the semiconductor (either by *ex situ* infiltration or *in situ* synthetic techniques) and the lack of control of the surface distribution of TiO_2_ nanoparticles in the small pores.^764–767^ Mesopores and macropores typically allow a better dispersion of the TiO_2_ nanoparticles than the micropores do, and therefore become useful for the degradation of large-molecule pollutants (i.e., dyes) which are preferentially adsorbed in those large pores.^768–770^

Regarding the photocatalytic reaction, although the porous carbons generally enhance the photodegradation apparent rate constants of the composite compared to the bare semiconductor (Figure 25A), surface areas do not seem to be essential to achieve high degradation rates. Several studies have reported that the degradation efficiency follows a dependence on the mass of both phases in the composites, with optimum results for TiO_2_:carbon ratios between 1:1 and 2:1 (Figure 25B,C).^745,771–773^ An example can be found in the studies on the superior photocatalytic activity of titania particles casted on spherical mesoporous carbon particles with a core/shell architecture for the degradation of various pollutants.^770,774^ The carbon nanoparticles displayed a high density of O-groups to favor anchoring TiO_2_ on the surface and the enhanced photocatalytic activity of these materials has been interpreted as a combination of the better light absorption of TiO_2_ due to the mesoporosity of the core/shell composite, and the visible light absorption of the functionalized carbon materials (Figure 25D,E). The charge transfer between porous carbons and TiO_2_ has also been evidenced by spectroscopic and transient photocurrent measurements, and several authors have pointed out the key role of the donor/acceptor character of the functional groups of the carbon in the charge transfer.^750,772,775–777^

**Figure 25:**
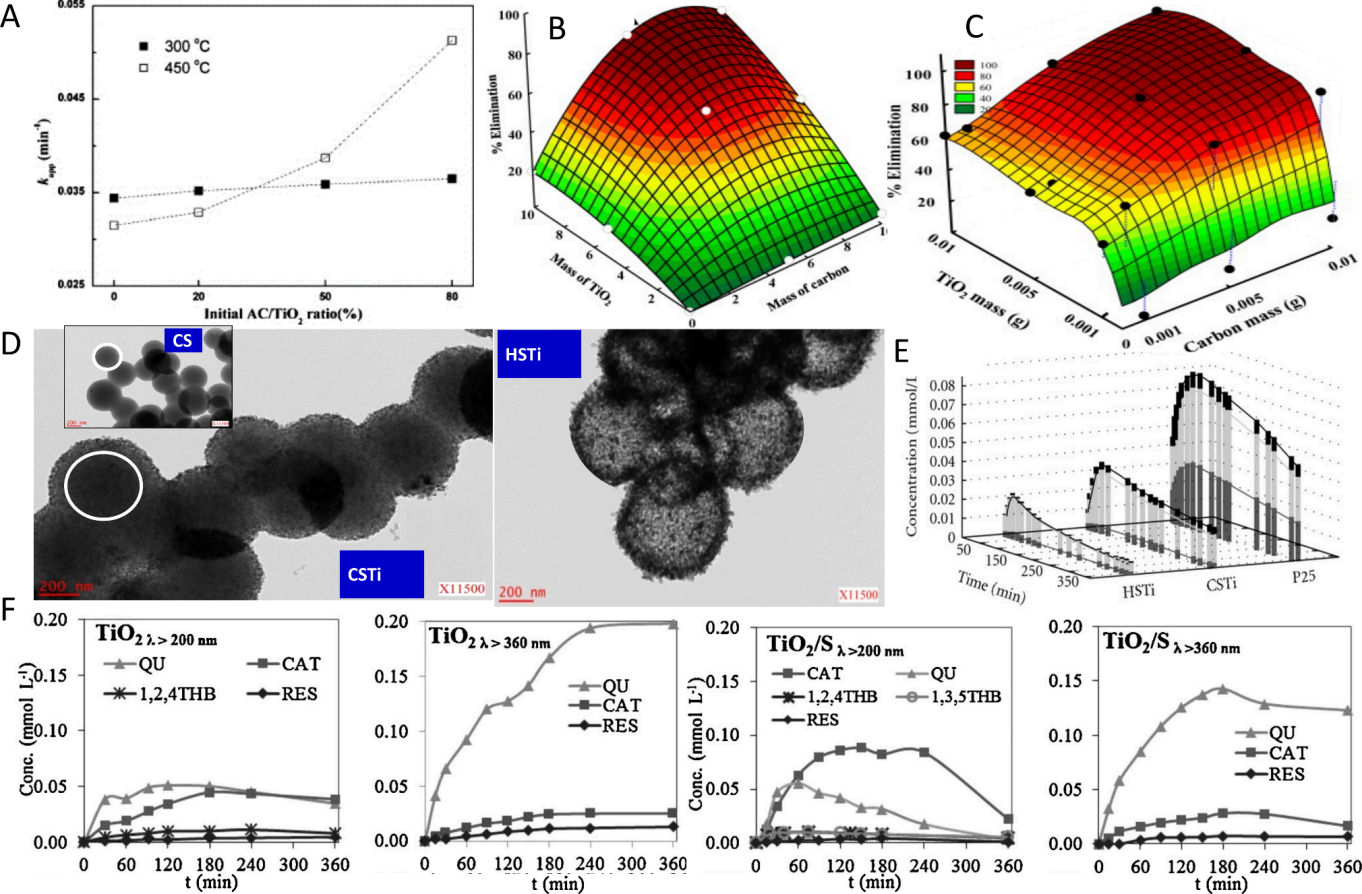
A) Apparent first-order rate constant for the degradation of chromotophe 2R in solution on TiO_2_/activated-carbon composites calcined at 300 and 450 °C. Reproduced with permission from ref.^772^ Copyright 2005, Elsevier; B) Elimination (B) 2,4-D and (C) cytarabine versus the mass of TiO_2_ and activated carbon. Reproduced with permission from ref.^773^ and from ref.^771^ Copyright 2012 and 2011, respectively, Elsevier; D) SEM images and E) evolution of phenol photodegradation intermediates—hydroquinone (dark gray); benzoquinone (light gray), and catechol (black)—in solution on TiO_2_/O-doped mesoporous carbon composite (CSTi) compared to mesoporous TiO_2_ (HSTi) and commercial TiO_2_ (P25). Adapted from ref.^770^ Copyright 2013, the authors under CC-BY 3.0 license; F) Evolution of phenol degradation intermediates upon irradiation at λ > 200 nm and λ > 360 nm of TiO_2_ and TiO_2_/carbon photocatalysts. QU-quinones; BZ-benzoquinone; CAT-catechol, RES-resorcinol; 124THB-1,2,4-trihydroxybenzene; 135THB-1,3,5-trihydroxybenzene. Reproduced from ref.^783^ Copyright 2015, Elsevier.

The presence of the carbon component in TiO_2_/carbon photocatalysts has also been reported to modify the degradation route of several pollutants due to the confinement in the nanopores of the carbon material (Figure 25F).^761,775,778–782^ The preferential regioselective photooxidation of phenolic derivatives through the *ortho*- and *para*-substitution (electrophilic substitution of the aromatic ring) has been reported for TiO_2_/C composites with carbons of basic character.^747,783,784^ This mechanism is more effective for achieving the complete mineralization of the pollutants as it involves a smaller number of subproducts. Such a modification of the photodegradation route has been attributed to the favorable interfacial interactions between the carbon material and the semiconductor, and the hydrophobic/hydrophilic character of the carbon material. Matos et al. reported the good photocatalytic activity of TiO_2_/C systems using hydrophobic carbons, and a detrimental effect for hydrophilic carbons.^745,785,786^

Ocampo-Perez et al. investigated a series of carbon/titania composites, with a focus on the impact of chemical modifications applied to the carbon component on the photocatalytic activity for the degradation of pollutants.^771,773,787^ The authors reported an increase in the photocatalytic conversion for the degradation of cytarabine (molecule of basic character) on all the composites, with the impact being more pronounced in the composites with oxidized carbons.^771^ This behavior was attributed to the high cytarabine adsorption capacity (due to the porosity of the carbon component) in the composites prepared using the carbons of a basic nature, and for the acidic carbons, to specific interactions between the acidic groups of the carbons and the oxygen radical species generated upon irradiation. The authors proposed that carboxylic acid groups present on oxidized porous carbons participate in the additional generation of hydroxyl radicals and in the capture of the photogenerated electrons, thereby boosting the photocatalytic activity of the composites.^771^ This, however, modifies the surface chemistry of the oxidized carbons, thus affecting the reusability of the materials (unfortunately not reported in the study). In contrast, O-containing groups have been reported to decrease the conversion of the photo-oxidation of phenol inside the pores of the porous carbon photocatalyst. In this configuration, the yield of the light conversion was very sensitive to the acidic/basic strength of the O-groups, particularly to the presence of acidic (CO_2_-releasing) groups.^788^

The ability of the semiconductor-free porous carbons to generate hydroxyl and superoxide radicals upon exposure to UV and visible light was later demonstrated by various approaches such as EPR and spin trapping with chemicals (Figure 26A,B).^189,190,789^ The formation of hydroxyl and superoxide radicals upon UV and solar illumination was not observed for all types of carbon materials when exposed to light, pointing out that this is not an intrinsic property of all porous carbons. Most of the carbons generated higher concentrations of hydroxyl radicals than superoxide radicals, and the concentration was higher under UV light than under solar light. This is consistent with the observed enhancement of the photocatalytic activity of TiO_2_/carbon composites under solar light for the degradation of aqueous pollutants.

**Figure 26:**
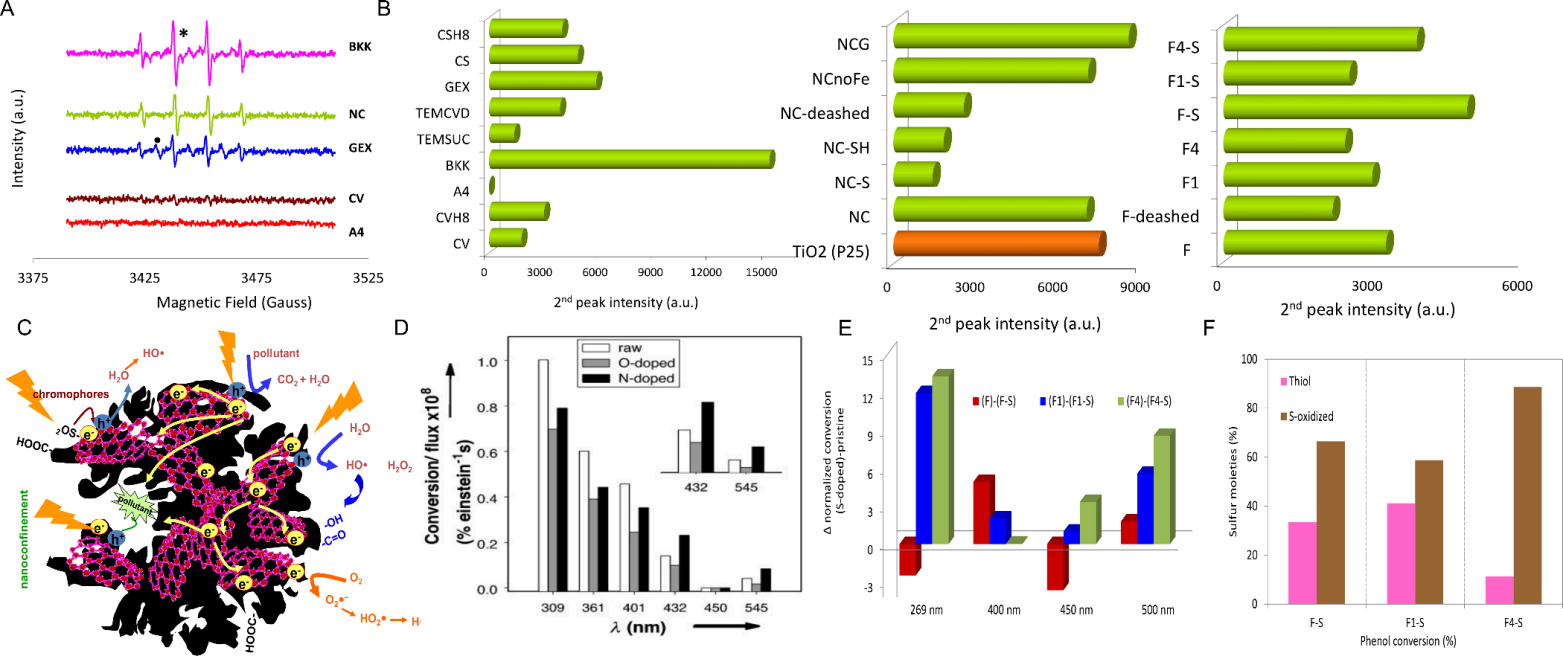
A) Example of the ESR signal corresponding to DMPO–OH adducts and B) quantification of the radical species (second peak intensity in the 1:2:2:1 patterns) on illumination of metal-free and semiconductor-free porous carbons with varied texture, surface chemistry and structure: examples of unfunctionalized carbons (NC, A4, KK, CV, CS, F, TEMCVD, TEMSUC), deashed carbons, S-doped (F–S series), O-doped (CV, NC-SH, NC-S, CS), annealed (CVH8,) and with graphitic carbons (NCG, GEX). Reproduced with permission from ref.^759^ Copyright 2018, Wiley-VCH; C) Schematic representation of the mechanism proposed for the formation and fate of holes and electrons upon irradiation of porous carbons. Reproduced with permission from ref.^761^ Copyright (2016), Elsevier; D) Effect of O- and N-functionalization on phenol conversion values per incident flux upon illumination at different wavelengths using monochromatic light. Reproduced with permission from ref.^798^ Copyright 2014, Wiley-VHC; E) Effect of sulfur on the conversion of phenol, defined as the difference between the normalized conversion in the S-doped and its corresponding as-received carbon, at different wavelengths and F) Effect of sulfur moieties on phenol conversion. Adapted with permission from ref.^759^ Copyright 2018, Wiley-VCH.

The amount of the photogenerated radicals seems to be influenced by the optical properties and the surface chemistry of the carbons, although contradictory effects have been reported. For instance, Velo-Gala et al. observed favorable formation of radical species on carbons with high concentration of surface oxygen (mainly ester and anhydride groups) under visible light irradiation,^789^ whereas Velasco et al.^189,190,788^ observed a different trend with a superior performance for the photooxidation of phenolic compounds in carbons either of low functionalization or with phenolic and quinone-type groups. The light conversion yield decreased with small changes in the acidic/basic strength of the oxygen groups, particularly in the presence of acidic groups. The authors rationalized this finding with the type of O-surface groups, reporting a correlation with the acidic/basic character of the carbons: the more acidic the carbon, the lower the ESR signal (lower amount of radicals), although some soft carbons broke this trend.^189,190^ The analysis of the impact of the structural order (graphitic, graphitized and amorphous carbons) did not show a clear trend, suggesting that the ability to photogenerate radicals is complex and most likely cannot be attributed to one single parameter. One hypothesis is that the presence of different types of intrinsic defects may contribute to the photogeneration of radical species—similarly to what has been proposed for explaining the photocatalytic degradation of pollutants in defective TiO_2_/3D graphene composites.^790^ Unfortunately this was not addressed in those studies.

This distinct photocatalytic activity of carbon materials has been attributed to the nature of the electronic transitions in carbon materials involving sp^2^ carbon domains and chromophoric groups on the surface (Figure 26C).^760,791,792^ Compared to TiO_2_, where both long-lived (Wannier) and short-lived (Frenkel) excitons are formed upon illumination,^793^ short-lived (Frenkel-type) excitons are predominant in porous carbons under UV illumination (involving π–π* and σ–π electronic transitions in zigzag and carbine sites).^760,794,795^ In functionalized carbons, charge transfer excitons activated at longer wavelengths may also be formed by localized electronic states involving heteroatoms, due to the favorable energy difference between the electronic levels.^366^

Other than oxygen, the photocatalytic activity of S- and P-doped carbons has been investigated for the degradation of several pollutants, with clear enhancements in the activity under visible light due to the effect of the heteroatoms.^286,788,796,796–799^ For S-doped carbons, the improved photoactivity has been correlated with the presence of oxidized sulfur chromophores capable of being activated by low energy photons. The higher amounts of oxygen radical species measured in the S-doped carbons and the strong dependence of the photocatalytic activity on the wavelength of the irradiation were demonstrated, with better results in carbons combining a high sulfur content and large average pore sizes (Figure 26E). This improved activity has been linked to the fast charge transfer in S-doped carbons and the stabilization of the photogenerated holes through the formation of radical species. An advanced analysis of the dependence of the photoactivity on the wavelength of the irradiation has shown that thiols and sulfides present a poor photoactivity towards phenol oxidation in the range of 269–500 nm, whereas oxidized forms of sulfur are more photoactive in this range.^286^

The functionalization with halogens has been less investigated, but a recent study reports the synthesis of iodine-doped porous carbon for achieving visible light photodegradation of aqueous pollutants. The authors attributed these features to the I-doping (mainly as I_3_^–^ and I_5_^–^) that would narrow the bandgap and facilitate the photogeneration of charge carriers.^800^

Regarding the wavelength dependence, studies using monochromatic light have shown that carbons with low functionalization and oxidized carbons present a higher activity under UV light, whereas functionalized carbons follow a U-shaped pattern with the wavelength energy, with a minimum photoactivity featuring between 400 and 450 nm.^788,798^ This explains the observations related to the modification of the degradation mechanisms upon the wavelength of the irradiation source,^783^ showing increased photoconversion values and region-selectivity in the oxidation pathway for TiO_2_/carbon catalysts using high energy photons. For O-doped carbons, the fall in photooxidative conversion of phenol has been attributed to the low stabilization of the photogenerated carriers through delocalization in the π-electron density of the carbons owing to the lower electron withdrawal effect induced by the O-moieties (Figure 26D). In contrast, N-doped carbons, decorated mainly with quaternary nitrogen and pyridine-type groups, exhibited an enhanced light absorption in the visible range at wavelengths above 400 nm (redshift in light absorption and increased conversion at 432 and 545 nm). This has been explained owing to a higher electron mobility and the creation of midgap states below the conduction band edge upon the functionalization^798^ due to the electron-donating character of the lone pair of electrons of nitrogen atoms. The use of filters on heteroatom doped carbon textiles also revealed that functionalized materials exhibited the highest photosensitivity upon irradiation at about 500 nm.^799^ For the incorporation of S-groups, an improved photoactivity has been reported at 269 and 500 nm for oxidized forms of sulfur (Figure 26E,F).^286^

The surface chemistry of the carbons also modifies the optical features of the semiconductor/carbon composite. The optical band gap is typically used to screen the ability of photocatalysts to absorb light and convert it to chemical reactions, although it only provides information about the energy needed to form excitons (excitation of an electron from the valence to the conduction band). Unfortunately, the estimation of the electronic band gap (energy needed to split the excitons and create free charge carriers that can trigger a photocatalytic reaction) through photoelectrochemical measurements is scarcely addressed. In hybrid semiconductor/carbon composites, it is important to distinguish between both concepts due to the high binding energies of the excitons in carbon materials.

Most studies report that the optical properties of TiO_2_/carbon composites are not significantly altered upon the incorporation of carbon additives. In most cases, the absorption/diffuse reflectance spectra show the characteristic sharp absorption edge of the semiconductor in the UV (ca. above 400 nm), and a broad background absorption in the visible light region that increases with the amount of the carbon material. Other studies report that carbon materials act as photosensitizers by an electron transfer from the carbon to the semiconductor due to the high density of C=C bonds (i.e., a high electron storage capacity),^777,801,802^ with spectral shifts toward the visible range that depend on the nature of carbon, the amount and method of the composite preparation and the functionalization of the carbon material.^41,771,776,778,796,803,804^ Evaluating the optical bandgap of semiconductor/carbon composites is rather a complex task.^804,805^ When the amount of carbon material is low, it is reasonable to assume that the dominant electronic transitions are those which are characteristic of the semiconductor. However, when interfacial interactions between both phases occur (as in highly functionalized carbons), mixed mechanisms for the electronic transitions should be considered and the application of Tauc graphical representation must be done with care. Gesesse et al. have analyzed the impact of the surface chemistry of porous carbons on the optical response of semiconductors/carbon composites for various types of carbons and semiconductors.^804^ The authors reported spectral shifts in the absorption features of semiconductor/carbon composites, mainly occurring at wavelengths near the edge of the semiconductor (majority transitions) and secondary shifts at long wavelengths corresponding to the visible range (Figure 27A). The dependence of the characteristics of the semiconductor/carbon composites on the synthesis method suggested the existence of intermediate gap states^804^ owing to changes in the density of electronic states on functionalized porous carbons.^798^ The impact of surface gap states introduced in TiO_2_ upon functionalization or the formation of heterojunctions has been widely documented.^806^ In amorphous carbons, chemical functionalization is considered to have a strong effect on the band gap associated with sp^2^/sp^3^ hybridization of carbon atoms, and the creation of defects and midgap states below the conduction band edge.^792^ The latter have been attributed to the presence of chromophoric moieties in the carbon materials involving O-, N-, S-heteroatoms that absorb light in the visible range. The study also highlighted the importance of using a double linear fitting mathematical approach for the calculation of the optical band gap in materials with strong light absorption and several optical features, to avoid the underestimation of the band gap values (Figure 27A). This is critical for materials with strong light absorption (semiconductor/carbon composites), since the band gap values are often used to evaluate the redox potential of the conduction and valence bands of the systems.^807–809^

**Figure 27:**
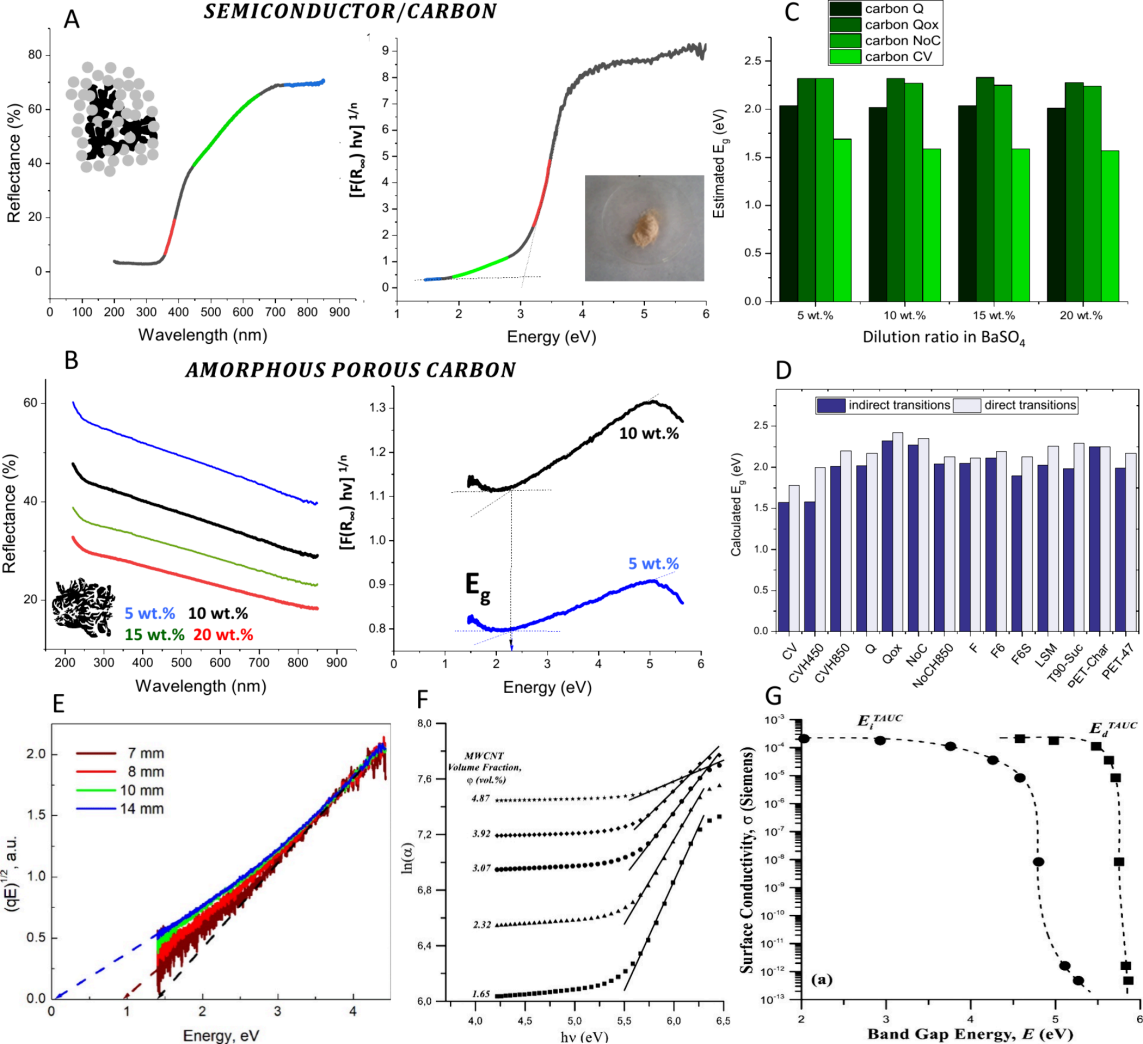
A) Diffuse reflectance spectra and Tauc graphical representation of semiconductor/carbon, B) and amorphous porous carbon photocatalysts. Experimental data (black line); fitted range (red line); baseline fitting (blue line); dotted lines represent the extrapolation using the double linear fitting approach to evaluate the optical band gap; C) Optical band gap values of selected carbons calculated using different dilution ratio; D) Optical band gap values of porous carbons with varied composition (O-, S-functionalized, annealed) and structural order, considering both direct and indirect transitions for the selected carbons. Reproduced with permission from ref.^804^ Copyright 2020, the authors under CC-BY 4.0 license; E) Tauc plot of carbon nanoparticles sampled at different heights (7–14 mm); indirect transitions are assumed; the linear extrapolation from low energies (Tauc region) is different depending on the sampling height. The linear extrapolation from high energies (Urbach region) is similar for different samples. Reproduced with permission from ref.^805^ Copyright 2022, the authors under CC-BY 4.0 license; F) Tauc plots of semiconductor/MWCNT composite films showing the slope values of the Urbach region fitting (lines). Reproduced with permission from ref.^814^ Copyright 2020, Elsevier; G) Surface conductivities of semiconductor/MWCNT composites versus direct (Ed) and indirect (Ei) band gap energies determined by Tauc approach. Reproduced with permission from ref.^814^ Copyright 2020, Elsevier.

A common practice in the optical characterization of metal oxides with strong absorption features is to use a dilution with a nonabsorbing matrix,^810^ since reflectance is only sensitive to the relative amount of optical absorbing materials. The dilution approach has proven to be useful to estimate the optical band gap of porous carbons alone (Figure 27B,C).^804,811^ Identifying the electronic transitions (direct, indirect) in carbons of a different nature remains a challenge, and even though no significant variations are usually found in the band gap energies calculated in either case (Figure 27C), allowed transitions would seem to be more suitable for describing amorphous carbons.^812^ Functionalized carbons with O- and S-groups displayed absorption profiles characterized by broad band tails spanning over a wide range of energies that point out the existence of multiphonon absorption processes in those materials.^286,804^ The band gap energies for functionalized carbons range between 1.5 and 2.3 eV, with no clear trend relating to the extent of the oxidation (samples CV, Qox, NoC compared to unfunctionalized samples CVH850, Q, NOCH850) or the presence of sulfur (samples F, F6, FS6)^804^ (Figure 27C,D). Similar responses occur in structurally disordered semiconductors.^813^

Recent studies have also shown that the analysis of the low energy (Urbach) region can complement the analysis of the high energy (Tauc) region when studying the spectral behavior of carbon materials^805^ and carbon/semiconductor composites^814^ (Figure 27F,G). Migliorini et al.^805^ pointed out that owing to their strong tail absorption, the traditional optical band gap concept may not accurately describe the optical features of carbon materials, whereas the analysis of the Urbach energy provides essential information to comprehend the optical properties of carbon nanomaterials (Figure 27G). The authors reported high Urbach energies for carbon materials that invalidate the optical band gap concept in describing light absorption, which calls for revisiting the theoretical description of light absorption in disordered carbon materials. Mergen et al.^814^ analyzed the optical transitions of semiconductor/MWCNT by various approaches, showing similar optical band gap energies upon the analysis of the Tauc and Urbach regions in the UV–vis diffuse reflectance spectra, compared to absorbance Spectrum Fitting methods. The authors showed a good agreement between both methods, and an indirect correlation between the band gap energies (calculated assuming both direct and indirect transitions) and the surface conductivity of the composites. This was explained by an increase in the charge carriers in the composites upon the incorporation of the carbon material (hence low band gap), and the increased mobility of those charge carriers (hence, higher surface conductivity), as corroborated by surface conductivity measurements.^814^

##### 4.4.3.2 Photoassisted Regeneration of Saturated Carbons

The good performance of TiO_2_/carbon composites over consecutive photocatalytic runs has demonstrated that these systems fulfill the requirements of long cycle-life and a good degradation efficiency.^756,815^ The photocatalytic efficiency of the TiO_2_/carbon composites based on hydrophilic carbons was somewhat lower than that obtained with hydrophobic carbons, with a marked accumulation of degradation intermediates as the number of photocatalytic cycles increased. A similar trend is typically reported for bare TiO_2_,^816^ leading in both cases to poor mineralization efficiency upon cycling. Conversion yields and mineralization rates were greatly enhanced in the presence of dissolved oxygen regardless of the nature of a pollutant. The effect is more pronounced with hydrophilic carbons, highlighting the key role of radical species to achieve a good long-term performance of the photocatalytic activity. The characterization of the carbons after several cycles illumination showed some clogging of the pores due to the incomplete degradation of the pollutant, with no significant changes in the composition.^756^

Aiming at understanding the mechanisms governing the photochemical activity of TiO_2_/carbon systems, photooxidation assays have been explored on porous carbons alone.^755,817^ Integrating adsorption in porous carbons and simultaneous photodegradation offers a unique and innovative strategy for an advanced wastewater treatment. Studies carried out by irradiating aqueous suspensions of the porous carbons preloaded with the targeted pollutants have demonstrated the photodegradation of the pollutant nanoconfined in the pores of the carbon material, with various extents of degradation depending on the nature of carbon. This has opened new perspectives on the application of photocatalytic processes to the (on-site) regeneration of saturated carbons,^815,818,819^ and may lay the grounds for a future implementation of carbons in advanced oxidation processes for the water treatment and regeneration of carbon adsorbents.

##### 4.4.3.3 Photooxidation of Water

Recent developments in the synthesis of hybrid heterojunctions combining semiconductors and in 3D porous architectures (e.g., metal organic frameworks, conjugated microporous polymers) have shown an interesting performance for the water splitting reaction under artificial irradiation and sunlight. Although it is known that many types of carbon (graphene derivatives, carbon nitrides, carbon nanotubes) are able to catalyze the water splitting reaction, research on the use of porous carbons for this reaction has received lesser attention, with some examples on CNT, carbon fibers and activated carbons.^96,820–831^

The majority of these studies report an enhancement in a productivity and/or efficiency of hybrid materials and attribute it to the combination of the photoactivity of a semiconductor with the pore structure of a carbon material that allows a homogeneous distribution of the semiconductor. Little attention has been paid to the understanding of the role of the composition (defect sites and heteroatoms) of the carbon materials in those heterojunctions, or to their potential activity as photocatalysts themselves. In this section, we will focus on those studies linking such a performance with the composition of the porous carbon material.

3D porous graphene (foams, aerogels, hydrogels) has received much attention in photocatalytic applications (degradation of pollutants, water splitting, CO_2_ photoreduction), since they display high surface areas while maintaining many of the electronic properties of the pristine graphene.^823,832–834^ 3D graphene combined with semiconductors and metallic photocatalysts has been largely studied for the photocatalytic production of H_2_. Besides the hackneyed effects on improving a charge separation, the dispersion of the photoactive material and stability provided by the photocatalytic performance. Most widely reported is N-doping, with numerous studies addressing the superior photocatalytic activity for hydrogen production under visible light irradiation upon the functionalization.^835–837^ Unfortunately, most of these studies do not provide deeper insights into the correlation between the performance and the nature of the N-sites.

Faria et al. reported the activity of CNT/TiO_2_ for the production of hydrogen from methanol and saccharides.^821^ The oxidation of CNT incorporated a variety of O-groups (phenols, carboxylic acids and anhydrides, carbonyls, lactones) that created stronger interface interactions between CNT and TiO_2_, and facilitated the dispersion of a metallic phase. The photocatalyst prepared by oxidizing CNT followed by a thermal treatment at 200 °C was the most active catalyst for the H_2_ generation, which was attributed to synergic effects of an enhanced charge separation and electron mobility (Figure 28A–C). The catalysts were annealed at 200 °C, which is not high enough to remove all the O-functionalities (phenols were predominant). Unfortunately, the authors disregarded the effect of those O-groups on the photocatalytic activity for hydrogen production.^821^

**Figure 28:**
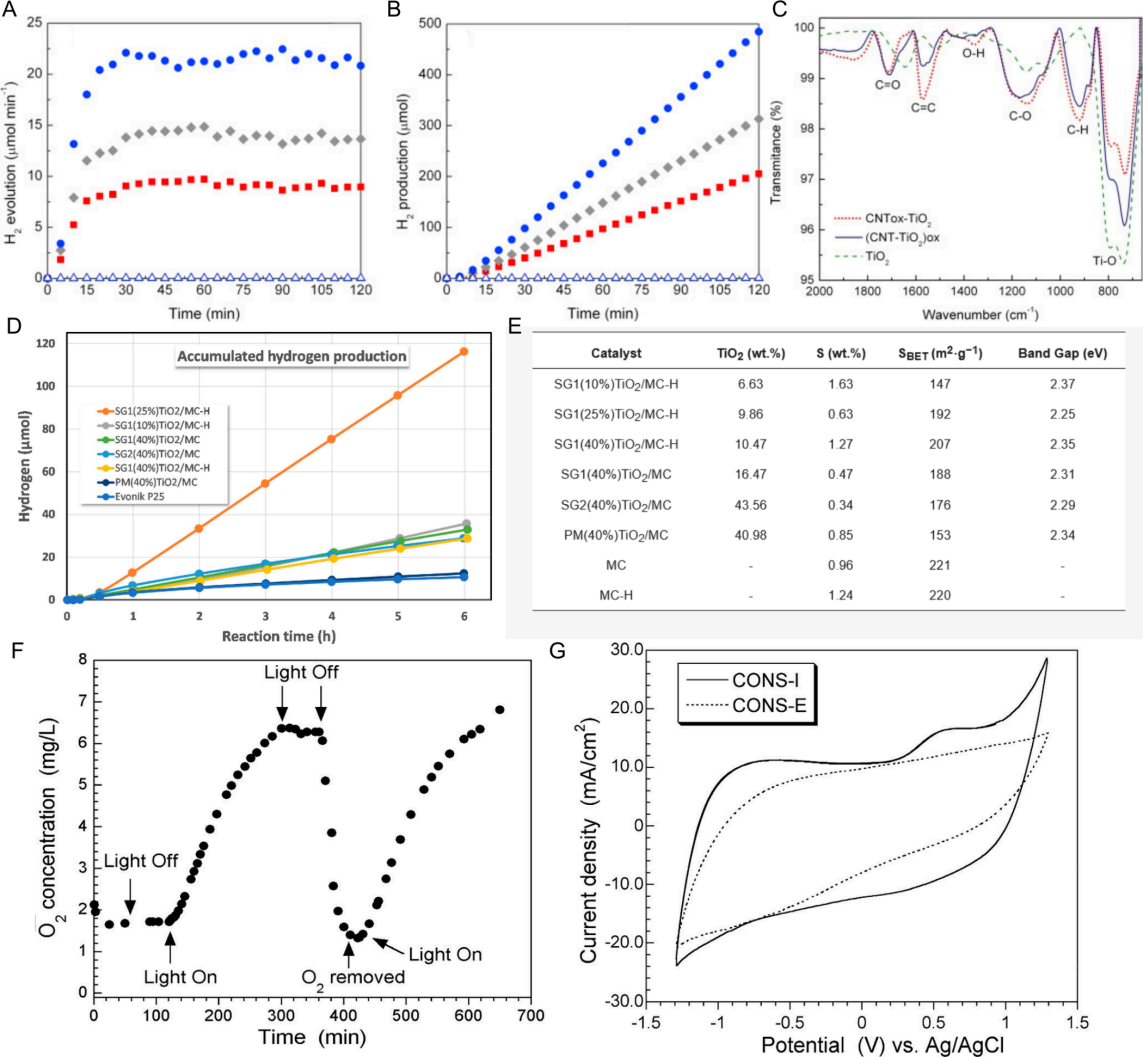
A) Rate of H_2_ evolution, and B) and total H_2_ production, and C) infrared ATR spectra of bare TiO_2_ (△), Pt/TiO_2_-473 (■), Pt/CNTox-TiO_2_-473 (⧫) and Pt/(CNT-TiO_2_)­ox-473 (●) from 10 vol % methanol solutions. Reproduced with permission from ref.^821^ Copyright 2014, Elsevier; D) Accumulated hydrogen production from aqueous glycerol photoreforming, and E) main textural, optical and composition parameters of TiO_2_-mesoporous carbons binary composites. Reproduced with permission from ref.^838^ Copyright 2020, the authors under CC-BY 4.0 license; F) O_2_ evolution upon on/off illumination and G) evidence of long-term photocorrosion of a CONS carbon photoanode. Reproduced with permission from ref.^277^ Copyright 2014, Elsevier.

Escamilla et al.^838^ reported the effect of the sulfur content on mesoporous carbon/TiO_2_ composites, showing that the incorporation of sulfoxide groups (ca. 1% total sulfur) did not modify significantly the optical band gap of the material. The composites prepared with functionalized mesoporous carbons achieved the highest hydrogen production markers in the photoreforming of glycerol (Figure 28D,E), displaying ca. 100 times higher activity than TiO_2_ powders (commercial P25).^838^ The authors explained this behavior to the superior dispersion of the titania particles, disregarding the effect of the composition of the carbon component.

Other authors have described the use of oxidized CNT for the immobilization of CdZnS active sites, reporting the excellent anchoring of semiconductor particles on the surface of oxidized CNT, owing to the abundance of oxygen-groups. Photocatalysts with an optimized formulation exhibited a high visible-light (λ ≥ 420 nm) photocatalytic H_2_-production rate, corresponding to an apparent quantum efficiency of nearly 8%.^839^ Similar results have been reported for porous carbon fibers/CdS,^840^ activated carbons/TiO_2_ ^830,841^ and carbon cloth/TiO_2_ composites.^842^ In all cases the photocatalytic activity was explained as the favored electron transport in the interface semiconductor/carbon material, which decrease the recombination rate of the photogenerated carriers in the semiconductors. Unfortunately, most of these studies do not provide direct experimental evidence of the improved electron transfer in the composites, nor consider the functionalization of the carbon support (as mentioned above, very often an oxidation treatment is performed to improve the dispersion of the photoactive phase on the surface of the carbon material).

Another recent study on TiO_2_/mesoporous carbon composites for the photocatalytic hydrogen production through glycerol photoreforming has reported the increased conversion on the composites prepared using oxidized carbon, owing to the incorporation of carboxylic groups.^838^ The conversion normalized per gram of TiO_2_ as the photoactive material increased 50–100 times in the composites with the oxidized carbon.

Doped porous carbons have also proven to be efficient protective layers of semiconductor photoanodes owing to the joint beneficial effect of the porosity and the functionalization of the carbon layer, that enhance the surface charge transfer and the strength of adsorption of water oxidation intermediates in the photoanode.^843,844^

Semiconductor and metal-free doped porous carbons have also demonstrated their potential as photoanodes to split water upon visible light irradiation (Figure 28F,G). Even though the performance is not as high as that of semiconductors or metal-containing photocatalysts for the same reactions,^277,845,846^ carbon photoanodes offer the possibility of tuning the porosity and composition to adjust the photochemical activity. The ability of these carbons to oxidize water has been mainly linked to the combination of a porous structure that favors the adsorption of water molecules in the pores close to the photoactive sites, high conductivity to facilitate the charge transfer and the presence of O-, N-, and S-containing photosensitizers -chromophore-like moieties such as thiophenic groups, sulfone/sulfoxides and some other, whose electrons are excited by irradiation, leaving reactive holes able to accept electrons from oxygen in water molecules.^277,845,846^ Interestingly, HRTEM observations of a S- and N-containing photoanode obtained by carbonization at 800 °C have shown the presence of graphite-like domains with size of ∼10 nm,^277^ which were suggested as determining the width of the band gap.^847^

Rendon-Patiño et al. reported microporous carbon photoanodes obtained from cyclodextrins and functionalized with N-groups (mainly as pyridinic and quaternary nitrogen) for the photocatalytic water splitting. The authors demonstrated the enhanced photocatalytic activity for H_2_ generation in the presence of methanol. An adequate microporosity (ca. average pore sizes of ca. 0.65 nm) and the nitrogen content of ca. 3.1 wt % allowed to achieve high hydrogen productivities in the presence of methanol, while the materials displayed lower efficiency in the overall reaction.^848^

The surface functionalization of the carbon photoanode can also lead to a decrease in a photocatalytic response upon prolonged illumination.^277,845^ A marked deactivation has been observed for N-, O-, and S-functionalized carbons due to photoinduced redox reactions involving some chromophoric groups (reduction of quaternary nitrogen, carboxylic, sulfonic acids, sulfones, thiophenes), that resulted in the consumption of the photoactive sites of the carbon photoanode and in charge transfer limitations from water molecules to the actives sites, which are crucial for water splitting on nanoporous materials (Figure 28G). For carbon photoanodes of low functionalization, the drop in performance upon long illumination is smaller, and the partial deactivation has been attributed to the oxidation of the carbon matrix forming carboxylic and carbonyl-like moieties. The reduced photoactivity upon oxidation of the carbon has been attributed to a lower density of free reactive sites at the edges of the carbon matrix (where the O-groups are incorporated), and the reduced charge carrier propagation through their delocalization in the carbon matrix as a result of the electron-withdrawing effect of O-groups.

##### 4.4.3.4 Photocatalytic Reduction of CO_2_

The photocatalytic reduction of CO_2_ through its conversion into fuels and other added-value compounds of industrial interest has become a hot research topic to face the need to reduce the amount of carbon dioxide emitted into the atmosphere. However, the photocatalytic reduction of CO_2_ is complex and typically delivers a low efficiency due to thermodynamic and kinetic factors. From a thermodynamic point of view, CO_2_ is one of the most stable molecules due to the C=O bond and linear structure, and its reduction in water is a highly endothermic process that requires multiple transfer of electrons.^849,850^ For instance, at least 2 electrons are required to produce CO and formic acid (making them the most common products), while the formation of methanol requires 6 electrons and that of higher hydrocarbons (ethylene) requires an even greater number of electrons. From a kinetic point of view, such a multielectron reduction process is unfavorable.^851,852^

Most efficient photocatalysts reported for this reaction are metal oxides, carbides and sulfides.^852–855^ However several issues related to their limited availability, cost, and poor selectivity from the competing hydrogen evolution reaction restrict their practical application. This has triggered the interest for alternative metal-free photocatalysts for this reaction, and recent studies have demonstrated that the use of porous photocatalysts (metal organic frameworks, conjugated microporous polymers) is an interesting strategy to achieve high photocatalytic conversions owing to their favorable (tunable) CO_2_ adsorption and transport properties, and the enhanced charge transfer and separation. Since the interaction between CO_2_ and the catalytic active sites can be enhanced due to the confinement in the nanopores, controlling the affinity for the pores and introducing photosensitive groups can position porous materials in the pool of catalysts for this application.^854,856–858^

In general, 3D and hierarchical porous photocatalysts with varied morphology (nanofibers, spheres, nanotubes) offer kinetic advantages (diffusion, mass transfer) for the reduction of CO_2_. In a typical case, 3D porous carbons are used as supports of photoactive materials, as it is believed that a hierarchical porous network facilitates the dispersion of the catalytic phase, and can significantly improve light harvesting and accelerate gas diffusion, provide abundant accessible catalytic sites, and superior charge separation efficiencies.^834,859–863^ Although the pathway of a charge carriers transfer and separation generally depends on the band gap of a photocatalyst, transient photocurrent measurements in TiO_2_/porous carbons reflect the influence of the porous carbon on improving the charge separation efficiency. Although CO_2_ reduction likely occurs at the catalytic sites on TiO_2_, it has been reported that porous carbons act as both CO_2_ adsorbents and photosensitizers, increasing the separation efficiency of the photogenerated electron–hole pairs via their interfacial interactions at TiO_2_. Furthermore, porous photocatalysts are determinant in minimizing light scattering losses in a photoreactor. Wang et al.^859^ have reported the excellent photocatalytic CO_2_ reduction on CdS immobilized on ordered N-doped macroporous carbon (Figure 29). Fast and strong adsorption of CO_2_ molecules on the catalyst’s surface accelerated the formation of a COOH* intermediate, that was then reduced to CO following a one proton–electron-transfer reduction process. While the study used a N-doped macroporous carbon, the role of a heteroatom (if any) in the photocatalytic activity was not described. Other authors have reported that dispersing mixed metal oxyphosphides (photoactive materials for the reaction) in the cavities of hollow carbon fibers enabled the efficient CO_2_ photocatalytic reduction under visible light, since the immobilization prevented the aggregation of the photoactive material and facilitated an electron–hole transport.^861^ Benefiting from the high surface area and abundant microporosity of polymeric and 3D graphene, Wang et al.^863^ reported enhanced in CO_2_ conversion efficiencies and selectivity for the CH_4_ formation with the negligible amount of H_2_ produced under visible-light irradiation for a polymer/TiO_2_/graphene composite photocatalyst.

**Figure 29:**
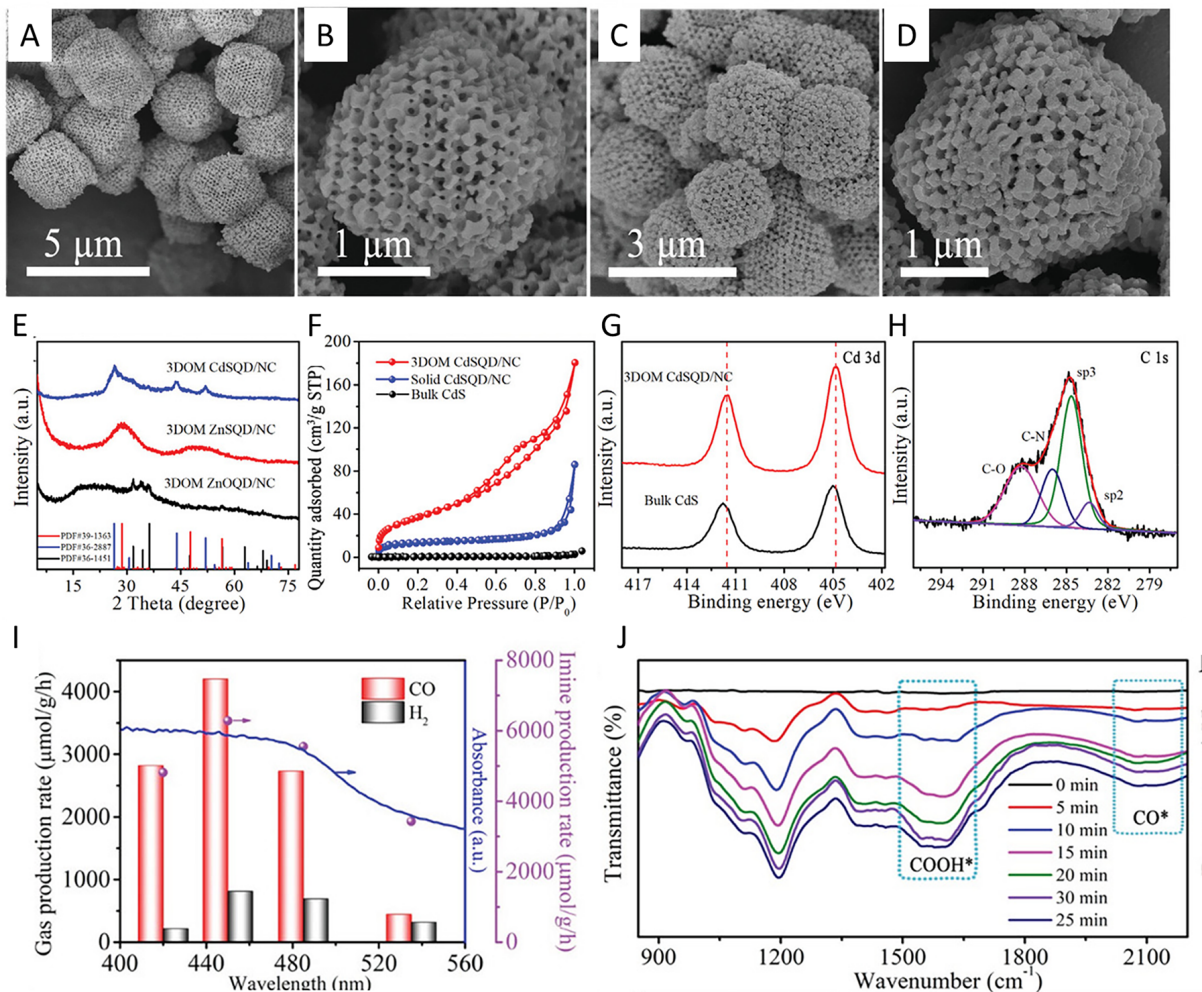
A–D) SEM images (A,B sample 3DOM ZnOQD/NC; C,D sample 3DOM CdSQD/NC), (E) XRD patterns, F) nitrogen adsorption/desorption isotherms at −196 °C and G), H) High resolution XPS spectra of Cd 3d and C 1s of 3D ordered macroporous N-doped carbon (NC) supported ZnO and CdS quantum dots; I) Wavelength dependent photocatalytic activity and light absorption spectrum of sample 3DOM CdSQD/NC; J) In situ FTIR spectra for the adsorption and photocatalytic activation of CO_2_ on 3DOM CdSQD/NC. Reproduced with permission from ref.^859^ Copyright 2021, Wiley-VCH.

Up to now, few studies have addressed the role of surface chemistry of the porous carbons in the photocatalytic activity for the reduction of CO_2_. Li et al. investigated carbon nitride/carbon composites with varied composition of the porous carbon for the photocatalytic reduction of CO_2_ with H_2_O under simulated solar light.^864,865^ The results showed that thiophenic-S increased the carbon photosensitivity and modified the band gap energy and band alignments of the photocatalyst, favoring the formation of CO.^864^ The improved photoactivity of the S-doped carbon was attributed to an increased charge transfer of the carrier owing to the increased conductivity of the material. However, the materials suffered from photocorrosion issues resulting from the modification of the functionalization of the carbon (oxidation of thiophenic sulfur).

Microporous graphitic carbons derived from cyclodextrin and functionalized with N- and P- have shown the photocatalytic activity in the reduction of CO_2_ with a preferential formation of CO, and an apparent quantum yield for CO below 1% under simulated solar light.^866^ The authors described the important role of the microporosity of the carbons. The catalyst showing the smallest pore size was the most active one, due to the tight fitting of CO_2_ to its narrow micropores. On the other hand, no positive influence of N- or P-doping observed under the applied experimental conditions was attributed to a misalignment of the band energy of the photocatalysts.^866^

Zhang et al.^867^ described the CO_2_ photoreduction on ZnO nanowire arrays deposited on the surface of 3D N-doped graphene, with selectivity for the production of methanol. The role of N-functionalization in the composite was discussed as facilitating the uniform growth and dispersion of the ZnO photocatalysts, improving the separation of electron–hole pairs and providing active sites for the adsorption (and reduction) of CO_2_. The authors reported a fast transfer of electrons between ZnO and N-doped carbon (e.g., photogenerated electrons on the semiconductor transferred to the carbon material acting as an electron sink), and that the multielectron CO_2_ reduction benefits from the accumulation of photogenerated electrons in the N-doped carbon matrix. Furthermore, they demonstrated that graphitic N-moieties favor the effective charge transfer from ZnO, with CO_2_ being reduced to methanol at pyrrolic and pyridinic N-groups.

Xia et al.^868^ reported a hierarchical composite based on 3D porous N-doped graphene and ZnIn_2_S_4_ nanowall arrays, with enhancement in solar-driven CO_2_ photoreduction into CH_4_, CO and CH_3_OH in the absence of cocatalysts and sacrificial agents, demonstrating by *in situ* irradiated XPS and Kelvin probe measurements the improved charge transfer owing to N-functionalization of the porous carbon.

##### 4.4.3.5 Other Photocatalytic and Photothermal Reactions

The interest in exploring the possibilities of metal-free and semiconductor-free carbons in photocatalysis is expanding rapidly, with various interesting works focusing on photochemical and photothermal carbocatalysis, using various forms of carbons for photocatalytic transformation reactions other than those described in the previous sections.^744^

The immobilization of photoactive moieties and molecules on carbon materials has been widely explored, with numerous examples on the covalent and noncovalent bonding on carbon materials (CNT, GO, RGO) and their successful application in various oxidative photocatalytic reactions.^801,869–871^ The oxidation of the carbon material is a common preliminary step to improve the immobilization of the photoactive molecules. However, despite abundant evidence of the remaining functionalization of the carbon matrix, this process is not sufficiently described.^872^ One can wonder if those O-groups contribute to the photocatalytic reaction, given that the density and type of oxygenated groups can confer photocatalytic activity to carbon materials, as discussed in previous sections.^869,873^

An illustrative example can be found in the work by Xu et al. about the photosensitization efficiency of 3D graphene aerogel with an organic dye, and the application in the photochemical hydrogenation of 4-nitroaniline and the photocatalytic reduction of Cr­(IV) under visible light irradiation^801^ (Figure 30A–C). The excellent photocatalytic activity in both reactions was associated with the favorable sensitizing effect of the dye in the interconnected conductive porous structure of the aerogel. The 3D graphene aerogel was synthesized upon hydrothermal expansion of graphene oxide, and although the oxygen content markedly decreased compared to that in the precursor, an XPS analysis showed the presence of carbonyl and hydroxyl groups on the surface of the material. It would have been interesting to consider the potential role and/or deactivation of such groups. A major challenge for the near future is to elucidate the role of these functional groups in the target reaction and/or the stability, reproducibility and long-term performance of the materials.

**Figure 30:**
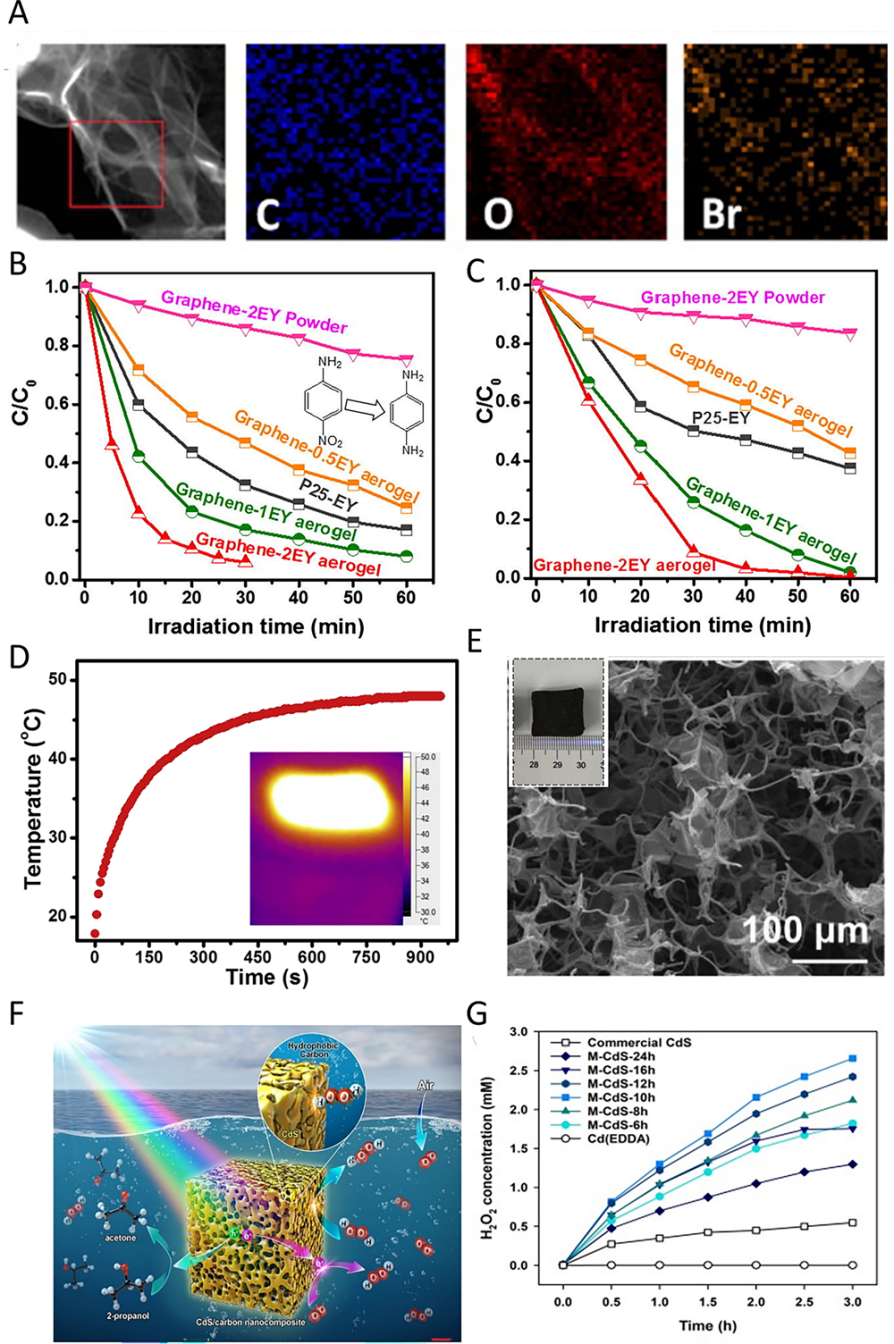
A) High-resolution TEM images and electron diffraction pattern of C, O and Br on the selected area of a graphene-dye sensitized aerogel; (B) visible-light-driven (λ > 420 nm) photocatalytic hydrogenation of 4-nitroaniline, and (C) reduction of Cr­(VI) over graphene–dye aerogels, graphene–dye powder and P25–dye powders. Reproduced with permission from ref.^801^ Copyright 2016, Elsevier; D) The temperature changing course and E) TEM images of a N-doped graphene aerogel under solar illumination of 3 kW m^–2^ (inset is the IR image after irradiation for 30 min). Reproduced with permission from ref.^874^ Copyright 2018, Elsevier; F) Schematic representation of the active sites of a porous sulfur-doped carbon/CdS photocatalyst for H_2_O_2_ production and G) Photocatalytic H_2_O_2_ production under visible light irradiation for various formulations prepared with different synthetic times (i.e., different carbon contents) in aqueous solution with 10% of 2-propanol. Reproduced with permission from ref.^875^ Copyright 2020, Elsevier.

Due to the high photothermal effects of many carbon materials, their use in photothermal photocatalytic reactions is also very promising.^876^ Fast temperature increases of ca. 20–60 °C have been observed under visible light irradiation of a few minutes, which has been successfully used to accelerate the conversion and rates of many reactions (Figure 30D,E). Examples can be found in N- and S-doped carbons for the photothermal production of hydrogen under visible light,^877^ solar driven desalination of seawater,^878–880^ or the Suzuki coupling reaction.^881^ The alteration of the electronic structure of the carbon atoms upon the incorporation of the heteroatoms is critical to improve photothermal conversion efficiencies and an increased stability and durability. The field is rising and merits further analysis of the role of the properties of carbon materials in the future.

Direct photoactivity of sulfur-containing carbons under UV and visible light (VL) irradiation was demonstrated by Seredych et al.^882^ They used S-modified carbon to adsorb dibenzothiophene (DBT) and dimethylbenzothiophene (DMBT) from simulated liquid fuel. As a next step, the spent carbons were exposed to UV and VL and kept in the dark. Then the adsorbed species were extracted with furan and a broad range of oxidized compounds originating from DBT and DMBT was detected including sulfonic acids, sulfoxides, sulfone and hydroxylated analogues of both adsorbates. The activity of sulfur doped carbon was much higher than that of as-received material and, interestingly, the higher level of oxidation was found under VL than upon UV irradiation, testifying about the importance of sulfur incorporation. However, the mechanism of the process was not analyzed in detail.

##### 4.4.3.6 Photocatalytic H_2_O_2_ Production

Hydrogen peroxide is a strategic oxidizing chemical for numerous productive sectors (paper, food and beverages, textiles and laundry, electronics) and a potential energy carrier to generate electricity in H_2_O_2_ fuel cells.^883^ The solar-driven photocatalytic generation of hydrogen peroxide has attracted the attention as a sustainable alternative^884–887^ to the industrial production of H_2_O_2_ based on an anthraquinone method, an energy-intensive process with several technological drawbacks (i.e., large waste production, low efficiency, mass transfer limitation, excessive solvent use).^888^

Investigated catalysts for the photocatalytic H_2_O_2_ production include carbon nitride, TiO_2_, CdS, MOFs, and COFs, and their performance is still hindered by several issues common to all photocatalytic reactions: a recombination of electron–hole pairs, limited visible light activity, or selectivity. Recent studies have demonstrated the potential of porous catalysts for the photocatalytic H_2_O_2_ production, yet the underlying mechanisms are lacking. The interest in porous carbons in this application is quite limited, with most research studies focused on porous g-C_3_N_4_ (out of the scope of this review) and few studies reporting hybrid composites with doped nanoporous carbons.^875,889,890^ Below we list some representative examples selected with a focus on highlighting the role of the surface chemistry of the porous carbon component.

Dang et al. reported the beneficial impact of embedding P- and N-doped carbons inside hollow sphere carbon nitride, to improve the selectivity for the two-electron ORR to produce H_2_O_2_.^889^ The authors attributed the effect to the improved transfer of electrons from the photoactive hollow spheres to the P- and N-carbon centers, contributing to decreasing the recombination of the photogenerated charge carriers. As a result, high H_2_O_2_ yield rates were obtained in water with and without sacrificial agents (ca. 240 and 4568 μmol h^–1^ g^–1^, respectively).^889^ On the other hand, Xian et al.^891^ prepared a composite of microporous catechol-formaldehyde resin microspheres and N-doped carbon nanoparticles with high photocatalytic H_2_O_2_ yield under visible light and without a sacrificial agent. The composite presented a high selectivity for H_2_O_2_ owing to the electron donor behavior of the C–N–H and C–O moieties in the composite. Lee et al. reported CdS/sulfur-doped carbon nanocomposites showing an improved photocatalytic production of H_2_O_2_ under visible light, owing to the hydrophobicity of the sulfur-doped carbon that would prevent the decomposition of generated H_2_O_2_ (Figure 30F,G).^875^

Mesoporous structures seem to be useful to achieve high solar-to-H_2_O_2_ conversion efficiencies. Tian et al.^890^ reported the synthesis of a sandwich-structured hybrid mesoporous carbon/rGO composite, combining a favorable electron transport in rGO and a fast diffusion in the mesoporous channels. The resulting material displayed a selectivity for two-electron reduction of oxygen to H_2_O_2_, and a stable activity during 2 h of illumination, which was attributed to the favorable adsorption of O_2_ in defective sites in the carbon network and the mesoporosity of the material.^890^

##### 4.4.3.7 Photocatalytic Disinfection

The interest in the development of photocatalytic antibacterial agents has risen in recent years, triggered by the COVID-19 pandemic.^892–894^ Radical oxygen species have proven to be efficient to prevent the growth of bacteria and completely eliminate fungi and viruses, among other microorganisms, thus main research directions of light-responsive antibacterial materials focus on the application of semiconductors (ZnO, TiO_2_, CuS, g-C_3_N_4_) and organic photocatalysts.^895,896^ The proven ability of carbon/semiconductor composites and carbons alone to generate radical species suggests that they may be also of interest in photocatalytic antibacterial applications. Yet, the field has not been much explored, but the few studies on the antibacterial activity of carbon materials have pointed out the importance of the surface chemistry for these applications.^897–900^ As examples, a recent study on the antibacterial activity of N- and S-doped carbon quantum dots clearly showed the dependence of the activity on both Gram-negative and Gram-positive bacteria on the surface chemistry and surface charge, with N–H groups as more active than oxidized sulfur groups in bacteria killing.^898^ Even though the photoactivity was not considered, the experiments were run in VL, which might affect the performance. Lopez Diaz et al.^901^ have analyzed the antibacterial activity of O- and N-doped carbon nanoparticles, showing the role of amine and imine groups on the antibacterial activity through electron transfer processes between the N groups of the carbon material and the functional groups of the bacteria, inducing oxidative stress. The antibacterial activity of carbon/semiconductor composites based on CNT, GO, and RGO has been extensively investigated and associated with the oxidative stress caused by the photogenerated radical species (similar to semiconductors alone), with higher antibacterial activity of GO—compared to rGO—owing to the hydrophilic functional groups,^902,903^ the antibacterial and disinfection properties are rarely analyzed in terms of the reactivity of the surface groups. The field deserves more attention.

### 4.5 Energy Storage

Carbon materials play a very important role in the energy storage field. In real applications, carbon materials like graphite are the main components of negative electrodes in Li-ion batteries and porous carbons are the core of both negative and positive electrodes in commercial double layer capacitors (DLCs). However, the possibilities and opportunities of carbon materials, more specifically porous carbons, extend into other topics like CH_4_ and H_2_ storage, which have been the subject of strong fundamental research in the last decades of the last century. The technology for these applications is almost ready (or even finished) and the commercialization is conditioned by market development and demands.

#### 4.5.1 Porous Carbon Materials in Supercapacitors

Batteries and electrochemical capacitors (the so-called supercapacitors) are the most important energy storage devices that are key players in clean energy storage and production. The energy storage mechanisms in these devices are different, which makes them complementary. Batteries are based on faradaic reactions that occur between the electrode and the electrolyte. They result in a high energy density that can reach values as high as 250 Wh kg^–1^.^904^ However, because of the kinetics of an electron transfer reaction and reaction mechanism, the specific power of the batteries is limited, and some irreversibility cannot be avoided what reduces the desired lifetime.

Electrochemical capacitors have high power density and durability (typically higher than 10^5^ cycles), but their energy density is lower compared to that of batteries (typically around 10 Wh kg^–1^ in commercial systems).^905–907^ Another disadvantage of capacitors is their higher rate of self-discharge compared to batteries, which can be problematic for energy storage over long periods. These devices have an important role as complement to other energy systems such as batteries or fuel cells or by themselves they are used in stationary systems or in transport applications.^907,908^ Electrochemical capacitors can be classified according to the main energy storage mechanism in electric double layer capacitors (EDLCs), in which the energy storage occurs through the formation of the electrical double layer due to ions adsorption under polarization conditions, and pseudocapacitors, in which the energy is stored through fast and reversible redox reactions taking place at the surface of the electrodes. The main mechanism in porous carbons is the capacitive process due to ions adsorption/desorption. The commercial electrochemical capacitors consist of two electrodes immersed in an electrolyte and connected by a separator. Most of the commercial devices use porous carbons as electrodes and, thus, they are considered as EDLCs. The high cycle life characteristic of these devices is a consequence of the mechanism of energy storage that ideally does not involve any faradic reaction. However, because of the voltage used in the capacitor, the potential reached by the electrodes is high and produces some undesired reactions with the electrolyte and collector, which are mainly responsible for their gradual degradation. The research on the mechanisms of the degradation process is one of the main topics for improving the supercapacitors properties and, for the porous carbon electrode, this is strongly determined by the surface chemistry.

The application of porous carbon materials as electrodes in supercapacitors (SCs) has been extensively studied during the last decades, and the effects of the porosity (including textural properties and structure) and surface chemistry have been investigated thoroughly. The synthesis of carbon materials with different properties and their effect on the electrical conductivity, wettability by an electrolyte and electrochemical stability have been the focus of numerous studies. An example of reviews where the fundamentals of electrochemical capacitors and double layer formation and charging dynamics have been discussed in ref. 633,905,909 and the topics related to carbon materials are covered in ref. 910–916

In the case of EDLCs, which is the one corresponding to porous carbons, the characterization of the device involves the measurement of energy and power.^905^ The energy is obtained from eq 1:

1 $$E=\int_{0}^{Q}VdQ=\int_{0}^{Q}\frac{Q}{C}dQ=\frac{1}{2}\frac{1}{3.6}CV^{2}$$

where *E* is the specific energy (Wh kg^–1^), *C* is the gravimetric capacitance (F g^–1^) and *V* is the applied voltage (V). These values should be also reported on volumetric basis (e.g., per unit volume of material/packed device), more reliable and precise parameters for evaluating the charge-storage capacity in mobile applications. This equation reflects the capacitance of a capacitor (i.e., a two-electrode cell) and, for a symmetric capacitor, the two-electrode capacitance corresponds approximately to one-quarter of the single-electrode capacitance.

The maximum power is obtained from eq 2 and the average power for specific discharge conditions is given by eq 3.

2 $$P_{\max}=\frac{V^{2}}{4ESR}$$

3 $$P=\frac{E}{t_{d}}$$

where *P*_max_ is the maximum power (kW) and ESR is the equivalent series resistance of the capacitor (Ω). The average power *P* (kW kg^–1^) is calculated from the energy *E* and the discharge time (*t*_d_). The power on volumetric basis is given in kW L^–1^.

From the above equations, the most important parameters in capacitors are the capacitance, voltage and equivalent series resistance (ESR), which are determined by the electrodes and electrolyte of the capacitor. Several publications address recommendations for the measurement of these properties as well as the durability of the capacitor (one of the main parameters that determines the applicability of a material as an electrode for SCs).^917–919^ These recommendations must be followed to have comparable results with other publications and to get the correct and fair characterization of the materials.

The total capacitance of an electrode in contact with an electrolyte has three contributions: the quantum capacitance (*C*_Q_), the dielectric capacitance (*C*_E_), and the double layer capacitance (*C*_EDL_).^633^

4 $$\frac{1}{C_{\mathrm{total}}}=\frac{1}{C_{Q}}+\frac{1}{C_{E}}+\frac{1}{C_{\mathrm{EDL}}}$$

A quantum capacitance is related to the variation in the electron density under the application of a potential; a dielectric capacitance is due to dielectric screening of the electrode; and a double layer capacitance is a consequence of the interactions between an electrode and electrolyte.^633^ From studies carried out on few layers graphene, it was concluded that *C*_Q_ increases with an increase in layer stacking while *C*_E_ decreases, reaching similar values for four graphene layers. However, *C*_EDL_ remains nearly constant with the number of graphene layers^920^ (Figure 31A). When the measured capacitance has a strong contribution from the quantum capacitance, the cyclic voltammetry curve usually has a butterfly shape,^921^ compared to the rectangular shape mostly observed for porous carbons, when the dominant contribution to the capacitance is EDL. In conventional porous carbons, *C*_EDL_ can be a dominant factor, although the contributions from the other factors should also be considered since they are very much dependent on a pore wall thickness and material composition. In this sense, although *C*_EDL_ may reach its highest value for a highly porous material, the total capacitance can be increased by increasing *C*_Q_ through defects incorporation, doping and surface functionalization.^920,922,923^ Dielectric screening could be decreased by increasing the conductivity or decreasing the pore wall thickness.^920^ It is important to note that dielectric screening contribution to the total capacitance is not usually considered in explaining the total capacitance of few layers graphene, although this contribution can be important when increasing graphene layer stacking.^920^

**Figure 31:**
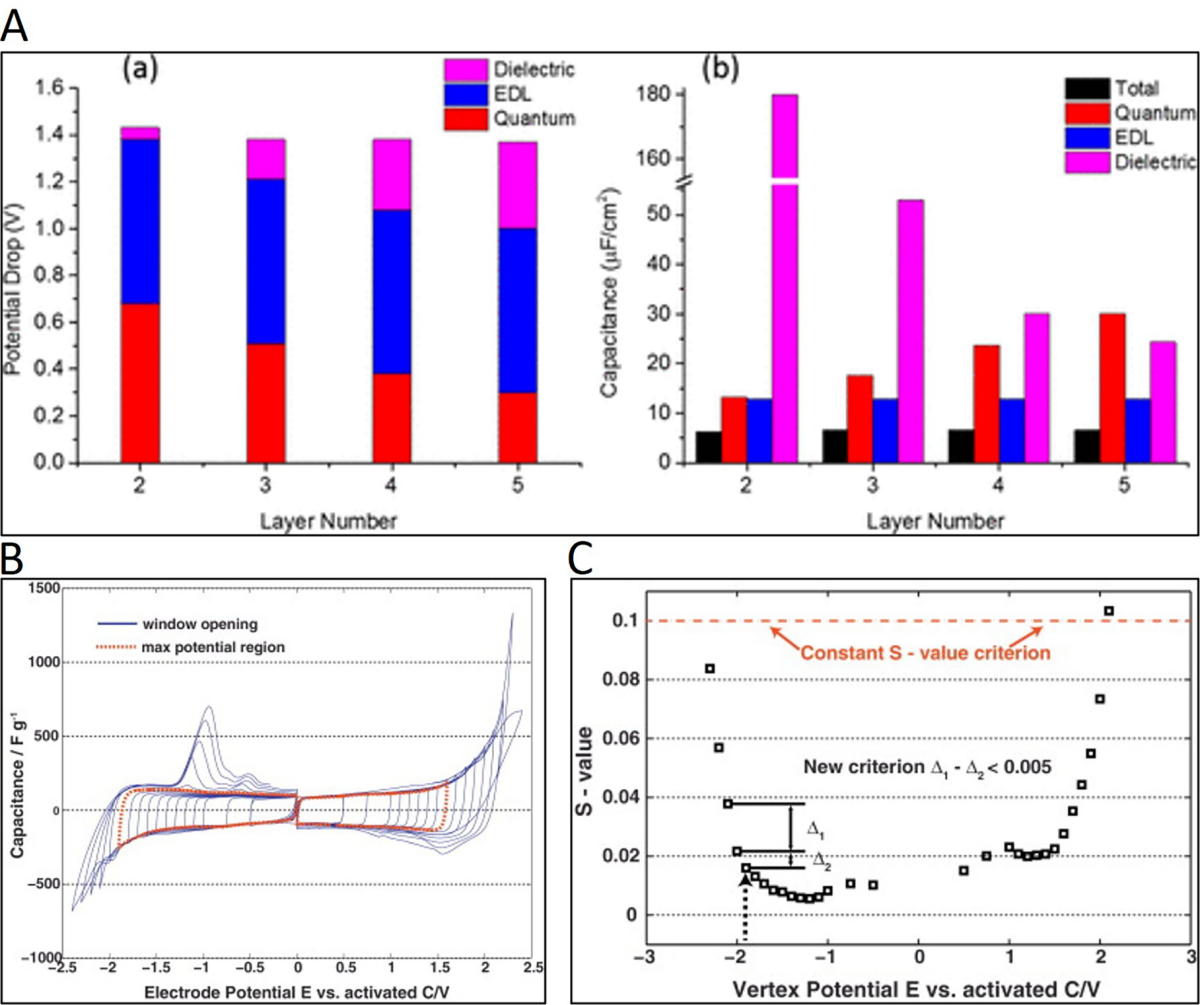
A) (a) Breakdown of the potential drop into quantum, EDL, and dielectric screening contributions with the layer number under the same surface charge density of 9 μC cm^–2^. (b) Corresponding capacitance contributions. Reproduced with permission from ref.^920^ Copyright 2016, American Chemical Society; B) Cyclic voltammetric potential window opening experiments. Working electrode: BP2000; RE and CE: YP17; electrolyte: [EMIM]­[BF_4_]; scan rate: 1 mV s^–1^. The dashed line indicates the maximum potential excursion. Pos./neg. sweeps in separate cells. Reproduced with permission from ref.^924^ Copyright 2013, Elsevier; C) S-value vs vertex voltage plot for IL [EMIM]­[BF_4_] at RT. The black arrow indicates the stability limit applying the new criterion. Dashed red line: stability criterion according to Xu et al.^925^ Reproduced with permission from ref.^924^ Copyright 2013, Elsevier.

The most important challenge in the field of SCs is increasing the stored energy so that they can compete with batteries in different application sectors without compromising the power characteristics of these devices. According to the equations presented above, the stored energy can be enhanced by increasing either the capacitance or operating voltage. Regarding the capacitance, it is primarily determined by the porous texture (including its structure) and surface chemistry. Concerning the porosity (which contributes to the capacitive energy storage) and even surface chemistry (which plays a role in Faradaic effects), porous carbons reach capacitance limits that prevent a substantial increase in energy stored compared to batteries.

In terms of operating voltage, the electrolyte stability determines the capacitor voltage.^911^ Even though the capacitors based on aqueous electrolytes provide the highest capacitance values and are more environmentally friendly, the thermodynamic decomposition potential of water restricts the capacitor voltage to around 1 V. Organic electrolytes enable operation at voltages between 2.5 and 3 V, and although their capacitance is significantly lower, an energy increase is greater due to the higher voltage. Other electrolytes, such as ionic liquids and their mixtures, allow the working voltages up to 5 V. They are more environmentally friendly than the organic electrolytes are, but have limitations due to their high viscosity.

It is worth noting that a suitable carbon material can help to increase the capacitor energy by increasing its operating voltage beyond the thermodynamic limit. This occurs due to the electrochemical stability of the electrolyte in the presence of the carbon material (resulting from the overpotential for electrolyte reactions). By adjusting the potential window of the positive and negative electrodes working in an asymmetric configuration and/or the incorporation of adequate additives, aqueous electrolytes can safely operate at voltages of up to 1.9 V.^926,927^ Therefore, a modification of surface chemistry of porous carbons to increase the overpotential of electrolyte reactions (which is typically more relevant in the positive electrode) may be more beneficial than its tailoring to introduce the pseudocapacitance to enhance the capacitance of the material.

On the other hand, determining the stability limits of the electrolytes in the EDLC is a highly relevant aspect for selecting the most appropriate potential window. These potential limits are kinetic constraints resulting from the overpotential of decomposition reactions occurring in the presence of carbon materials. Several methods have been proposed to define these potential windows.^924,925,928,929^ Figure 31B,C contains relevant information about the experimental work for the determination of the electrochemical stability limits for electrochemical double layer capacitors.^924^ Figure 31C presents the S-value calculated from the charge obtained during charge and discharge in the cyclic voltammograms (such as those in Figure 31B) obtained at different potential window openings (S= $\frac{Q_{\mathrm{charge}}}{Q_{\mathrm{discharge}}}-1$ ).

These S values are used to define the electrochemical stability windows for DLC. Regardless of the method applied, the experiment should be performed under conditions as close as possible to the actual application, avoiding the use of model materials with low double-layer charge. This ensures that the obtained results can indicate the electrolyte stability under the measured conditions, although variations may arise depending on the properties of the porous carbon material used, given the substantial influence of surface chemistry and the porosity on its reactivity with the electrolyte. For instance, it is known that oxygen functional groups promote electrolyte decomposition.^925,926,930^ Therefore, determining electrolyte stability using the porous material intended for the application in EDLC is essential.^929^

Regarding the ESR of a capacitor, it is determined by various factors, including an electrode resistance (which includes inter- and intraparticle resistance), the resistance between the electrode and the current collector, electrolyte resistance, ion diffusion resistance within the material porosity, separator resistance, and external contact resistance. When ESR increases, power output decreases, leading to heat generation in the system, which negatively affects the performance. The progressive degradation of the capacitor caused by high power demands, a temperature rise, gas generation, and electrode deterioration results in an increased ESR and a reduced life of the device. This highlights the necessity of minimizing ESR to maintain an optimal performance as long as possible. Different approaches exist to reduce ESR in capacitors. One method is enhancing the conductivity of the carbon material used, while another involves modifying the overall structure and morphology of the electrode. In the former, highly conductive materials such as carbon nanotubes or graphene-based materials could be employed, although their capacitance tends to be lower than that of activated carbons traditionally used in this application. The latter involves modifying the electrode during its fabrication. Supercapacitor electrodes typically consist of porous materials with particle sizes in the micrometer range (typically around 10 μm), a conductivity promoter (such as high-conductivity carbon black), and a polymer binder (e.g., PTFE, CMC, PVDF, ...) to provide a shape and cohesion.^931^ While the binder improves a particle contact, it is an insulator, necessitating the presence of a conductivity promoter. New electrode preparation strategies aim to further reduce the resistance, including the use of highly conductive materials, a direct deposition of the carbon material on the current collector, or the fabrication of monolithic structures with continuous architectures that eliminate grain boundaries.^932–935^

Consequently, from the point of view of the porous carbon materials, the most important requirements can be summarized as follows: (i) A high development of a (micro)­porosity to achieve high capacitance values; (ii) an ordered porous structure and the presence of mesopores to facilitate ion diffusion within the porous network, thereby ensuring the high power performance; (iii) low electrode resistance, contributing to a lower ESR; (iv) suitable surface chemistry to enhance various properties, such as wettability, electrical conductivity, Faradaic effects (i.e., pseudocapacitance), and the electrochemical stability of the carbon/electrolyte system.

Concerning *porosity and porous structure*, it is known that capacitance increases with the specific surface area. However, constant capacitance values have been found for surfaces larger than approximately 1500 m^2^ g^–1^,^936^ although other studies report proportional capacitance values for even higher specific surface areas.^937,938^ The differences seem to be related to the specificity of the porous structure. It is likely that the thickness of pore walls influences the propagation of the electric field within the pores (i.e., dielectric capacitance). Moreover, as the porosity increases, the pore size also increases, which weakens ion adsorption and probably, the increase in the surface area is offset by the reduction in adsorption energy.^939^

The pore size, ion size, and structure significantly influence the formation of the electrical double layer, and the presence of micropores produces higher capacitance values. Previous studies have reported^939–941^ that specific capacitance increases as pore size decreases in both aqueous and organic electrolytes (Figure 32A), reaching a maximum at an optimal pore size, which is larger in organic electrolytes than in aqueous ones. The experimentally obtained optimal pore sizes are close to the size of desolvated ions, demonstrating that ions can adsorb in pores whose dimensions are comparable to those of the ions without their solvation shell. This optimal pore size corresponds to the narrow micropores, typically around 0.7 nm. Despite extensive efforts to understand the mechanism behind the capacitance increase in the pores of this size, no clear conclusion has been reached, probably due to the complexity of the electrical double layer and the different models used to describe it.^633^ Nevertheless, some interesting studies propose the formation of a “superionic state” due to the opposite charges generated at the pore walls upon polarization that screen the repulsive interactions between ions of the same sign inside the pores.^633,942,943^ Furthermore, the effect of pore size and its optimization in relation to the electrolyte used may be influenced by a molecular sieve effect, either because pores smaller than the desolvated ions are inaccessible^944^ or because complete desolvation does not occur, preventing electroadsorption, as observed with strongly solvated cations.^945–947^ Therefore, this effect is highly relevant and must be evaluated in each specific case.

**Figure 32:**
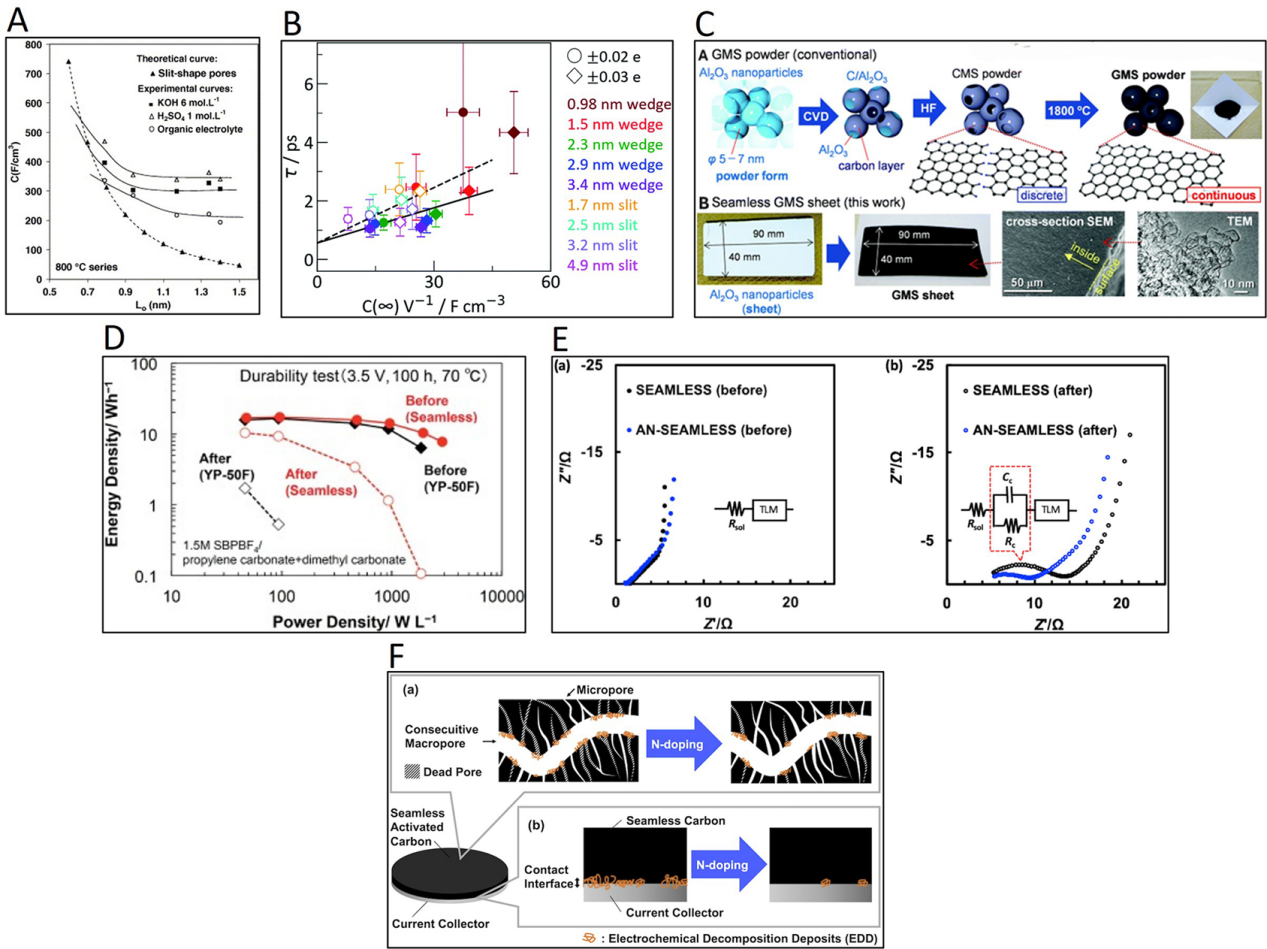
A) Relation between the volumetric capacitance (theoretical and experimental) and the pore size in aqueous or organic medium for the 800 °C series activated carbons. Reproduced with permission from ref.^932^ Copyright 2006, Elsevier.; B) Capacitance performance in various types and sizes of pores. The relaxation time of capacitance vs. volumetric capacitances in the partial charges ± 0.02 (circle) and ± 0.03*e* (diamond). Open symbols and dashed line: slit pores, and closed symbols and solid line: wedge pores. Reproduced with permission from ref.^948^ Copyright 2022, Royal Society of Chemistry; C) (A) Preparation scheme of the GMS powder, together with its photograph. For each step of the preparation, illustrations of a small portion of the Al_2_O_3_ nanoparticles (only five aggregated grains are shown in solid color) and carbon coating layers are given. The graphene structures in CMS and GMS are depicted. (B) Photographs of sheet-molded Al_2_O_3_ nanoparticles (template) and a resulting seamless GMS sheet. Cross-sectional SEM and TEM images of the GMS sheet are also shown. Reproduced with permission from ref.^964^ Copyright 2019, Royal Society of Chemistry; D) Ragone plots of the EDLC (two-electrode) cell using densified seamless activated carbon electrode or conventional activated carbon (YP50F) composite electrode before and after floating charge durability test (3.5 V, 100 h, 70 °C). Reproduced with permission from ref.^966^ Copyright 2023 Elsevier; E) Nyquist plot (a) before and (b) after floating durability test (3.5 V, 100 h, 70 °C) for a seamless porous carbon and the material after N functionalization (AN-Seamless). One M TEMABF_4_/PC, Two-electrode cell, 40 °C, 0 V, 1.0 × 10^–2^–2 × 10^3^ Hz. Reproduced with permission from ref.^963^ Copyright 2020, IOP Publishing; F) Model of (a) pore blocking and (b) contact resistance caused by electrochemical decomposition deposits (EDD) on tested electrodes to remark the effect of N functionalization. Reproduced with permission from ref.^963^ Copyright 2020, IOP Publishing.

The presence of wide micropores and narrow mesopores is also crucial to ensure rapid ion diffusion in the capacitors and their high-power performance.^948^ Figure 32B shows the relationship between the capacitance relaxation time and volumetric capacitance, showing that the highest relaxation times occurred for the narrowest pores, suggesting that wider micropores or narrow mesopores are beneficial to increase this parameter. However, an excessive amount of mesopores may negatively impact the volumetric energy storage capacity, which is a very important parameter from an application viewpoint. The existence of an interconnected pore network (including narrow and wide-micropores and narrow mesopores) that facilitates the fast diffusion of ions to the entrance of the narrow micropores supports the importance of a hierarchical porous structure in carbon-based materials.^603,949,950^ The connectivity of the pores with the particle surface, enabling a rapid ion diffusion within the porous network with a low tortuosity, is also a crucial aspect of the porous structure to ensure a high capacitance retention at fast charge and discharge rates.^951^ For this reason, porous carbon materials with an ordered porous structure are highly important for achieving high-capacity and high-power capacitors.^935,950,952^

Thus, porous carbon materials with a high porosity (high micropore volume), ordered or not, having interconnected porosity, and wide micropores or narrow mesopores might be the most suitable as electrodes for supercapacitors. Among these, the following stand out:

1) *Superporous carbons*, with apparent surface areas exceeding 3000 m^2^ g^–1^, which can provide high capacitance values in various electrolytes due to their high microporosity and the presence of narrow mesopores.^953–955^

2) *Ordered porous carbons*, such as zeolite templated carbons (ZTCs) and ordered mesoporous carbons (OMCs).^956,957^ ZTCs have the interconnected porosity with an average size of 1.2 nm, enabling rapid ion diffusion,^956^ and exhibit the high capacitance, especially in aqueous media.^958^ However, ZTCs have a high concentration of edge sites that results in their high reactivity,^149,959^ which limits their applicability as the electrodes in supercapacitors. An important property of these materials is their high pseudocapacitance, due to the presence of functional groups, which is found in both aqueous and organic media.^959,960^

3) *Carbon monoliths*, prepared using a binder-free strategy, which have a high density and a three-dimensional structure with a high degree of interconnection. These result in low ESR and a high electrochemical stability due to the low density of reactive sites or grain boundaries,^932,950,961–963^ decreasing the formation of decomposition product deposits responsible for pore blocking and increased ESR. This effect is further improved after nitrogen functionalization since it reduces the concentration of the most reactive sites.^930,932^ Figure 32D contains experimental results that compare the performance of a conventional activated carbon electrode (using commercial YP-50F activated carbon) and seamless porous carbon, showing the benefits of using a binder free strategy. Figure 32E,F shows the beneficial effects of nitrogen functionalization (an aspect that will be addressed below in detail).
4) *Carbon mesosponges*. These materials are synthesized via template-assisted CVD, which produces an elastic mesoporous material with a high surface area and minimal edge sites. They show an exceptional stability, excellent electrical conductivity, with a voltage window of up to 4 V, and an outstanding performance as electrodes in supercapacitors.^932,964,965^ Figure 32C includes a scheme showing the synthesis procedure of these materials.

Surface chemistry plays a fundamental role in the performance of the carbon-based capacitors. It contributes to the Faradaic effects, thereby increasing the stored energy. It can also enhance the wettability of the electrolyte, facilitating its access to the material porosity, and is crucial for the stability of both the electrode/electrolyte system and the electrode/current collector interface. The incorporation of heteroatoms into the structure of carbon-based materials significantly modifies surface chemistry and electronic properties, and the surface composition can be tailored to adapt materials for specific applications. For this reason, extensive research has been conducted on the effect of heteroatom incorporation on the behavior of carbons as electrodes in supercapacitors, with a particular focus on nitrogen and oxygen functional groups. While the studies on oxygen functional groups are numerous and some general conclusions can be drawn, further investigations are needed on the role of nitrogen-containing groups, because it is difficult to avoid the coexistence of both functional groups, making it challenging to deconvolute their individual roles.

It is well-known that functional groups play a significant role in wettability and an electrical conductivity, especially in aqueous electrolytes. The oxygen and nitrogen-containing functional groups enhance the interactions between the carbon-based material and the aqueous electrolyte, improving the wettability.^151,967,971–973^ In particular, carbonyl and, especially, phenolic groups are the most effective in promoting the electrolyte wettability. Regarding the nitrogen-containing groups, studies have shown that graphitic or quaternary nitrogen species may be responsible for this property. Additionally, these types of groups help to reduce the material resistance, thus contributing to an improvement in the electrical conductivity (Figure 33A).^967^ On the other hand, an excessive presence of oxygen-containing groups, especially CO_2_-type groups (due to their electron-accepting nature, reducing charge delocalization in the material), decreases the electrical conductivity of the carbon-based materials.^974^

**Figure 33:**
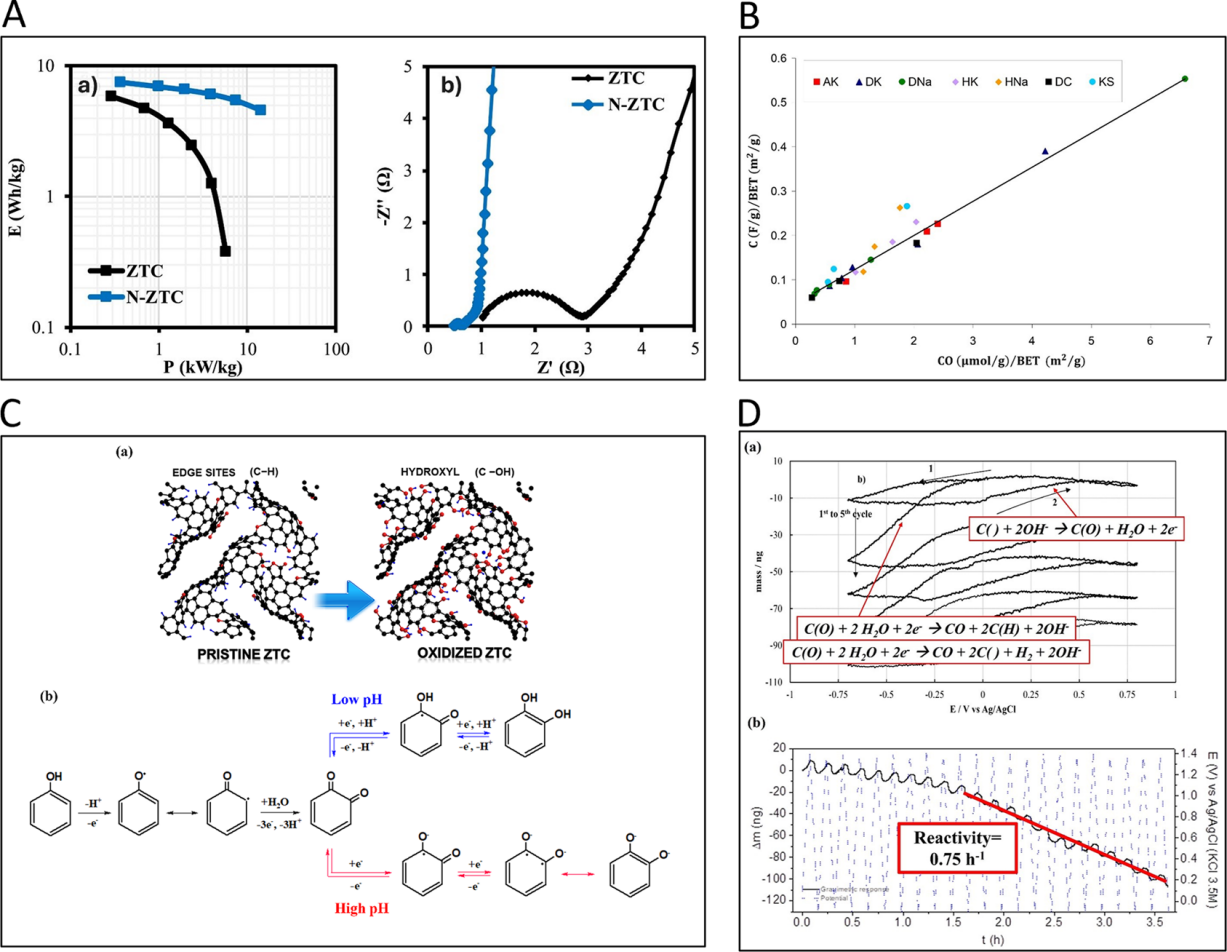
A) (a) Ragone plot obtained for ZTC- and N-ZTC-based supercapacitors at 1, 2.5, 5, 10, and 20 A g^–1^. *V* = 1.2 V. (b) Electrochemical impedance spectra for ZTC- and N-ZTC-based supercapacitors. *V* = 0.05 V. One M H_2_SO_4_. Reproduced with permission from ref.^967^ Copyright 2018, Elsevier; B) Specific capacitance vs CO content divided by BET surface area. Reproduced with permission from ref.^954^ Copyright 2005 Elsevier; C) (a) Structural model of pristine and oxidized ZTC. Reproduced with permission from ref.^968^ Copyright 2012, Elsevier. (b) Mechanism for the generation of carbonyl and quinone-type groups (in *ortho* position) from phenol under oxidation conditions. The scheme also includes the proposed reduction mechanism for quinone in low (blue pathway) or high (red pathway) pH. Reproduced with permission from ref.^969^ Copyright 2022, The Carbon Society of Japan; D) (a) Electrode mass behavior of a commercial activated carbon (AC) electrode in a solution pH of 12 at 50 mV s^–1^ for five cyclic voltammograms. Reproduced with permission from ref.^970^ Copyright 2009, Elsevier. (b) Gravimetric response between −0.10 and 1.40 V of ZTC at 5 mV s^–1^ in 1 M H_2_SO_4_ solution during 22 cycles of the 4th cyclic voltammetry experiment. Reproduced with permission from ref.^960^ Copyright 2015, Elsevier.

Oxygen and nitrogen functional groups also significantly contribute to the capacitance due to their electroactivity, which generates the pseudocapacitance. This phenomenon has been observed in both aqueous and organic electrolytes. Research on this topic is abundant and has primarily focused on oxygen functional groups and aqueous electrolytes. The findings and conclusions related to the oxygen and nitrogen functional groups in both aqueous and organic electrolytes are included in numerous reports.^149,151,954,959,967,972,973,975–982^ In the case of nitrogen functional groups, their electroactivity has been associated with pyridine-type groups,^976,978^ pyridones,^975^ and amines.^977,979^ However, the mechanisms of the redox reactions of the nitrogen functional groups and the specific species responsible for these processes remain unclear, due to the coexistence of oxygen functional groups, which can mask or modify their behavior.

The effect of the combination of nitrogen and oxygen groups on the pseudocapacitive behavior was reported.^980,983^ The authors analyzed the capacity of nitrogen-modified carbon in an acidic electrolyte. They found that pores between 0.5 and 0.6 nm were the most effective in a double-layer formation, following the size of hydrated ions and that pyridinic nitrogen, pyrrolic nitrogen and quinone oxygen, presumably located in pores bigger than 1 nm, markedly contributed to the pseudocapacitance. Quaternary and pyridinic N oxides showed enhancing effects on the capacitance due to their positive charge and thus an improved electron transfer. It was especially visible at higher current loads when the double-layer capacitance was more pronounced than the Faradaic effects.

The electroactivity of oxygen functional groups has been studied in great depth, mostly in aqueous electrolytes, leading to interesting conclusions about electroactive groups and the reaction mechanisms, even though each oxygen group may exhibit a distinct electrochemical behavior.^984,985^ This complexity is further increased because of the surface heterogeneity of the carbon materials. In this regard, a study has reported a correlation between the specific capacitance of various porous carbons and the content of CO-type groups, determined by temperature-programmed desorption (TPD (Figure 33B).^954^ The results indicated that CO-type groups are the main groups responsible for the redox processes observed in acidic aqueous electrolytes, which is in agreement with findings from other studies.^959,972,982,984^ Although anhydride groups have also been proposed as contributors to the pseudocapacitance,^151^ the primary contribution comes from CO-type groups. The CO-type groups mainly originate from the decomposition of phenolic, carbonyl, ether, anhydride, and quinone groups, making it challenging to identify the specific functional group responsible for the electroactivity. In this sense, a recent study^969^ focused on analyzing the electroactive CO-type groups with an aim to get insights into their chemical nature. Using the literature data, the authors estimated the charge of the redox processes in acidic aqueous electrolytes (subtracting the contribution from the electrical double layer) for various carbon materials (porous materials with similar structures and an electrochemically oxidized zeolite templated carbon (ZTC)). Assuming a one-electron redox process per the CO group, the quantity of the electroactive CO-type groups could be estimated. The findings indicated that the percentage of the electroactive sites relative to the total amount of the CO-type groups ranges from 12% to 20% in conventional porous materials, whereas in electrochemically oxidized ZTC (where selective electrochemical oxidation generates a large number of the CO-type groups without disrupting its structure^960,968^ ) this percentage reaches 60%. The higher number of the electroactive CO groups exists in ZTC due to its unique structure. It consists of three-dimensional buckyball-like nanographene with numerous armchair edge sites that can oxidize hydroxyl groups, which can be subsequently be converted into quinones. Figure 33C (part a) includes a structural model for ZTC showing the high concentration of edge sites and their selective oxidation to hydroxyl type groups. The transformation from hydroxyl to quinone groups can occur as previously reported for the formation of *ortho*-quinone from phenol (Figure 33C, part b).^986^

The electrochemical properties of molecules containing quinone groups have been extensively studied in both protic and aprotic solvents (see, for example, references 986–989 ). The properties of these molecules largely agree with the electrochemical characteristics of the redox processes observed in carbon materials, supporting the idea that the quinone groups are responsible for the redox activity observed in both aqueous and aprotic solvents. For instance, there is a clear dependence of the redox potential on pH (Figure 33C, part b). In acidic media, the second reduction reaction occurs very rapidly and overlaps with the first, resulting in a single peak in cyclic voltammetry. As the pH increases, the redox potential decreases, and the two redox reactions occur at different potentials. In some cases, the second reaction may be irreversible or even does not occur, which is attributed to the instability of the species formed. In that sense, carbon materials commonly show unclear redox peaks at high pH levels. In aprotic solvents, a similar trend was observed, where the occurrence of both reduction processes depends on the electrolyte used (which is consistent with experimental observations in carbon materials). Furthermore, the redox potential of quinones is influenced by their composition and structure (see data in reference 989), which explains broad and different peaks observed for carbon-based materials at different potentials. In summary, the quinone groups are the most probable species responsible for the electroactivity observed in carbon materials in different electrolytes. The amount of these groups that can be generated in a carbon material is structure dependent, since it will determine the amount of carbon edge sites where carbonyl or phenol groups in an *ortho* position can be incorporated for an efficient electroactivity.

Nevertheless, although the electroactivity of functional groups can provide some additional energy storage to the device, their presence is detrimental to the life of the capacitor since they are responsible for the increased *reactivity with the electrolyte*. In fact, the influence of surface chemistry on the reactivity of carbon materials with the electrolyte is one of the most critical aspects not only in the supercapacitors but also in other energy storage and conversion devices such as fuel cells and batteries. Reactions with the electrolyte should be minimized, as possible, because they contribute to the device degradation. In fuel cells, the reaction between the carbon-based cathode and the electrolyte (which remains under positive polarization and works at potentiodynamic conditions during startup and shutdown) leads to its oxidation and corrosion.^990–992^ Similarly, in metal-ion batteries (especially lithium-ion batteries), the formation of a solid electrolyte interphase (SEI) occurs due to reactions between the electrolyte and the electrode. The formation of this SEI is crucial for ensuring the optimal operation of batteries.^993–995^ In these batteries, the electrolyte is subjected to potentials beyond the thermodynamic limits of its oxidative and reductive decomposition. Once this interface is formed, these reactions are suppressed, allowing the electrolyte to exist under kinetic stability conditions. Regarding the supercapacitors, the reactions of the electrode (and a current collector) with the electrolyte contribute to the aging and degradation of the devices. Many studies have examined an electrolyte decomposition in relation to the reactions with the carbon electrodes,^930,996–1000^ as well as with a current collector, which is one of the most critical factors in the degradation of the aqueous capacitors.^1001^ Among the proposed solutions for the current collector protection, besides the use of anticorrosive solvents, such as deep eutectic solvents,^1002^ there are other options that are described in the literature.^1001,1002^ Despite the detailed research on this topic, there are still controversies about the oxidation and corrosion mechanisms of carbon materials. Probably, this is mainly because previous studies have not sufficiently explored the role of surface chemistry in the reactivity of these materials with the electrolyte. Since the reactivity (and surface functionalities) is positively related to the porosity, the understanding of the relation between these two qualities is of great relevance in the porous carbons’ development and applications.

The analysis of the effect of surface chemistry on the reactivity with the electrolyte can be divided into addressing aqueous and organic electrolytes. Regarding the aqueous electrolytes, extensive studies have been conducted on their interaction with carbon materials, focusing on ion adsorption processes during charge and discharge cycles and using various *in situ* techniques such as solid-state NMR, an electrochemical quartz crystal microbalance, electrochemical dilatometry, and scanning electron microscopy.^960,1003–1009^ These investigations have been very useful to explain fundamental aspects of the mechanisms of ion transport within the porosity and pseudocapacitance. Recent findings obtained using electrochemical dilatometry and scanning electron microscopy have confirmed that electrodes undergo volumetric changes during charge and discharge, which are determined by an ion radius. Significant differences have been observed between cation and anion adsorption, with anions being less efficiently adsorbed.^1003^ Additionally, the electrochemical quartz crystal microbalance has proven to be a valuable instrument for understanding the importance of ion desolvation in the supercapacitor performance.^1008^ It is also very important to highlight the relevance of the proper electrolyte wettability of the carbon electrode surface to ensure a stable operation of the capacitor.^1003^ For this reason, electrochemical preconditioning should always be applied before material characterization or its practical application. If this is not done, changes in the performance of the capacitor over time can occur that might lead to the misinterpretation of experimental results or, from an application point of view, to a reduction in the lifespan of the device.

For the reactions that may occur between an aqueous electrolyte and carbon material, the oxidation potential of carbon to CO_2_ is 0.207 V vs NHE (reaction 1) (this potential corresponds to the standard reduction potential of CO_2_/C). Therefore, at potentials higher than this value, the reaction is thermodynamically favored.^992^

1a $$C\left(\right)+2H_{2}O\to CO_{2}+4H^{+}+4e^{-}$$

And it is proposed that this reaction occurs in different steps:

2a $$C\left(\right)+H_{2}O\to C\left(\mathrm{OH}\right)+H^{+}+e^{-}$$

3a $$C\left(\mathrm{OH}\right)\to C\left(O\right)+H^{+}+e^{-}$$

4a $$C\left(O\right)+H_{2}O\to C\left(\mathrm{OOH}\right)+H^{+}+e^{-}$$

5 $$C\left(O\right)+H_{2}O\to CO_{2}+2H^{+}+2e^{-}$$

Since the last reaction is highly irreversible and requires a high overpotential,^992,1010^ carbon materials can be used as electrodes under positive polarization conditions that are superior to those predicted by the thermodynamic potential. Carbons can react with the electrolyte either under potentiostatic or potentiodynamic conditions.^605,1011,1012^ However, a corrosion rate observed under potentiodynamic conditions is higher than that occurring at a constant potential, which is associated with an oxidation/reduction cycle of oxygen functional groups.^960,970,990^ Under potentiostatic conditions, oxidation readily forms a superficial layer of functional groups, which, due to the irreversibility of the gasification reaction 5, reduces the oxidation/corrosion rate and may serve as a passivating layer on the carbon surface.^1013^ However, under potentiodynamic conditions, oxygen groups are formed during positive polarization that can subsequently be reduced under cathodic conditions, generating CO or CO_2_ and, possibly, H_2_,^960,970^ through the following reactions:

6 $$C\left(O\right)+2H^{+}+2e^{-}\to \mathrm{CO}+2C\left(H\right)\;\mathrm{or}\;\mathrm{CO}+C\left(\right)+H_{2}$$

7 $$C\left(O_{2}\right)+3H^{+}+3e^{-}\to CO_{2}+3C\left(H\right)\;\mathrm{or}\;CO_{2}+C\left(\right)+C\left(H\right)+H_{2}$$

At these conditions, the gasification rate is higher because the electrode remains at high potentials for a short time. As a result, a high concentration of oxidized sites is not generated, and when the potential is reduced, new free sites can form to participate in a new oxidation process.^1014^ Overall, the oxidation reactions that generate oxygen functional groups and their subsequent reduction correspond to the global carbon gasification reaction with H_2_O molecules. These oxidation/reduction cycles, which lead to a mass loss, have been found using an electrochemical quartz crystal microbalance (Figure 33D, part a),^960,970^ showing that the reaction rate under potentiodynamic conditions is similar to typical carbon gasification rates with steam (Figure 3D, part b). Thus, based on the measured mass variation during oxidation/reduction cycles between −0.1 and 1.4 V NHE in 1 M H_2_SO_4_, a reactivity of 0.75 h^–1^ was calculated, which is close to the typical reactivity values observed in steam carbon gasification at 800 °C.^969^

These findings using the electrochemical quartz crystal microbalance have recently been confirmed by employing mass spectrometry coupled with electrochemical measurements, in a study on the electrochemical expansion of graphite in different electrolytes.^1015^ The results showed that the anodic expansion of graphite (closely related to the electrochemical oxidation of this material) is significantly dependent on the pH of the electrolyte. In alkaline media, the degree of surface oxidation is important, although significant carbon gasification (i.e., CO_2_ evolution) has not been found. On the contrary, in acidic media, graphite gasification is more pronounced, with relevant CO_2_ evolution under the anodic conditions. Additionally, an H_2_ evolution under the cathodic conditions is accompanied by the CO_2_ evolution, which aligns with the reactions proposed under the reduction conditions, based on the results obtained from the electrochemical quartz crystal microbalance. The effect of pH on the oxidation of other type of carbon materials has been also found by mass spectrometry coupled with electrochemical measurements.^1016^ However, in that study, only the oxidation behavior under positive polarization conditions was analyzed using a HClO_4_/NaClO_4_ electrolyte at various concentrations.^1016^ This study suggested that carbon oxidation is influenced by the pH and that, at acidic pH, the initial step in carbon corrosion is an acid-catalyzed hydration reaction with a carbocation formation. This reaction is favored at edge sites of the material, where a proton attack on a sp^2^ carbon atom, followed by the reaction of a water molecule with the generated carbocation, initiates electrochemical oxidation. These findings would explain the high electrochemical stability of boron-doped diamond materials, as they contain sp^3^-carbon atoms, in which this reaction cannot take place. A study performed using accelerated durability tests of carbon materials and employing mass spectrometry coupled with the electrochemical experiment has shown that the most stable materials are those with a higher concentration of sp^2^ carbon atoms and a lower content of oxygen, although the authors emphasized the influence of CO_2_-type functional groups.^1017^

Additionally, the fundamental aspects of the electrochemical oxidation of carbon materials have been studied using different electrolytes.^968,1015^ These studies indicated that oxidation can occur through two mechanisms that involve either direct oxidation of the carbon material due to electrode polarization (which essentially corresponds to the reactions explained before), or indirect oxidation—through the oxidizing species in a solution formed from the electrolyte.^968^ Thus, the degree of oxidation depends not only on electrode polarization but also on the chemistry of the electrolyte used and on the operating temperature.^1018^ The latter parameter is particularly significant for the capacitor performance, as it affects an ion transport, the dielectric constant of the electrolyte, and the reactivity between the electrolyte and the electrode. However, insufficient attention has been paid to this experimental parameter, making further research on this topic necessary.

Therefore, the oxidation process of carbon materials is a complex phenomenon that still requires more detailed studies. These studies should focus, for example, on the development of experimental procedures that permit the measurement of the electrochemical reactivity in a way that can be compared among different materials, electrolytes, and temperatures. In this sense, the research on an accelerated aging of carbon materials in supercapacitors^1019,1020^ and self-discharge measurements^1021–1023^ could serve as the basis for establishing the aforementioned experimental procedures that may assess, correctly and widely comparable, the properties of the materials for this application. These studies should focus on carbons with different structural characteristics, porosity and surface chemistry, as these parameters will determine the reactivity and the selectivity of the oxidation products generated and will impact their real applications as electrodes in electrochemical devices.

Capacitors that use organic electrolytes and ionic liquids can operate at a wide voltage window, storing more energy compared to that in aqueous electrolytes.^911^ These electrolytes, particularly the organic ones, are widely used in both research on supercapacitors and industrial applications. They can undergo decomposition when exposed to high voltages or elevated temperatures,^997,1018^ with these parameters exponentially accelerating the electrolyte decomposition. Several studies have analyzed electrolyte decomposition using various *in situ* techniques,^998–1000^ and the influence of functional groups, trace amounts of water, and inorganic elements on electrolyte degradation is well-known.^996^ However, most of these studies focus on the degradation of the organic electrolyte and even examine the effect of a capacitor architecture,^1024^ without giving much attention to the properties of the carbon materials. The most important conclusions from these studies (primarily focused on propylene carbonate-based electrolytes, which are the most used) suggest that both the positive and negative electrodes can undergo reactions that lead to the formation of propylene polycarbonate deposits. In the negative electrode, degradation seems to be strongly influenced by traces of water that remain adsorbed on the oxygen functional groups, from which it cannot be easily removed.^1000^ However, other studies have not reported the same influence of traces of water on the electrolyte decomposition when measuring the amount of gas detected.^1025^

As mentioned, these studies have not paid sufficient attention to the surface chemistry of the carbon materials, despite the fact that it is well-known that propylene carbonate can react with oxygen functional groups, such as phenols and carboxylic acids, forming hydroxy-alkyl derivatives and releasing CO_2_. Consequently, the presence of oxygen functional groups can significantly influence a solvent stability, as confirmed in a study that analyzed the effect of carbon surface chemistry on the degradation of the capacitor.^930^ This study found that the exhaustive purification of the carbon material (through removal of inorganic impurities that may catalyze electrolyte decomposition) and the elimination of oxygen functional groups by high-temperature hydrogen reduction resulted in materials with a high stability, even under extreme voltage and temperature conditions. Furthermore, it has been demonstrated that oxygen functional groups and edge sites in carbon materials have the greatest influence on the reactivity of the positive electrode with the electrolyte,^1027^ whereas electrolyte reactions with the negative electrode are more related to the graphene basal planes.^1026^ Figure 34A includes a scheme proposed for carbon material degradation under positive and negative polarization conditions^1026^ and Figure 34B includes a plot showing the positive correlation between reactivity of the positive electrode and functional groups and edge sites.^1027^ Understanding the fundamentals of the reactivity between carbon materials and the electrolyte, as discussed above, is essential for designing strategies to improve the overall performance of electrochemical capacitors. One of the main conclusions is that oxygen functional groups play a crucial role in electrolyte and electrode degradation (as well as other components like the current collector). Thus, instead of incorporating electroactive groups, the improvement of the device could be achieved by modifying, blocking, or efficiently removing these sites, leading to materials with a high electrochemical stability capable of operating at wider voltage windows and higher temperatures. This would constitute a significant breakthrough in the field of supercapacitors. The most common strategies for tailoring the surface chemistry of carbon materials for this purpose include the removal of oxygen functional groups through hydrogen reduction, the incorporation of functionalities or molecules that inhibit the redox reactions of oxygen functional groups, the introduction of heteroatoms such as nitrogen or phosphorus, or decrease in the concentration of edge sites (which involves structural modifications).

**Figure 34:**
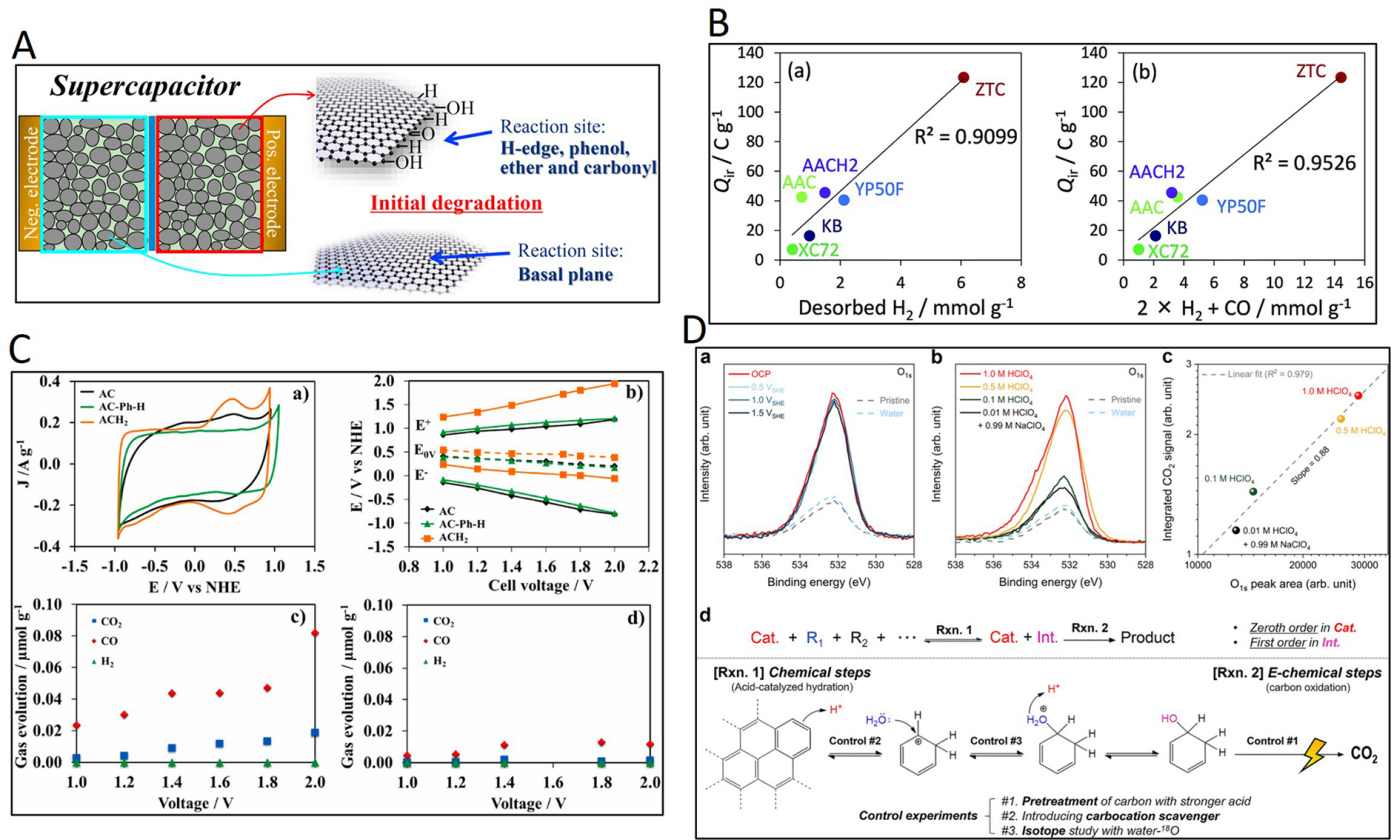
A) General scheme for carbon material degradation under positive and negative polarization conditions. Reproduced with permission from ref.^1026^ Copyright 2020, Elsevier; B) (a) The plot of *Q*_ir_ against the amount of H_2_ evolution. (b) *Q*_ir_ versus the sum of the entire H-edge sites and the total CO evolution up to 1800 °C. Reproduced with permission from ref.^1027^ Copyright 2019, Royal Society of Chemistry; C) Cyclic voltammograms of AC, AC-Ph-H, and ACH_2_ materials recorded in a three-electrode cell at 2 mV s^–1^ in 2 mol L^–1^ Li_2_SO_4_. Fifth scan recorded (a). Potential limits of positive (*E* ^+^) and negative (E^–^) electrodes during cycling from 1.0 to 2.0 V of AC-, AC-Ph-H-, and ACH_2_-based symmetric supercapacitors in 2 mol L^–1^ Li_2_SO_4_. The *E*_0 V_ values (dashed lines) correspond to the electrodes potential when the working voltage is shifted to 0 V before each change of maximum voltage (b). Evolved gases amounts while polarization from 1.0 to 2.0 V of symmetric supercapacitors built by ACH_2_ (c), and AC-Ph-H (d). Reproduced with permission from ref.^1028^ Copyright 2025, Elsevier; D) Proposed reaction mechanism of carbon corrosion. (a) XPS O 1s spectra of carbon samples collected after polarization at constant potentials (1.5, 1.0, and 0.5 V_SHE_ or OCP) for 15 min in the Ar-saturated 0.5 M HClO_4_ electrolyte. For comparison, the XPS O 1s spectra of the pristine and H_2_O-treated samples are also shown. (b) XPS O 1s spectra of carbon samples collected after immersion (i.e., OCP) for 15 min in Ar-saturated 0.01–1.0 M HClO_4_ solutions or DI water. For the 0.01 M HClO_4_ solution, 0.99 M NaClO_4_ was added for a fair comparison with the electrochemical results. (c) Correlation plot between the CO_2_ evolution amount and oxygen coverage estimated from XPS after immersion of carbon samples. The amount of CO_2_ evolution was estimated by integrating the DEMS signals monitored during the first CV cycle. The correlation is plotted on a logarithmic scale, with the slope of the linear fit indicating the reaction order. (d) General regime for pseudo zeroth and first order kinetic reactions and the consequently proposed mechanism for carbon corrosion in acidic environments. The mechanism consists of preceding chemical steps (i.e., acid-catalyzed hydration) and subsequent electrochemical steps (i.e., electrochemical oxidation of carbon). Three suggested control experiments to support this mechanism are also provided. Reproduced with permission from ref.^1016^ Copyright 2024, American Chemical Society.

Regarding the treatment with hydrogen, it has been reported as effective in organic electrolytes,^930^ provided that other inorganic elements (which may catalyze or promote electrolyte decomposition) are properly removed. However, this treatment does not seem to be appropriate for aqueous electrolytes,^1028^ since the electrochemical oxidation of C–H groups can be important in such media (Figure 34C). This observation agrees with the proposed mechanism for the initiation of electrochemical oxidation in carbon materials and its dependence on the pH^1016^ (Figure 34D). Blocking redox processes associated with oxygen functional groups may be an effective strategy to enhance the stability in aqueous electrolytes.^926,1028^ In the first mentioned study, anionic metallacarboranes were incorporated; these are highly stable molecules that interact strongly with CO-type functional groups and increase the overpotentials for subsequent carbon oxidation or electrolyte decomposition reactions.^926^ Another study^1028^ reported that phenyl group grafting led to significant improvements in the device stability, possibly due to a combined effect of blocking the active edge sites in graphene sheets and decreasing the local pH at the interface (Figure 34C).^1028^ However, the latter argument seems to contradict the study that suggested that electrochemical oxidation initiates more easily at lower pH.^1016^

The *incorporation of heteroatoms* such as nitrogen (N) and phosphorus (P) (especially nitrogen) is a widely used strategy to enhance the stability of the carbon electrode/electrolyte system. Nitrogen functional groups generally exhibit basic properties, facilitating the interaction with acidic molecules, and making them valuable for various applications (as highlighted in this review). Numerous studies demonstrate the beneficial effects of nitrogen groups on the stability of carbons.^937,963,966,1029–1032^ (see Figure 32E,F as an example of this beneficial effect of N groups). From these studies, it is concluded that nitrogen functional groups contribute positively to the capacitance, electrical conductivity, wettability, and (most notably) stability. The stability in organic, aqueous, and ionic liquid electrolytes appears to be associated with the presence of nitrogen heterocycles (such as pyridine and pyrroles), quaternary nitrogen, and amide groups, whereas amine groups are not beneficial.^961,977,1032,1033^ Further research is still needed to better understand the effects of different types of nitrogen functional groups, as this could be one of the most versatile strategies to significantly improve commercial carbon materials, as well as new promising structural configurations, such as seamless monoliths.^963^ Regarding phosphorus functional groups, studies have also reported their effectiveness in increasing the stability of carbon materials.^162,1034,1035^ Phosphorus groups inhibit oxidation in carbon materials, what is in agreement with research on graphite oxidation inhibition via phosphorus addition, conducted during the 1980s.^81^ It has been proposed that the phosphorus groups block active sites.^1035^ However, recent studies suggest that the mechanism involves the active participation of the phosphorus groups, which experience progressive oxidation from less-oxidized to more-oxidized states, and a slow oxygen transfer from highly stable oxidized phosphorus sites to the neighbor carbon atoms.^162^ However, the oxidized phosphorus groups have a strong acidic character that can be detrimental to the stability of the propylene carbonate containing electrolytes.

Reducing the concentration of edge sites is a recent strategy for the development of highly stable carbon materials,^932^ which can be further enhanced by applying some of the previously mentioned treatments. One approach is the fabrication of a seamless carbon electrode, consisting of a porous carbon monolith (for example, one material is commercially available as “CROUS®” by AION Co., Ltd.). Unlike conventional particle-based electrodes that require the use of a binder and a conductive promoter, this material eliminates interfaces between particles, thereby improving the electrode conductivity and decreasing reactivity.^1036^ This material exhibits a significant improvement in the stability after the incorporation of nitrogen groups through a low-temperature functionalization strategy.^963^ Another highly innovative strategy is based on synthesizing a mesoporous graphene-based material constituted by curved graphene sheets forming spherical pores. This structure results in a very low edge site concentration, providing the material of an exceptional stability at 60 °C and 3 V, as well as at 25 °C and 4.4 V in organic electrolytes.^964,965^ These characteristics make this carbon material one of the best-performing ones currently available. These carbons could revolutionize the supercapacitors market, provided that they are economically viable compared to conventional activated carbons. Additionally, boron-doped diamond materials exhibit extraordinary stability, likely due to the presence of sp^3^-hybridized carbon atoms. These materials could also be of a great promise for the future, if their production at a large scale (with a competitive price) and specific surface area are significantly improved.^932^

In summary, we can conclude that, in order to expand the range of applications for supercapacitors based on porous carbon materials, it is necessary to increase the operating voltage window, which inevitably leads to electrode degradation. A fundamental study of the reactivity of carbon materials with specific electrolytes is essential to understand how to optimize surface chemistry, porosity, and structure. From the analysis of surface oxidation processes, it can be inferred that the development of continuous carbon electrodes (with minimal edge-site concentration and reduced particle contact points), along with modified surface chemistry to further decrease the reactivity, could be the key to achieving the optimal materials. These carbons can also be used to design other capacitor configurations, like metal-ion capacitors in which the positive electrode is the porous carbon and the negative electrode is that for the corresponding metal-ion battery. These asymmetric configurations with a battery-type electrode open new possibilities for hybrid energy storage devices that can compete with conventional batteries. Understanding of all describe-above factors is necessary for the proper selection of the porous carbon.

#### 4.5.2 Porous Carbon Materials in Batteries

##### 4.5.2.1 Lithium-Ion Batteries

Carbon materials undoubtedly play a fundamental role in the field of batteries, although the most used are not porous carbon materials, except in very specific cases. Considering the wide application of carbon materials in batteries, in this review we will focus on lithium-ion and sodium-ion batteries, highlighting, with specific examples, the effect of porosity and surface chemistry of the material on energy storage and the need to further deepen our understanding of these properties.

The most representative example is that of Li-ion batteries. In this specific case, the most used material is graphite (theoretical storage capacity of 372 mAh g^–1^), because its behavior as a negative electrode is highly reliable and has not been surpassed by other materials, except for modifications incorporated into the graphite-based electrode to improve its performance. Among these, the addition of other components increases the stored energy (such as Si, whose theoretical capacity is 4212 mAh g^–1^ when forming Li_22_Si_5_,^1037^ as well as conductive additives or coatings that enhance the electrical conductivity and thereby improve a charging rate). Hard carbon materials (which refer to nongraphitizable carbon that contains narrow and closed porosity, and receive this name because most of them are “hard”^1038^) are used in high-power LIBs due to their fast charge/discharge rates. In other batteries currently under development or at an early industrialization stage, carbon-based materials are one of the best options as anode (such as Na-ion, K-ion, Li–S batteries, etc.). For the Li-ion batteries specifically, numerous reviews analyze the different aspects of these devices from the perspective of their various components. To further explore this topic, the references briefly mentioned below can be used as examples of the sources of available information.

The fundamental aspects of lithium batteries, including battery safety issues and comparisons with other batteries, have been recently addressed.^994,1039–1042^ Regarding electrolytes, the work by Li et al.^1043^ is a highly detailed review on new concepts in electrolytes, whose chemistry determines the formation of the Solid Electrolyte Interphase (SEI) that can facilitate the incorporation of new electrodes in batteries. In this sense, ion solvation in the SEI formation is particularly relevant, since the structure of the solvation shell determines interfacial chemistry.^1043^ This aspect is so crucial that electrolytes can be classified as strong or weak solvation electrolytes,^1044^ highlighting the importance of understanding ion solvation chemistry for the development of suitable electrolytes and materials. A promising concept for the development of new liquid electrolytes is *localized high concentration electrolytes*,^1044–1046^ where the addition of a nonsolvating solvent with Li ions to a high-concentration electrolyte reduces a viscosity, improves wettability, increases the working temperature window, and enhances electrochemical kinetics.^1044^ The reactivity of the electrolyte with the negative electrode determines the SEI properties, which is also relevant to the positive electrode, leading to the concept of cathode electrolyte interphase (CEI). There are several studies that include experimental findings on the SEI formation and the impact of the electrolyte and graphite material,^1047^ as well as theoretical aspects of the SEI growth.^1048^ The mechanisms of both electronic and ionic transports in the active phases and composite materials have been extensively discussed by Quilty et al.,^1049^ and the authors also explored strategies for developing new or improved electrodes. Concerning anodes, Cui et al.^1050^ collected studies on different types of anodes, including those based on intercalation reactions, alloy formation reactions, conversion reactions, and a Li metal. Qi et al.^1041^ described the behavior of various anodes with special emphasis on carbon materials, and Wang et al.^1051^ and Saju et al.^1052^ focused on biomass derived carbon materials and hard carbons for an energy storage. A very important technological issue is the coating of graphite particles with a carbon film, in order to control the electrolyte reactivity and thus achieve a decrease in the irreversible capacity and enhance the stability, allowing the use of PC as a solvent.^1053–1056^ These studies demonstrate the great importance of edge sites and surface chemistry in the controlled formation of SEI.^1057^ In particular, it has been shown that there is a correlation between the irreversible capacity and active surface area (ASA) during the first cycle and that carbon coating (either hard carbon or ground graphite) with a thin pyrolytic carbon film reducing this irreversibility. These results indicate that the carbon film prevents the diffusion of solvated lithium ions to the active sites, which allows the generation of an appropriate SEI. This study demonstrates the critical role of surface chemistry of a carbon material in this application, since it determines the reactivity with the electrolyte (as it has also been explained in detail in the case of supercapacitors).

Since one of the limitations of the Li ion batteries is their power, significant attention has been paid to the development of fast-charging batteries. Several reviews address this aspect from the points of view of the different components, such as an electrolyte, electrode, their reactivity, and the formation of the SEI, among others.^1041,1058–1060^ The slow kinetics of intercalation processes in graphite electrodes and the possibility of Li plating during fast-charging make it necessary to develop new electrodes with a high electrical conductivity, high Li-ion diffusion rate, and fast insertion kinetics. Increasing the electrical conductivity of electrodes is essential to fully utilize the electrochemical properties of active phases, as mentioned above. Enhancing the electrical conductivity of the electrodes and the generation of a conductive network that increases contact points between particles are two key strategies for improving the battery performance and for increasing its power.^1058^ In this sense, the synthesis of seamless carbon monoliths or monolithic materials with effective particle interactions can be crucial for improving negative electrodes. To increase a Li ion diffusion rate, different strategies have been proposed, including the generation of porosity in graphite particles or the use of porous carbons.^1059^ For example, the effect of the activation of graphite with KOH to improve the battery power has been studied.^1061^ The results show that this treatment did not modify the capacity of the material and the irreversible capacity, although a significant enhancement in the power was achieved. The authors attributed this improvement to the creation of holes at the surface that increased the intercalation and deintercalation rates of Li ions, while reducing the Li ion diffusion path. However, porosity characterization showed that the KOH treatment did not significantly increase the porosity, in agreement with previous studies in which the effect of crystallinity on the development of the porosity by a KOH and NaOH activation was studied.^1062^ While KOH does not increase the porosity in graphite, it is effective in etching of the most reactive sites and, as observed, produces a more ordered material.^1061^ This treatment seems to be effective in controlling the reactivity with the electrolyte and may reduce to some extent the particle size, the number of graphene layers in the particle, and increase the local order. These factors seem to be the main reasons for the observed improvements and reinforce the need to control the surface chemistry of the carbon materials to control the electrolyte reactivity and ensure a proper SEI formation.

Although the presence of highly developed porosity is associated with a very high initial irreversible capacity and a great difficulty in controlling SEI characteristics, publications on the use of activated carbons with highly developed porosity can be found. For example, Van et al. presented activated carbons prepared from rice husk by activation with KOH and NaOH with *S*_BET_ higher than 2000 m^2^ g^–1^ as negative electrodes in Li-ion batteries.^1063^ Even though differences were found depending on the activation conditions, the results showed a significant decrease in the capacity during the first cycles, suggesting that these materials may not be very suitable for a commercial use. A detailed characterization of the chemical composition is also necessary since this precursor contains Si, and the presence of this element in the final product may also contribute to charge storage.

Regarding the possibility of using porous carbon materials with or without the incorporation of heteroatoms such as O, N or P, these do not generate a charge and discharge profile with a significant plateau, but rather show a region with a slope in the potential versus capacity plot that reduces the battery voltage. In addition, an appropriate initial Coulombic efficiency cannot be achieved, which limits the application of these materials.^1059,1064,1065^ For example, the incorporation of nitrogen heteroatoms to a hard carbon derived from olive pits showed that they exhibited a high irreversible capacity in the first charge/discharge cycle, which is a major disadvantage compared to graphite, and made them inappropriate for applications requiring the high energy storage. However, these materials had a high rate capability making them adequate for high power systems.^1064^ It must be noted that, in that study, the authors have used carbons with high N contents (two samples with 4.1 and 12.7 at. % N) and different N groups that might have different reactivity with the electrolyte (as explained in the previous section). Thus, it would be interesting to study in more detail the effect of the type and quantity of the N-groups on the electrochemical behavior. The authors also emphasized that the analysis of materials should not only focus on the measurement of the capacity and cyclability, but that there are other practical parameters of great importance that are frequently disregarded in the literature, such as a voltage hysteresis, energy efficiency and Coulombic efficiency. We agree with the authors that a detailed and correct evaluation of the electrochemical behavior is essential to understand the applicability of carbon materials; and this is a common drawback in the literature.

The sloping region in the highest voltage regime observed in the typical electrode charging experiments is a consequence of the contribution of Li ion adsorption on the surface, defects, or heteroatoms (which is closely related to the energy storage mechanism in electrochemical capacitors). However, there are significant controversies regarding the mechanism of the charge/discharge process in these types of materials. Thus, different mechanisms are proposed based on the pore insertion, adsorption, and filling processes that may occur, which are named as pore insertion-filling, adsorption-pore filling, or adsorption-insertion mechanisms.^1066^

A recent study of the Li and Na ion insertion mechanism in porous carbons (hence, materials of low structural order) showed that the adsorption of Li ions is the most important contribution to lithium storage in the whole voltage range studied and that Li insertion occurs at low potentials, although it is difficult to clearly separate both mechanisms as a function of the potential (Figure 35A).^1066^ On the other hand, this study also demonstrated that the C–H sites exhibit a high reactivity with lithium, so that, during discharge, when low potentials are reached, the C–H bonds can break and the dangling sites formed can react with lithium, which reduces the stability and the rate performance of the material.^1066^ Therefore, although the porosity can increase the capacity and reduce the diffusion distance of lithium ions, it reduces the Coulombic efficiency by providing a higher density of active sites for the reaction with the electrolyte. Although hydrogen atoms in the material structure can provide additional sites for the insertion and adsorption of lithium ions, they are also responsible for the generation of a greater number of active sites for the decomposition of the electrolyte. However, these conclusions are different from those recently published by Zhou et al.^1067^ From that study, a correlation between the initial Coulombic efficiency and the presence of the C–H groups in hard carbon was reported, and the authors propose that a surface rich in this type of groups favors the formation of thinner and more conductive SEI (Figure 35B). Zhou et al. studied hard carbons with different H and O contents, obtained through heat treatments in Ar, H_2_, or oxidation with H_2_O_2_. They found that the oxygen groups facilitate the reaction with the electrolyte; thus, the incorporation of the C–H groups improves the behavior of the materials. Therefore, the elimination of the oxygen groups is undoubtedly beneficial (see the previous section on supercapacitors). Thus, these results, which may seem contradictory, might not be that inconsistent, since the analysis of surface chemistry of carbon materials must be carried out from a global perspective, considering the reactivity of the carbon materials with the electrolyte, which is determined by their structure, porosity, defects, and the presence of various heteroatoms. In this regard, it has been demonstrated that the elimination of active sites, related to material defects (measured as ASA by O_2_ chemisorption) with which the electrolyte can react, is essential for achieving improved materials (Figure 35C).^1057^ However, work still needs to be done to identify the specific contribution of the different factors to the reactivity, taking into account the complexity of the process and the carbon materials. Consequently, a proper design of the carbon materials in terms of particle size, structural order, porosity, and surface chemistry, together with an adequate control of reactivity toward the electrolyte, are crucial aspects for achieving improved carbons. As highlighted in the previous section, studying the reactivity of materials with electrolytes is essential for understanding the physical and chemical processes that occur and that would be of great help for tailoring the characteristics of carbons for the specific application.

**Figure 35:**
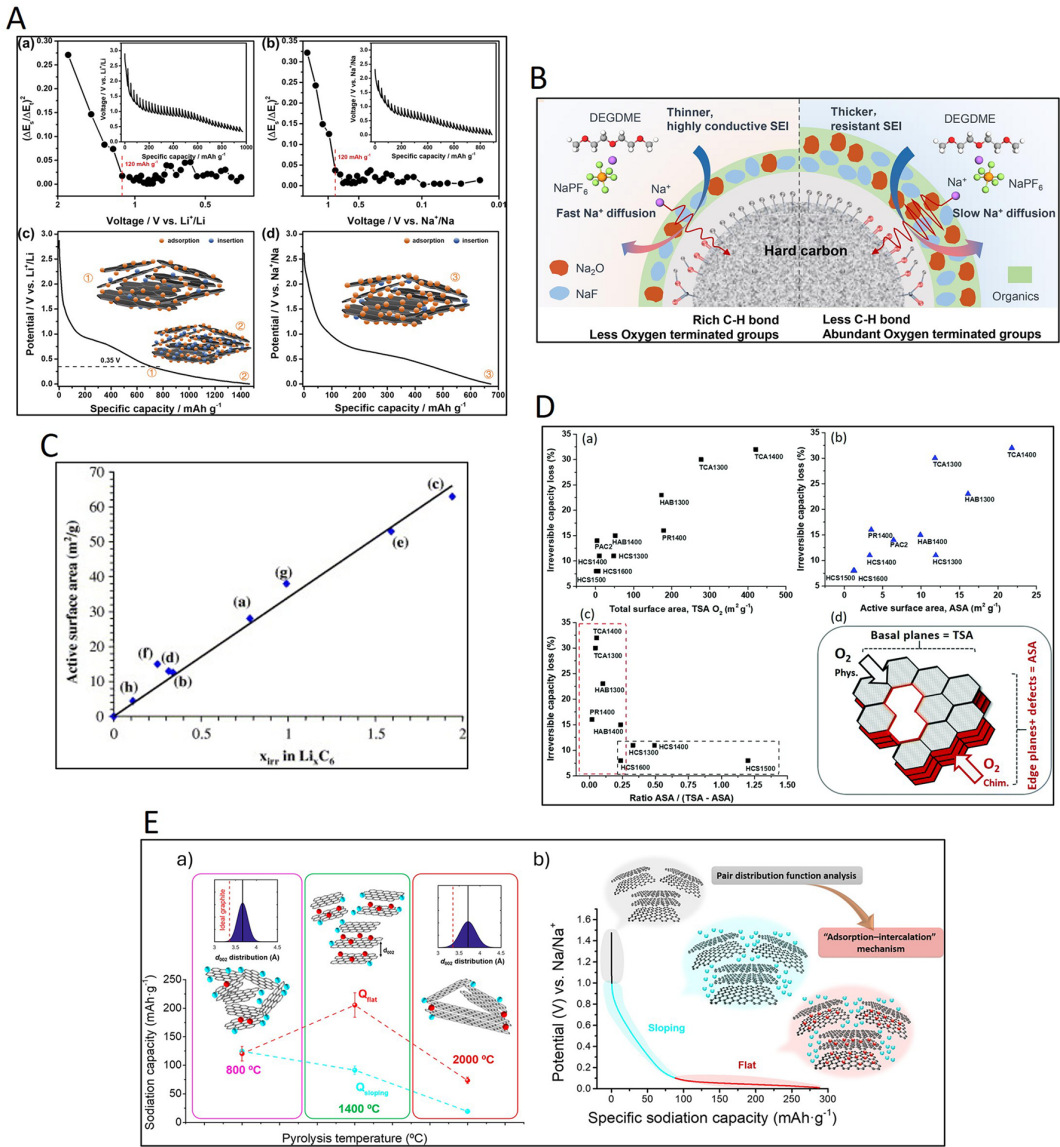
A) Apparent diffusion coefficient (insets for the GITT potential profiles in the first cycle) of porous carbon ((a) for lithium and (b) for sodium), and potentiogram and schematic of (c) lithium and (d) sodium storage. Reproduced with permission from ref.^1066^ Copyright 2020, American Chemical Society; B) Scheme of the Na ions diffusion process within the electrode/electrolyte interphase of HHC- and LHC-electrode (i.e., electrodes with high and low H contents, respectively). Reproduced with permission from ref.^1067^ Copyright 2025, Elsevier; C) Relation between the active surface area and the irreversible capacity *x*_irr_ of graphite samples ball-milled in different conditions, or ball-milled and subsequently coated by pyrolytic carbon. (a) 10 h in vacuum; (b) 10 h in vacuum + pyrolytic carbon deposition; (c) 10 h under H_2_; (d) 10 h under H_2_ + pyrolytic carbon deposition; (e) 10 h under O_2_; (f) 10 h under O_2_ + pyrolytic carbon deposition; (g) 20 h in vacuum; (h) 20 h in vacuum + pyrolytic carbon deposition. Reproduced with permission from ref.^1057^ Copyright 2005, Elsevier; D) Relationship between the 1st irreversible capacity loss and (a) total surface area (TSA O_2_), (b) active surface area (ASA), (c) ratio ASA/(TSA O_2_-ASA). (d) Representation of hard carbon structure containing the active surface (edge planes and defects) and the basal planes surface (TSA-ASA). Reproduced with permission from ref.^1068^ Copyright 2022, Royal Society of Chemistry; E) a) Schematic of the main conclusions of the study. Flat and sloping capacities are shown for three selected carbonization temperatures, and insets show the interplanar spacing distribution as estimated from PDF (pair distribution function) measurements and schematic depictions of the structure in each case, showing the contribution of sloping and flat regions. Reproduced with permission from ref.^1069^ Copyright 2019, American Chemical Society. b) Scheme for the proposed “adsorption-intercalation” mechanism. Reproduced with permission from ref.^1069^ Copyright 2019, American Chemical Society.

##### 4.5.2.2 Na-Ion Batteries

The second example of batteries discussed in this review is that of Na-ion batteries because, on one hand, it is a promising technology to complement other batteries and, on the othe hand, because the chemical processes involved and the properties of the carbon material suitable as negative electrode are substantially different from those of lithium-ion batteries.

To understand the important differences between the two types of batteries, it is useful to review the fundamental aspects of the intercalation processes of group 1 elements into carbon materials. The energy storage in carbons and in the context of group 1 and 2 elements, such as Li, Na, K, Mg, or Ca, is based on the intercalation of ions under negative polarization conditions and the formation of intercalation compounds, whose synthesis and properties are known since the beginning of the 20th century in the case of potassium intercalation compounds.^1070^ Research in this field was intense in the 1980s and 1990s, and different aspects of the intercalation processes in carbon materials with different structures were studied, leading to the determination of the influence of this property from theoretical and experimental viewpoints. Two of the earliest reviews on this topic were published in 1975 ^1071^ and in 1987.^1072^ Interestingly, the latter covers the interaction of Na and K in carbon materials of different structures, paying attention to coke. It is because the main objective of that review was to compile existing research on the interaction of alkaline elements with metallurgical cokes and with anodes and cathodes used in the manufacture of aluminum.

The thermodynamic properties of the formation of the intercalation compounds determine the feasibility of intercalation and, consequently, the application of carbons in the corresponding batteries. It is well-known that Li and K easily intercalate into graphite, forming the corresponding intercalation compounds, and the reversibility of their formation is the basis for rechargeable batteries. In the case of Na, intercalation is poor and not thermodynamically favored, making graphite-based anodes not useful for these batteries. Although the intercalation chemistry of group 1 elements has been understood for years, a recent study provides valuable insights into the differences in the intercalation of Li, Na, and K.^1073^ It analyzes the thermodynamic stability of the formation of the compounds with respect to the structural deformation of graphite and the different contributions to the bonding energy.^1073^ In summary, the study concluded that the element that deviates from the expected trends is Li, in which the covalent contribution to the bond with graphite, together with the greater contribution of the van der Waals energy between the graphene layers, results in a greater stability of the intercalation compounds. This is essentially one clear example of the differences in the chemical behavior of a head of a group element compared to the rest of the elements. Consequently, the intercalation of sodium ions in graphite, which acts as the negative electrode of the battery, results in a very poor storage, making this material unsuitable for this application. However, intercalation of Na in other types of carbon materials is feasible.

The intercalation of Na is related to the structural order of a carbon material, with lower structural order leading to easier and higher degrees of intercalation. This has been interpreted considering that the presence of electron-accepting defects modifies the position of the Fermi energy of the carbon material, in a way that more disordered materials have a lower Fermi energy value and a higher intercalation capacity.^1072,1074^ It was also found that a fraction of Na remains adsorbed within the porosity of the carbons used. That porosity is there due to their low structural order, and it might be even filled out by sodium.^1072^ Additionally, the electrochemical intercalation of Na was studied in carbons with different structural orders (similar to current experiments on Na-ion batteries), revealing that regular staging did not occur.

The differences in the intercalation capacity of Na and K have also been shown to have a significant effect on the development of porosity as a result of a chemical activation, using the corresponding hydroxides. The KOH activation of carbons with higher structural order is more effective than with NaOH due to the contribution of the intercalation step during the activation process.^1062^ In this sense, it has been demonstrated that differences in the intercalation of these elements determine the porosity development of carbons originating from precursors with different degrees of structural order.^1075^

Furthermore, it is known that Na intercalation is favored in the presence of other electronegative elements such as oxygen, chlorine, etc., leading to a phenomenon of cointercalation.^1076^ This research agrees with more recent observations that the cointercalation of partially solvated sodium ions in graphite is thermodynamically favorable, suggesting that sodium-ion batteries incorporating these cointercalation processes could be feasible using graphite as an anode.^1077,1078^ This strategy could be applied to achieve ultrafast charging rates in sodium-ion batteries with nongraphitizable carbon-based electrodes such as hard carbons. The cointercalation phenomenon is a versatile tool for modifying electrode properties that can be extended to different electrode/electrolyte combinations, and is a subject that requires extensive research to identify systems with potential practical applications.^1079^

Consequently, the fundamentals of the intercalation of group 1 elements can help to interpret the experimental results of sodium-ion batteries (and other types of batteries), allowing for the design of optimal materials. These principles can be summarized as follows: (i) sodium intercalation in graphite is not favorable, whereas it can occur in carbons with a lower structural order, which is related to the lower Fermi energy of these materials; (ii) the presence of electron-accepting sites promotes intercalation (for this reason, boron-doped graphite appears to exhibit a higher degree of intercalation); (iii) in addition to the sodium atoms inserted into the carbon structure, adsorption also occurs, forming clusters that can fill the carbon porosity; (iv) the cointercalation of sodium with electronegative elements is indeed favorable, as it stabilizes the intercalated species. Based on all these observations, it follows that nongraphitizable carbon materials with a low structural order exhibit a good performance as electrodes in sodium-ion batteries, where insertion and intercalation processes occur under reduction conditions during battery charging and are reversible. Moreover, the possibility of using a suitable solvent that allows Na ions to form partially solvated species stabilized on the surface of these carbon materials opens new possibilities for the development of this type of battery. Thus, in this specific case (although it may extend to other ions), porous carbon materials with very narrow porosity and favaorable surface chemistry are the most appropriate for this application. However, these emerging aspects still need to be explored in more depth, despite an extensive research being done on this topic. In this regard, carbon materials with a low structural order, having some porosity that allows the access of partially solvated species, with a low reactivity to prevent uncontrolled SEI growth, and a high electrical conductivity, are the most suitable candidates for sodium-ion batteries. This is why hard carbons, among other porous materials with low crystallinity, are extensively studied in this field, and there are reviews covering their synthesis and the effect of their properties on the electrochemical performance.^1052,1080–1082^ When these low-structural-order materials are used as negative electrodes in these batteries, different steps in the variation of voltage can be observed in galvanostatic charge measurements: an initial sloping region followed by a nearly constant voltage zone. These stages are interpreted considering insertion and adsorption mechanisms, with the possibility of the deposition of metallic clusters, as previously mentioned in the case of lithium. However, it seems that the assignment of each region to a major mechanism remains controversial in explaining the Na storage in hard carbons. Several models have been proposed to describe these stages, including the insertion-adsorption model, adsorption-insertion model, adsorption-pore filling model, and adsorption-insertion-pore filling model.^1052,1066,1083–1085^ A recent study^1066^ suggested that adsorption is the dominant mechanism for the storage of both lithium and sodium in porous carbons in the whole potential window studied (Figure 35A). Using ^7^Li NMR spectroscopy, the authors detected adsorbed and inserted lithium but no other forms of lithium, supporting the adsorption-insertion mechanism. This interpretation was extrapolated to sodium considering the observed electrochemical behavior, although no spectroscopic measurements were presented. However, a later study using pitch-derived hard carbon, where porosity was controlled by the KOH activation and analyzed with ^23^Na NMR, indicated that the main mechanism in the sloping region is adsorption-insertion, while in the plateau region, it is adsorption-filling.^1083^ The study concluded that the optimal material should contain a closed porosity (defined by the authors as pores smaller than 1 nm), to allow the incorporation of desolvated sodium, and have a low mesopore volume, to prevent irreversible adsorption or decomposition of solvated sodium ions. These examples, with quite different conclusions, highlight that research on sodium-ion battery materials is still in its early stages compared to lithium-ion batteries, with many aspects requiring further detailed investigation.

In any case, numerous studies can be found on the effect of the properties of hard carbons, and similar materials, in their application as negative electrodes for sodium-ion and lithium-ion batteries. The properties studied are analogous to those analyzed in the previous sections, specifically the porosity, structure, and surface chemistry (mainly heteroatom doping). Nevertheless, there are still no clear conclusions about the effects of these features, as research has not been done as detailed as that of lithium-ion batteries, and in some cases, somewhat generic and contradictory conclusions can be found, demonstrating the need for further progress in understanding this system.

Regarding porosity, a recent study has analyzed in detail the effect of the total specific area and active surface area (ASA) on the irreversible capacity of hard carbon in the sodium-ion batteries (Figure 35D).^1068^ The authors indicated that the porosity of the material is one of the main factors determining the irreversible capacity and that a correct and accurate determination of the porosity is crucial for predicting the behavior of carbon. In this study, the authors state that N_2_ adsorption at −196 °C is not appropriate for measuring the narrowest porosity and propose the use of O_2_ adsorption at −196 °C. Doing this, the authors found a good correlation between the irreversible capacity and specific surface area, and this correlation is similar to the obtained using CO_2_ adsorption at 0 °C up to 1 atm. The authors concluded that high specific surface area, high pore volume, and narrow pores contribute to the irreversible capacity. However, with this adsorbate (O_2_ at −196 °C), isotherms with hysteresis at low relative pressures were obtained, which are indicative of equilibrium issues during adsorption due to the presence of very narrow micropores. This suggests that porosity evaluation may be incomplete. As the authors propose, other adsorbates such as H_2_ might be more suitable. In this sense, we believe that high-pressure CO_2_ adsorption could be very useful to cover the whole range of relative pressures and to obtain a complete characterization of the different ranges of porosity of the material. One has to be aware that CO_2_ is not suitable as a porosity probe in the case of carbons rich in heteroatoms.^9^

Another very important factor highlighted by Beda et al.^1068^ is the presence of edge sites (that refer to active sites where the electrolyte can react), which concentration is also determined by the structure of carbon. These sites were measured using O_2_ chemisorption (ASA) and a very clear correlation between the irreversible capacity and ASA was found. In this sense, active sites are key to control the reactivity of the carbon electrolyte and must be correctly evaluated and identified. Through ASA, active sites can be accurately measured, although their chemical nature cannot be discerned; thus, further research in this area is necessary. The deviations in the irreversible capacity versus ASA plot in certain carbons were also found. They might be due to the presence of inorganic impurities in the hard carbon precursor, which have a strong influence on the synthesis and performance of carbon materials,^1086^ or due to the existence of heteroatoms such as N and S (the authors indicated that the specific effects of these elements on irreversible capacity are not clear). The concentration of active sites could be minimized through a structural modification that introduces a greater local order or a structure with a lower density of edge sites (such as the previously mentioned carbon mesosponge) or by an appropriate modification of surface chemistry. Another possibility recently described to minimize the effect of the reaction with active sites is the use of “sieving carbons” with abundant nanopores but with narrow pore entrances (which is similar to the concept of carbon molecular sieves, well-known in the carbon science literature) that impedes the formation of SEI inside the pores, but permits the formation of sodium clusters. In this type of carbons high capacities (up to 400 mAh g^–1^) were reported^1087^ and this strategy opens opportunities for the development of improved carbon materials. These results are in agreement with those described in ref.^1083^ However, some authors suggested that the presence of defects and active sites for Na ion interaction might be beneficial to increase the storage capacity.^1088^ These different conclusions show that it is necessary to study the electrochemical stability of the materials in depth, to verify the beneficial effect of these active sites and defects, as well as to use their proper and detailed characterization. In this sense, characterization under *in operando* conditions would be an important contribution to this topic.

Regarding the structure of carbon materials, this effect has been analyzed recently.^1069^ Figure 35E summarizes the most relevant findings from this work. The authors studied the effect of pyrolysis temperature on the local structure of hard carbon and its performance as a negative electrode in the Na-ion batteries. The results show that an increase in the local order that increases the size of the crystalline domains without reducing the interlayer distance below 0.37 nm (an interlayer space at which Na insertion becomes unfavorable), and that reduces the concentration of defects, is the most suitable approach for this application. It was proposed that the main storage mechanism for Na ions in hard carbon is the adsorption–intercalation mechanism, where Na intercalation occurs in the low, flat potential region. Similar findings regarding these structural requirements have been reported elsewhere.^1089^ In this context, all those processes that can improve the local order in a controlled manner, and that maintain the porosity (i.e., the interlayer spacing) above the indicated threshold but without the creation of an excessive microporosity, could be useful strategies to obtain the most suitable carbons. The use of catalysts for the graphitization of carbons could be a possibility for controlling the local order more effectively. One example of this approach can be found in ref.,^1090^ where iron is used as a catalyst to regulate the degree of graphitization of carbons. In any case, and as previously mentioned, a correct measurement of the porosity of carbon is necessary, using adsorbates that ensure a proper accessibility to the pores used during the electrochemical application. In this way, measurements using N_2_ at −196 °C (which is the one most commonly used in publications in this field) should be complemented with other adsorbates, for example CO_2_ (at 0 °C) or H_2_.

The effect of heteroatom doping in carbon applied for the Na storage has been studied mainly by analyzing the influence of the incorporation of functional groups containing O, N, P, S, B, and F. A recent review provides the main information available on this topic.^1091^ In summary, heteroatoms (in the form of the different functional groups) can mainly affect three fundamental aspects that influence the behavior of carbon materials as anodes in Na-ion batteries: (i) interlayer spacing: this parameter must be larger than that of graphite (but not below 0.37 nm) and the incorporation of heteroatoms can allow to control it. (ii) Interaction between the carbon material and Na ions: heteroatoms can create strong adsorption sites, which may be beneficial (by increasing the storage capacity) or detrimental (since a very strong interaction can increase the irreversible capacity during the first cycles). (iii) Reactivity with the electrolyte: heteroatoms modify the reactivity with the electrolyte, which is crucial for achieving the optimal performance. Therefore, the design of selectively doped carbon materials with the adequate functional groups that may allow to control the three aforementioned factors is one of the strategies for optimizing carbon materials for this application. It requires nanoscale-oriented synthesis supported by computational studies.

However, contradictory results still exist, as already mentioned in the case of the Li ion batteries. Specifically, some authors concluded that a high content of C–H terminations reduces the reactivity of the materials, allowing a better control of the SEI formation,^1067^ although opposing results have also been published,^1066^ for Na ion batteries. In any case, understanding the reactivity of the electrolyte with the carbon material, which is determined by its porosity, surface chemistry, and structure, is essential to understand the highly different results that can be found in the literature.

Although hard carbons are probably the most studied materials for the Na-ion batteries, they also have important disadvantages that may restrict their technological development. One of the disadvantages is their high cost due to the preparation conditions used.^1081,1082,1091^ The process involves a pretreatment with acid or base washing (which is crucial for obtaining a high-quality material and that is well-known since biomass is used to produce activated carbons), a precarbonization step and a subsequent thermal treatment at temperatures of around 1200 °C. The treatments applied and the duration of the process make hard carbon expensive. Additionally, the variability of biomass (not only because of different types that can be used, but also to differences between batches of the same material) makes challenging the reproducibility of the materials in large-scale manufacturing. This is a very important difference compared to the Li-ion batteries, where graphite is the most commonly used material and it has much more consistent and reproducible properties. All this makes the development of shorter, more efficient, and reproducible synthesis processes essential to scale up production and ensuring the availability of appropriate carbons for these batteries. Furthermore, the lack of sufficient biomass, with similar properties in different batches, constitutes a significant technological limitation, considering the huge amount of hard carbon that would need to be synthesized. Therefore, intensive efforts are needed to develop synthesis methodologies that can be industrially applied, while reducing costs, and ensuring reproducibility of the final product.

Following this, there are several aspects that still require further research. Processes that enhance the material structural order under milder reaction conditions could represent a significant advancement from an applied point of view. For example, the incorporation of species that catalyze graphitization could be very useful, as has been reported for carbons applied in the Li-ion batteries,^1090^ provided that the local structure of carbon, especially the interlayer spacing, could be controlled. Replacing conventional heat treatments with others that reduce processing times, increase yields, control the reactivity or regulate the structure could be very useful. Examples include hydrothermal treatments^1092,1093^ or microwave heating.^1094^ Probably, those materials with a low concentration of reactive sites (or without edge sites), with an appropriate porosity and high electrical conductivity, can represent a significant advancement in the design of the most appropriate carbon material for the Na-ion batteries. It is possible that the mesosponge and seamless porous carbons mentioned in the previous section may be very promising in this application.

### 4.6 Porous Carbons as Sensors

Recent focus on the properties of graphene and its broad applications based on its conductivity, along with a need to monitor the quality of our environment in terms of a quickly developing modern technology, directed the attention of scientists to porous carbons as feasible sensors for monitoring the content of air, water and also the state of human health. Even though their conductivity is 3 orders of magnitude smaller than that of graphene, and was reported to be in the order of 20–50 S cm^–1^,^1095^ it seems to be sufficient to exhibit changes upon exposure to various species affecting the population of holes/electrons. This is reflected in the change in the resistance or helps to exhibit irreversible or reversible redox reaction patterns. Those changes in the signal are generally used when porous carbons are applied as resistive or electrochemical sensors, respectively. Their significant assets, not existing in graphene, are their developed porosity combined with a relative easiness of their surface chemical modifications. Even though porosity is of paramount importance, we limit the focus of this section to the effects of functional groups and doped heteroatoms incorporated to the porous carbon matrix on sensing performances. The graphene-derived materials, including porous foams or specific geometric arrangements of the graphene sheets, are considered beyond the scope of this review.

#### 4.6.1 Gas Phase Resistive Sensors and Field-Effect Transistors

The research on the application of porous carbons for gas sensing is still in its infancy. Nevertheless, there have been reports addressing the importance of carbon surface chemistry, especially when target species are small molecule gases exhibiting distinct chemistry, either acidic or basic. For a resistive sensing, not without importance is the nature of charge carriers in the carbon matrix, which is strongly influenced by the presence of electron donating (alcohol (−OH), amines −NH_2_, ethers (−OR), and alkyl groups) or electron withdrawing surface groups (−NO_2_, −CN, −SO_3_H, cyano groups, esters, and −COOH).

##### 4.6.1.1 Ammonia Sensors

The most common sensors/detectors of gases of a basic nature are those targeting ammonia. NH_3_ is rather weakly adsorbed on carbons and reports addressing its adsorption mechanism clearly indicate the importance of surface acidic groups.^1096^ However, some of the carbon groups can chemically react with ammonia,^1097^ causing the change in the carbon conductivity/resistivity. Since for sensors, reproduction of a sensing signal is needed, the carbon surface needs to be “passivated” by prior exposing it to ammonia, followed by air purging. This process should lead to the “ammonia” stable surface demonstrated by Travlou et al.^1098^ In that work, wood-based carbon, BAX and its nitric acid oxidized counterpart (BAX-O) were tested as the sensors of ammonia in the concentration range between 100 and 500 ppm. By using the Mott–Schottky approach, the authors demonstrated that initial BAX was predominantly a p-type semiconductor with holes as main charge carriers, while BAX-O exhibited an n-type electron conductivity. Upon exposure to ammonia, due to its electron-donating character, both carbon chips exhibited changes in normalized resistance. It increased for BAX and decreased for BAX-O (Figure 36A). Nevertheless, a linear dependence on the ammonia concentration was found, supporting not only the detection but also sensing concept (Figure 36B).

**Figure 36:**
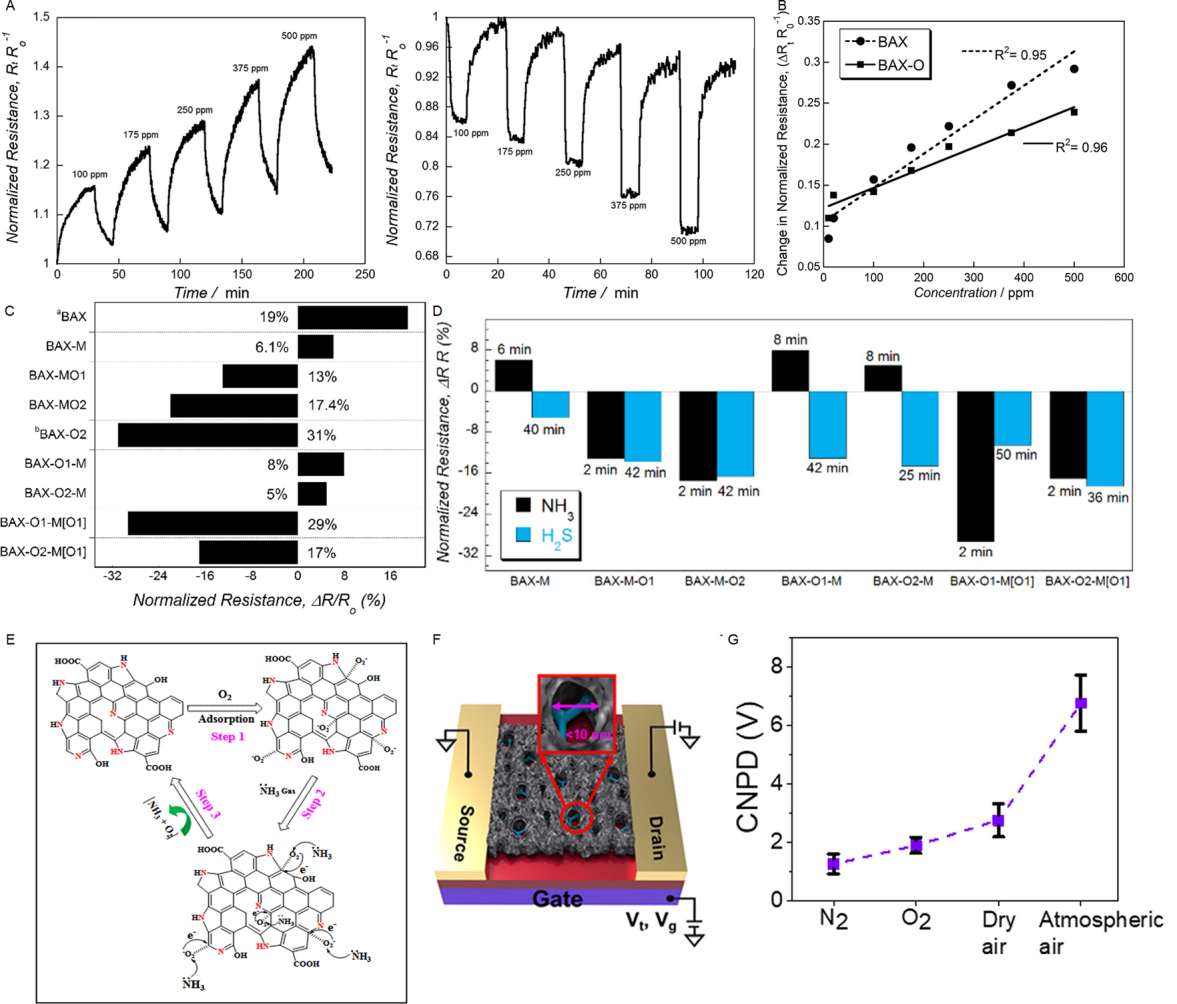
A) Response curves for BAX and BAX-O at various ammonia concentrations. Reproduced with permission from ref.^1098^ Copyright 2015, the Royal Society of Chemistry. B) The dependence of the change in the normalized resistance of the studied samples on the ammonia concentration in the challenge gas. Reproduced with permission from ref.^1099^ Copyright 2016, American Chemical Society; C) Comparison of the normalized resistance changes of all carbon samples tested upon exposure to 500 ppm of NH_3_. Reproduced with permission from ref.^1099^ Copyright 2016, American Chemical Society; D) Comparison of the selectivity of the carbon sensors in terms of the changes of their normalized resistance (sensitivity of the sensors). Reproduced with permission from ref.^1099^ Copyright 2016, American Chemical Society; E) The sensing mechanism. Reproduced with permission from ref.^1101^ Copyright the Royal Society of Chemistry; F) Device schematics of the a-CF-GFET sensor showing porous a-C on graphene. Reproduced with permission from ref.^1105^ Copyright 2021, American Chemical Society; G) Dependence of the CNPD (charge neutrality point disparity) value of the a-CF-GFET. Reproduced with permission from ref.^1105^ Copyright 2021, American Chemical Society.

Further modifications of that carbon surface were done based on an introduction of nitrogen through a thermal treatment with melamine at 450 °C (BAX-M).^1099^ That sample was further oxidized with 20% (BAM-M-O1) or 50% (BAM-M-O2) HNO_3_. Other samples were prepared by oxidation of BAX with 20% or 50% HNO_3_ (BAX-O1 or BAX-O2), treated with melamine (BAX-O1-M and BAX -O2-M) and further oxidized with 20% HNO_3_ (BAX-O1-M-[O1] or (BAX-O2-M-[O1]). These sequences of complex modifications resulted in distinct surface chemistries reflecting not only on interactions with ammonia but also on the type of a semiconductor represented by each sample. Even though those carbons had the predominance of holes as charge carrier and were p-type semiconductors in the as-received forms, the direction of changes in the normalized resistance upon exposure to ammonia/passivation was more complex than for the samples exposed only to the oxidative treatment (Figure 36C). The samples, modified with melamine in the last step showed an increase in the normalized resistance upon exposure to ammonia, while the samples oxidized in the last step showed decreases in the normalized resistance upon exposure to ammonia. This behavior was linked not only to the complex nitrogen chemistry but also to its changes upon interactions with ammonia in air. Amines and pyridines predominated on the surface of BAX-M, where melamine was used as the final treatment, and exposure to ammonia decreased the population of holes, increasing the resistivity. The situation of the oxidized and melamine treated samples and then reoxidized was more complex. The XPS analysis of the samples exposed to ammonia indicated the marked changes in surface chemistry, suggesting their conversion to n-type materials during “passivation”. Thus, on the ammonia exposed/passivated samples the population of pyridines decreased with an increase in NO_*x*_ and NH_2_ groups, indicating the formation of amino-nitropyridines where amino moiety is electron donating and nitro group is electron accepting species. Since pyridine defects are sensitive to ammonia^1100^ the plausible explanation was that the change in the conduction type occurred during ammonia sensing from *n* to *p*. Another scenario contributing to the complexity of the sensing mechanism imposed by the complexity of the carbon surface was based on the XPS results of the samples exposed to ammonia that suggested oxidation of NH_3_ to NO_2_ due to the presence of superoxide ions on nitrogen -modified carbons.^232^ That NO_2_ might have oxidized the carbon surface. Since the surface chemistry of nitrogen species and their acidity/basicity played a pivotal role in the ammonia sensitivity, their effect on H_2_S sensing was tested on the same carbons. Hydrogen sulfide is also an electron donating species but of an acidic character, as opposite to the basic one of ammonia. Interestingly, even though the sensitivity of the sensors in terms of the response was similar for some carbons, especially for oxidized ones, that sensitivity levels were reached after much longer time for H_2_S than for NH_3_ (order of magnitude) and this factor could suggest the selectivity to ammonia (Figure 36D). An important observation, addressed in the discussion when the comparison of the response to H_2_S and NH_3_ was analyzed, was that for melamine treated carbons resistivity decreased, which was an opposite trend to that for NH_3_. This behavior was linked to the small amount of water adsorbed in small pores of carbons and its contribution to H_2_S dissociation in a basic environment provided by the incorporated nitrogen groups. Formed HS^–^ could be oxidized to SO_2_ ^363^ and that formed SO_2,_ through its electron withdrawing character, could decrease the normalized resistance.

Nitrogen incorporated to carbon matrix was also indicated as important for sensing by Sadhanala et al.^1101^ Even though they indicated the importance of oxygen groups on the surface for the performance of the sensors in terms of a detection range, the sensitivity and response time increased with an increase in N content up to 7 at. %. The authors stressed the importance of the oxygen presence in the atmosphere for ammonia sensing. They suggested that its adsorption on the sensor surface extracted electrons from the N-doped carbon surface (n-type material) and thus reduced the conductivity/current. Nitrogen in a carbon matrix introduced the charge imbalance due to the electronegativity difference and the nearby carbon atoms acquired a slightly positive charge adsorbing oxygen (Figure 36E). Upon exposure to ammonia, the adsorbed oxygen was released and the conductivity/current increased. It was also noticed by analyzing the FT-IR spectra of the NH_3_ exposed samples that the intensity of the C–N band increased and the N–H was detected, implying the interactions of pyridinic N, −COOH, and OH groups with NH_3_.

The effects of sulfur incorporated to the carbon matrix on the ammonia sensing were investigated by Singh et al.^1102^ Carbons tested (C-1 and C-2), had a small content of surface sulfur (2.3 and 1.4 at. %, respectively) with its majority in reduced species such as bisulfides, thiols and mercaptans with small contributions of sulfoxides, sulfones and sulfonic acid. The oxidized sulfur species reacted with ammonia during chip “passivation”, and the reduced sulfur rather provided the p-type conductivity. Upon NH_3_ exposure, the resistivity of the chips increased and the difference in the response of the sensors was linked to different pore volumes and the extent of hydrogen bonding interactions of ammonia with surface polar groups. Thus, more porous and more chemically heterogeneous carbon (C-1) showed greater response than that of the smaller porosity and less polar surface (C-2). In the work of Singh et al., the role of specific S-containing groups was mainly discussed in the aspect of carbon surface passivation where chemisorption of ammonia took place, affecting its electrical response. The steric effects of bulky surface groups such as sulfones were indicated as increasing the response time of the sensors owing to the slower kinetics of ammonia adsorption due to a limited access to small pores.

Carbons doped by both N and S and containing considerable amounts of oxygen were also tested as ammonia sensors by Travlou et al.^1103^ As in their previous studies, carbons exhibited the p-type conductivity. The results suggested that the main mechanism governing the reversible electrical behavior involved the weak interactions of NH_3_ with the surface and electron/hole conductivity. The former included hydrogen bonding, dipole/dipole and dispersive interactions. The surface groups participating in the former were sulfoxides and sulfones, thiols, phenols, alcohols and carboxylic groups. Among N-containing groups, positively charged pyridine N-oxides and pyridines weakly interacted with the NH_3_ molecule. All of these were indicated as contributing to hoping of the charge carriers across the micro- and ultramicropores, providing alternate paths for a charge transport.

Generally, it is a common finding that the acidity of carbon introduced by various oxidation methods or by an incorporation of heteroatom-based groups increases the performance of ammonia sensors, especially for the detection of dry gas. Detection of ammonia on a carbon film derived from biomass and activated by phosphoric acid was investigated by Ranasinghe et al.^1104^ Since their carbon films were exposed to 1000 ppm of ammonia vapors, their results rather focused on the detection, and at very high concentrations, than on the sensing. Besides ammonia, the authors also investigated the carbon response to methanol, ethanol and acetone vapors. In all cases the resistance increased. As one could expect, phosphoric acid-activated carbon was likely rich in surface oxygen groups; however, the authors did not discuss the effects of surface chemistry or porosity on the response time or sensitivity. Apparently, the differences in the response measured for their target polar species could be attributed both to the extent of adsorption and to the kinetics of this process.

Agbonlahor et al.^1105^ built an activated carbon functionalized graphene field-effect transistor (a-CF-GFET) as a room-temperature ammonia-selective sensor with a sensitivity of 500 ppt in atmospheric air. They used plasma oxidation to oxidize carbon to increase the ammonia selectivity and to impose minimal p-doping. The oxidized carbon surrounded the nanometer size pores in graphene (Figure 36F). In the sensing mechanism, the carbon phase acted as Bronsted acid, leading to the acid–base Bronsted interactions around the pores and it resulted in the attraction of ammonia molecules to graphene and consequently in the n-doping of graphene. Importantly, oxidized carbon surrounding the pores decreased the amount of oxygen in contact with the graphene surface, improving the selectivity (Figure 36G) and limiting the gas sensing response to ammonia adsorption on graphene. The molecular-sieve functionality of the a-CF-GFET sensor was based on the ammonia selectivity of the a-C–graphene interface. It resulted from the effects of both the a-C-induced gas adsorption to the graphene/a-C interface and the size-induced sensitivity of the CVD graphene device. In this work, the adsorption of ammonia effect was based on speculations since the actual adsorption on the oxidized activated carbon-modified graphene composite was not measured. Since the response time of that sensor was 3 s, the authors suggested its application for medical diagnosis and environmental monitoring.

When detection of ammonia in moist air/ambient air was considered, conflicting results were reported. Travlou et al.^1106^ showed that on hydrophilic carbon rich in acidic oxygen and sulfur groups, sensing of ammonia was improved due to adsorption of water and solubility of ammonia in the adsorbed water film, followed by its protonation. Interestingly, on hydrophobic carbon, where the adsorption of water was limited, the sensor response was hardly affected compared to that in dry air. Also, Agbonlahor et al.^1105^ showed a good performance of their a-CF-GFET sensor in the high relative humidity (35–68%), where oxidized carbon was an important component of that sensing device (Figure 36G). On the other hand, Mutuma et al.^1107^ found that the ammonia response of the initial carbon spheres rich in carboxylic and OH groups was limited and significantly decreased upon the presence of humidity. Upon annealing of carbon at 600 °C, the sensitivity to ammonia increased and it was attributed to an increase in the surface area, a decrease in the amount of surface groups and thus to an increase in the adsorption of ammonia. That annealed carbon exhibited a good sensitivity to ammonia even at high humidity. Unfortunately, and what is quite common in sensor studies, no information on the surface chemistry evaluation was provided in that report.

Interestingly, the activated carbons were also tested as humidity sensors by Kathiravan et al.^1108^ Carbon was prepared from hen egg shells and activated in nitrogen between 800 and 1000 °C. The resistance of a carbon membrane linearly increased with an increase in humidity, which was attributed to a water uptake by carbon (Figure 37A). A target of this research was the application of that carbon in wearable electronics. At 35% humidity, the sensor was selective to water and its response was not affected by H_2_, NH_3_, CO, and C_3_H_6_O (100 ppm). The difference in the sensor response was linked to difference in the amount of water adsorbed; however, neither this aspect nor surface chemistry was investigated.

**Figure 37:**
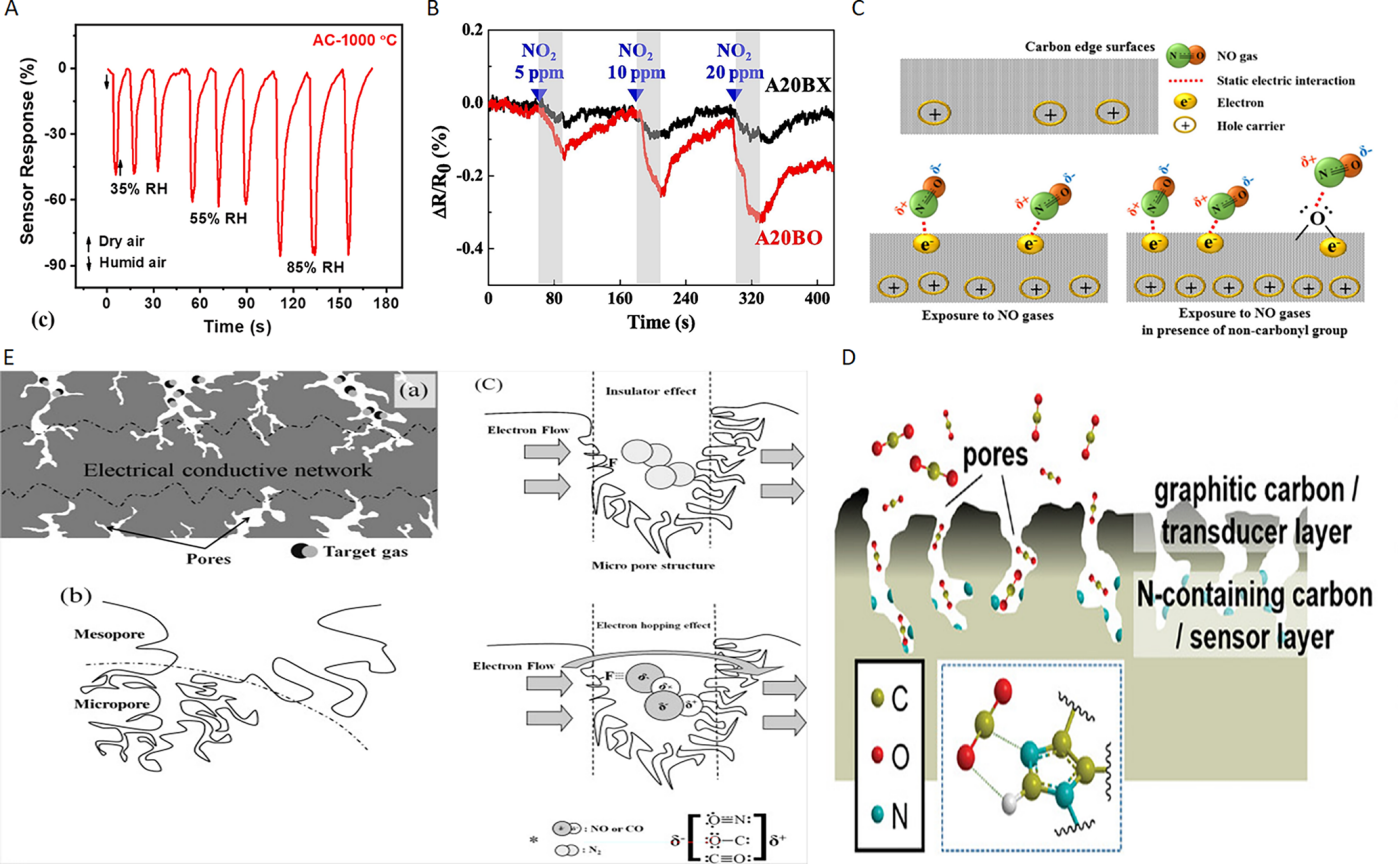
A) Transient sensor response curves of relative humidity (RH) ranges from 35 to 85%. Reproduced with permission from ref.^1108^ Copyright 2022, Elsevier; B) Response curves of undoped and boron doped activated carbon fibers with respect to various NO_2_ concentrations. Reproduced with permission from ref.^1110^ Copyright 2020, Elsevier; C) Schematic representation of the noncarbonyl group effect on carbon material surfaces. Reproduced with permission from ref.^1111^ Copyright 2016, the authors, under CC-BY-NC 3.0 license; D) Suggested mechanism of gas sensing: (a) suggested structural diagram of the prepared gas sensor in this study, (b) structure of micro- and mesopores, (c) induced fluorine effects for high sensitivity of gas sensor. Reproduced with permission from ref.^1112^ Copyright 2010, Elsevier; E) Schematic function of sensor. Reproduced from ref.^1113^ Copyright 2022, the authors under CC-BY 4.0 license.

##### 4.6.1.2 NO_*x*_ Sensors

Besides ammonia, nitrogen oxides, NO_2_ and NO, have been targeted in research addressing the applications of porous carbon as gas sensors; however, in some of them, a proposed sensing mechanism is based only on physical interactions of the gas molecules with the carbon surface.^1109^ Interesting results were presented by Kim et al.^1110^ who used activated carbon fibers doped with boron through a thermal treatment with boric acid at 1500 °C. The boron doping significantly improved the sensor performance (Figure 37B) and 20 s response time and 40 s recovery were recorded upon exposure to 10 ppm of NO_2_. This good performance was attributed to narrow micropores and strong interactions with boron-containing functional groups, mainly with both graphitic (BC3) and functionalized forms (BCO2 and BC2O), detected on the fiber surface by XPS. These results were supported by the DFT calculations of the binding energies of NO_2_ interactions with those surface entities. The sensing mechanism was explained based on an electron transfer process. Doping with boron made A20BO to behave as a p-type semiconductor causing the electron transfer from A20BO to the NO2 molecules, leaving holes in ACF. That increase in the hole density led to a decrease in the resistance of A20BO when upon NO_2_ adsorption on its surface.

A few studies indicated the importance of carbon surface groups for the NO and CO sensing. The carbon surfaces oxidized by various methods or reduced by thermal treatments have been tested. Park et al.^1111^ linked an increase in the sensor response upon E-beam irradiation to the changes in oxygen functional groups and noncarbonyl (−C–O–C−) groups. According to them, the exposure to E-beam under KOH solution converted O=C, HO–C, and HOOC-formed in the oxidation process to noncarbonyl groups, which more positively charged the carbon through interactions between the −C–O–C– groups and NO gas (Figure 37C). That increase in hole carriers reduced the resistance, and led to an increase in the NO gas sensitivity. In the further research from this group^1114,1115^ the positive effect of porous ACF treated under E-beam in oxidized atmosphere was confirmed.

On the other hand, Kwak et al.^1116^ found that surface oxygen groups of carbon activated with KOH increased the interaction time of NO with the carbon surface and therefore the response of the sensor was enhanced for the carbon with less oxygen incorporated to the surface. The carbon fibers chemically modified with KOH and fluorinated were investigated by Sun et al.^1112^ as sensors of NO and CO. The XPS results indicated that, semi-ionically bound fluorine, covalent CF and perfluorinated CF bonds were introduced to the surface. These groups increased the response of the sensors for both gases and the electrical resistance decreased whether it was p-type or n-type. The fluorine in the carbon matrix, due to is high electronegativity, caused that NO and CO were attracted strongly to the sensor due to the electronic structures of the NO and CO molecules (Figure 37D). Thus, the electron localizing interactions were suggested as causing electron hoping effects in pores, increasing the sensor performance. Similar trend upon the activation of polyacrylonitrile fibers/CNT sensors was found by Chang et al.;^1117^ however, the developed surface area upon activation was indicated as important.

##### 4.6.1.3 Sensors for Other Gases

Very limited number of studies focus on the application of carbon sensors for such gases as HCl or CO_2_. In the case of the former, the electrical resistance of the carbon increased upon the exposure to reducing HCl which provided electrons to the carbon surface, decreasing a hole density.^1118^ In the case of CO_2_ sensing^1113^ the carbon layer was prepared by a laser treatment of nitrogen containing organic compounds with an addition of a pore former. The carbon film was rich in imidazolic/pyrrolic nitrogen which increased the interactions of CO_2_ with the surface (Figure 37E). The carbon also had plenty of polar groups such as C–O and C=O, which were suggested as enhancing the sensor response; however, CO_2_ is not considered as a polar species.

#### 4.6.2 Porous Carbon-Based Electrodes for Electrochemical Sensing

Electrochemical sensors are a group of chemical sensors where the electrochemical interactions between an analyte and electrode are transformed into a useful signal. In many of such sensors, porous carbons are used as electrodes on which irreversible chemical reactions take place. For the response of such an electrode, often referred to as a carbon paste electrode (CPE), the rate of an electron transfer between the electrode and analyte is important. There, the carbon porosity combined with rich and specific surface chemistry might positively affect the charge transfer kinetics, resulting in high currents and low overpotentials. The carbon electrode development started from graphite with a gradual move, especially in the last 20 years, toward porous carbon materials synthesized by various methods, including ordered mesoporous carbons and carbons derived by carbonization/pyrolysis of MOFs. The functional groups of carbons play an important role in the generation of detection signals. Even though numerous applications of such sensors focus on the detection of important biomolecules,^1119–1121^ many of them are directed toward environmental pollutants and following the scope of this review, such sensors addressing the significance of porous carbon surface chemistry, are addressed below.

An important environmental problem is the detection of pesticides, even in small concentrations. One of those target substances is methyl parathion and a sensor for its detection was described by de Oliveira and co-workers.^1122^ The authors activated biochar with nitric acid and used it as CPE. For comparison, nonactivated biochar was also tested. The response of the sensor was measured by differential pulse voltammetry (DPV) (Figure 38A). The char was prepared from castor oil cake biomass by carbonization at 400 °C. The treatment with nitric acid, besides the removal of some inorganic matter also introduced oxygen and nitrogen to the carbon matrix through the oxidation process. This treatment also resulted in an 80% increase in the surface area and pore volume. As a result, the sensitivity of the activated biochar electrode, CPME-AB, increased compared to that of untreated biochar, CPME-PB (0.76 μA L μmol^–1^ for CPME-AB compared to 0.46 μA L μmol^–1^ for CPME-PB). A linear dynamic range was 0.1 to 70 μmol L^–1^ for the CPME-AB and 0.1 to 50 μmol L^–1^ for the CPME-PB. Even though the functional groups were evaluated only quantitatively by FTIR, the authors linked an increase in the response to methyl parathion to increased amounts of carboxyl, hydroxyl, lactonic and phenolic groups, that interacted with the analyte mainly through hydrogen bonding (Figure 38B).

**Figure 38:**
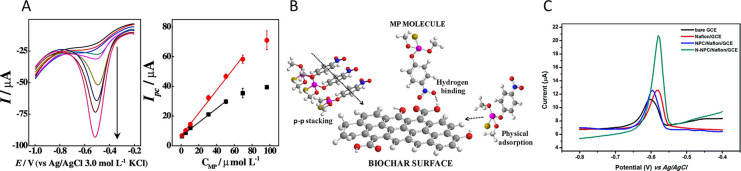
A) Analytical curves for different concentrations of MP using the modified electrodes with precursor (ack square marker) and activated biochar (red dot marker). Preconcentration conditions: acetate buffer solution pH 5.0 containing MP, preconcentration time of 5 min. Measurement conditions: acetate buffer solution pH 4.0, pulse amplitude of 100 mV s^–1^ and pulse time modulation of 100 ms. Adapted with permission from ref.^1122^ Copyright 2017, Elsevier; B) Possible adsorption mechanisms between biochar surface and methyl parathion. Reproduced with permission from ref.^1122^ Copyright 2017, Elsevier; C) DPASV curves at different electrodes in 0.1 MHAC–NaAc buffer (pH 5.0) containing 100 mg L^–1^ of Pb­(II). Reproduced from ref.^1129^ Copyright 2019, the authors under license CC BY 3.0.

Another example of using porous carbon/biochar as the CPE electrode is the detection of herbicide paraquat (1,1′-dimethyl-4,4′-bipyridinium chloride, PQ2+).^1123^ The performance was measured by Differential Pulse Adsorptive Stripping Voltammetry (DPAdSV). Biochars were prepared in the temperature range between 200 and 600 °C, without any activation processes and the best sensing performance in terms of the measured current was obtained with the sample pyrolyzed at 400 °C. Even though the porosity determined by adsorption of nitrogen was very small, FTIR results showed a decrease in the intensity of the FTIR band for chars heated at temperature >400 °C. Based on it, the authors hypothesized that paraquat interacted with carboxylic acid, hydroxyl and aromatic carbon groups of the biochar obtained at 400 °C, increasing the performance, which was better than that on the graphite CPE.

Other compounds which might exist in water as contaminants are phenols. The detection of catechol (CAT), 4-ethylcatechol (4-EC), and 4-ethylguaiacol (4-EG) by cyclic voltammetry was tested on various porous carbon materials by Kalinke et al.^1124^ on CPE modified with activated biochar, CPME-AB, addressed in details in ref 1122 where methyl parathion was addressed. Both cyclic voltammetry and electrochemical impedance spectroscopy (EIS) measurements were performed. In comparison with unmodified CPE based on graphite powder, the electrode modified with activated biochar (CPME-AB) showed a decrease in the peak current and an increase in the separation of anodic and cathodic potential peaks. This was attributed to the low electrical conductivity of CPME-AB modifier and to a negative surface due to carboxylic acids present at biochar. Since no additional surface chemistry analyses were performed to strengthen this argument, this conclusion was based only on FTIR qualitative evaluation addressed in ref 1122.

The detection of Catechol (CC), resorcinol (RC), and hydroquinone (HQ) by differential pulse voltammetry (DPV) was tested by Chen and co-workers.^1125^ Their pig lung-derived carbon had a high surface area, micro/mesoporous structure, and nitrogen incorporated to the matrix was in pyridinic, pyrrolic/pyridone and quaternary nitrogen structures. The modified CPE was able to selectively detect three target dihydroxybenzene isomers with a large linear range. LOD for HQ, CC, and RC were 0.317, 0.078, and 0.057 mmol L^–1^, respectively. Moreover, the response of the senor was stable (5% change in the current) in the presence of phenol, o-nitrophenol, m-nitrophenol, p-nitrophenol, Na^+^, Cl^–^, Zn^2+^, SO_4_^2–^, Ca^2+^, Cu^2+^, K^+^, Mg^2+^, NO_3_^–^ in 10-fold concentration of that of Catechol (CC), resorcinol (RC), and hydroquinone (HQ). The authors hypothesized that nitrogen in the carbon matrix enhanced the electron-donor ability, improved the conductivity and caused a net positive charge on the carbon atoms. It was indicated that all of these features could bring unique redox properties affecting the electrochemical oxidation kinetics, due to an electron cloud distribution and polarity imposed by different electronegativity of nitrogen and carbon atoms.

Derylo-Marczewska et al.^1126^ evaluated the sensing ability of commercial Organosorb −10 activated carbon to detect 4-chlorophenol. Prior to sensing, carbon was deashed and oxidized by exposure to O_3_ at 25 °C for two different times. The samples’ chemistry was evaluated by a thermal analysis, NaOH uptake and XPS. The results clearly showed that longer exposure time to ozone introduced more acidic groups, mainly in the form of carbonyls/carboxylic acids and ethers/OH groups. The measured peak current representing phenol oxidation strongly depended on the carbon oxidation level (more oxidized-smaller current) and it was concluded that the sensing performance was governed by 4-chlorophenol adsorption ability of carbons, which decreased with an increase in the oxidation level. However, oxidation might have also decreased the conductivity affecting negatively the current. Nevertheless, the best sensitivity was found for the unoxidized carbon with the limit of detection (LOD) of 2.38 μmol L^–1^, which was over 30 times lower than that for the bare electrode made of graphite.

Emerging contaminants, such as pharmaceuticals and their metabolites have been also targeted in electrochemical detection and sensing using CPE containing porous carbons. Ramadhass et al.^1127^ used 3D-graphen-like porous activated carbon nanosheet (3D-PAC) obtained from the Borassus flabellifer biomass to detect furazolidone. The nitrogen containing char was activated by KOH at 1000 °C. The surface chemistry of porous carbon was analyzed by XPS and C=O, N–C=O, C–N, and C=C pyridinic, pyrrolic, graphitic nitrogen, along with oxygen in aromatic C=O, aliphatic C–O, and aromatic C–OH configurations were detected based on the deconvolution of C 1*s*, N 1*s*, and O 1*s* core energy level spectra. The content of oxygen and nitrogen was 9.77 and 2.77 at. %, respectively. The sensing of the porous carbon-containing electrode, 3D-PAC/GCE was evaluated in detail and compared to that of GCE. The modified electrode showed the high capacitive current (due to porosity) and a sharp reduction peak for furazolidone at the reduction potential of −0.38 V with the cathodic peak current of −76 μA. On the other hand, the bare GCE reduced the drug at the potential of −0.42 V, and the current was −11 μA. The observed reduction peak was linked to the nitro group conversion into hydroxylamine. Even though the nitrogen content on the carbon surface was indicated as significantly enhancing the performance, no further information on the role of specific surface groups was provided, in spite of the detailed XPS analysis presented by the authors. They suggested that a unique interconnected 3D-porous structure and a large number of active sites, along with a high surface area, decreased the diffusion path and thus improved the electrocatalytic activity. The sensor containing porous carbon had a detection limit of 0.5 nM and the marked sensitivity of 5.05 μA μM^–1^ cm^–2^.

The detection of the mixture of three toxic environmental compounds, namely quinone, hydroquinone and NO_2_^–^ ion was tested on popcorn-derived carbon obtained by KOH activation.^1128^ It was indicated that the selection of the precursor ensured self-N-doping of the carbon matrix. The carbons were activated at 700, 800, and 900 °C and have the surface area between 940 and 1200 m^2^ g^–1^, with the latter carbon having the smallest pores. FTIR spectra showed C–O and C=O bonds and nitrogen in N–H and N–O bonds. It was indicated that XPS spectra, however not included in the paper, suggested the presence of oxygen in C–O and C=O bonds and nitrogen in pyridinic N, pyrrolic N, and graphitic N. Elements’ atomic contents were not provided. The sensing experiment in terms of *I*–*V* curves showed that the electrode containing carbon obtained at 900 °C was able to simultaneously determine hydroquinone, quinone, and nitrite ion in the concentration range of 3.0–700 μM, 3.0–500 μM and 5.0–10000 μM with detection limits of 1.45 μM, 0.49 μM, and 1.5 μM, respectively. The good sensing performance was linked to a high density of edge-plane-like defect sites, and a uniform nanopore structure with a large-scale distribution. The authors indicated that the edge-plane carbon with a higher density of defects improved the electron transfer kinetics of quinone. They suggested that in the nanopores the residence time of the analytes increased due to confinement and this process enhanced the adsorption of the analytes, which in turn increased the electron transfer. Unfortunately, the article did not present any results on the density of defects or their nature and the surface chemistry results were not analyzed deeply to account for existing selectivity of the response.

The concentration of metals in water can be also tested using porous carbons. Their significant advantage is the ability to detect those species at very low concentrations. Baikeli et al.^1129^ prepared porous carbons from almond shells using KOH activation (NPC) and then modified their surface by thermal treatment with urea [N-NPC]. Differential pulse anodic stripping voltammetry (DPASV) was used as a testing method. The carbons exhibited high surface areas and micromesoporous structures. Both contained about 9% of oxygen and in N-NPC 2.3% of nitrogen was detected (specific units were not indicated). While nitrogen was in pyridinic N, pyrrolic N, and “oxidative species” (as assigned by the authors), oxygen was in C=O and C–O, with majority of the former species. Since the article does not specify on which carbon that XPS analysis was carried out, one could assume that N-NPC surface was targeted and that in N-NPS pyridinic groups were in vast majority. No information about the specific composition of the NPS surface was provided. The treatment with urea markedly improved the sensor performance (Figure 38C). Its response was not affected by interfering ions (Ca^2+^, Mg^2+^, Al^3+^, Fe^2+^, Fe^3+^, Zn^2+^, Co^2+^, Cr^3+^, Mn^2+^) of concentrations higher than that of Pb^2+^, which in fact might have been related to their different reduction potentials. Only in the case of Cu^2+^ interference was detected. In the pH range from 4 to 6, the highest current was measured at pH 5 and then a decrease was recorded. Unfortunately, the authors did not discuss the differences in the chemistry of both carbon materials. The good performance of N-BPB was linked to the presence of nitrogen that provided an increase in conductivity (although the conductivity was not measured) without indicating any specific role of surface groups. The dependence on pH might suggest that dissociating surface groups, such as carboxylic acids, are also important sites of metal cation binding.

Porous carbons obtained from dead mango leaves (SNAC) were tested as sensors of Cd­(II), Pb­(II), Cu­(II), and Hg­(II) by Madhu et al.^1130^ Their limits of detection for Cd­(II), Pb­(II), Cu­(II), and Hg­(II) ions at the SNAC-modified GCE were 24.4 nM, 5.7 nM, 23.2 nM, and 24.6 nM, respectively, and the sensitivities for all tested metals were less than 0.2 mA μM^–1^ cm^–2^. Carbons had a high surface area in the range of 1500 m^2^ g^–1^ and the elemental analysis indicated the content of C, H, N, and S as about 72%, 6%, 6.6%, and 7.5%. Even though the content of ash was not mentioned, the carbons were washed with HCl and the high surface area suggests the small content of mineral matter and thus the content of oxygen might have been about 8%. Based on the elemental analysis, and without discussion of the oxygen content or its speciation on the surface, the authors suggested that the adsorption of metal ions on SNAC occurred through the formation of carbon–oxygen complexes, as in the following reactions, proposed by Madhu et al.^1130^ with +1 charge on metals: 2COH + M^+^ → (CO)_2_M^+^ + 2H^+^ and COH_2_ + 2M^+^ → COM_2_^+^ + 2H^+^), and indicated that these interactions were supported by a decrease in the pH of the final solution. The ion exchange capacity of the specific ions was suggested as important for sensing; however, this quantity was not measured. Moreover, the authors did not consider the possibility of the contribution of the marked content of N and S groups to the sensing performance. A high surface area and specific porosity were indicated as important and in fact it might have contributed to the favorable dispersion of surface groups/adsorption active centers.

### 4.7 Microwave Absorption

Even though carbon-based materials are ones of the first microwave absorbents whose exploration started from the middle of the last century,^1131^ the recent developments of nanomaterials and demands for Stealth technology directed the attention of scientists to porous carbon structures. One of the general objectives behind the development of microwave absorbing materials (MAMs) is to avoid or to delay a detection of airborne objects during tactical operations. Since most wireless communications operate at high frequency (500 MHz to 5 GHz), another objective leading to the recent boom in the MAMs’ development is the elimination of interferences which might negatively affect electronic devices and also human health.

Incident microwave consists of an oscillatory electric field and magnetic field. MAMs are expected to interact with either one or with both. Both dielectric loss and magnetic loss are important for dissipation of electromagnetic (EM) waves in microwave absorbing materials.^1132–1134^ Good absorbents are expected to possess a large reflection loss (*R*_L_) and a wide effective absorption band. Not without importance is their lightweight. Carbons, especially porous ones, have a low density (linked to their high porosity), are chemically and mechanically stable, not expensive, and easy processable. They exhibit a strong dielectric loss, and a high frequency coverage (8–18 GHz). The MW energy dissipates in carbons by a conduction loss mechanism and interfacial polarizations. By changing the forms of carbons (shapes, density, allotropic form), a reflection loss (*R*_L_) and absorption frequency bandwidth can be adjusted. Even though graphene and CNT are good MAMs mainly due to their high electrical conductivities, their high costs limit large scale applications, leaving this emerging field for their porous allotropes.

An important asset of porous carbons in MW absorption is their “composite” nature, which consists of a carbon matrix and air trapped in a pore system. Such composition provides a favorable/low density and increases the impedance matching.^1135^ Moreover, the porous structure contributes to a more interface polarization loss leading to an increased absorption of the electromagnetic wave energy.^1134^ Another important factor is a broad variety of porous carbon surface properties, including their pore volume, sizes of pores, their distribution and organization, surface chemistry, graphitization level and electronic properties/conductivity of their frameworks. The latter is strongly influenced by heteroatom doping. Nevertheless, with all these positive factors, porous carbons suffer from impedance mismatching, which causes a microwave reflection from the carbon surface, and provides the limited mechanism of a dielectric loss.^1135^

The impedance mismatching problem in porous carbons is addressed by alterations of their pore structure using various activation methods or complex synthesis protocols.^308,1136–1138^ An increase in the pore volume, and thus space occupied by air enhances EM wave penetration and thus increases the impedance matching with free space. Pore space also increases the contact probability of EM wave with the absorber. A developed porosity is also associated with more carbon surface-air interface for the accumulation of charge and thus it leads to an increase in the interface polarization. In fact, the majority of efforts that have been directed to increasing the performance of porous carbon as the EM wave absorbents focus on understanding the effects of the pore morphology.^1137,1138^ Regarding the pore sizes/shapes, it was found that uniform in size pores as for instance cage-like pores,^1139^ especially in a mesopore range lead to longer propagation tunnels for the dissipation of energy. They also provide multiple reflection and scattering sites.^1136^

To provide more EM wave dissipation mechanisms and to improve the impedance matching, semiconductors, metal oxides, and magnetic materials are used as additive microwave absorbing components. The analysis of this kind of surface modification is beyond the scope of this review and the reader is referred to detailed information included in ref. 1131,1140 With a focus on carbon matrix chemistry, we provide examples of its effects on the dissipation of the MW energy. The specific examples of heteroatom doping and defects/graphitization levels are discussed below. Even though the results on the direct effects of that chemistry are rather limited, such information is withdrawn from the published results to emphasize the importance of the “isolation” of this feature and to prompt the further investigations of surface chemistry effects in this emerging carbon application field.

The importance of defects in general in the carbon structure for microwave absorption was indicated by Hou et al.^1141^ Their carbon was obtained from potassium citrate in a sealed tube in an argon atmosphere. It had a developed porosity and a high defect level. The latter was determined by Raman spectra (*I*_D_/*I*_G_ and a deconvolution approach). Even though the amount of surface oxygen was not provided, an XPS survey suggested its rather high content and the deconvolution of O 1*s* core energy level spectra indicated the presence of quinones, phenols, ether and carboxyl groups; however, the specific contribution of each oxygen group was not included in the paper. Their presence might be a consequence of the decomposition of carboxylic acids in an inert atmosphere in the sealed tube. The authors indicated the intrinsic defects formed due to pressure generated during carbonization (Figure 39A). It contributed to the well-matched impedance and strong polarization loss beneficial for EM wave dissipation. The reflection loss was considered as strong and reached −58.2 dB with an ultralow filler loading of 5% (Figure 39B). Even though surface chemistry was evaluated in detail, the effects of the specific groups/defects on the polarization loss were not discussed. Internal defects were also indicated as important for EM wave absorption by Zhu et al.^1142^ They activated hollow carbon spheres by KOH. The process led to the formation of defects/dangling bonds, distinguished by their geometrical arrangements. Those unsaturated bonds were indicated as susceptible to the EM field, inducing electromagnetic resonance signals. More dangling bonds are associated with more defects in carbons and thus more sites for interactions with EM waves. The authors also suggested that the EM field might have induced the rotation and vibration of dangling bonds leading to their displacements and conversion of the EM energy into mechanical and heat energy. Overall, those entities were indicated as increasing the dielectric loss due to their contribution to the interfacial polarization and space charge polarization. Even though atoms attached to the dangling bonds were mentioned, their chemical nature was not investigated.

**Figure 39:**
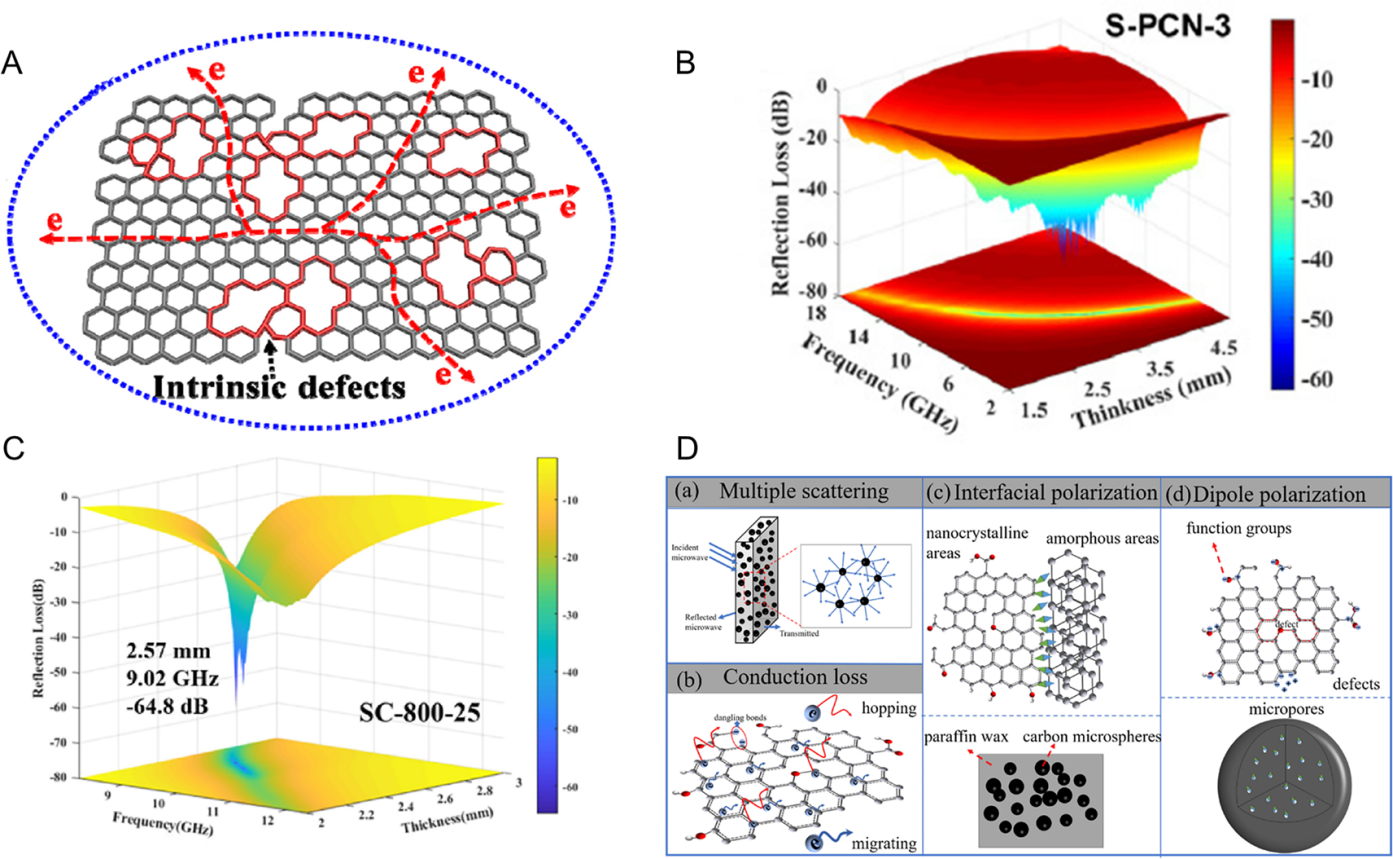
A) Defects in the porous carbon nanosheets. Adapted with permission from ref.^1141^ Copyright 2021, Elsevier; B) Reflection loss curves of SPCN-3 Reproduced with permission from ref,^1141^ Elsevier; C) The 3D reflection loss plots of SC-800-25. Reproduced with permission from ref.^1143^ Copyright 2021, Elsevier; D) Scheme of microwave attenuation models of SC/paraffin composites. Reproduced with permission from ref.^1143^ Copyright 2021, Elsevier.

The role of defects and oxygen-containing functional groups in absorbing the microwave energy was analyzed to a greater extent by Nan and co-workers.^1143^ The carbon spheres were obtained from starch using atomization followed by carbonization at a temperature range between 750 and 1000 °C. Based on HRTEM, carbon spheres were considered as microporous; however, a direct porosity analysis was not carried out. The combined analyses of HRTEM, XRD, and Raman spectra suggested that the intrinsic defects of graphitic edges and micropores (quantity not specified) were at maximum upon heating at 800 °C. The XPS analysis revealed the presence of nonspecified amounts of oxygen. The deconvolution of O 1*s* core energy level spectra indicated the presence of quinones, ether, carbonyl and carboxylic groups on the surface of all carbons and an increase in the heating temperature caused the conversion of C–O–C/C=O to C=C/C–C. The oxygen was still detected upon treatment at 1000 °C. The best performing sample as a microwave adsorbent was the one carbonized at 800 °C (Figure 39C) with a high content of defects and oxygen. The complex mechanism proposed by the authors is displayed in Figure 39D. While absorbent particles caused multiscattering, the conduction loss was a result of an electron migration and hoping among the nanocrystalline areas and the electrical charge accumulated on a heterogeneous interface due to the difference in the electronegativity of the components of the carbon surface. Surface defects, functional groups and micropores acted as polarization centers. Upon applying an electric field, dipoles were generated, imposing a dipole polarization loss to attenuate the wave. Regarding the oxygen-containing functional groups, carbonyls were predominant on the surface of these carbons. This group has a planar geometry and its carbon atom is electrophilic (electron-deficient), and oxygen is nucleophilic (electron-rich) owing to its higher electronegativity. It would be interesting to evaluate the effects of their high dipole moments (∼2.6–28 D), compared to other detected groups (carboxylic: 1.7–2.7 D; ether: ∼1.3 D; quinones: 0.67 D), on the polarization loss.

The specificity of oxygen groups and their effects on microwave absorption is often neglected during the analysis of the absorption performance. The efforts mainly focus on the morphology or on the development of porosity. Especially in biomass derived carbons for MW absorbing, KOH is often the activation agent of choice.^1144^ Its application, besides the development of pores, leads to the formation of defects and surface oxygen functional groups. Such carbons, when not acid washed, might also contain marked amounts of mineral components. These factors might also affect the MW attenuations, and have not been discussed yet.

The effect of nitrogen groups incorporated to the porous carbon surface on attenuating the MW energy has been analyzed by Wang et al.^1145^ The mesoporous carbon was obtained by a complex soft templating method from GO and polypyrrole. Carbonization was carried out at 500, 700, and 900 °C. The best performing carbon was carbonized at the highest temperature and exhibited *R*_L_ of −66.1 dB with an effective bandwidth of 8.2 GHz (Figure 40A). An increase in the heating temperature caused an increase in the surface area (253 m^2^ g^–1^), which was linked to the generation of micropores. *I*_D_/*I*_G_ also increased. The XPS analysis detected N and O as heteroatoms, but only the former was further analyzed by the deconvolution of its core energy level spectra. Pyrrolic, pyridinic and graphitic nitrogen were detected on the surface. With an increase in the temperature, pyrrolic nitrogen was converted into pyridinic and graphitic with the slight predominance of the latter in the sample carbonized at 900 °C. The surface spectra suggested that the contents of both N and O decreased with an increase in the heat treatment temperature. The good performance was linked to an excellent impedance matching due to the mesoporous structure and numerous carbon-air interfaces. The stress was also placed on the role of N-doping on generating the dipolar polarization, which contributed to the dielectric loss. The presence of graphene led to a conductive network, which facilitated the hopping and migration of electrons. Since the nitrogen doping combined with the presence of graphene resulted in a large charge density distribution at the interface of air and carbon, under the electromagnetic field, the electric field was formed generating a marked interfacial polarization leading to the dielectric loss. The proposed mechanism with the dipole and defect polarization is presented in Figure 40B. As in the case of oxygen species reported by Nan and co-workers,^1143^ the authors did not analyze the effects of the differences in the dipole moments of nitrogen species. The pyridine groups have a high dipole moment (2.26 D) and their population in the best performing sample was very similar to that of quaternary nitrogen. Similar conclusion on the dipole polarization effect due to N-doping was presented by Dai et al.^1146^ However, their N-doped carbon fibers were not porous. There the authors pointed out the effect of graphitic nitrogen on enhancing the conductive loss. Tang et al.^1147^ stressed the coupling effects of polarization and a resistance loss in nitrogen containing carbon of a specific micronano hierarchical structure on strongly enhancing the MW dissipation (Figure 40C). Their carbons were obtained from polypyrrole and exhibited *R*_L_ of −75.10 dB with the effective bandwidth of 6.08 and 4.05 GHz. They also had the high population of defects (*I*_D_/*I*_G_ close to 1.3). The content of nitrogen and oxygen evaluated by XPS was 9.3 and 4.2 at. %, respectively, with traces of iron (0.37 at. %). Oxygen was in C=O and C–O groups. Pyrroles and pyridine nitrogen species were present on the surface. The authors indicated that both nitrogen species worked as polarization centers owing to their attachment to two atoms, leading to the formation of vacancy defects.

**Figure 40:**
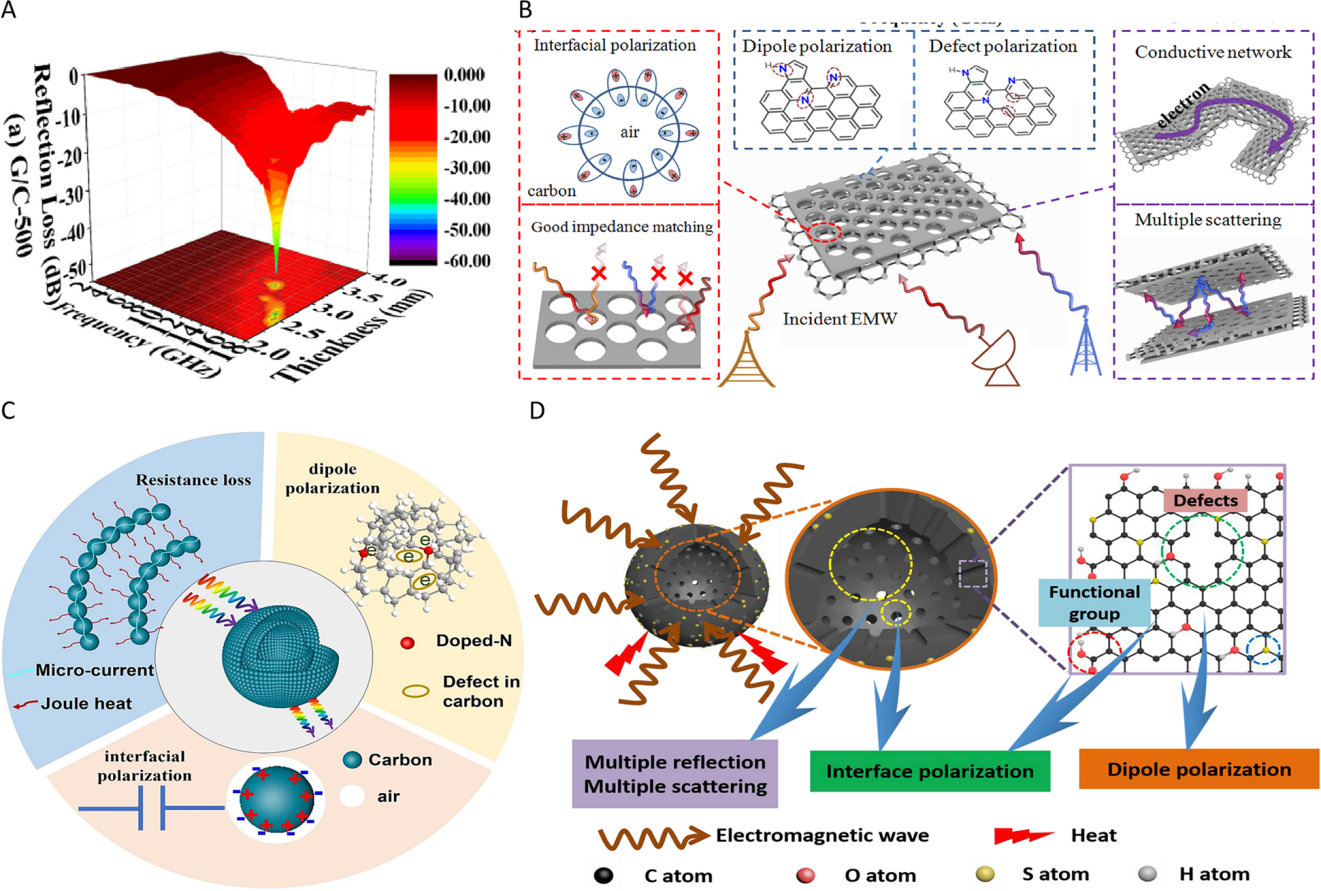
A) 3D projection plots of G/MC-900 3D RL Adapted with permission from ref.^1145^ Copyright 2021, Elsevier. B) Schematic diagram of microwave absorption mechanism of G/MC-900 composite. Reproduced with permission from ref.^1145^ Copyright 2021, Elsevier; C) Schematic illustration of enhanced microwave dissipation mechanisms in double-shelled N-doped carbon hollow microspheres. Reproduced with permission from ref.^1147^ Copyright 2024, Elsevier; D) Microwave absorption mechanism of S-doped PCHMs. Reproduced with permission from ref.^1149^ Copyright 2020, Elsevier.

Sulfur-doped porous carbons have been investigated as microwave absorbing materials by Zhang et al.^1145^ Their carbon spheres with mesoporous shell were hydrothermally treated with various amounts of thiourea at 180 °C. Upon the treatment, S and O were detected on the surface of carbons and, interestingly, nitrogen existing in thiourea was not reported as incorporated to the carbon matrix. Sulfur was indicated as in C–SO_*x*_–C and C–S–C bonds. Oxygen, based on O 1*s* spectra, was in C–O, C=O, and −OH bonds. The survey spectra and EDS elemental maps indicated its rather high amounts. The contributions of C=O and −OH bonds were predominant and almost equals. The S 2p spectra were rather noisy and not deconvoluted. The binding energy values suggested a very high contribution of oxidized sulfur. The heteroatoms were evenly dispersed on the surface of the spheres. The best results with minimum *R*_L_ of −51.83 dB at 15 GHz were obtained for the sample with 10 times more thiourea used to treat the carbon spheres. Based on the intensity of S 1*s* core energy level spectra, one could conclude that this sample had the highest contribution of oxidized sulfur, although small compared to the reduced sulfur, among all tested samples. The authors suggested that functional groups containing sulfur acted as dipole centers, inducing dipole polarization and converting the electromagnetic energy into heat, causing the relaxation loss. The authors did not discuss the sulfur or oxygen content. The latter is certainly linked to the presence of oxidized sulfur. Even though the authors referred to them as C–SO_*x*_–C, they might be in the form of sulfones, which have a very high dipole moment (4–5 D). This, combined with the high dipole moments of carbonyls, might contribute to the marked dipole polarization effects. The carbon matrix with defects and O and S incorporated to the carbon matrix presents a very peculiar arrangement of atoms as proposed by the authors in Figure 40D, visualizing the mechanisms of MW attenuation on their S-doped carbons.

There are also quite frequent reports addressing the positive aspect of dipole polarization originating from the carbon support of complex chemistry in microwave energy dissipations in carbon/inorganic phase composites.^1135^ Many of them address rather non porous carbon materials such a graphene. Even though addressing these composites and nonporous carbons allotropes are beyond the scope of this review, we briefly mention the effect of other heteroatom than solely, O, N, or S, with justification that the particular surface chemistry can be also implemented to the porous carbons, providing an inspiration for an increase in the performance of the porous carbon microwave absorbents. In the majority of cases, the exact content of heteroatoms or specific groups is not analyzed quantitatively. Nevertheless, their effects on the dipole polarization are mentioned. Also, the addition of an inorganic phase provides more diverse mechanisms of the microwave energy dissipation. One example is the carbon composites with Ni_3_S_2_ obtained from sucrose and thiourea.^1148^ In this case, besides sulfur, nitrogen originating from thiourea was incorporated to carbon and surface heterogeneity was indicated as contributing to the dipole polarization effect. That effect decreased with the heating temperature as the content of heteroatoms decreased. The content of the elements was not provided. C–N, C–S, C–O, and C=O were detected from the deconvolutions of the core energy level spectra. In this case, the content of nitrogen seems to be relatively high (comparable to that of oxygen based on the survey spectra) and pyridines were the predominant groups, besides pyrrolic and graphitic nitrogen. The sulfur 2p spectrum suggested the presence of thiophenes in the carbon matrix.

The beneficial effect of the incorporation of boron and nitrogen to the carbon matrix was addressed by Kang et al.;^1150^ however, in their work nonporous GO was modified with urea and ammonia at 900 °C. In the ammonia treated sample, the amount of N, B and O was 32.64, 28.31, and 7.78 at. % respectively, suggesting rather a separate phase of h-BN boron nitride and the presence of BCN. While nitrogen was in N–B, B–N–C (predominant) and in graphitic form, boron was in B–C, B–N (predominant), and B=O. Boron carbide and carboxylic acid were also present on the surface of the materials. The authors suggested that the boron and nitrogen incorporation adjusted the location of the reflection peaks and was beneficial for a reflectivity. The dipole effects often associated with the presence of heteroatoms were not discussed.

A recent exponential increase in the number of research papers addressing microwave absorption stresses the significance of this emerging application of porous carbon materials. Even though the morphology effects on the MW energy dissipation have gained marked interests of researchers, a detailed investigation of the effects of surface chemistry and defects still remains in its infancy. Since the microwave properties and wave attenuation are often investigated by physicists and a broad range of engineers (Stealth technology, electrical, aeronautics, etc.), this field might benefit from closer collaboration with materials scientists/carbon researchers, being able to isolate and to better understand the effects of the diverse populations of carbon surface features on microwave energy attenuations.

## 5 Concluding Remarks

The objective of our work was to provide, in a critical way, a comprehensive view of the importance of porous carbon surface chemistry determining their applications in a broad range of the energy–environment nexus. Following this, we have addressed, with a critical analysis, the importance of the carbon surface chemical environment to separation, catalysis, energy storage, sensing and microwave absorption. That application range is very broad and involves scientists and engineers of various disciplines. In our opinion, the extent of this review testifies on the importance of the issues we would like to share. We realize that for some readers, due to the broad range of chemical and physical principles used in the applications covered, it might be challenging to go through a whole review with all its details. Nevertheless, those interested in a specific problem within a scientific discipline can still focus on their topic of relevance addressed in a specific section of this review.

The performance of porous carbons in many fields is determined by a complex combination of features, including a wide range of structures and nanostructures at long- or short-range distances, defects, the presence of heteroatoms and porosity. The combination of all these parameters contributes to the understanding of the role of different parameters, which is essential to achieve the best performance. Unfortunately, the convolution of the contribution from each factor can result in quite different performance for apparently similar materials, which makes it difficult to identify the main contributor. Reporting disaggregated data on porosity and composition, along with a complete characterization of the surface groups (beyond heteroatom contents) is the key to unravelling properties–structure–performance correlations. The application of *operando* techniques is also essential for understanding reaction mechanisms through real-time experiments.

Recent advances have demonstrated the major role of computational modeling and machine learning approaches in the understanding of the role of surface chemistry of carbon materials in various application fields. Future directions should focus on developing more accurate models to simulate gas–liquid–solid interactions and incorporate effects as irradiation and electric fields to bridge experimental and computational advances and improve our understanding of properties–performance relationships.

In the field of energy storage, a deep knowledge of carbon materials’ interactions with electrolytes is essential for optimizing their performance. In this sense, surface chemistry is the most determining property for the reactivity with the electrolyte. Understanding the fundamentals of the reactivity between carbon materials and the electrolyte is essential for designing strategies to improve the overall performance of electrochemical capacitors and metal-ion batteries. For example, oxygen functional groups play a double (and opposite) role. While they contribute to pseudocapacitance, oxygen functional groups can also accelerate electrode degradation. Then, strategies should focus on removing or blocking these sites to enhance stability and expand the voltage window, in the case of capacitors, or to reduce irreversible capacity and improve stability in metal-ion batteries. Surface chemistry tailoring methodologies like hydrogen reduction, heteroatom doping (e.g., nitrogen, phosphorus), and structural modifications to reduce edge sites are promising approaches for improving carbon materials. Consequently, designing advanced electrodes like monolithic structures with continuous architectures that eliminate grain boundaries with minimal edge sites and tailored surface chemistry could significantly boost electrochemical stability and performance. These carbon materials can be connected to battery-like electrodes building asymmetric configurations that may offer new possibilities for hybrid devices that can be competitors with conventional batteries. Na-ion batteries technology is in its initial stages of development and there is room for optimization through designing optimal porous carbon. In this field, hard carbons and other low-crystallinity porous materials are promising for sodium-ion batteries, though cost and reproducibility remain challenges. Consequently, there is a need for scalable, cost-effective synthesis methods that reduce costs and ensure material consistency since this is critical for large-scale application of carbon materials.

We anticipate that a truly reliable understanding of metal-free carbon catalysis can only emerge from a holistic approach incorporating a rigorous experimental design, comprehensive characterization and advanced molecular modeling. Firstly, the experimental design must be conceived to ensure that carbons differ exclusively in the surface functional groups under study. All other variables, such as porosity, graphitic order, and residual heteroatoms, must remain constant. This can be achieved through stepwise thermal annealing, which removes functional groups according to their stability. This methodology has already been validated for O-, N-, P-, S-, and B-doped carbons. Alternatively, chemoselective “poisoning” can be used with probe molecules that react with just one type of functional group. Second, any catalyst series should be fully characterized before, after, and, if possible, during the reaction. Quantitative analyses, e.g. XPS, FTIR, solid-state NMR, TEM, or XAS (ideally applied in operando), allow one to follow the changes of each surface group in real time and to correlate structural evolution with activity or selectivity. Thirdly, molecular modeling needs to move beyond ideal graphene sheets towards realistic carbon fragments that incorporate dopants, edge sites and vacancies, while also accounting for the actual reaction environment. Such simulations can then be matched directly to an *operando* data. Only by merging insights from these three approaches can we identify true active sites, extract reliable turnover frequencies, and design carbon catalysts optimized for industrial reactions.

The dual nature of porous carbons with well-defined pore architectures and composition is essential in photocatalytic applications. On one side, adsorption in the pores is important and matching the porosity of the carbon to the molecular dimensions of the target molecules is critical to ensure the affinity of the molecules to the carbon surface. On the other side, surface functionalization can affect the strength of the adsorption, but most importantly can modify the light absorption properties of the photocatalyst by incorporating chromophoric surface moieties capable of absorbing light at long wavelengths (boosting solar driven applications) and of photogenerated additional charge carriers to increase conversion. As in classic catalytic applications, those properties can control the selectivity of a photocatalytic reaction, which is important for instance for the reduction of CO_2_. The difficulty stands from understating the effect of both aspects and disaggregating the contribution of the porosity and surface chemistry. While this may be considered as a utopic final goal, more attention should be paid to better characterize the carbon photocatalysts to understand the fundamentals of the light/carbon interactions at different levels, and reporting disaggregated data on porosity and composition. We anticipate that these advanced approaches would show that some popular functionalization strategies may (or not) be as useful as they would seem to enhance photocatalytic conversions.

Even though the application of porous carbons as adsorbents and separation media is the one with the oldest history and record of achievements, and therefore it could be considered as the most developed branch of a carbon-environment nexus, there is still room for its improvement and a need for comprehending mechanisms. Nowadays the field has to focus on the separation of emerging contaminants and xenobiotic compounds harmful to a biosphere in general, at very low concentrations. Their removal under natural environmental conditions makes the specific interactions of these molecules with a carbon surface critical and here the incorporation of heteroatoms in specific chemical configurations and location in a carbon pore system might contribute to the well-being of humanity.

Promising strategies for integrating defect engineering with device-level design in porous carbons, include tailoring defect types for specific functionalities, creating hierarchical porous structures, and engineering of the interface with biomolecules to achieve efficient interactions. These can also be extended to metals incorporation to enhance performance in energy and environmental applications. Defects and functionalities can be engineered to modulate electronic conductivity, stability, catalytic activity, and adsorption properties. While synthetic methodologies need to be developed to selectively create specific functionalities or defects, the urgent need is to unambiguously identify the structure and composition of those features. Combining micro- and mesopores in a hierarchical structure allows for an optimized ion transport and active site accessibility and it is a well-known requirement in many applications, and many publications include this aspect. However, the determination of a real hierarchical structure is still an issue that cannot be established from conventional gas adsorption techniques. Combination of defect-rich carbon materials with other porous materials like MOFs or 2D materials like graphene-based materials or MXene, is another approach to achieve enhanced electrical, mechanical and chemical properties. The synthetic approach must consider the integration of the different phases at the nanometer scale and using methodologies that enable efficient interactions without negatively impacting the intrinsic properties of the pristine materials. In this sense, electrochemical methods offer a promising way to precisely control the reaction conditions.

Porous carbons can be of great interest for anchoring of biomolecules, such as enzymes, DNA, peptides, and others, that can be used in biocatalysis, biosensors or drug delivery. The most important is to tailor not only the porosity but, especially, the surface chemistry to achieve a correct interaction. The latter specifically refers to interactions which are strong and provide the adequate orientation of the biomolecules. In this sense, computational studies are of great relevance to define the specific functionality or defect that can produce the best results. Another important issue is to create defects/functionalities that mimic the active sites of enzymes, which would allow the synthesis of biocatalysts-like materials with an enhanced selectivity and activity that would prevent the inevitable denaturation of enzymes.

Defects or functionalities in porous carbons are crucial in stabilizing isolated metal atoms to create highly active and selective catalytic sites for different reactions. However, this is not only true for single atoms but also for small metal clusters, which may contain more active and selective sites for the reactions than the single atoms. This issue tends to be overlooked. Thus, the presence of defects/functionalities is mandatory to get sufficiently strong interactions, with an adequate change in an electronic distribution that favors the formation of specific active sites with a correct atom ensemble. Designing adequate heterojunctions through interface engineering between carbon and metal species to optimize electronic band structure and facilitate charge separation is of relevance in hotocatalysis or electrocatalysis. In this context, computational studies are the first approach to establishing the correct combination of carbon/metal structure/composition prior to the synthetic efforts.

Last but not least to address are the emerging applications of porous carbons such as sensing or microwave absorption. They, and especially the latter, go beyond the concept of molecule–surface interactions and deeply involve solid state physics electromagnetism, and quantum mechanics, bringing more abstract and more convoluted concepts of the carbons-surface involvement. Here, a closer collaboration of chemists and physicists, mechanical and aerospace engineers could help to further advance this new field of strategic carbons applications, especially that recent reports suggest that surface chemistry might play a significant role in electromagnetic wave attenuation.

Generally speaking, out-of-the-box thinking and different perspectives are welcome means to improve and advance our understanding of these old but still attractive and exciting porous carbon materials, we address in this review.

## Supplementary Material



## Notes

Table S1 – Advantages and limitations of the most used methods to characterize the surface chemistry of porous carbons (PDF)
