# Supplementary material for: Advancing Porous Carbons: Understanding the Importance of Surface Chemistry for the Energy–Environment Nexus
Source: Chem Rev. 2026 Feb 5;126(4):2678–800. doi: 10.1021/acs.chemrev.5c00719 (PMC12947625; doi:10.1021/acs.chemrev.5c00719)
Supplement: Supplementary file 1 [file cr5c00719_si_001.pdf]

Advancing Porous Carbons: Understanding the Importance of Surface Chemistry for the Energy-Environment Nexus

Conchi Ania[1], Teresa J. Bandosz[2, 3]*, Diego Cazorla-Amorós[4], M. Fernando R. Pereira[5]

[1] CNRS (CEMHTI, UPR 3079), Université d'Orléans, 45071 Orléans Cedex 2, France
[2] Department of Chemistry and Biochemistry, The City College of the City University of New York, New York, NY 10031, USA
[3] Faculty of Chemistry, Maria Curie Sklodowska University, 20-031 Lublin, Poland
[4] Department of Inorganic Chemistry and Materials Institute, University of Alicante, Ap. 99, Alicante, E-03080, Spain
[5] LSRE-LCM, ALiCE, Faculty of Engineering, University of Porto, Rua Dr. Roberto Frias, 4200-465 Porto, Portugal

* Corresponding author Email: tbandosz@ccny.cuny.edu; Teresa.bandosz@mail.umcs.pl

Supporting information

Table S1 – Advantages and limitations of the most used methods to characterize the surface chemistry of porous carbons.

**Method / Description** (Selected references)	**Advantages**	**Limitations**
**X-ray Photoelectron Spectroscopy (XPS)** [1–6] Analyzes the surface chemistry by measuring the binding energies of core electrons.	• Provides the elemental surface composition for a wide range of elements (from lithium to uranium).<br>• Small changes in the chemical environment result in chemical shifts in the binding energies, which allow for the identification and quantification of the different surface groups.<br>• It is a very sensitive surface technique, exclusively analyzing the uppermost surface layers (1 to 10 nm).<br>• Sputtering allows for the analysis of composition changes with depth.<br>• Several software and databases are available to aid in the interpretation and analysis of XPS spectra, facilitating the	• Instruments and their respective maintenance are expensive.<br>• The technique requires high vacuum conditions, which are usually quite different from those used in the applications of carbon materials, and a rearrangement of the surface chemistry can occur.<br>• Samples often require extensive preparation to avoid contamination and, for non-conductive samples, charging effects.<br>• It only analyzes the very top layer (ca. 10 nm depth), which can be different from the bulk material.<br>• XPS spectra interpretation can be complex, requiring

	identification and quantification of specific groups.	significant expertise and reference data.<br>• It can be time-consuming, mainly if depth profiling and high-resolution scans are considered.<br>• Detection of light elements such as hydrogen is not possible.<br>• If depth profiling is considered, sputtering can originate changes in chemical states, making the interpretation difficult.
**Fourier Transform Infrared Spectroscopy (FTIR)** [7–10]<br>Measures the absorption of infrared radiation by the material, identifying functional groups.	• FTIR can be effective at identifying specific functional groups.<br>• The technique is non-destructive.<br>• Minimal sample preparation is needed and time of analysis is fast.<br>• Both qualitative and semi-quantitative information on the surface groups can be obtained<br>• The penetration depth (a few micrometers) is deeper than in XPS and gives information more representative of the bulk material.<br>• It can be used (with appropriate cell design) for in situ studies to observe changes in surface chemistry during processes like adsorption or catalysis.	• Limited to functional groups with IR-active vibrations.<br>• Carbon materials are black and absorb most of the IR irradiation, resulting in low signal-to-noise ratios; in some cases, samples need to be diluted with IR-transparent compounds (e.g., KBr), which adds additional preparation steps and potential errors.<br>• It can be challenging to distinguish the different surface groups when their absorption bands overlap.<br>• Careful handling is required to avoid interferences due to moisture and other atmospheric contaminants.<br>• Accurate quantitative analysis is very challenging for porous carbons.
**Temperature Programmed Desorption (TPD)**[10–12]<br>Measures the amount and type of gases released from the carbon surface as a function of temperature.	• Can identify different types of active sites on the carbon surface based on the type and desorption temperature of specific gases.<br>• Provides quantitative data on the amount of gas evolved, which is related to the number of surface sites; deconvolution methodologies allow for their quantification.	• Analyzing the TPD data can be challenging.<br>• Overlapping desorption peaks can make it difficult to distinguish between different species or functional groups.<br>• Temperature must be controlled with precision; otherwise, the desorption profiles can be affected.<br>• The experimental conditions (e.g., carrier flow rates and

	• TPD is well established for oxygen-containing surface groups.<br>• Offers insights into the thermal stability of surface functional groups.<br>• Provides information on the kinetics of desorption, including activation energies for desorption processes.	heating ramps) must be optimized to avoid side reactions.<br>• TPD can only detect surface groups that originate gas species upon heating.<br>• The high temperatures applied during the TPD experiment can provoke side reactions that may change the surface chemistry; temperatures higher than those used in the synthesis of the materials should be avoid.
**Chemical Titrations** [13–15]<br>Quantify the amount of specific surface groups using selective titrants.	• It provides quantitative information on specific surface groups, mainly for acidic ones.<br>• Titration methods are relatively inexpensive compared to advanced characterization techniques.<br>• It is a straightforward technique that does not require expensive equipment.<br>• Titrants targeting carboxylic acids, phenols, lactones or carbonyl groups are already established.	• It may not be able to detect surface groups present at very low concentrations.<br>• Surface groups present in the micropores may not be quantified.<br>• Overestimation or underestimation of the surface groups may occur due to the presence of non specific reactions with the titrant.<br>• The technique only applies to surface groups that react with the available titrants.
**Potentiometric Titrations** [16–19]<br>Quantifies surface acidic or basic groups by monitoring electrode potential changes during titrant addition.	• Provide precise quantification and pKa of acidic and basic functional groups on the surface of carbon materials.<br>• This technique can determine the pKa values of surface groups, given information about the strength and distribution of acidic and basic sites.<br>• Compared to simple titrations: they are usually more sensitive, allowing for the detection of a broader range of functional groups; they can be carried out automatically, reducing human error and increasing reproducibility.	• The titration curves can be difficult to interpret, particularly for materials that have a wide range of functional groups and pKa values.<br>• The titration process can be time-consuming, particularly for detailed analyses requiring multiple titrants or conditions.<br>• Similar to what happens in simple chemical titrations, surface groups present in small micropores may not be quantified due to diffusional effects.

**CHNS-O Elemental Analysis** [9,20]<br>Quantifies the overall elemental composition (e.g., C, H, N, O, S) of the material.	• Measures the bulk composition, giving an overall elemental distribution in the material.<br>• Specifically designed for detecting light elements (C, H, N, S, O) that are critical in carbon materials.	• Does not provide information on the chemical state or functional groups.<br>• Provides bulk composition without information on the spatial distribution of elements.
**Energy Dispersive X-ray Spectroscopy (EDX)** [21,22]<br>Integrated with SEM/TEM for elemental analysis via X-ray emission	• Can detect a wide range of elements, from boron (often carbon on standard systems) to uranium, including metals and heavy elements.<br>• Relatively fast analysis with minimal sample preparation; it can be used alongside SEM/TEM.<br>• Provides elemental mapping, showing the spatial distribution of elements across the surface.	• EDX is unable to detect hydrogen and exhibits low sensitivity to light elements, such as C, N, or O, making their quantification more challenging and prone to inaccuracy.<br>• It only provides qualitative or semi-quantitative results, contrasting with the precise quantitative data from CHNS-O analysis.<br>• It does not give information about the chemical state (oxidation/bonding) of the elements.
**X-ray Absorption Fine Structure (XAFS, including XANES and EXAFS)** [23–26]<br>Measures X-ray absorption at energies near an element's absorption edge to reveal that element's oxidation state and local atomic environment.	• Provides element-specific insights by targeting the X-ray absorption edge of a selected element (e.g., N, O, or metal dopants in carbon).<br>• Determines oxidation states via XANES features, helping identify functional group types or metal valence in the carbon material.<br>• It has been especially useful to confirm the presence of single atoms on carbon materials.<br>• Penetrating X-rays probe the bulk of the material.<br>• Can be performed under in situ or operando conditions (with appropriate cell design) to monitor changes in surface chemistry during reactions.<br>• It provides information about light (C, N, O K-edges) and heavy elements, enabling the	• Access to synchrotron radiation or specialized X-ray sources is required, making this technique less accessible and more expensive than standard laboratory equipment (e.g., XPS, FTIR, Raman).<br>• Data analysis is complex; EXAFS generally requires advanced modeling/fitting, and XANES often needs reference spectra and/or theoretical calculations.<br>• Lacks spatial resolution in standard form.<br>• Limited sensitivity to very low-concentration elements or dilute functional groups.<br>• Soft X-ray edges (e.g., C, N, O) require high vacuum and thin samples to avoid self-absorption effects.

	analysis of dopants or supported metals in carbons.	
**Solid-State Nuclear Magnetic Resonance (NMR) Spectroscopy**[27–32]<br>Uses nuclear magnetic resonance of nuclei (e.g., ^{13}C, ^{1}H, ^{31}P, ^{19}F) in solid carbon materials to reveal local chemical environments and functional groups.	• Identifies specific functional groups on carbon surfaces (e.g., carboxyl, phenolic, lactone) by their distinct chemical shift signatures in ^{13}C NMR spectra.<br>• Provides insight into the structure of the carbon matrix, including the degree of aromaticity and aromatic ring clustering.<br>• ^{13}C magic-angle spinning (MAS) NMR or ^{1}H–^{13}C cross-polarization (CP) MAS NMR can distinguish carbon atoms bonding to different O-containing surface groups, aliphatic carbon, and sp^2 carbon in graphene planes.<br>• It can probe other nuclei, such as ^{15}N or ^{31}P, in doped carbons, thereby providing a broader picture of surface chemistry when heteroatoms are present.	• Low sensitivity for nuclei at natural abundance (especially ^{13}C at ~1% abundance) often requires long acquisition times or the use of special techniques to detect surface functional groups.<br>• Instrumentation is expensive and specialized (e.g., high-field magnets, magic-angle spinning probes) and may not be readily available in all laboratories.<br>• Disordered carbons typically present overlapping peaks originating broad spectra, which are difficult to resolve and assign to specific functional group signals.<br>• The presence of unpaired electrons or magnetic impurities, which is common in activated carbons or carbon-supported catalysts, can broaden or suppress NMR signals.<br>• Data interpretation demands a high level of expertise; assigning NMR peaks to specific functional groups or structures can be challenging for carbon materials.
**Raman Spectroscopy**[1,33–37]<br>Uses inelastic light scattering (Raman effect) to probe vibrational modes of the carbon materials, giving information about their bonding structures (e.g., the disorder-related D-band and graphitic G-band).	• Non-destructive and fast technique with minimal sample preparation.<br>• Reveals carbon structural order and defects: the G-band (c.a. 1580 cm^{-1}) indicates graphitic sp^2 carbon domains, while the intensity of the D-band (c.a. 1350 cm^{-1}) reflects defects or functionalized edges.<br>• Useful for comparing the degree of graphitization or disorder among samples (e.g., via the D/G intensity ratio), which is related to the amount of edge sites and surface defects.	• It does not provide quantitative elemental or functional group concentrations.<br>• Raman spectra of porous carbons often show broad, overlapping D and G bands, making it difficult to distinguish between specific types of defects.<br>• There is a risk of sample heating or damage under the laser, especially for highly absorbent carbons; excessive laser power can change the surface or cause thermal decomposition during analysis.

	• Micro-Raman offers high lateral resolution (c.a. 1 μm), enabling the mapping of structural heterogeneity across a carbon sample.<br>• Measurements can be performed at ambient conditions and even in situ (e.g., during heating or gas exposure, with appropriate cell design) to monitor changes in real time.	
**Time-of-Flight Secondary Ion Mass Spectrometry (TOF-SIMS)** [38–40]<br>The carbon surface is bombarded with a focused primary ion beam and the mass of the secondary ions ejected are analyzed, providing detailed molecular and elemental information about the outermost surface layers.	• Highly sensitive for the outermost surface chemistry since it only probes the uppermost monolayers (c.a. 1 to 2 nm) of the material.<br>• Detects specific fragment ions and molecular species, resulting from the ion beam bombardment of specific functional groups or adsorbates on the surface.<br>• Very low detection limits for trace elements and compounds on the surface, in the range of ppm-ppb levels.<br>• Allows spatially resolved analysis, originating chemical maps with high lateral resolution (down to c.a. 100 nm); it can be paired with a secondary beam (dual sputter gun) to perform a depth profiling analysis.<br>• It can detect all elements, including hydrogen and isotopes.	• It is a destructive technique since the ion beam sputters material away from the surface of the analyzed area.<br>• ToF-SIMS instrumentation is available only in specialised laboratories due to its high cost and the need for skilled operators.<br>• This technique is generally not quantitative; the quantitative analysis is challenging due to matrix effects (ion yield depends on the surrounding material) and a calibration is needed for each material.<br>• The analysis of the resulting mass spectra is complex, as a large number of fragment peaks is obtained, which requires significant expertise and reference databases to accurately assign peaks to specific functional groups or compounds.
**Electron Paramagnetic Resonance (EPR)** [41–43]<br>Observation of the energy required to reverse the direction of an electron spin in the presence of an external magnetic field. It is based on	• EPR measurement gives the spin population concentration in the bulk sample.<br>• EPR signals can be related to conduction electrons or localized paramagnetic states in carbon-centered radicals (reactivity, aromatic π radicals) and defective carbon structures (e.g., dangling bonds with terminating O-/N-groups, trapped pi	• The EPR signal is strongly dependent on adsorbed species on the surface of the material; thus the preparation of the sample is critical (particularly in highly porous carbons).<br>• Contact with atmospheric oxygen should be avoided (strict degassing - vacuum and temperature- and sealing conditions).

the absorption of microwave electromagnetic radiation by a paramagnetic sample placed in a static magnetic field.	electrons, vacancies, zigzag states).<br>• Relaxation rates and signal shapes can be modified by the surface chemistry or adsorption in the case of porous, large-surface-area samples (e.g., activated carbon).<br>• Powerful tool to investigate paramagnetic intermediates in reactions through radical mechanisms.	• EPR is sensitive only to paramagnetic (ferro/antiferromagnetic) states, thus it needs to be complemented by other characterization techniques to comprehensively probe the nature of the surface states.<br>• Relaxation times of certain short-lived paramagnetic species may be too short for typical EPR timescale (seconds), requiring the use of spin-trapping agents to form stable paramagnetic adducts.
**Magnetic susceptibility**[44]<br>Measure of the magnetization of a material under an applied magnetic field (alignment extent with the magnetic field); diamagnetic materials (negative alignment) and paramagnetic (positive alignment)	• The spin–spin and spin–lattice relaxation times are affected by the surface oxidation/reduction state and the conductivity, thus it can detect physicochemical and structural transformations of carbon materials (e.g., thermal treatment, electrochemical polarization, photochemical exposure, surface functionalization) by probing the unpaired electron density).<br>• It can provide an excellent check on the presence of magnetic impurities in carbons.	• Sensitivity at ambient temperature may not be sufficient, making the use of cryogenic temperatures recommended to obtain good resolution.<br>• Higher spin–lattice relaxation rates are observed in samples with high carbon content and structural order.<br>• Relaxation mechanisms are not clearly understood, as they can be related to several factors: phonons, conduction electrons, spin–spin, spin-lattice relaxation.
**Electron Microscopy tomography (TEMt, SEMt, FIB-SEM-3D)** [45,46]<br>Tomography analysis includes sample processing, imaging analysis, and quantitative 3D reconstruction.	• Coupling with EDS, EBSD and EELS allows gathering of chemical, crystallographic and topological data.<br>• FIB-SEM: suited for the characterization of micron and submicron scale to a minimum resolution of 10–15 nm.<br>• SEMt is very well suited for the analysis of pore space within a few nm to a few hundred nm size.<br>• TEMt provides distribution of elements with different contrast (particularly metallic particles) in the matrix.	• SEMt: only pore and solid spaces can be distinguished.<br>• TEMt does not provide porosity information.<br>• FIB-SEM: difficult to image structures smaller than 5–10 nm.<br>• Contrast of elements is needed, making it adequate for metallic functionalization but less adequate for heteroatoms.

**Ion Beam Analysis Techniques: Rutherford backscattering spectroscopy (RBS), nuclear reaction analysis (NRA)** [47–49] A beam of energy ions is impinged on the sample and the backscattering of the beam is measured and related to the structure and composition. RBS is based on elastic Coulomb scattering between the beam and the target nuclei; NRA is based on element specific nuclear reactions.	• Useful to quantify light elements (C,H,O,N, B,F)<br>• Depth distribution can be obtained.<br>• RBS is usually applied to obtain data for most, if not all, elements present in the sample.<br>• Depth resolution: a few tens of Angstrom (NRA); a few hundreds Angstrom (RBS).	• High energy Ion Beams accelerators are needed.<br>• Thin films.<br>• Sensitivity and depth resolution can be compromised in heavy matrices.<br>• Application is limited by radiation /thermal damage of the sample.
**Electrochemical Characterization Techniques (e.g., voltammetric techniques, impedance spectroscopy and others)**[50–52] Measurement of the response of a material under controlled polarization conditions (applied electric current) or the reverse process in which an electric current is generated by a chemical reaction (batteries).	• Capacitive (surface area) and pseudocapacitive (functionalization) contributions can be differentiated upon the operating conditions (electrolyte, potential window) and the selection of the technique.<br>• The electrochemical response is very sensitive to adsorbed species and applied conditions.	• The electrochemical response is very dependent on several factors (e.g., electrode support, type of electrolyte, redox probes, impurities), which difficult straightforward comparison.<br>• Only electrochemically active moieties are detected.<br>• Electrochemical activation of carbon electrodes may affect the distribution of active sites and provoke the generation /modification of surface groups.

References

(1) Ania, C. O.; Armstrong, P. A.; Bandosz, T. J.; Beguin, F.; Carvalho, A. P.; Celzard, A.; Frackowiak, E.; Gilarranz, M. A.; László, K.; Matos, J.; Pereira, M. F. R. Engaging Nanoporous Carbons in “beyond Adsorption” Applications: Characterization, Challenges and Performance. *Carbon* **2020**, *164*, 69–84. https://doi.org/10.1016/j.carbon.2020.03.056.

(2) Desimoni, E.; Casella, G. I.; Morone, A.; Salvi, A. M. XPS Determination of Oxygen-containing Functional Groups on Carbon-fibre Surfaces and the Cleaning of These Surfaces. *Surface & Interface Analysis* **1990**, *15* (10), 627–634. https://doi.org/10.1002/sia.740151011.

(3) Desimoni, E.; Casella, G. I.; Cataldi, T. R. I.; Salvi, A. M.; Rotunno, T.; Di Croce, E. Remarks on the Surface Characterization of Carbon Fibres. *Surface & Interface Analysis* **1992**, *18* (8), 623–630. https://doi.org/10.1002/sia.740180809.

(4) Blume, R.; Rosenthal, D.; Tessonnier, J.; Li, H.; Knop-Gericke, A.; Schlögl, R. Characterizing Graphitic Carbon with X-ray Photoelectron Spectroscopy: A Step-by-Step Approach. *ChemCatChem* **2015**, *7* (18), 2871–2881. https://doi.org/10.1002/cctc.201500344.

(5) Morgan, D. J. Comments on the XPS Analysis of Carbon Materials. *C* **2021**, *7* (3), 51. https://doi.org/10.3390/c7030051.

(6) Castle, J. E. Practical Surface Analysis by Auger and X-ray Photoelectron Spectroscopy. D. Briggs and M. P. Seah (Editors). John Wiley and Sons Ltd, Chichester, 1983, 533 Pp., £44.50. *Surface & Interface Analysis* **1984**, *6* (6), 302–302. https://doi.org/10.1002/sia.740060611.

(7) Otake, Y.; Jenkins, R. G. Characterization of Oxygen-Containing Surface Complexes Created on a Microporous Carbon by Air and Nitric Acid Treatment. *Carbon* **1993**, *31* (1), 109–121. https://doi.org/10.1016/0008-6223(93)90163-5.

(8) Biniak, S.; Szymański, G.; Siedlewski, J.; Świątkowski, A. The Characterization of Activated Carbons with Oxygen and Nitrogen Surface Groups. *Carbon* **1997**, *35* (12), 1799–1810. https://doi.org/10.1016/S0008-6223(97)00096-1.

(9) Figueiredo, J. L.; Pereira, M. F. R.; Freitas, M. M. A.; Órfão, J. J. M. Modification of the Surface Chemistry of Activated Carbons. *Carbon* **1999**, *37* (9), 1379–1389. https://doi.org/10.1016/S0008-6223(98)00333-9.

(10) Kohl, S.; Drochner, A.; Vogel, H. Quantification of Oxygen Surface Groups on Carbon Materials via Diffuse Reflectance FT-IR Spectroscopy and Temperature Programmed Desorption. *Catalysis Today* **2010**, *150* (1–2), 67–70. https://doi.org/10.1016/j.cattod.2009.05.016.

(11) Herold, F.; Gläsel, J.; Etzold, B. J. M.; Rønning, M. Can Temperature-Programmed Techniques Provide the Gold Standard for Carbon Surface Characterization? *Chem. Mater.* **2022**, *34* (19), 8490–8516. https://doi.org/10.1021/acs.chemmater.2c02449.

(12) Ishii, T.; Kyotani, T. Temperature Programmed Desorption. In *Materials Science and Engineering of Carbon*; Elsevier, 2016; pp 287–305. https://doi.org/10.1016/b978-0-12-805256-3.00014-3.

(13) Boehm, H. P. Chemical Identification of Surface Groups. In *Advances in Catalysis*; Eley, D. D., Pines, H., Weisz, P. B., Eds.; Elsevier, 1966; pp 179–274. https://doi.org/10.1016/s0360-0564(08)60354-5.

(14) Boehm, H. P. Some Aspects of the Surface Chemistry of Carbon Blacks and Other Carbons. *Carbon* **1994**, *32* (5), 759–769. https://doi.org/10.1016/0008-6223(94)90031-0.

(15) Schönherr, J.; Buchheim, J.; Scholz, P.; Adelhelm, P. Boehm Titration Revisited (Part I): Practical Aspects for Achieving a High Precision in Quantifying Oxygen-Containing Surface Groups on Carbon Materials. *C* **2018**, *4* (2), 21. https://doi.org/10.3390/c4020021.
(16) Noh, J. S.; Schwarz, J. A. Effect of HNO3 Treatment on the Surface Acidity of Activated Carbons. *Carbon* **1990**, *28* (5), 675–682. https://doi.org/10.1016/0008-6223(90)90069-B.
(17) Contescu, A.; Contescu, C.; Putyera, K.; Schwarz, J. A. Surface Acidity of Carbons Characterized by Their Continuous pK Distribution and Boehm Titration. *Carbon* **1997**, *35* (1), 83–94. https://doi.org/10.1016/S0008-6223(96)00125-X.
(18) Fuente, E.; Menéndez, J. A.; Suárez, D.; Montes-Morán, M. A. Basic Surface Oxides on Carbon Materials: A Global View. *Langmuir* **2003**, *19* (8), 3505–3511. https://doi.org/10.1021/la026778a.
(19) Zhang, Z.; Flaherty, D. W. Modified Potentiometric Titration Method to Distinguish and Quantify Oxygenated Functional Groups on Carbon Materials by pKa and Chemical Reactivity. *Carbon* **2020**, *166*, 436–445. https://doi.org/10.1016/j.carbon.2020.05.040.
(20) Kandioller, W.; Theiner, J.; Keppler, B. K.; Kowol, C. R. Elemental Analysis: An Important Purity Control but Prone to Manipulations. *Inorg. Chem. Front.* **2022**, *9* (3), 412–416. https://doi.org/10.1039/d1qi01379c.
(21) Newbury, D. E.; Ritchie, N. W. M. Performing Elemental Microanalysis with High Accuracy and High Precision by Scanning Electron Microscopy/Silicon Drift Detector Energy-Dispersive X-Ray Spectrometry (SEM/SDD-EDS). *J Mater Sci* **2015**, *50* (2), 493–518. https://doi.org/10.1007/s10853-014-8685-2.
(22) Goldstein, J. I.; Newbury, D. E.; Echlin, P.; Joy, D. C.; Lyman, C. E.; Lifshin, E.; Sawyer, L.; Michael, J. R. *Scanning Electron Microscopy and X-Ray Microanalysis: Third Edition*; Springer US: Boston, MA, 2003. https://doi.org/10.1007/978-1-4615-0215-9.
(23) Rehr, J. J.; Albers, R. C. Theoretical Approaches to X-Ray Absorption Fine Structure. *Rev. Mod. Phys.* **2000**, *72* (3), 621–654. https://doi.org/10.1103/revmodphys.72.621.
(24) Stöhr, J. *NEXAFS Spectroscopy*; Springer Series in Surface Sciences; Springer Berlin Heidelberg: Berlin, Heidelberg, 1992. https://doi.org/10.1007/978-3-662-02853-7.
(25) Wartner, G.; Müller-Hülstede, J.; Trzesniowski, H.; Wark, M.; Wagner, P.; Seidel, R. *Operando* X-Ray Absorption Spectroscopy of Fe–N–C Catalysts Based on Carbon Black and Biomass-Derived Support Materials for the ORR. *Sustainable Energy Fuels* **2024**, *8* (10), 2309–2320. https://doi.org/10.1039/d4se00342j.
(26) Qi, P.; Wang, J.; Djitcheu, X.; He, D.; Liu, H.; Zhang, Q. Techniques for the Characterization of Single Atom Catalysts. *RSC Adv.* **2022**, *12* (2), 1216–1227. https://doi.org/10.1039/d1ra07799f.
(27) *Solid-State NMR Spectroscopy Principles and Applications*, 1st ed.; Duer, M. J., Ed.; Wiley, 2001. https://doi.org/10.1002/9780470999394.
(28) Puziy, A. M.; Poddubnaya, O. I.; Socha, R. P.; Gurgul, J.; Wisniewski, M. XPS and NMR Studies of Phosphoric Acid Activated Carbons. *Carbon* **2008**, *46* (15), 2113–2123. https://doi.org/10.1016/j.carbon.2008.09.010.
(29) Diyuk, V. E.; Zaderko, A. N.; Grishchenko, L. M.; Afonin, S.; Mariychuk, R.; Boldyrieva, O. Yu.; Skryshevsky, V. A.; Kaňuchová, M.; Lisnyak, V. V. Surface Chemistry of Fluoroalkylated Nanoporous Activated Carbons: XPS and 19F NMR Study. *Appl Nanosci* **2022**, *12* (3), 637–650. https://doi.org/10.1007/s13204-021-01717-7.

(30) Szewczyk, I.; Rokicińska, A.; Michalik, M.; Chen, J.; Jaworski, A.; Aleksis, R.; Pell, A. J.; Hedin, N.; Slabon, A.; Kuśtrowski, P. Electrochemical Denitrification and Oxidative Dehydrogenation of Ethylbenzene over N-Doped Mesoporous Carbon: Atomic Level Understanding of Catalytic Activity by ^{15}N NMR Spectroscopy. *Chem. Mater.* **2020**, *32* (17), 7263–7273. https://doi.org/10.1021/acs.chemmater.0c01666.

(31) Chen, Z.; Jaworski, A.; Chen, J.; Budnyak, T. M.; Szewczyk, I.; Rokicińska, A.; Dronskowski, R.; Hedin, N.; Kuśtrowski, P.; Slabon, A. Graphitic Nitrogen in Carbon Catalysts Is Important for the Reduction of Nitrite as Revealed by Naturally Abundant^{15}N NMR Spectroscopy. *Dalton Trans.* **2021**, *50* (20), 6857–6866. https://doi.org/10.1039/d1dt00658d.

(32) Ando, H.; Suzuki, K.; Kaji, H.; Kambe, T.; Nishina, Y.; Nakano, C.; Gotoh, K. Dynamic Nuclear Polarization – Nuclear Magnetic Resonance for Analyzing Surface Functional Groups on Carbonaceous Materials. *Carbon* **2023**, *206*, 84–93. https://doi.org/10.1016/j.carbon.2023.02.010.

(33) Ferrari, A. C.; Robertson, J. Interpretation of Raman Spectra of Disordered and Amorphous Carbon. *Phys. Rev. B* **2000**, *61* (20), 14095–14107. https://doi.org/10.1103/physrevb.61.14095.

(34) Yuan, R.; Guo, Y.; Gurgan, I.; Siddique, N.; Li, Y.-S.; Jang, S.; Noh, G. A.; Kim, S. H. Raman Spectroscopy Analysis of Disordered and Amorphous Carbon Materials: A Review of Empirical Correlations. *Carbon* **2025**, *238*, 120214. https://doi.org/10.1016/j.carbon.2025.120214.

(35) Dresselhaus, M. S.; Jorio, A.; Saito, R. Characterizing Graphene, Graphite, and Carbon Nanotubes by Raman Spectroscopy. *Annu. Rev. Condens. Matter Phys.* **2010**, *1* (1), 89–108. https://doi.org/10.1146/annurev-conmatphys-070909-103919.

(36) Zhang, P.; Fan, J.; Wang, Y.; Dang, Y.; Heumann, S.; Ding, Y. Insights into the Role of Defects on the Raman Spectroscopy of Carbon Nanotube and Biomass-Derived Carbon. *Carbon* **2024**, *222*, 118998. https://doi.org/10.1016/j.carbon.2024.118998.

(37) Liu, X.; Choi, J.; Xu, Z.; Grey, C. P.; Fleischmann, S.; Forse, A. C. Raman Spectroscopy Measurements Support Disorder-Driven Capacitance in Nanoporous Carbons. *J. Am. Chem. Soc.* **2024**, *146* (45), 30748–30752. https://doi.org/10.1021/jacs.4c10214.

(38) Lockyer, N. P.; Aoyagi, S.; Fletcher, J. S.; Gilmore, I. S.; Van Der Heide, P. A. W.; Moore, K. L.; Tyler, B. J.; Weng, L.-T. Secondary Ion Mass Spectrometry. *Nat Rev Methods Primers* **2024**, *4* (1). https://doi.org/10.1038/s43586-024-00311-9.

(39) Graham, D. J.; Gamble, L. J. Back to the Basics of Time-of-Flight Secondary Ion Mass Spectrometry of Bio-Related Samples. I. Instrumentation and Data Collection. *Biointerphases* **2023**, *18* (2). https://doi.org/10.1116/6.0002477.

(40) Mei, H.; Laws, T. S.; Terlier, T.; Verduzco, R.; Stein, G. E. Characterization of Polymeric Surfaces and Interfaces Using Time-of-Flight Secondary Ion Mass Spectrometry. *Journal of Polymer Science* **2022**, *60* (7), 1174–1198. https://doi.org/10.1002/pol.20210282.

(41) Wagoner, G. Spin Resonance of Charge Carriers in Graphite. *Phys. Rev.* **1960**, *118* (3), 647–653. https://doi.org/10.1103/physrev.118.647.

(42) Cheng, H. N.; Wartelle, L. H.; Klasson, K. T.; Edwards, J. C. Solid-State NMR and ESR Studies of Activated Carbons Produced from Pecan Shells. *Carbon* **2010**, *48* (9), 2455–2469. https://doi.org/10.1016/j.carbon.2010.03.016.

(43) Agrachev, M.; Giulimondi, V.; Surin, I.; Mitchell, S.; Jeschke, G.; Pérez-Ramírez, J. Electron Paramagnetic Resonance Spectroscopy for the Analysis of Single-Atom Catalysts. *Chem Catalysis* **2024**, *4* (12), 101136. https://doi.org/10.1016/j.checat.2024.101136.

(44) Tomaszewski, D.; Tadyszak, K. Electron Spin Relaxation in Carbon Materials. *Materials* **2022**, *15* (14), 4964. https://doi.org/10.3390/ma15144964.

(45) Thiele, S.; Fürstenhaupt, T.; Banham, D.; Hutzenlaub, T.; Birss, V.; Ziegler, C.; Zengerle, R. Multiscale Tomography of Nanoporous Carbon-Supported Noble Metal Catalyst Layers. *Journal of Power Sources* **2013**, *228*, 185–192. https://doi.org/10.1016/j.jpowsour.2012.11.106.

(46) Nan, N.; Wang, J. FIB-SEM Three-Dimensional Tomography for Characterization of Carbon-Based Materials. *Advances in Materials Science and Engineering* **2019**, *2019*, 1–8. https://doi.org/10.1155/2019/8680715.

(47) Jeynes, J. C. G.; Jeynes, C.; Kirkby, K. J.; Rümmeli, M.; Silva, S. R. P. RBS/EBS/PIXE Measurement of Single-Walled Carbon Nanotube Modification by Nitric Acid Purification Treatment. *Nuclear Instruments and Methods in Physics Research Section B: Beam Interactions with Materials and Atoms* **2008**, *266* (8), 1569–1573. https://doi.org/10.1016/j.nimb.2007.12.105.

(48) Touhara, H.; Okino, F. Property Control of Carbon Materials by Fluorination. *Carbon* **2000**, *38* (2), 241–267. https://doi.org/10.1016/s0008-6223(99)00140-2.

(49) Braun, M. Surface Analysis by RBS and NRA. *Vacuum* **1984**, *34* (12), 1045–1052. https://doi.org/10.1016/0042-207x(84)90222-7.

(50) Carbó, A. D. *Electrochemistry of Porous Materials*, 2nd ed.; CRC Press: Boca Raton, 2021. https://doi.org/10.1201/9780429351624.

(51) Chauhan, R.; Fogel, R.; Purcarea, C.; Necula-Petrareanu, G.; Fanjul-Bolado, P.; Ibañez, D.; Vasilescu, A.; Banciu, R. M.; Limson, J. Electrochemical Characterization of Carbon Black in Different Redox Probes and Their Application in Electrochemical Sensing. *Carbon Trends* **2024**, *17*, 100408. https://doi.org/10.1016/j.cartre.2024.100408.

(52) Kasuk, K.-A.; Nerut, J.; Grozovski, V.; Lust, E.; Kucernak, A. Design and Impact: Navigating the Electrochemical Characterization Methods for Supported Catalysts. *ACS Catal.* **2024**, *14* (16), 11949–11966. https://doi.org/10.1021/acscatal.4c03271.
